# miRVine: a microRNA expression atlas of grapevine based on small RNA sequencing

**DOI:** 10.1186/s12864-015-1610-5

**Published:** 2015-05-16

**Authors:** Jayakumar Belli Kullan, Daniela Lopes Paim Pinto, Edoardo Bertolini, Marianna Fasoli, Sara Zenoni, Giovanni Battista Tornielli, Mario Pezzotti, Blake C. Meyers, Lorenzo Farina, Mario Enrico Pè, Erica Mica

**Affiliations:** Institute of Life Sciences, Scuola Superiore Sant’Anna, Piazza Martiri della Libertà 33, 56127 Pisa, Italy; Department of Biotechnology, University of Verona, Strada Le Grazie 15, 37134 Verona, Italy; Department of Plant and Soil Sciences, University of Delaware, 15 Innovation Way, 19711 Newark, DE USA; Department of Computer, Control and Management Engineering, University of Rome “La Sapienza”, Via Ariosto 25, 00185 Rome, Italy; Genomics Research Centre, Consiglio per la Ricerca in Agricoltura e l’Analisi dell’Economia Agraria, Via S. Protaso 302, 29017 Fiorenzuola d’Arda (PC), Italy

**Keywords:** Grapevine, microRNAs, Deep Sequencing, RT-qPCR, Berries, Inflorescences, Plant development, Ethylene biosynthesis

## Abstract

**Background:**

miRNAs are the most abundant class of small non-coding RNAs, and they are involved in post-transcriptional regulations, playing a crucial role in the refinement of genetic programming during plant development. Here we present a comprehensive picture of miRNA regulation in *Vitis vinifera* L. plant during its complete life cycle. Furthering our knowledge about the post-transcriptional regulation of plant development is fundamental to understand the biology of such an important crop.

**Results:**

We analyzed 70 small RNA libraries, prepared from berries, inflorescences, tendrils, buds, carpels, stamens and other samples at different developmental stages. One-hundred and ten known and 175 novel miRNAs have been identified and a wide grapevine expression atlas has been described. The distribution of miRNA abundance reveals that 22 novel miRNAs are specific to stamen, and two of them are, interestingly, involved in ethylene biosynthesis, while only few miRNAs are highly specific to other organs. Thirty-eight miRNAs are present in all our samples, suggesting a role in key regulatory circuit. On the basis of miRNAs abundance and distribution across samples and on the estimated correlation, we suggest that miRNA expression define organ identity. We performed target prediction analysis and focused on miRNA expression analysis in berries and inflorescence during their development, providing an initial functional description of the identified miRNAs.

**Conclusions:**

Our findings represent a very extensive miRNA expression atlas in grapevine, allowing the definition of how the spatio-temporal distribution of miRNAs defines organ identity. We describe miRNAs abundance in specific tissues not previously described in grapevine and contribute to future targeted functional analyses. Finally, we present a deep characterization of miRNA involvement in berry and inflorescence development, suggesting a role for miRNA-driven hormonal regulation.

**Electronic supplementary material:**

The online version of this article (doi:10.1186/s12864-015-1610-5) contains supplementary material, which is available to authorized users.

## Background

The completion of the grapevine (*Vitis vinifera* L.) genome sequence [1] and subsequent research to elucidate gene function and molecular mechanisms involved in plant development, yield and quality represent a milestone in the genomics of fruit tree species. Transcriptomic and functional analyses have greatly contributed to the description of the gene regulatory network underlying grapevine development [2–6] with a particular focus on berry ripening, due to its relevance in wine production and fresh fruit consumption. Vast collections of grapevine cultivars exist, making grapevine a model fruit tree species that offers the scientific community the opportunity to study genetic diversity.

Many studies have shed light on the regulatory layer mediated by small RNAs, [7–11] revealing their crucial role in the genetic programming and fine tuning of plant biology. MicroRNAs (miRNAs) are a class of small RNAs (21–24 nucleotide long) coded by specific endogenous genes, which act as negative gene regulators. miRNAs guide mRNA cleavage, translational repression and chromatin modification of corresponding target genes [12] and are involved in a variety of developmental processes, which include organ morphogenesis [7,13–16], transition from vegetative to reproductive plant growth [17,18] and responses to stress [19–21]. Other classes of small RNAs are involved in regulating genome methylation levels, and genome stability, thus keeping transposon and repetitive elements silent [22,23].

Considering the role that miRNAs and other small regulatory RNAs play in plant biology, any comprehensive genomic description should include a detailed structural and functional characterization of these regulatory molecules. It is necessary to detail the regulatory layer mediated by these molecules in grapevine, as well, not only because of its great economic importance, but also because grapevine has a complex life cycle, thus providing the opportunity to explore different developmental stages spanning a two-season period. Next generation sequencing technologies have strongly favoured the analyses of small RNA regulatory networks, detecting rare molecules and small RNA variants with unprecedented precision and sensitivity. There is a relatively large number of grapevine miRNAs deposited in miRBase (Release 20 www.mirbase.org) and/or reported in the literature [15, 24–31]. Their expression profile has been analyzed in a few critical tissues/organs, predominantly in developing berries and flowers.

Here we describe a genome-wide transcription atlas of miRNAs in grapevine, analyzing the spatio-temporal distribution of known and newly discovered miRNAs, in the widest range of grapevine samples considered thus far. The identification of targets mRNA by *in silico* prediction methods complements our characterization of the miRNA transcriptome and deepens our understanding of the biological functions of miRNAs in grapevine development.

Our aim is to establish a reference for the future development of targeted functional studies of miRNA regulation.

## Results

### Sequencing statistics

We constructed and sequenced 70 bar-coded libraries from *Vitis vinifera* L. cv. Corvina and the *V. vinifera* clone PN40024, the reference clone used for genome sequencing [1] (Table 1).

**Table 1:** Samples list of *Vitis vinifera* cv Corvina and PN40024 clone, used for small RNA libraries

Sample	Developmental stages	Replicates	Library codes	Modified E-L system stages
BUD	Latent bud	2	Bud_L	E-L 23
	Winter Bud	2	Bud_W	E-L 1
	Bud burst (green tip)	2	Bud_B	E-L 4
	Bud after burst (Rosette of leaf tips visible)	2	Bud_AB	E-L 5
LEAF	Young	2	Leaf_Y	EL-14
	Mature	2	Leaf_M	E-L 29
	Leaf-Senescence	2	Leaf_S	E-L 43
STEM	Green stem	2	Stem_G	E-L 30
	Woody stem	2	Stem_W	E-L 43
ROOT	*In vitro* cultivation	2	Root	
TENDRIL	Young (Pool of tendrils from shoot bearing 7 separated leaves)	2	Tendril_Y	E-L 14
	Well-developed (Pool of tendrils from shoot bearing 12 separated leaves)	2	Tendril_WD	E-L 17
INFLORESCENCE	Young inflorescence	2	Inflorescence_Y	E-L 14
	Well-developed inflorescence	2	Inflorescence_WD	E-L 17
FLOWER	Beginning of flowering (10% caps off)	2	Flower_FB	E-L 20
	Flowering (50% caps off)	2	Flower_F	E-L 23
STAMEN	Pool of stamens from flowers at 10% and 50% caps off	2	Stamen	E-L 20/E-L 23
POLLEN	Pollens from flowers at 80% caps off	2	Pollen	E-L 25
CARPEL	Pool of carpels from flowers at 10% and 50% caps off	2	Carpel	E-L 20/E-L 23
BERRY	Fruit set	2	Berry_FS	E-L 29
	Post fruit set	2	Berry_PFS	E-L 31
	Pre-*Veraison*	2	Berry_PV	E-L 34
	*Veraison*	2	Berry_V	E-L 35
	Pre-ripening	2	Berry_MR	E-L 36
	Ripening	2	Berry_R	E-L 38
	Post-harvest withering (1st month)	2	Berry_PHWI	
	Post-harvest withering (2nd month)	2	Berry_PHWII	
	Post-harvest withering (3rd month)	2	Berry_PHWIII	
SEED	Collected from green berries (Pool of berries from FS, PFS, PV)	1	Seed_G	
	Collected from Mature berries (Pool of berries from V, MR, R)	1	Seed_M	
RACHIS	Fruit set	2	Rachis_FS	E-L 29
	Post -fruit set	2	Rachis_PFS	E-L 31
	*Veraison*	2	Rachis_V	E-L 35
	Pre-ripening	2	Rachis_MR	E-L 36
	Ripening	2	Rachis_R	E-L 38
**Total Corvina**	**35**	**68**		
ROOT	Pool of *in vitro* roots of PN40024 clone	1	Root_PN	
LEAF	Pool of *in vitro* leaves of PN40024 clone	1	Leaf_PN	
**Total PN40024**	**2**	**2**

**Table 2:** List of miRNA targets, discussed in the paper. List of miRNA and their respective targets as identified by TARGET FINDER, and divided per plant organ. GRAPE_IGGP_v1 12X assembly - v2 annotation was used. Only the miRNA/targets pair discussed in the paper are shown. All these miRNA targets are also confirmed by psRNATarget, with the exception of VIT_210s0003g00870, VIT_216s0022g02450, VIT_216s0100g00030

Tissues	vvi-miRNA	Target	Predicted function
Inflorescence/Flowers	miR156a; miR156b-5p; miR156c-5p; miR156d-5p; miR156e; miR156f; miR156g-5p; miR156i	VIT_208s0007g06270	Squamosa promoter-binding-like protein 9-like
		VIT_211s0065g00170	Squamosa promoter-binding-like protein 12-like
		VIT_201s0010g03710	Squamosa promoter-binding-like protein 2
		VIT_201s0011g00130	Squamosa promoter-binding-like protein 6-like
		VIT_201s0010g03910	Squamosa promoter-binding-like protein 13-like
		VIT_215s0021g02290	Squamosa promoter-binding-like protein 7-like
		VIT_217s0000g01260	Squamosa promoter-binding-like protein 13
		VIT_217s0000g05020	Squamosa promoter-binding-like protein 6-like
	miR156a; miR156b-5p; miR156c-5p; miR156d-5p; miR156e	VIT_214s0068g01780	Squamosa promoter-binding-like protein 16-like
	miR156b-5p; miR156c-5p; miR156d-5p; miR156e	VIT_219s0090g01180	Uncharacterized protein
	miR156b-5p; miR156c-5p; miR156d-5p	VIT_215s0046g01020	Uncharacterized protein loc100241322
	miR156e	VIT_204s0008g02640	Plastid-lipid-associated protein 8
	miR172c-3p	VIT_213s0019g03550	AP2 domain-containing transcription factor
	miR172c-3p; miR172d	VIT_207s0031g00220	Transcription factor Apetala2
		VIT_206s0004g03590	Transcription factor Apetala2
		VIT_208s0040g03180	AP2 domain-containing transcription factor
	miR172d	VIT_200s0184g00102	AP2 domain-containing transcription factor
	miR390-5p	VIT_210s0003g01890	LRR receptor-like serine threonine-protein kinase RFK1
		VIT_216s0098g01090	Leucine-rich repeat receptor-like protein kinase PEPR1
	miR393a-5p; miR393b	VIT_214s0068g01330	LRR receptor-like serine threonine-protein kinase RFK1
		VIT_207s0104g01320	Transport inhibitor response 1
		VIT_214s0030g01240	Transport inhibitor response 1
	grape-m9269	VIT_208s0007g05240	Phosphatidylinositol-4-phosphate 5-kinase
Stamen	grape-m5380; grape-m7774	VIT_205s0049g00340	1-aminocyclopropane-1-carboxylate oxidase homolog 1
		VIT_200s1313g00010	1-aminocyclopropane-1-carboxylate oxidase homolog 1
		VIT_205s0049g00330	1-aminocyclopropane-1-carboxylate oxidase homolog 1
		VIT_205s0049g00370	1-aminocyclopropane-1-carboxylate oxidase homolog 1
		VIT_216s0022g02420	Protein
		VIT_216s0100g00030	Cucumisin-like
		VIT_216s0022g02450	Protein
		VIT_205s0049g00350	1-aminocyclopropane-1-carboxylate oxidase homolog 1
Berry development	miR319b-3p; miR319c-3p; miR319f-3p; miR319g	VIT_213s0067g01630	Transcription factor gamyb-like
		VIT_210s0003g00870	Transcription factor TCP4-like
	miR319c-5p	VIT_214s0081g00480	Protein
	miR395a; miR395c-3p; miR395d; miR395e-3p; miR395f-3p; miR395g-3p; miR395h-3p; miR395i-3p; miR395j-3p; miR395k-3p; miR395l-3p	VIT_207s0031g00940	Sulphate bicarbonate oxalate exchanger and transporter SAT-1
	miR395h-5p; miR395k-5p	VIT_214s0066g00910	Protein
	miR395n	VIT_205s0020g04210	ATP sulphurylase
	miR396a-3p	VIT_207s0031g02320	Protein
		VIT_200s0208g00090	Protein trichome birefringence-like 16
	miR396a-5p	VIT_210s0597g00040	Pentatricopeptide repeat-containing protein
		VIT_202s0025g04910	Growth-regulating factor
		VIT_215s0048g01740	Growth-regulating factor
		VIT_200s0494g00010	Transcription activator
		VIT_218s0001g08650	Uncharacterized protein loc100260890
	miR396a-5p; miR396c-5p; miR396d-5p	VIT_216s0039g01450	Growth-regulating factor 1
		VIT_209s0002g01350	Growth-regulating factor 5
		VIT_211s0016g01250	Growth-regulating factor 5
		VIT_208s0007g03760	Growth-regulating factor 4
		VIT_216s0098g01080	Protein
		VIT_202s0025g02680	Uncharacterized protein loc100258227
	miR396a-5p; miR396b-5p	VIT_207s0191g00220	Uncharacterized protein loc100251922
	miR396b-3p	VIT_201s0011g01220	Double top
	miR396c-3p	VIT_205s0077g02140	Glyoxysomal fatty acid beta-oxidation multifunctional protein MFP-A
	miR396d-3p	VIT_215s0024g00350	TATA box binding protein associated factor-like protein
	grape-m6905	VIT_213s0064g00290	bifunctional dihydroflavonol 4-reductase flavanone 4-reductase-like
		VIT_213s0047g00940	bifunctional dihydroflavonol 4-reductase flavanone 4-reductase-like
		VIT_203s0110g00350	Cinnamoyl reductase-like protein
		VIT_213s0047g00990	bifunctional dihydroflavonol 4-reductase flavanone 4-reductase-like
		VIT_213s0064g00340	Phenylacetaldehyde reductase
		VIT_213s0067g00530	bifunctional dihydroflavonol 4-reductase flavanone 4-reductase-like
		VIT_213s0067g00460	bifunctional dihydroflavonol 4-reductase flavanone 4-reductase-like
		VIT_213s0047g00540	bifunctional dihydroflavonol 4-reductase flavanone 4-reductase-like

One database was established using the grapevine genome, and made available on the website (https://mpss.udel.edu/dbs/index.php?SITE=grape_sRNA_atlas), in order to store and assist the visualization of all the sequenced libraries.

Approximately 1.26 billion small RNA raw reads were produced. Adapter sequences were recognized and trimmed from 739,396,104 raw reads which correspond to 61,883,599 unique sequences, ranging from 18 to 34 nt (Additional file 1). A total of 377,328,013 reads represented by 27,318,558 unique sequences were perfectly mapped to the *V. vinifera* PN40024 reference genome [1], excluding reads mapping to rRNA, tRNA, snRNA, and snoRNA sequences.

**Fig. 1:**
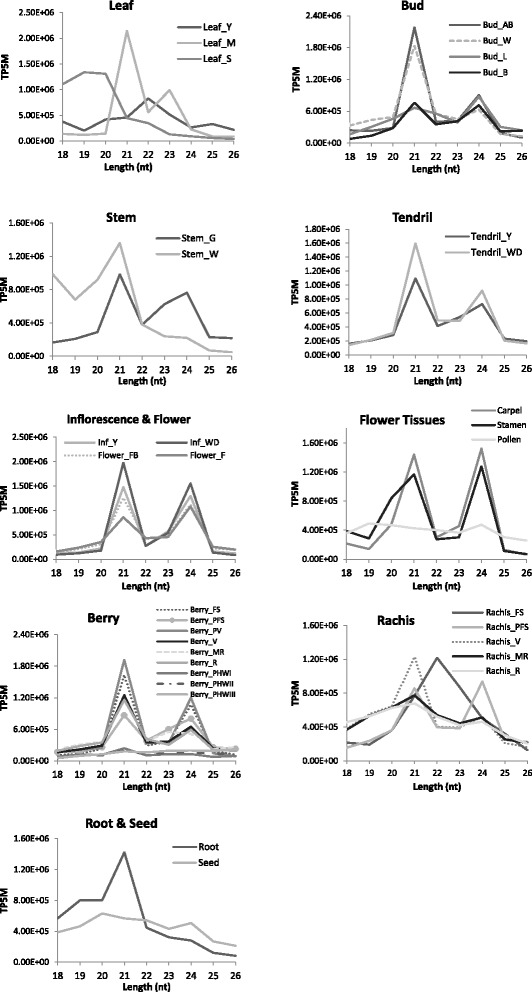
Size distribution of small RNAs from all samples of grapevine. X-axis: length in nucleotides (nt); Y-axis: summed normalized abundances for each size class as an average of two biological replicates. Library codes are indicated in Table 1

In most samples, mapped reads show a similar pattern of size distribution, with distinct peaks at 21 and 24 nt (Fig. 1), in agreement with known products of Dicer-Like (DCL) protein cleavage. In particular, the 24 nt-long molecules were the most abundant class in latent buds, flowers (Flower_FB and Flower_F) and floral organs, such as carpels and stamens. The predominance in floral tissues of the 24 nt molecules, already described in grapevine by Wang et al. [30], may reflect the shift of the small RNA population towards siRNAs during plant development, shift linked to transposons and repeated sequences. The remaining samples had the highest proportion of 21 nt-long sequences, typically represented by miRNAs. The 21 nt class showed the highest degree of redundancy, suggesting that they derive from precursors which produce well-defined short sequences, whereas the 24-nt class showed a lower redundancy, indicating their origin in loci producing populations of heterogeneous small RNA molecules. Size profiling indicated an insufficient quality for further quantitative analyses of a few libraries (Fig. 1), namely those derived from roots, leaves in senescence, young leaves, woody stems, pollen, post-harvest berries and seeds, which were used only to identify known vvi-miRNAs and to predict novel miRNA candidates, but not for the expression analysis. These libraries did not present the typical 2-peaks profile expected in angiosperms [32] for small RNA sequencing data and/or were not sufficiently abundant in terms of distinct genome matched reads (Additional file 1).

### Identification of annotated and novel grapevine miRNAs

We applied a powerful miRNA identification pipeline, developed by Jeong, Zhai and collaborators [33,34], successfully applied to several species [35–40], to our set of 70 small RNA libraries in order to identify already annotated miRNAs (known) and to discover novel candidates.

We fed the pipeline with 32,214,912 and 3,393,929 distinct small RNA sequences from the 68 libraries of cultivar Corvina and the two libraries of clone PN40024, respectively.

First, we searched for known vvi-miRNAs in the Corvina data set and identified 98 unique sequences, representing 157 out of 186 known mature vvi-miRNAs. We manually investigated all the 157 known miRNA loci identified in the Corvina libraries, checking for the existence of the correct precursor and whether the annotated miRNAs corresponded to the most abundant one. We retrieved the highest abundant sequence and, when possible, also the complementary 3p or 5p sequence. For twenty-three miRNAs the most abundant sequence appeared as a different variant (isomiR) of the annotated miRNA, showing different length or a shift of the map position in the precursor (Additional file 2). For instance, the annotated sequence of vvi-miR171b showed an abundance of only 6 TP5M, whereas its variant, which starts 3 bp downstream of the annotated one, had an abundance of 4243 TP5M. The 3 bp shift of vvi-miR171b was already observed and deposited in miRBase. A difference in length was noted for 16 vvi-miRNAs from ten families (Additional file 2). In line with Jeong et al. [34], Devers et al. [41] and Marco et al. [42], we observed cases where the complementary sequence of the annotated vvi-miRNA was more abundant than or as abundant as the annotated sequence, suggesting that complementary sequences might have biological functions. This was true for five members of the vvi-miRNA169 family, for vvi-miR398a, vvi-miR479, and vvi-miR482. For three other loci, vvi-miR159c, vvi-miR169w and vvi-miRNA3639, we found two distinct duplexes produced from the same precursor, which we designated as vvi-miR159c.2, vvi-miR169w.2 and vvi-miRNA3639.1 (Additional file 3). The sequencing reads originating from vvi-miRNA159c and vvi-miRNA169w were arranged in phase, either in tandem (vvi-miR169w) or separated by a gap of 21 nucleotides (vvi-miR159c). In contrast, the two duplexes produced by vvi-miRNA3639 had a 13 bp overlapping region, and the most sequenced reads corresponded to the not annotated reads, as observed for the vvi-miRNA169w locus.

In conclusion, we confidently identified 88 unique, known sequences, corresponding to 110 known mature vvi-miRNAs from which, 80 also had the complementary (3p or 5p) sequence identified (Additional file 2). These identified vvi-miRNAs belong to 41 conserved plant miRNA families. Seven grapevine miRNA known families (vvi-miR828, vvi-miR845, vvi-miR3626, vvi-miR3630, vvi-miR3631, vvi-miR3636 and vvi-miR3638), were not identified in our libraries.

For the identification of novel miRNAs, the previous pipeline was used with Corvina and PN40024 libraries as input. In total, 905 unique miRNA candidates generated from 1216 precursors were kept. These candidates were compared to those in miRBase identifying 45 sequences with >90% similarity to known plant miRNAs, 32 were new members of 13 known *Vitis* families, and 13 candidates were members of 10 new *Vitis* families (Additional files 4, 5, 6 and 7). After subtracting these 45 sequences, 1171 completely novel grapevine miRNA candidates were retained.

Interestingly, we found two new members of the miRNA172 family, vvi-miRC172e and vvi-miRC172f, in antisense to vvi-miR172b and vvi-miR172a respectively. The 3’ sequences produced from both antisense duplexes were predicted to target *Apetala2* (*AP2*) gene and other *AP2*-related genes, which are already well known targets of the miRNA172 family (Additional file 8). As for the 5’sequences of the duplexes, both had the DNA-directed RNA polymerase III subunit C3 as a predicted target (Additional file 8).

For the identification of completely novel miRNAs candidates, in order to prevent the selection of siRNA-like miRNAs, we selected from the list of candidates passing the pipeline’s filters, only 20, 21 and 22 bp long candidates. Their precursor secondary structures were then inspected manually (UEA sRNA toolkit, plant version [43]). Our analyses indicated that 132 novel vvi-miRNA candidates (109 from Corvina and 23 from PN40024) (Additional files 4, 5, 6 and 7) matched the criteria for miRNA annotation [44]. For ten sequences the complementary sequence (3p or 5p) was also retrieved with a high abundance, and is, hence, reported in Additional file 3. As observed in conserved miRNAs, around 45% (60 out of 132) of novel candidates began with a 5' uridine, which is a characteristic feature of miRNAs (Additional files 4 and 5).

Finally, we compared our novel miRNA candidates with those reported previously in grapevine, and not deposited in miRBase [27, 29–31] cross checking sequences, allowing some nucleotide shift, and, when available, the genomic coordinates. Only 31 miRNAs were previously identified. The mature sequences of 14 out of these candidates, were commonly retrieved in [27, 29, 30], while no sequences/precursors were in common with [31].

### *In silico* identification of target genes

We predicted the targets for all the miRNA set here identified, comprising a total of 396 miRNA sequences (considering known, and novel miRNAs and their complementary sequences when is the case, from both Corvina and PN40024 data). A total of 2068 predicted targets were retrieved using TargetFinder [45]. Only targets with a score of 3.0 were considered and further investigated (Table 2, Additional files 8 and 9). On average, for each miRNA sequence, two targets were found, but we observed several cases in which one miRNA targets up to ten distinct coding genes. Since *in silico* prediction of miRNA targets is prone to false positive targets [46], to optimize both accuracy and sensitivity we checked our results with psRNATarget, a second *in silico* pipeline [47]. Seventy-one per cent of the predicted targets shown in Additional file 8 were confirmed (data not shown).

For each predicted miRNA:target pair, we calculated the Pearson correlation between the level of expression of the miRNA (discussed in the next paragraphs) and the expression values of the target gene available from the cv. Corvina transcriptomic atlas [4]. Considering all the common sample shared between our atlas and [4], and a p-value cutoff of 0.05, we identified the miRNAs negatively correlated to their predicted targets (Additional file 10)

### Expression analyses of Corvina libraries

To evaluate the abundance of each miRNA identified in our libraries, we used the normalized abundance (TP5M) of each sequence in each library, which represents the relative cloning frequency. We considered, for further analyses, a miRNA as expressed only when both biological replicates met the set threshold of 10 TP5M. Accordingly, we eliminated only a few 3p or 5p sequences of known miRNA and nearly 40 newly predicted miRNAs that were below the set threshold. To establish the miRNA expression atlas, we used sequencing data of our Corvina libraries, discarding some samples that showed an insufficient sequencing-quality (see Sequencing Statistics for details).

**Fig. 2:**
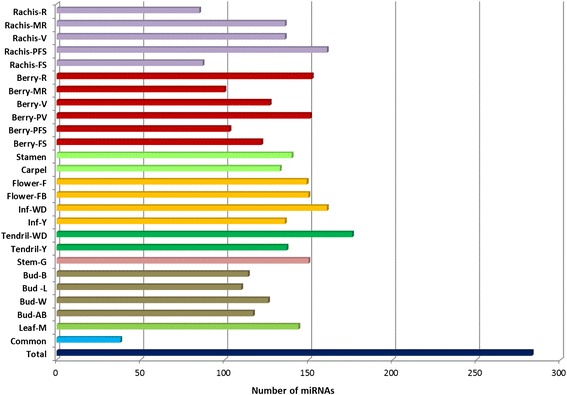
Number of miRNA expressed in 25 different grapevine samples used to define the atlas. X-axis: number of miRNAs expressed in individual samples; Y-axis: different samples. Common refers to the number of miRNA expressed in all the samples. Total is the whole set of miRNAs expressed above the set threshold of 10 TPM in both replicates

**Fig. 3:**
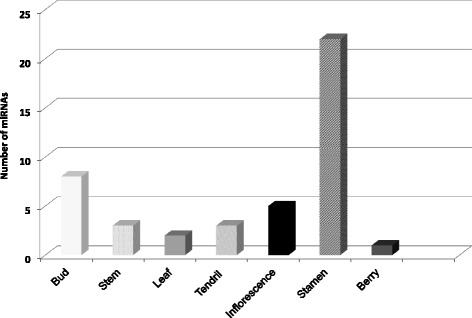
Number of organ specific miRNAs identified in Corvina derived libraries. X-axis: number of miRNAs; Y-axis: different organs

**Fig. 4:**
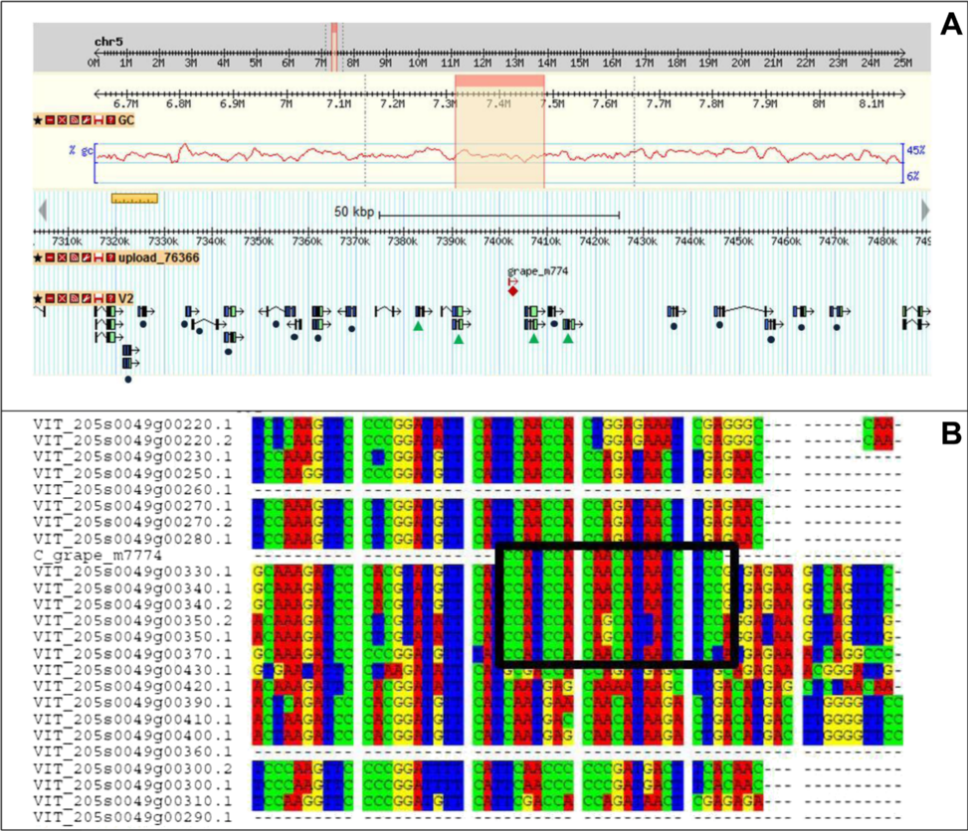
Genomic analyses of miRNA grape-m774 and its targets. **A**: The genomic region of chromosome five hosting grape-m774 and 19 genes coding for ACC oxidase, using Gbrowse (http://genomes.cribi.unipd.it). Closed diamond: miRNA; Closed dot: *ACC oxidase homolog* not target; Closed triangle: *ACC oxidase homolog* target of the miRNA. **B**: Alignment of the 19 *ACC oxidase* transcripts. When more than one transcript is present for a given gene, it is reported and indicated. The reverse complement of the miRNA is shown. Black box highlights the region of sequence similarity between miRNA and targets

Figure 2 shows the distribution of miRNAs expressed in each of the 25 different samples considered. The number of expressed miRNAs is relatively high in the 25 samples, ranging from 85 in ripening rachis to 176 in whole developed tendrils. Forty four miRNAs were classified as organ-specific (Fig. 3), *i.e.* miRNAs expressed exclusively in one organ, in at least one of its developmental stages. Stamen and buds expressed the highest number of organ-specific miRNAs. Of the 22 stamen-specific miRNAs, two have targets coding for a protein involved in TMV resistance (Additional file 8), while two (grape-m5380 and grape-m7774) have, among their targets, five genes coding for Aminocyclopropane-1-carboxylate oxidase homolog (*ACC OX* genes). Interestingly, grape-m7774 maps on chromosome five in a region where four out of the five targets map (Fig. 4). Thirty-eight miRNAs were identified as common to all samples; a part from grape-m3245, 3p and 5p sequence, all the other common miRNAs belong to known miRNA families.

**Fig. 5:**
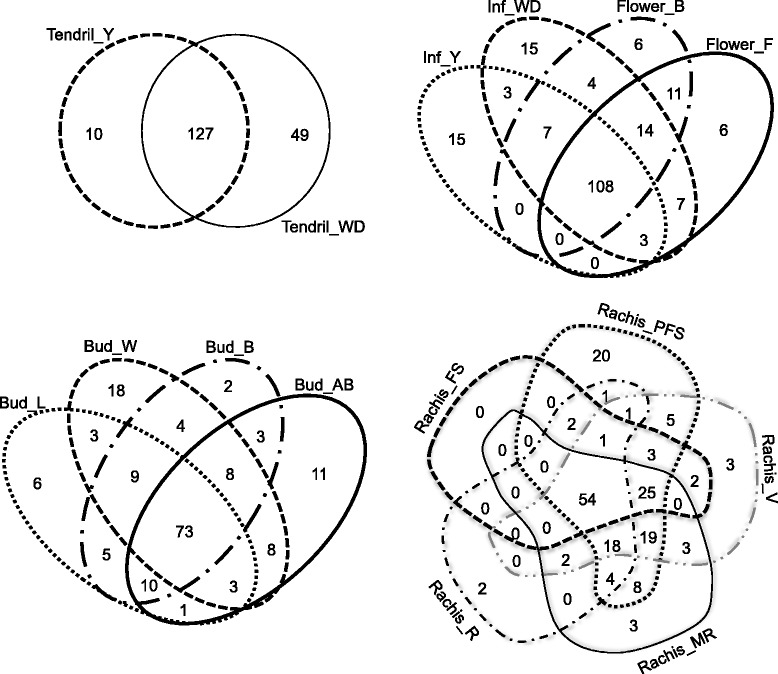
Shared miRNAs among the different developmental stage of each organ. The description of library codes is in Table 1

To deepen the analysis on miRNAs distribution during plant development, we investigated each plant structure separately (*i.e.* berries, inflorescences/flowers, tendrils, rachis and buds) considering the distinct time points sampled. miRNAs expressed in all developmental phases of a given organ range from a minimum of 30.6% (54/176) in rachis, to a maximum of 68.3% in tendrils (Fig. 5). In the six stages of berry development 74 miRNAs (40%) are in common. The number of miRNAs present in only one developmental time point is very low, when compared to the total number of miRNAs expressed in the sample.

We analyzed the top 20 expressed miRNAs in each sample (Additional file 11) to check whether the most abundant miRNAs were shared among samples. Our analysis showed that in fact highly abundant (more than 1000 TP5M) miRNAs are similar in almost all the samples, but stamen and carpels, with vvi-miR166 family being by far the most abundant (above 30,000 TP5M and reaching 1 million TP5M), followed by vvi-miR3634-3p (a grapevine specific miRNA with unknown target), by vvi-miR159c.1-3p and by the novel candidate grape-m3245. vvi-miR159c.1-3p and four different members of miR319 family (above 59,000 or 70,000 TP5M), whose 3p sequence is predicted to target TCP4-like and a GAMYB-like transcription factors, are the most expressed in stamen and carpel. vvi-miR159c.1-3p is not predicted to target a MYB transcription factor, as is usual for miR159 molecules, but a gene encoding a Lactoylglutathione Lyase protein (Glyoxalase family), putatively involved in plant tolerance to stresses.

**Fig. 6:**
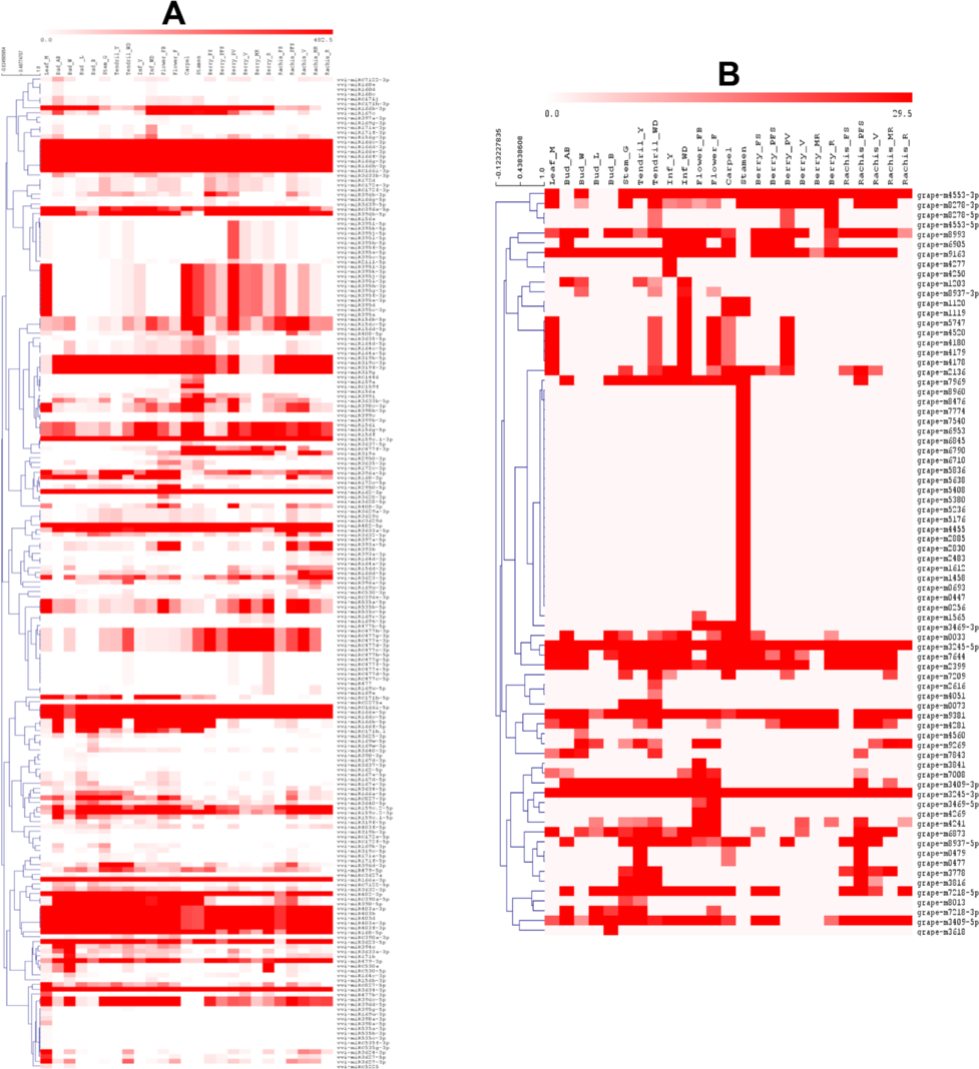
Heat map and hierarchical cluster analysis (HCL) of miRNAs identified in Corvina derived libraries. The HCL tree was generated with the average linkage clustering method. **A**: Heat map with known miRNAs including new members of known families. **B**: Heat map with novel miRNAs. miRNAs with an abundance below the set threshold of 10 TP5M in both replicates were set to zero. miRNAs that are zero in all the 25 samples were removed from these heat maps. Red and white represent high and low expression, respectively

To analyze the overall expression profile of all the miRNAs in our atlas, we represented their expression values in a heat map, subjecting the normalized count of reads to hierarchical clustering (Fig. 6). Known miRNAs (Fig. 6A), and candidate miRNAs, putatively grapevine specific (Fig. 6B), were analyzed in two different heat maps, to reduce the complexity of the figure and allow the graphical representation of these two groups of miRNAs whose average expression level is very different: above 3000 TP5M for the group of known miRNAs and below 300 TP5M for the novel miRNAs. The expression profiles of the 109 novel candidates vvi-miRNA revealed that nine novel candidates are relatively highly expressed in most of the 25 samples (grape-m8278-3p, grape-m4553-3p, grape-m3245-3p, grape-m9163, grape-m3245-5p, grape-m7644, grape-m2399, grape-m9381, grape-m8993), while the remaining candidates showed a medium to low expression level, being enriched in few samples, such as grape-m4269 and grape-m 3469-5p expressed in flowers at beginning of flowering and at full bloom (Flower_FB and Flower_F), or as grape-m4250 and grape-m4277 expressed only in young inflorescences, and grape-m4553-5p and grape-m8278-5p, expressed in well developed tendrils, berry at veraison and berry at ripening (Fig. 6B). It is striking the presence of the 22 stamen-specific miRNAs, all belonging to our novel candidate group. Differently, the heat map representing the known miRNAs (Fig. 6A) reveals a reduced level of organ/tissue specificity and a high expression of numerous families, often nearly ubiquitous. Some miRNAs are specific to a few groups of samples, such as vvi-miR395-3p, which is strongly regulated during flower and berry development, or miRC477-3p mainly expressed in berries and rachis, and miR393 a,b -5p expressed in developing rachis and flowers.

### miRNA profiles define organ identity

**Fig. 7:**
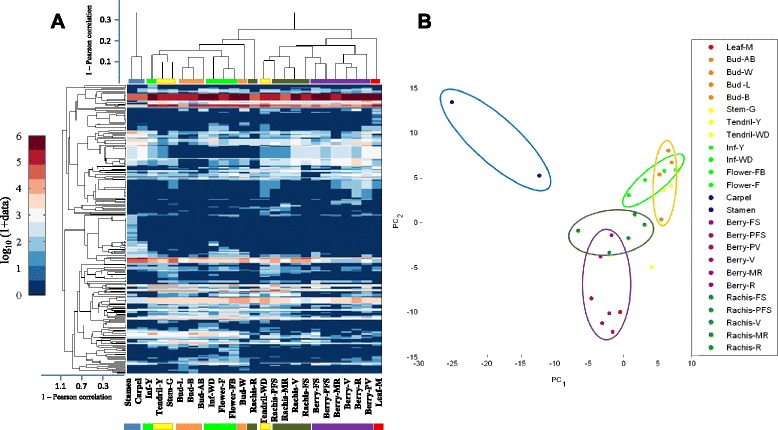
Clustergram and principal component analysis of the 25 different grapevine samples of the miRNA atlas. (**A**) The picture reports the hierarchical clustering analysis for both samples and miRNAs, the corresponding dendrogram and the data heat map. Data were normalized as described in Methods. The X-axis of each dendrogram is the 1 minus Pearson correlation distance metric. We used an optimal leaf order value which determines the leaf order that maximizes the similarity between neighbouring leaves. (**B**) A PCA analysis has been performed on the dataset. Data were preliminarily z-scored. X-axis represents the first components and the Y-axis the second component. The two components explain 34.5% of total variance. Each organ is associated to the same colour and ovals indicate clusters approximately related to organ identity. Blue for reproductive organs, purple for berries, red for leaf, orange for buds, yellow for stem and tendrils, light green for inflorescences and dark green for rachis. The same colours are used in the dendrogram. Each color represents a different cluster. One minus Pearson correlation was used as a metric distance

In order to define the relationships among the 25 different samples used in our atlas, a correlation matrix (Additional file 12), a dendrogram and a PCA (Fig. 7) were established. The distance was calculated using one minus Pearson correlation metric of normalized data, as explained in full details in Methods, to evaluate the level of similarity among the samples from the miRNA perspective. The correlation matrix identifies two main clusters (see Additional file 11): the first groups inflorescences, buds, tendrils and stem, the second includes all the developing berries and their respective rachis stages. The leaf is scarcely related to any of the samples, and far from the inflorescence/buds group. The carpel and stamen are part of a distinct group not correlated with any other tissue.

Dendrogram and PCA analyses (Figs. 7A and 7B) are consistent with correlation matrix, revealing the existence of the following functional groups: a) reproductive organs (stamen and carpel); b) leaves; both less correlated to the other following groups: c) green and maturing berries; d) rachis; e) inflorescences and opened flowers; f) buds and g) other green tissues. These analyses suggest that miRNAs reflect the functional specificity of different organs, as developmental stages of the same organ, and organ with a similar function are mainly grouped together, with few exceptions: well developed tendril that is closed to rachis, and not to other green tissues, the rachis at ripening stage and the woody bud that are less correlated to other rachis and buds samples, respectively.

### Expression of miRNAs in berries and inflorescences

**Fig. 8:**
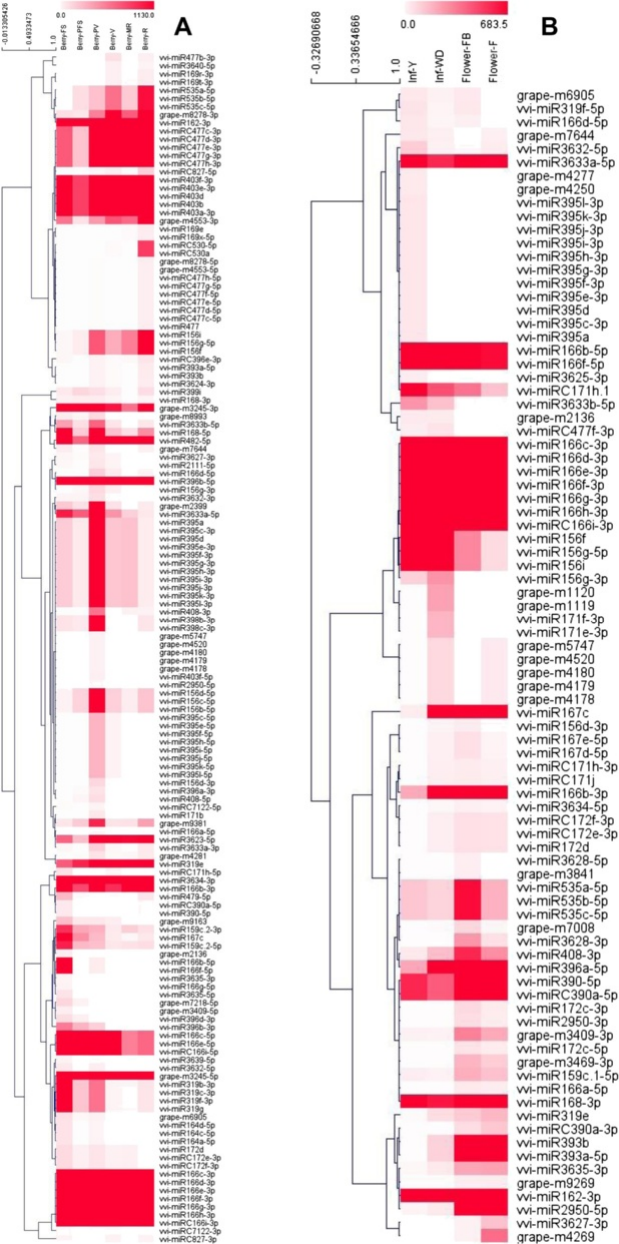
Heat map and hierarchical cluster analysis (HCL) of miRNAs with fold change greater than five. **A**: berries, **B**: flower tissues. The HCL tree was generated with the average linkage clustering method. Dark grey (red for online) and white represent high and low expression, respectively

**Fig. 9:**
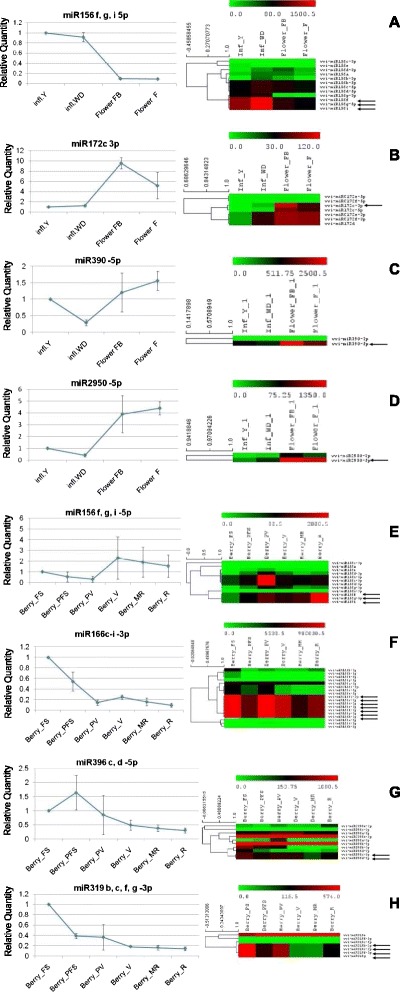
Real-Time PCR validation of deep sequencing data. Bars: standard error of the averages of each replicate. Heat maps on the right of each Real-Time graph refer to deep sequencing data. All the members of a given family are shown in heat maps, comprising 5p and 3p sequences. miRNAs tested by Real-Time PCR are indicated by an arrow. Panels A-D: miRNAs tested in the inflorescences/flowers samples; Panels E-H: miRNAs tested in the berries samples

Considering the wide spectrum of sampling in our atlas, we focused on the spatio-temporal modulation of miRNA across inflorescences and berries development. We compared the abundance level of each miRNA (average of all replicates) among the four stages of inflorescences and, separately, among the six stages of developing berries. We investigated which miRNAs showed a fold change of at least five in at least one of all possible comparisons (Fig. 8). Additionally, we performed Real Time PCR (RT-qPCR) for eight of these miRNAs to confirm their abundance patterns (Fig. 9).

In inflorescences, the well-known regulatory circuit involving miR156 and miR172 is confirmed by both sequencing and RT-qPCR data (Figs. 9A and 9B). As observed in other species [48–50], in grapevine an increase in miR156 levels corresponds to a low expression of miR172 and viceversa. From our data, miR156 family members target nine different *SPL* genes, six of which are negatively correlated to vvi-miR156 when considering their abundances (see Additional file 10).

Other two interesting miRNAs are vvi-miR390 and vvi-miR393, both involved in auxin signalling regulation, are up-regulated during inflorescence and flower development (Figs. 8B and 9C). miR390 regulates the Auxin Response Factor (ARF) through *Trans-Acting siRNA locus 3* (*TAS3*), as experimentally validated in [28], while miR393 targets the auxin receptor TIR and an F-box protein [28] highlighting the importance of auxin regulation during flower development. Other conserved miRNAs, such as miR171 and miR395, show an interesting pattern of abundance in inflorescences (Fig. 8B), although their involvement in flower development is not clear. Our data show that miR171h decreases during inflorescence development, as already shown in *Prunus* [51]: in both *Prunus* and *Vitis* miR171 targets a GRAS family transcription factor, and from our data vvi-miR171b is negatively correlated to its predicted target VIT_202s0154g00400 (Additional file 10). miR395 family members are expressed in the young inflorescences (Infl_Y – 68.5 TP5M) and are then shut down in subsequent developmental stages. It is worth noting that while in open blooming flowers miR395 is below the set threshold of expression, its abundance is very strong in stamen (369.5 TP5M) and especially in carpel (658.5 TP5M), sampled from the same open flowers (Fig. 6A) This miRNA is also present in the berry at the fruit-set stage, peaking at pre-veraison (1571 TP5M), and decreasing from *veraison* through maturation, as already observed in our previous analyses [25] that suggests a role for this miRNA, regulating sulphate metabolism, at fruit setting and veraison.

Among the newly discovered miRNAs, showing a minimum fold change of five during flower development, we wish to highlight the role of grape-m6905, discussed later, and grape-m9269. This miRNA is below the set threshold of expression in the inflorescences (Infl-Y; Infl-WD) and is expressed at higher levels (around 50 TP5M) in flowers at the beginning of flowering and in open flowers (Flower_FB and Flower_F); it targets genes annotated as encoding aphosphatidylinositol-4-phosphate 5-kinase, known to mediate pollen tube growth in *Arabidopsis* and *Nicotiana tabacum* [52, 53].

Considering the six developmental stages of the berry and the relative heat map (Fig. 8A) we found some conserved miRNAs that reveal an interesting pattern of relative abundance. First of all, as observed in inflorescences, miR156 and miR172 are inversely correlated, and seem to be actively involved in fruit maturation: vvi-miR156 shows a gradual increase during ripening (as confirmed by RT-qPCR Fig. 9E), with a corresponding decrease of vvi-miR172. Furthermore, vvi-miR396, which targets *Growth Regulating Factor* genes (*GRF*) appears to be involved in berry development. Even though some discrepancies are visible between RT-qPCR and deep sequencing (Fig. 9G), both analyses indicate a clear reduction in abundance of this miRNA after *veraison*, when the berries enter the second growth phase, doubling their volume.

Vvi-miR166c/d/e/f/g/h/i-3p abundance profile is noteworthy: this molecule is the most abundant in our sequenced libraries. It shows a decrease after fruit set (Berry_FS), and peaks at *veraison* (Berry_V). Real Time PCR (Fig. 9F) does not show a strong increase at *veraison*, nor a peak at pre-*veraison* stage, raising some doubts on the huge abundance shown by sequencing (one third of the total sequences). Among newly discovered miRNAs, grape-m6905 is interesting in the context of both flower and berry development. It is switched off only in open flowers (Flower_F) and it accumulates (69.5 TP5M) in carpels, and, later in development, shows a peak (138.5 TP5M) in berries at the fruit-set (Berry_FS). It is then nearly switched off (below 25 TP5M) after veraison. This miRNA targets genes coding for enzymes (bifunctional dihydroflavonol 4-reductase flavanone 4-reductase-like DFR-like and cinnamoylreductase-like) involved in secondary metabolite biosynthesis, such as flavonoids and anthocyanin. The *DFR* gene transcript (VIT_213s0064g00290) is negatively correlated to its miRNA grape-m6905 (see Additional file 10), considering their relative abundances, thus reinforcing our *in silico* target prediction.

## Discussion

To comprehensively define and describe the genetic programming of grapevine biology and development, it is important to extensively study genetic and epigenetic regulations driven by miRNAs, the most representative class of small non-coding RNAs. With the grapevine genome sequenced [1] and the extensive advances in next generation sequencing technologies, numerous miRNAs have been predicted in grapevine [24–31]. However, all these studies are based on few grapevine samples, mainly berries, inflorescences, leaves or tendrils at one/two developmental stages; therefore an expression atlas, covering a much wider spectrum of plant structures during grapevine life cycle, is required to provide a detailed overview of miRNA regulation and a solid base for functional studies.

### miRNA populations in grapevine

The sequencing of 70 small RNA libraries, constructed from samples of Corvina cv. and the clone PN40024, produced a total of 1,219,741,533 and 39,268,213 reads respectively. Taking into consideration the enormous inventory of small RNAs in plants and their huge complexity, the identification of a relatively small portion of miRNAs from this vast stock posed a challenge [34]. By combining high-throughput data with a well-established and stringent bioinformatics pipeline and the *in silico* prediction of miRNA targets, we studied 110 conserved and 175 novel vvi-miRNA.

The conserved vvi-miRNAs identified represent 41 families out of the 48 annotated in grapevine. The absence, in our data, of 7 annotated miRNA families is ascribed to their low abundance in the analyzed samples (*i.e.* for miR828; miR3626; miR3631; miR3636 and miR3638), or to their nature of siRNA-like miRNAs, since most of them are not annotated with high confidence in miRBase (as explained in Kozomara and Griffiths-Jones, [54]). However we cannot rule out the possibility of differences between the Corvina cultivar and the reference genome sequence derived from the clone PN40024. For example, miR3630 is present in our libraries, but was discarded by the pipeline because of a sub-optimal pairing between the miRNA and its complementary sequence. miR845 was not found in any of our libraries, confirming previous papers [25] and miRBase findings that report a very faint expression of vvi-miR845a and vvi-miR845b.

Of the 175 novel grapevine miRNA candidates unravelled and described in our analysis, 45 showed similarity with other plant miRNAs deposited in miRBase. Interestingly, two of those were generated in antisense to two members of the vvi-miRNA172 family: vvi-miRC172e and vvi-miRC172f were reverse complementary (RC) to the known vvi-miR172b and vvi-miR172a, respectively. Shao et al. [55] reported these molecules in *Vitis* as members of a novel category of miRNAs, named RC-miRNAs. They have a small regulatory activity by cleaving target mRNAs, and might possess siRNA-like activity by mediating DNA methylation [56]. Very few papers have reported this class of miRNAs, first identified in animals [57–59]. Comparing novel miRNAs identified in the present paper (either completely novel or similar to other plant miRNAs) with those reported from the hybrids of *V. vinifera* and *V. labrusca* [29, 30] and from other *V. vinifera* cultivars [31] we found only 31 shared sequences. Similarly, comparing our newly identified Corvina and PN40024 miRNAs, only two miRNAs are in common. These findings strongly suggest that, apart from a group of well-defined miRNAs, each *Vitis* variety and species has its own specific set of miRNAs, or exhibit a different spatio-temporal pattern expression because of a different genetic background or an effect of genome plasticity, as already suggested by Alabi and colleagues [31].

Moreover, our data reinforce recent findings that the population of miRNAs is highly heterogeneous across species and varieties, since the processing of miRNA precursors is not always precise and equal under every condition. We found different sequence variants (isomiR) for each miRNA and we also showed that in some loci, the sequence on the opposite arm of the annotated miRNA (usually known as miRNA*) had a higher read number than their respective annotated sequence. Although miRNAs* was conventionally been thought to be degraded and non-functional [7, 60, 61], it is now clear that they accumulate in the cells, and are able to down-regulate mRNA targets [40–42, 62]. It was also shown that the protein complex guiding target inhibition (named RISC) may be loaded with one or the other sequence of the miRNA duplex [63, 64].

We found two conserved loci (vvi-miR159c, vvi-miR169w) and one *Vitis*-specific locus (vvi-miRNA3639) producing additional small RNA duplexes besides the annotated ones (Additional file 12). All the three alternative duplexes identified show a two-nucleotide 3’-end overhang, typical of RNase III products and the vvi-miR159c and vvi-miR169w are arranged in phase [40]. The observation that multiple unique small-RNA reads could be generated from the same miRNA precursor has already been investigated by other authors [34, 55, 65, 66]. Much evidence suggests that these molecules, different from the canonical mature miRNAs, widely exist in plants and some are actually authentic miRNAs, with predicted targets.

### Fluctuations in miRNA abundance define organ identity

We established a miRNA expression atlas in the grapevine cultivar Corvina, including 25 different samples, and 354 miRNA sequences. This atlas, because of its dimension, represents a crucial step in the development of future targeted functional studies.

We analyzed the distribution of known and novel miRNAs and their relative abundance, in several plant structures such as berries, inflorescences, tendrils, buds, carpels, stamens, leaf, stem and rachis.

Considering the normalized miRNA abundances, we observed that known and conserved miRNAs are, on average, more abundant (average expression 15,000 TP5M) than novel grapevine-specific miRNAs (average expression around 150 TP5M), confirming that recently evolved molecules are less expressed and generally more tissue-specific (Fig. 6) [30, 34, 67–69]. Moreover, the analysis of the top-most expressed miRNAs suggests that miRNAs expression trend is characterized by the presence of few abundant miRNAs (more than 1000 TP5M) (Additional file 11), while the majority of the miRNAs accumulates at a lower level in the different samples.

We then compared the distribution of the miRNAs across our samples: the set of miRNAs expressed appears highly variable: only 38 miRNAs are expressed in all our samples and few miRNAs are highly organ specific miRNAs (for example only three miRNAs are specific to the stem), with the exception of stamen, where 22 miRNAs appear to be specific. Wang and collaborators [30], similarly, identified in grapevine a small number of tissue-specific miRNAs, but a large number of miRNAs expressed in all their six samples. This result is not in contrast with our findings, if we consider the higher number of samples used in the present work. Indeed, if we consider only the various developmental stages of a given organ, we identify a high proportion (more than 40%) of expressed miRNAs as shared among samples. This suggests that these miRNAs are needed for organ-identity and functionality, and that less miRNAs are involved in a specific developmental phase. Rachis has a different behaviour, since only 30% of miRNAs are shared among all the five stages considered, while many molecules are stage-specific (i.e. 20 miRNAs expressed only in Rachis at post fruit set, when compared to all other rachis samples) or shared by three or four stages (Fig. 5). This would seem to indicate that different developing stages of rachis are characterized by a heterogeneous miRNA population that drives a well-defined gene regulatory network.

Hierarchical clustering analyses, together with PCA and correlation matrix, sustain the evidences that miRNAs influence organ identity, highlighting the similarities among grapevine samples derived from the same plant structure. The correlation matrix (Additional file 12) together with the dendrogram (Fig. 7A) and the PCA (Fig. 7B) show that stamen and carpel are distant from all other samples, and moderately related to each other. Data from all our analyses suggest the presence of two major clusters: one including all the berries and their rachis, and the second comprising inflorescences, buds and the green vegetative tissues. Leaf is always distant from the rest of the considered samples, closer to berries than to other green tissues.

These analyses suggest that fluctuations in the levels of the miRNAs in different samples, without high organ specificity, may have a crucial functional role, in defining organ identity and developmental programming.

This observation differs from what described in Fasoli et al. [4], who performed a transcriptomics analysis on the same samples we used, showing that grapevine tissues undergo a transcriptional reprogramming during maturation. In their work, grapevine tissues were mainly clustered based on the maturity stage rather than organ identity, with green berries more similar to young leaves and tendrils than to ripe berries. This means that miRNAs may act differently from other protein coding genes, establishing a regulatory layer that strictly influences organ identity.

### miRNA involved in berry and inflorescence development

The regulation of grapevine flowering is still poorly characterized [70], and our results highlight some key regulatory steps driven by miRNAs. Similarly, our studies on berry development enrich present knowledge on miRNA involvement in fruit (berry) maturation, only partially disclosed in tomato [71, 72].

In inflorescence, a well recognized role is played by miR156-miR172 regulation: miR156 decreases during inflorescence development while miR172 increases (Figs. 9A and 9B). This is because *SPL*, the target of miR156, is an elicitor of miR172 promoter region which, in turn, targets APETALA2. This regulatory unit is fundamental for vegetative to reproductive changes and for the subsequent flowering onset and flower organ identity [49, 73, 74]. Our data also suggest a role of miRNAs in the hormonal regulation of floral development: in particular for auxin and ethylene. Auxin-regulating miRNAs are clearly modulated in our data: miR390, targeting ARF via ta-siRNA production, and miR393, targeting auxin receptors are both up-regulated (Fig. 8B) at late inflorescence development (Flower_FB and Flower_F), with open flowers, thus suggesting a role for this hormone in the first stages of inflorescence development. A previous work in the hybrid Summer Black (*V. vinifera* x *V. labrusca*) showed a down-regulation of miR390 and miR393 in open flowers, compared to young inflorescences [27]. This difference could be an indication of a diverse mechanism governing auxin signalling during inflorescence development in the two *Vitis* species. *V. labrusca* and *V. vinifera* were already shown to have a different auxin accumulation pattern, in berry development as well [75]. Among the stamen specific miRNAs, two miRNAs (grape-m5380 and grape-m7774) were predicted to target five ACC oxidases, key enzymes in ethylene biosynthesis, suggesting a role for these miRNAs in hormone regulation during anther and pollen development. In grapevine, ethylene production peaks at anthesis before berry fruit set (phenological stage EL 23 – EL 26), inducing fruitlet abscission [76]. Subsequently, its level rises until pre-veraison stage, and declines at maturity [77]. Ethylene treatment of berries at pre-veraison induces an over expression of numerous grapevine-specific miRNAs [30]. Although these miRNAs were not detected in our libraries, this evidence supports the existence of ethylene responsive miRNAs. The role of ethylene in anther development has not been extensively studied [78, 79]. Recently it was shown that ACC oxidase gene is more expressed in female flowers of wild *Vitis* than in hermaphrodite or male flowers, corroborating our hypothesis of an active role of ethylene in female structure development [80]. Interestingly, grape-m7774 maps in a chromosomal region where four out of its targets also map. A total of 19 genes coding for ACC oxidase map in this region (Fig. 4) suggesting that grape-m7774 could have arisen from a tandem duplication of a target gene, followed by genetic drift, leaving only a short region of complementarity for the gene target, a mechanism suggested for miRNA evolution [81]. Among the newly discovered miRNAs, we found that grape-m9269 might be potentially involved in pollen growth, as it was predicted to target a gene coding for a Phosphatidylinositol-4-phosphate 5-kinase [52, 53], responsible for the synthesis of the minor membrane phospholipid phosphatidyl inositol - 4,5-bisphosphate. This enzyme is involved in the modification of the plasma membrane during pollen tube elongation. In agreement with this putative role, grape-m9269 is expressed in open flowers and flowers at the beginning of blooming (Flower_F and Flower_FB), but is absent in stamens and carpels. This observation suggests a role in confining the target gene expression in the organs where the pollen grains grow and develop the pollen tubes.

Considering berry development, our atlas provides interesting hints on the maturation of a non-climacteric fruit, in which the regulatory role played by miRNAs has been scarcely investigated. Some of the most conserved miRNAs reveal intriguing profiles: among others, miR156 and miR164 seem to be involved in berry maturation, in an ethylene independent manner. *SPL* genes, targeted by vvi-miR156, decrease during berry ripening in grapevine [82] and, accordingly, we showed that vvi-miR156 is more expressed in the last stage of berry maturation. In tomato, an epimutation in an *SPL* gene is responsible for *Cnr* mutants, which result in colorless fruits with loss of cell-to-cell adhesion [82]. *SPL* genes are upstream of ethylene synthesis in tomato, activating maturation through alternative programs [83]. Furthermore, in *Arabidopsis*, an over expression of miR156 determines a reduced *SPL* genes activity and, by a subsequent regulation of several genes, lead to an accumulation of anthocyanins [84], likely connected to *DFR* gene activation [85], a flavonoid biosynthetic gene. These data reinforce the idea vvi-miR156 may be responsible for inducing maturation programs in grapevine berries, in an ethylene independent manner, through *SPL* genes regulatory networks and anthocyanins accumulation, secondary metabolites accumulating in mature berries of red grapevine varieties.

vvi-miR164 is also modulated during berry development (125.5 TP5M in berry at fruit set, then declines below the threshold of expression), confirming previous observations [24, 86] that show its gradual decrease during ripening. vvi-miR164 target gene is *VvNAC33* [28, 86], reported to increase during ripening [87]. Interestingly, its tomato homolog *LeNOR* exerts a direct role in berry ripening progression, being upstream of ethylene regulatory signals such as *CNR* [88, 89]

Considering the particular interest of secondary metabolites during berry maturation, and the large variety of phenylpropanoid compounds present in grapevine, two miRNAs caught our attention because their abundance patter may indicate a connection of miRNAs function to secondary metabolism. Vvi-miR395, whose abundance decreases from pre-*veraison* to maturity, as already reported in [25], down-regulates a class 2-sulphate transporter and it has been usually involved in sulphur uptake and assimilation, or in nutrient starvation response [90–92]. Class 3 sulphate transporters were shown to be down-regulated during late berry maturation, reinforcing the role of sulphate metabolism in berry ripening [93]. The link between sulphur depletion and flavonoid and stilbene biosynthetic pathways was studied in grapevine by Tavares et al. [94] in both photosynthetic and non photosynthetic tissues. In this respect, it is plausible that miR395, by regulating sulphate transporter, may be implicated in the activation of phenylpropanoid pathways.

The grapevine-specific miRNA grape-m6905, whose expression peaks in carpels and in berries at fruit set, being down-regulated at the onset of ripening, was predicted, instead, to target seven genes (Additional file 8) whose amino acid sequences share an identity ≥70% and are annotated as bifunctional dihydroflavonol 4-reductase flavanone 4-reductase-like (DFR-like) and cinnamoylCoA reductase-like proteins (CCR). *DFR* is a structural gene of the flavonoid biosynthesis pathway, specifically involved in anthocyanin and proanthocyanins biosynthesis [95], while CCR (GO:0006694; GO:0008207) might be involved in steroid and phenylpropanoid biosynthesis. Brassinosteroids (BR) are important regulators of ripening onset in grapevine [75], and induce the expression of the anthocyanin biosynthesis genes, such as *DFR*, when exogenously applied to berry skins [96]. BR endogenous level during berry ripening in grapevine accumulates in flower and early berry development (2 weeks after flowering, coincident with our berry at fruit set), and at late maturation stage [75]. Accordingly, grapevine genes involved in BR synthesis are up-regulated during berry development [75]. A reduction of grape- m6905 abundance right at the onset of ripening could coincide with an activation of BR synthesis and consecutively with berry maturation programs.

Our results on miRNA involvement in berry and inflorescence development highlight the importance of studying species-specific and conserved miRNAs in the context of specific metabolic pathways, such as berry maturation and secondary metabolism, and in specific organs, such as stamen and carpel, to uncover new roles for miRNAs, not limited to the conventional involvement in plant growth and development regulation.

## Conclusions

In the present study, we applied next-generation sequencing technology to study miRNA involvement in grapevine development. Our small RNA libraries, covering a large set of plant structures at different developing stages, allowed us to identify not only110 known miRNAs but also 175 novel miRNA candidates, considerably extending the number of miRNAs described in grapevine.

Our work assesses the spatio-temporal distribution of miRNAs, defining the role of miRNAs in sculpturing organ identity and fine tuning developmental programs. Combining target prediction and expression profile, we shed light on the functional role of some miRNAs adding insight on inflorescence and fruit development, reinforcing, among others, the role of the miR156/miR172 regulatory circuit in both inflorescence and berry development and suggesting a role of miRNAs in regulating hormonal-driven fruit maturation and inflorescence development. For example, stamen specific miRNAs are putatively involved in regulating ethylene production, stimulating stamen development.

Our atlas aims at becoming the reference for the future development of targeted functional studies, a first indispensable step towards the definition of miRNA involvement in grapevine development.

## Methods

### Plant material

All samples of *Vitis vinifera* L. cv Corvina were collected from a 7-year-old vineyard in Montorio, during 2008/2009 growing seasons.

Thirty five grapevine samples (listed in Table 1) from 13 different plant structures at different developmental stages were collected, sampling two biological replicates, except for seeds. All samples used in this study are those used for the transcriptomics atlas [4], from which RNA was isolated. Developmental stages were recorded according to the modified Eichorn and Lorenz system [97, 98] (for a detailed description of each sample see Additional file 13 and Fasoli et al. [4].

Leaves and roots at juvenile stage were collected from *Vitis vinifera* clone PN40024 grown *in vitro* on half strength Murashige and Skoog [99] MS medium with 15 g/l sucrose, at 25 °C with a 16 h light cycle.

### RNA extraction and library preparation

The small RNA fraction was isolated by two different methods, depending on tissue types. Specifically, small RNAs from leaf, stem, flower, tendril, anther, and carpel, were extracted with miR Premier™ MicroRNA Isolation Kit (Sigma-Aldrich®), following the manufacturer’s protocol. Small RNAs from bud, berry, rachis were first extracted using Plant RNA Isolation Reagent (PRIR – Life Technologies™) and followed by the miR Premier™ MicroRNA Isolation Kit (Sigma-Aldrich®). A total of 100 mg of ground tissue was used as input material. Small RNA quality and quantity were evaluated with a Nanodrop 1000 spectrometer (Thermoscientific). RNA integrity was determined on an Agilent 2100 Bioanalyzer using a small RNA chip (Agilent Technologies) according to the manufacturer’s instructions.

Small RNA libraries were constructed as previously described [25] following the TruSeq Small RNA Sample Preparation guide (Illumina Inc.). Seventy small RNA libraries were constructed starting from 50 ng of small RNA. Bar-coded libraries were amplified with 15 cycles PCR, prior to 6% PAGE gel purification. Corvina derived libraries were grouped in pools containing 6/7 libraries each and sequenced on an Illumina Hiseq2000 (Illumina Inc.). Small RNA libraries from PN40024 were constructed following the Small RNA Sample Prep Kit (Illumina Inc.), starting from 2 μg of small RNAs, and sequenced independently on a GAIIx Illumina Sequencer (Illumina Inc.). Library sequencing was performed by IGA Technology Services (Udine, Italy).

The sequencing data was submitted to GEO – NCBI under the accession number GSE59802.

### Computational analysis of sequencing data

Raw reads were trimmed by removing adaptor sequences and selecting reads between 18 and 34 nt long, which were then mapped to the reference *Vitis vinifera* L. genomic sequence (PN40024 [1] using Bowtie [100]. Perfect matches were retained, excluding those matching rRNAs, tRNAs, snRNAs and snoRNAs.

The abundance of each sequence was normalized to overcome sequencing bias. Data were normalized using the linear count scaling method [101] calculated as:

$$ \mathrm{T}\mathrm{P}\mathrm{M}\kern0.5em \mathrm{abundance}=\left[\frac{\boldsymbol{raw}\kern0.5em \boldsymbol{value}}{\left(\mathrm{total}\kern0.5em \mathrm{genome}\kern0.5em \mathrm{matches}\hbox{-} \mathrm{t}\backslash \mathrm{r}\backslash \mathrm{s}\mathrm{n}\backslash \mathrm{s}\mathrm{n}\mathrm{o}\backslash \mathrm{chloroplast}\backslash \mathrm{mitochondria}\kern0.5em \mathrm{matches}\right)}\right]\times \mathrm{n}\_\mathrm{base} $$

Where n_base is 5,000,000 and TP5M is Transcript Per 5 Million.

### Identification of annotated and novel vvi-miRNA

Annotated vvi-miRNAs (known vvi-miRNAs) were characterized in the 68 small RNA Corvina derived libraries, whereas novel vvi-miRNAs were identified using the whole set of Corvina and PN40024 libraries, by independently applying the miRNA identification pipeline. The analysis was performed as described by Jeong and colleagues [33] and Zhai and co-authors [34].

Finally, annotated vvi-miRNAs identified by the pipeline were passed through a careful manual inspection, evaluating their precursors and retrieving the most abundant isoform within the reads mapping on each precursor.

To discover novel vvi-miRNA candidates, we retained only those for which the most abundant sequence was 20, 21 or 22 nt long. All novel vvi-miRNA candidates were compared against miRBase version 20 to identify high-similarity homologs. Novel candidates were manually evaluated for precursor secondary structures, using the plant version of the UEA sRNA hairpin folding and annotation tool, with default settings [43]. For both annotated and novel miRNAs, complementary 3p/5p were retrieved when identified by the pipeline or when it was possible to recognize them according to the miRBase annotation.

### miRNA accumulation analysis

We defined a miRNA as “expressed” only when the values of both biological replicates were greater than or equal to the threshold set at 10TP5M. Any miRNA below this threshold was considered as “not expressed”. We defined a miRNA as “organ-specific” when it was present only in a given organ, in at least one of the developmental stages of that given organ and its abundance exceeded the threshold of 10TP5M. The final miRNA expression counts were obtained by calculating the average of the two normalized biological replicates.

### Target prediction and analysis

miRNA targets were identified using TargetFinder, release 1.6 (www.carringtonlab.org [44]). miRNAs were matched against the grape mRNA and long non-coding RNA sequences version 2.1 (V2.1_mrna.fa downloaded from http://genomes.cribi.unipd.it/grape/). Analyses were conducted setting a score cut off of four but results show targets with a score ≤ 3, to reduce false positives. psRNATarget [47] was used, with a score cut off 4.0, UPE:50.9 to check previous predictions.

We computed Pearson correlation values among all considered miRNA:target pairs, using the normalization procedure indicated in the following paragraph of the "Methods" section. To obtain a proper comparison, we also log10 transformed the values of the available expression data coming from [4], considering all the 24 common samples shared between the two datasets. A p-value cutoff was set to 0.05 and we selected all those pairs showing a negative correlation.

### Correlation and PCA analyses

Normalized reads (NR) of all miRNAs detected and predicted in this study were normalized for correlation analysis using the log_10_ (1 + NR) of expression value (normalized reads) profiles. The log_10_ (1 + NR) normalization was chosen first because of the very large range of expression values which produced a log-unimodal distribution and significant biases in the un-normalized correlation analysis. Second, we added a unity to the expression value due to the presence of zero entries. With this last choice, a value of zero still corresponds to zero of the log_10_ (1 + NR) function, thus making consistent the comparisons between profiles. As a distance metric we used the usual “one minus Pearson correlation” to compare the values of miRNA in 68 samples by taking the average of the log-normalized NR reads. The log-normalized values were also used to evaluate the reproducibility of our replicates (Additional file 14). A Principal Component Analysis (PCA) has been performed on the same dataset.

### Real-Time PCR on mature miRNAs

Total RNA was extracted from two biological replicates of inflorescence and berries samples, using the Spectrum™ Plant Total RNA Kit (Sigma-Aldrich®), and subsequently DNase treated using DNA-Free™ Kit (Life Technologies™). RNA quality was checked using a spectrophotometer (DU640 Beckman) and its integrity was determined on an Agilent 2100 Bioanalyzer using a RNA 6000 Nano Kit (Agilent Technologies). Treated total RNA (200 ng) was subjected to pulsed reverse transcription following Varkonyi-Gasic [102], with minor modifications and using Superscript® III (Life Technologies™) and stem-loop specific primers. Absence of genomic DNA was verified by performing a control PCR on the Actin gene (*VIT_204s0044g00580*), whose primers span an intron, checking amplicon length.

RT-qPCR was performed using the SYBR Green PCR Master Mix (Life Technologies™), with reaction volumes of 25 μl, three technical replicates and running each plate on the 7300 Real-Time PCR System (Life Technologies™). Eight different miRNAs were tested using a poly-ubiquitin transcript (VIT_219s0177g00040, [103] and a snRNA U6 transcript (u6wg_chr6_15577690_15577792_+) as internal standards. Primers are listed in Additional file 15. The relative quantification of each miRNA tested was calculated from Ct value, using the 2-ΔΔCt method.

### Availability of supporting data

The sequence data supporting the results of this article are available in the GEO – NCBI data libraries under accession number GSE59802. Additionally, a publicly available database was set up at the following website https://mpss.udel.edu/dbs/index.php?SITE=grape_sRNA_atlas, hosting our small RNA libraries.

## Additional files

Additional file 1:
**Small RNA Libraries Sequencing Statistics.** A pool of 8 barcoded libraries were sequenced in a single channel, except for libraries constructed with tissues of PN40024 clone which were sequenced in a separate and individual channel. The description of library codes is indicated in Table 1. Additional file 2:
**Known vvi-miRNAs identified in Corvina-derived libraries.** List of reliable vvi-miRNAs from known miRNA precursors identified in Corvina-derived libraries. Additional file 3:
**Stem-loop structure of two miRNA loci generating more than one duplex.** Dot-bracket notation representing RNA stem-loop structure of some examples of miRNA loci, generating more than one distinct duplex from the same precursor. For each precursor MIR locus and mapping position (chromosome, strand, coordinates) are presented. Below the dot-bracket notation, distinct miRNA duplexes are aligned and the sum of the abundance (TP5M) of the reads is shown in parenthesis. The most abundant duplex is highlighted. Additional file 4:
**Novel vvi-miRNAs identified in Corvina-derived libraries.** List of novel *Vitis vinifera* miRNAs identified in small RNA libraries of Corvina-derived tissues. A temporary name has been given to each miRNA sequence either using a sequential numbering associated with the abbreviation mirC (miRNA Candidate) for those similar to other known plant miRNAs or using “grape-m” with a random numbering for those completely new candidates. Additional file 5:
**Novel vvi-miRNAs identified in PN40024-derived libraries.** List of novel *Vitis vinifera* miRNAs identified in small RNA libraries from tissues of the PN40024 clone. A temporary name has been given to each miRNA sequence either using a sequential numbering associated with the abbreviation miRC (miRNA Candidate) for those similar to other known plant miRNAs or using “grape-m” with a random numbering for those completely new candidates. Additional file 6:
**Secondary structure of novel miRNAs identified in Corvina-derived libraries.** List of all RNA secondary structures of novel miRNA precursors, predicted using the RNA folding tool of the UEA sRNA toolkit – Plant version [43]. The sequence of the mature miRNA is highlighted in red and the complementary sequence (miRNA*) is highlighted in pink when present. Additional file 7:
**Secondary structure of novel miRNAs identified in PN40024-derived libraries.** List of all RNA secondary structures of novel miRNA precursors, predicted using the RNA folding tool of the UEA sRNA toolkit – Plant version [43]. The sequence of the mature miRNA is highlighted in red and the complementary sequence (miRNA*) is highlighted in pink when present. Additional file 8:
**Putative targets of known and novel vvi-miRNAs**. List of putative targets as identified by TARGET FINDER. Only targets with score ≥ 3 are listed. GRAPE_IGGP_v1 12X assembly of *Vitis vinifera* genome and the v2 annotation were used. Additional file 9:
**Number of putative miRNA targets.** Number of putative miRNA target within the (A) PN40024 and (B) Corvina data sets, identified using the TargetFinder (release 1.6). Novel and known refer to the different classes of miRNAs. Additional file 10:
**miRNA - target correlations.** From a list of 933 predicted miRNA - target pairs we could find 815 pairs for which the target gene expression values are available from a public dataset [4]. The table reports the Pearson correlations of the pairs for which the P value is less than 0.05. The P value is computed for testing the hypothesis of no correlation against the alternative that there is a non-zero correlation. The annotations reported are the same as in Additional file 8. Additional file 11:
**Top 20 expressed miRNAs per tissue/developmental stage.** Top 20 expressed miRNAs in each tissue/developmental stage studied. Reads refers to the average of TP5M of both replicates. Additional file 12:
**Correlation matrix of the 25 grapevine samples used to define the miRNA atlas.** One minus Pearson correlation was used as a metric distance. Main clusters identified are highlighted with a red box. Additional file 13:
**Additional plant material details.**
Additional file 14:
**Pearson correlation between replicates.** Values of Pearson correlation between replicates using log2 transformed data. Additional file 15:
**Primers used for Real Time PCR (RT-qPCR).**

## Abbreviations

ACC ox: Aminocyclopropane-1-carboxylate oxidase homolog
AP2: APETALA 2 transcription factor
ARF: Auxin Response Factor
BR: Brassinosteroids
CCR: Cinnamoyl CoA reductase-like proteins
CNR: Colorless non-ripening mutation
DCL: Dicer-Like protein
DFR: Bifunctional dihydroflavonol 4-reductase flavanone 4-reductase
grape-mXXXX: Novel grapevine microRNA candidate
GRAS: Transcription factor of the family Gibberellin-Insensitive, Repressor of ga1-3, Scarecrow
GRF: Growth Regulating Factor
isomiR: Sequences that have variations with respect to the reference miRNA sequence
LeNOR: *Lycopersicon esculentum* non-ripening
miRNA*: microRNA star, the complementary strands of functional mature miRNA
MS: Murashige and Skoog medium
MYB: Transcription factor containing a Myb DNA binding domain
NR: Normalized reads
PCA: Principal Component analysis
PRIR: Plant RNA Isolation Reagent
RC-miRNAs: Reverse complementary microRNAs
RISC: RNA-induced silencing complex
RT-qPCR: Quantitative real-time polymerase chain reaction
snoRNA: small nucleolar RNA
snRNA: small nuclear RNA
SPL: Squamosa Promoter binding Like protein
TAS3: Trans-Acting siRNA locus 3
TCP: Transcription factor of the family Teosinte branched 1 – Cycloidea- PCF
TMV: Tobacco Mosaic Virus
TP5M: Tags per 5 million
vvi-miRC: *Vitis vinifera* miRNA candidate
vvi-miRNAs: *Vitis vinifera* microRNAs
VvNAC: *Vitis vinifera* NAM ATAF1/2 and CUC transcription factor
