# Supplementary material for: miRVine: a microRNA expression atlas of grapevine based on small RNA sequencing
Source: BMC Genomics. 2015 May 16;16(1):393. doi: 10.1186/s12864-015-1610-5 (PMC4434875; doi:10.1186/s12864-015-1610-5)
Supplement: Additional file 1: — Small RNA Libraries Sequencing Statistics. A pool of 8 barcoded libraries were sequenced in a single channel, except for libraries constructed with tissues of PN40024 clone which were sequenced in a separate and individual channel. The description of library codes is indicated in Table 1. [file 12864_2015_1610_MOESM1_ESM.pdf]

Library[a]	Total raw reads	Total trimmed and chopped reads [b]	Genome matched reads[c]	Distinct genome matched reads[d]	Norm. Base Value
Leaf_Y_1	3,654,530	2,703,403	1,847,507	45,605	5M
Leaf_Y_2	14,047,558	10,259,599	7,076,384	205,228	5M
Leaf_M_1	23,919,119	16,781,407	10,496,403	332,423	5M
Leaf_M_2	11,229,400	7,571,706	5,080,020	114,891	5M
Leaf_S_1	61,913,309	3,561,231	961,790	44,115	5M
Leaf_S_2	35,661,460	2,325,212	836,624	44,711	5M
Bud_AB_1	24,910,567	16,455,864	11,887,356	840,267	5M
Bud_AB_2	38,002,867	18,178,396	11,866,561	593,414	5M
Bud_W_1	90,370,886	63,309,882	29,818,837	1,219,258	5M
Bud_W_2	25,103,137	14,378,785	5,345,542	238,304	5M
Bud_L_1	5,767,134	4,155,696	2,167,221	221,768	5M
Bud_L_2	12,202,177	9,639,987	4,903,784	493,314	5M
Bud_B_1	12,191,778	6,634,330	2,816,937	411,134	5M
Bud_B_2	8,216,185	4,928,458	2,786,044	321,073	5M
Stem_G_1	14,844,088	10,841,283	5,461,486	564,999	5M
Stem_G_2	11,887,764	9,007,741	4,597,608	419,102	5M
Stem_W_1	5,332,592	1,446,311	511,273	38,498	5M
Stem_W_2	9,068,998	1,846,916	773,875	109,113	5M
Tendril_Y_1	7,813,615	5,504,235	2,715,954	285,216	5M
Tendril_Y_2	6,006,154	4,020,708	2,083,571	178,309	5M
Tendril_WD_1	36,504,545	32,098,256	14,793,660	1,437,853	5M
Tendril_WD_2	35,079,190	29,829,202	14,941,857	795,317	5M
Inf_Y_1	8,289,504	6,927,349	4,151,269	614,257	5M
Inf_Y_2	2,445,199	1,798,221	1,075,373	280,721	5M
Inf_WD_1	17,935,860	14,736,534	8,784,401	1,439,149	5M
Inf_WD_2	16,309,663	12,784,241	7,364,686	1,245,667	5M
Flower_FB_1	13,854,867	9,901,145	5,039,538	850,752	5M
Flower_FB_2	15,631,539	10,508,193	5,477,879	867,524	5M
Flower_F_1	12,177,992	7,213,821	3,638,341	496,054	5M
Flower_F_2	7,482,840	4,469,464	2,209,586	341,591	5M
Carpel_1	25,519,681	19,151,781	12,830,932	404,996	5M
Carpel_2	14,926,800	9,839,619	5,854,619	332,553	5M
Stamen_1	6,075,758	134,009	68,939	19,341	5M
Stamen_2	9,157,957	5,087,330	2,642,802	171,886	5M
Pollen_1	26,159,587	16,259,893	5,914,794	173,807	5M
Pollen_2	32,812,315	15,894,623	5,641,490	198,145	5M
Berry_FS_1	5,254,324	4,032,657	2,041,767	366,954	5M

Library[a]	Total raw reads	Total trimmed and chopped reads [b]	Genome matched reads[c]	Distinct genome matched reads[d]	Norm. Base Value
Berry_FS_2	14,124,522	12,342,702	7,005,085	1,034,486	5M
Berry_PFS_1	8,521,962	5,194,406	2,367,687	263,136	5M
Berry_PFS_2	12,540,245	5,453,367	3,204,290	136,005	5M
Berry_PV_1	4,891,497	389,291	177,342	48,498	5M
Berry_PV_2	18,907,560	14,621,426	7,125,979	402,872	5M
Berry_V_1	23,457,803	7,472,458	3,257,214	275,432	5M
Berry_V_2	28,502,576	15,589,003	8,160,504	414,250	5M
Berry_MR_1	17,067,774	5,681,954	2,712,130	185,918	5M
Berry_MR_2	4,705,105	2,332,135	1,174,580	78,802	5M
Berry_R_1	18,805,503	13,191,573	5,541,863	420,900	5M
Berry_R_2	15,100,094	10,406,519	4,484,614	299,989	5M
Berry_PHWI_1	20,626,256	13,443,373	8,015,033	183,806	5M
Berry_PHWI_2	28,943,491	20,533,068	12,821,178	189,743	5M
Berry_PHWII_1	31,558,460	19,029,491	10,657,068	299,342	5M
Berry_PHWII_2	25,282,044	15,221,931	7,204,637	330,922	5M
Berry_PHWIII_1	21,857,218	10,805,331	4,924,114	169,996	5M
Berry_PHWIII_2	13,476,822	5,278,299	2,207,471	117,501	5M
Rachis_FS_1	5,431,763	3,395,323	2,443,782	88,328	5M
Rachis_FS_2	8,679,594	6,243,727	3,342,944	475,288	5M
Rachis_PFS_1	9,966,365	7,452,776	3,518,223	301,464	5M
Rachis_PFS_2	20,646,854	16,814,495	8,111,747	703,561	5M
Rachis_V_1	35,803,893	21,536,834	10,439,751	738,904	5M
Rachis_V_2	9,263,610	5,656,371	2,272,928	139,412	5M
Rachis_MR_1	26,098,541	14,918,832	7,098,892	492,298	5M
Rachis_MR_2	9,726,086	4,838,391	2,106,212	183,749	5M
Rachis_R_1	20,213,091	8,707,116	3,918,208	153,547	5M
Rachis_R_2	8,476,449	2,863,567	1,204,647	66,783	5M
Root_1	2,338,768	687,381	280,394	43,743	5M
Root_2	2,309,685	495,593	230,058	34,156	5M
Seed_G	17,666,703	9,120,131	3,944,960	214,301	5M
Seed_M	26,990,260	15,135,973	6,100,650	145,549	5M
Summed Total (Corvina-derived libraries)	1,219,741,533	709,070,325	362,560,895	25,389,990	
Leaf_PN[e]	15,413,571	11,924,156	8,786,077	1,449,032	5M
Root_PN[e]	23,854,642	18,401,623	5,981,041	479,536	5M

Library[a]	Total raw reads	Total trimmed and chopped reads [b]	Genome matched reads[c]	Distinct genome matched reads[d]	Norm. Base Value
Summed Total (PN40024-derived libraries)	39,268,213	30,325,779	14,767,118	1,928,568	
Summed Total	1,259,009,746	739,396,104	377,328,013	27,318,558	

[a] Small RNA libraries from biological replicates are indicated as “1” and “2” after the library code
[b] total sequences from 18 to 34 nt after trimming the adaptors
[c] Total number of sequences ranging from 18 to 34 nt which perfectly matched to the Grapevine genomic sequence, excluding sequences matched to structural RNAs (t/r/sn/snoRNAs) (GRAPE_IGGP12Xv1).
[d] Number of genome-matched sequences which are uniquely found within the set, excluding sequences matched to structural RNAs (t/r/sn/snoRNAs).
[e] these libraries were prepared and sequenced using different sequencing platform and kit (Small RNA Sample Prep kit – Illumina; GAIIx Illumina Sequencer)
