# Supplementary material for: miRVine: a microRNA expression atlas of grapevine based on small RNA sequencing
Source: BMC Genomics. 2015 May 16;16(1):393. doi: 10.1186/s12864-015-1610-5 (PMC4434875; doi:10.1186/s12864-015-1610-5)
Supplement: Additional file 2: — Known vvi-miRNAs identified in Corvina-derived libraries. List of reliable vvi-miRNAs from known miRNA precursors identified in Corvina-derived libraries. [file 12864_2015_1610_MOESM2_ESM.pdf]

Family	miRNA	chr[a]	start[a]	end[a]	strand[a]	5p sequence	3p sequence	Abundance 5p[b]	Abundance 3p[b]
	vvi-miR156a	19	8708913	8709017	-	TTGACAGAAGAGAGGGAGCAC		833	
	vvi-miR156b	4	5356984	5357115	-	TGACAGAAGAGAGTGAGCAC	GCTCATTTCTCTTTCTGTCAGC	43355	2047
	vvi-miR156c	4	848189	848322	-	TGACAGAAGAGAGTGAGCAC	GCTCACTCTCTATCTGTCATC	43355	356
	vvi-miR156d	11	7623202	7623334	-	TGACAGAAGAGAGTGAGCAC	TGCTCACCTCTCTTTCTGTCAGC	43355	4244
	vvi-miR156e	11	1504195	1504301	-	TGACAGAGGAGAGTGAGCAC		302	
	vvi-miR156f	14	26463671	26463773	+	TTGACAGAAGATAGAGAGCAC		63788	
	vvi-miR156g	17	3046310	3046441	-	TTGACAGAAGATAGAGAGCAC	GCTCTCTAGACTTCTGTCATC	63788	4006
miR156	vvi-miR156i	14	19727139	19727358	-	TTGACAGAAGATAGAGAGCAC			63788
	vvi-miR159a	15	18469215	18469323	-		-TTGGAGTGAAGGGAGCTCTC		1203
	vvi-miR159c.1	17	2609190	2609409	-	GAGCTCCTTGAAGTCCAATAG	TTTGGATTGAAGGGAGCTCTA	5376	3260544
miR159	vvi-miR159c.2	17	2609190	2609409	-	AGCTGCTGACTTATGGATCCC	CTTGCATATCTCAGGAGCTGC	60183	113250
	vvi-miR160c	10	11745661	11745763	+	TGCCTGGCTCCCTGTATGCCA		251	
	vvi-miR160d	8	13017021	13017120	+	TGCCTGGCTCCCTGTATGCCA		251	
miR160	vvi-miR160e	13	5447916	5448036	+	TGCCTGGCTCCCTGTATGCCA		251	
miR162	vvi-miR162	17	4716504	4716636	+	GGATGCAGCGGTTCATCGATC	TCGATAAACCTCTGCATCCAG	328	465426
	vvi-miR164a	7	3287455	3287605	-	TGGAGAAGCAGGGCACGTGCA	CACGTGCTCCCCTTCTCCAAC	2667	451
	vvi-miR164c	8	10080445	10080636	+	TGGAGAAGCAGGGCACGTGCA	CATGTGCCCCTCTTCCCCATC	2667	1418
miR164	vvi-miR164d	14	1414550	1414697	-	TGGAGAAGCAGGGCACGTGCA	CACGTGCTCCCCTTCTCCAAC	2667	451
	vvi-miR166a	8	3302784	3302939	-	AATGAGGTTTGATCCAAGATC	TCTCGGACCAGGCTTCATTCC	5392	7852683
	vvi-miR166b	12	17937384	17937510	+	GGAATGTTGGCTGGCTCGAGG	TCGGACCAGGCTTCATTCCTC	67391	97673
	vvi-miR166c	15	16978558	16978745	-	GGAATGTTGTCTGGCTCGAGG	TCGGACCAGGCTTCATTCCCC	215923	24298680
	vvi-miR166d	16	21405202	21405388	-	GATTGTTGTCTGGCTCGAGGC	TCGGACCAGGCTTCATTCCCC	4808	24298680
	vvi-miR166e	2	2255708	2255901	+	GGAATGTTGTCTGGCTCGAGG	TCGGACCAGGCTTCATTCCCC	215923	24298680
	vvi-miR166f	7	19450000	19450127	+	GGAATGTTGGCTGGCTCGAGG	TCGGACCAGGCTTCATTCCCC	67391	24298680
	vvi-miR166g	7	453844	453971	-	GGAATGTTGTCTGGTTCGAGA	TCGGACCAGGCTTCATTCCCC	463	24298680
miR166	vvi-miR166h	5	6741174	6741301	-	GGAACGCTGTCTGGTTCGAGA	TCGGACCAGGCTTCATTCCCC	134	24298680
	vvi-miR167c	Un	7495686	7495775	+	TGAAGCTGCCAGCATGATCTC		17196	
	vvi-miR167d	Un	7490478	7490622	+	TGAAGCTGCCAGCATGATCT-	GGTCATGCCCTGACAGCCTCACT	2773	87
miR167	vvi-miR167e	5	5845370	5845489	+	TGAAGCTGCCAGCATGATCT-	AGATCATGTGGCAGTTTCACC	2773	2214
miR168	vvi-miR168	2	17944786	17944947	-	TCGCTTGGTGCAGGTCGGGAA	CCCGCCTTGCATCAACTGAAT	98080	48883
	vvi-miR169e	14	25082720	25082864	-	TAGCCAAGGATGACTTGCCT--		626	
miR169	vvi-miR169g	8	21104448	21104568	+	CAGCCAAGGATGACTTGCCGA	CCGGCAAGTTGTCTTTGGCTAC	11	417

Family	miRNA	chr[a]	start[a]	end[a]	strand[a]	5p sequence	3p sequence	Abundance 5p[b]	Abundance 3p[b]
	vvi-miR169k	11	16108538	16108658	+	CAGCCAAGGATGACTTGCCGG	GGCAGGTTGTCCTTGGCTACC	77	1273
	vvi-miR169r	11	16415128	16415239	+	TGAGTCAAGGATGACTTGCCG	GGCAAGTTGACTTGACTCAGT	4	532
	vvi-miR169t	11	16399564	16399676	+	CGAGTCAAGGATGACTTGCCG	GGCAAGTTGACTTGACTCAGT	2	532
	vvi-miR169u	11	16409398	16409510	+	TGAGTCAAGGATGACTTGCCG	GGCAAGTTGACTTGACTCTGT	4	137
	vvi-miR169w	14	29685578	29685790	+	CAGCCAAGGATGACTTGCCGG		77	
	vvi-miR169w.1	14	29685578	29685790	+	TGTGGGGATGAGGTTATGTGGTG	TTTCTGCCTCATCTTCTCATG	208	386
	vvi-miR169x	17	355697	355851	-	TAGCCAAGGATGACTTGCCT-	CAGTCATTCCTTCGGCTAAACT	626	600
	vvi-miR171b	12	5542396	5542529	-		---TTGAGCCGCGTCAATATCTCC		4243
	vvi-miR171e	11	5203303	5203435	+	CGATGTTGGTGAGGTTCAATC	---TTGAGCCGCGCCAATATCACT	412	903
	vvi-miR171f	9	7012483	7012615	+	CGATGTTGGTGAGGTTCAATC	TTGAGCCGCGCCAATATCACT	412	903
miR171	vvi-miR171h	17	1828650	1828753	-		---TTGAGCCGCGCCAATATCCCG		228
	vvi-miR172c	13	3217492	3217656	+	GGAGCATCATCAAGATTCACA	GGAATCTTGATGATGCTGCAG	334	1077
miR172	vvi-miR172d	8	12667173	12667279	+		TGAGAATCTTGATGATGCTGC--		2569
miR2111	vvi-miR2111	8	15368664	15368748	-	TAATCTGCATCCTGAGGTCTA	GTCCTCTGGTTGCAGATTACT	939	39
miR2950	vvi-miR2950	7	14340405	14340517	+	ATTCCATCTCTTGCACACTGG-	TGGTGTGCACGGGATGGAATA	6772	866
	vvi-miR319b	1	4189562	4189755	+		TTGGACTGAAGGGAGCTCCC-		538637
	vvi-miR319c	2	855548	855756	-	AGAGCTTTCTTCAGTCCACTC	TTGGACTGAAGGGAGCTCCC-	754	538637
	vvi-miR319e	11	4317223	4317329	+		TTTGGACTGAAGGGAGCTCCT		74092
	vvi-miR319f	6	9137286	9137416	+	AGCTGCCGACTCATTCATTCA	TTGGACTGAAGGGAGCTCCC-	1226	538637
miR319	vvi-miR319g	17	3675979	3676209	-		TTGGACTGAAGGGAGCTCCC-		538637
miR3623	vvi-miR3623	18	24650004	24650148	+	TCACAAGTTCATCCAAGCACCA	TGGTGCTTGGACGAATTTGCT	123542	24390
miR3624	vvi-miR3624	8	2139181	2139402	-	TAGTATGCTGCTGTCTTTAGA	TCAGGGCAGCAGCATACTACT	43	11606
miR3625	vvi-miR3625	17	2378212	2378319	-	TTCCAGCAGTCATCTCCAAGG	CGGGAGATGACTACTGGAAGC	85	1628
miR3627	vvi-miR3627	14	28302559	28302681	-	TTGTCGCAGGAGAGACGGCACT	TCGCCGCTCTCCTGTGACAAG	2119	5705
miR3628	vvi-miR3628	13	16716076	16716181	-	ATGCGAGAGCCGTGCTTAGTA	CTAAGCACAGCTCTCGCATCC	125	799
	vvi-miR3629a	13	18253987	18254170	+	CGCATTTTCTCAGCAGCCAAG	TGGCTGCTGAGAAAATGTAGG-	27	1912
miR3629	vvi-miR3629c	17	822209	822338	+	TGGCTGCTGAGAAAATGTAGG-		1912	
miR3632	vvi-miR3632	14	23394889	23395015	+	GGATTGGGGGCCGATGGAAAGG	TTTCCCAGACCCCCAATACCAA	5399	4088
	vvi-miR3633a	17	5521913	5522060	+	GGAATGGATGGTTAGGAGAG	TTCCTATACCACCCATTCCCTA	102848	5952
miR3633	vvi-miR3633b	17	5521557	5521691	+	GGAATGGGTGGCTGGGATCT-	GTTCCCATGCCATCCATTCCTA	15449	3499
miR3634	vvi-miR3634	17	5681202	5681314	-	GGCATATGTGTGACGGAAAGA	TTTCCGACTCGCACTCATGCCGT	1695	7058807
miR3635	vvi-miR3635	18	27357610	27357786	+	GGCATGTGTGGGGCATAATAG	ATTATGTCCCACACATGCCTC	1231	2099

Family	miRNA	chr[a]	start[a]	end[a]	strand[a]	5p sequence	3p sequence	Abundance 5p[b]	Abundance 3p[b]
miR3637	vvi-miR3637	16	20659663	20659834	-	ATTTATGTATTGTGTTTTGTCGGA	TTTCGACAAGACACAATGCATAAA	488	111
	vvi-miR3639	9	21823045	21823186	+	ATTGACTTCTGAAAGGCTAAAAGC	GAGCTTTTGGCTTCTCAGAAGTCA	808	269
miR3639	vvi-miR3639.1	9	21823045	21823186	+	AACCTGTTTGGATTGACTTCTGAA	TCAGAAGTCAATCCAAACAAGGTC	3	8
miR3640	vvi-miR3640	16	11986830	11987020	+	ACCTGATTGGTGATGCTTTTTTGG	ATCGAAAAGGCATCATCAATCAGG	3925	1684
miR390	vvi-miR390	6	8159504	8159673	+	AAGCTCAGGAGGGATAGCGCC	CGCTATCTATCCTGAGTTTCA	38339	189
	vvi-miR393a	16	17247172	17247327	-	TCCAAAGGGATCGCATTGATC	ATCATGCTATCCCTTAGGAAC	13555	649
miR393	vvi-miR393b	13	4265132	4265213	+	TCCAAAGGGATCGCATTGATC		13555	
miR394	vvi-miR394c	18	3551261	3551361	-	TTGGCATTCTGTCCACCTCC--		6417	
	vvi-miR395a	1	6527921	6528019	+		CTGAAGTGTTTGGGGGAACTC		11329
	vvi-miR395c	1	6499899	6500020	-	GTTCCCTTGACCACTTCACTG	CTGAAGTGTTTGGGGGAACTC	1316	11329
	vvi-miR395d	1	6512760	6512848	+		CTGAAGTGTTTGGGGGAACTC		11329
	vvi-miR395e	1	6505233	6505354	+	GTTCCCTTGACCACTTCACTG	CTGAAGTGTTTGGGGGAACTC	1316	11329
	vvi-miR395f	1	6489527	6489642	+	GTTCCCCTGACCACTTCACTG	CTGAAGTGTTTGGGGGAACTC	1316	11329
	vvi-miR395g	1	6482098	6482213	+	GTTCCCCTGAGCACTTCATTG	CTGAAGTGTTTGGGGGAACTC	194	11329
	vvi-miR395h	1	6566637	6566758	+	GTTCCCTTGACCACTTCACTG	CTGAAGTGTTTGGGGGAACTC	1316	11329
	vvi-miR395i	1	6562627	6562742	+	GTTCCCCTGACCACTTCACTG	CTGAAGTGTTTGGGGGAACTC	1316	11329
	vvi-miR395j	1	6553011	6553126	+	GTTCCCCTGACCACTTCACTG	CTGAAGTGTTTGGGGGAACTC	1316	11329
	vvi-miR395k	1	6536764	6536885	+	GTTCCCTTGACCACTTCACTG	CTGAAGTGTTTGGGGGAACTC	1316	11329
	vvi-miR395l	1	6559083	6559199	+	GTTCCCCTGACCACTTCACTG	CTGAAGTGTTTGGGGGAACTC	1316	11329
miR395	vvi-miR395n	17	6408994	6409134	-		-TGAAGAGTCTGGAGGAACTCT		43
	vvi-miR396a	9	7372506	7372649	-	TTCCACAGCTTTCTTGAACT-	GCTCAAGAAAGCTGTGGGAGG	35942	3217
	vvi-miR396b	11	5246778	5246913	+	TTCCACAGCTTTCTTGAACTT	GTTCAAGAAAGCTGTGGGAAA	120399	5334
	vvi-miR396c	4	5119576	5119713	-	TTCCACAGCTTTCTTGAACTG	GCTCAGGAAAGTTGTGGAACA	104918	798
miR396	vvi-miR396d	11	5253095	5253244	-	TTCCACAGCTTTCTTGAACTG	GTTCAATAAAGCTGTGGGAAG	104918	9615
miR397	vvi-miR397a	Un	11971875	11972030	-	TCATTGAGTGCAGCGTTGATG	TTGGCGCTGCACTCAATCATG	13817	8008
	vvi-miR398a	1	731589	731729	+	CAAGGGAGTGGCACCTGAGAACA	----TTCTCAGGTCACCCCTTTGGG	382	203
	vvi-miR398b	6	16503529	16503646	-	GGTGTGACCTGAGAATCACATG	TGTGTTCTCAGGTCGCCCCTG	242	284479
miR398	vvi-miR398c	6	15575566	15575683	+	GGAGTGACCTGAGAATCACATG	TGTGTTCTCAGGTCGCCCCTG	225	284479
	vvi-miR399a	10	2989448	2989546	+		TGCCAAAGGAGAATTGCCCTG		193
	vvi-miR399b	16	15618693	15618839	-	GGGCACCTCTTTCTTGGCAG	TGCCAAAGGAGAGTTGCCCTG	53	819
	vvi-miR399c	15	15232197	15232281	+		TGCCAAAGGAGAGTTGCCCTG		819
miR399	vvi-miR399h	10	2983543	2983634	+		TGCCAAAGGAGAATTGCCCTG		193

Family	miRNA	chr[a]	start[a]	end[a]	strand[a]	5p sequence	3p sequence	Abundance 5p[b]	Abundance 3p[b]
	vvi-miR399i	2	4101798	4101922	+		CGCCAAAGGAGAGTTGCCCTG		6558
	vvi-miR403a	5	65232	65376	+	AGTTTGTGCGTGAATCAAACC	TTAGATTCACGCACAAACTCG	136	200843
	vvi-miR403b	5	600176	600266	+		TTAGATTCACGCACAAACTCG		200843
	vvi-miR403d	5	166477	166567	+		TTAGATTCACGCACAAACTCG		200843
	vvi-miR403e	5	168084	168228	+	AGTTTGTGCGTGAATCAAACC	TTAGATTCACGCACAAACTCG	136	200843
miR403	vvi-miR403f	7	4179658	4179795	-	AGTTTGTGCGTGACTCTAAAA	TTAGATTCACGCACAAACTCG	2675	200843
miR408	vvi-miR408	7	5011920	5012056	+	CGGGGACGAGGTAGTGCATGG	ATGCACTGCCTCTTCCCTGGC	24612	63260
	vvi-miR477	1	22740264	22740350	+	---TCCCTCAAAGGCTTCCAATTT		409	
miR477	vvi-miR477b	2	1237529	1237808	+	ACTCTCCCTCAAGGGCTTCTG-	CGAAGTCTTTGGGGAGAGTGG	237	1512
miR479	vvi-miR479	16	21573744	21573866	+	TGTGGTATTGGTTCGGCTCATC	CGAGCCGAACCAATATCACTC	8385	57764
miR482	vvi-miR482	1	3865560	3865686	+	CATGGGCGGTTTGGTAAGAGG	TCTTACCAACACCTCCCATTCC	415921	127869
	vvi-miR535a	7 random	1392238	1392368	-	TGACAACGAGAGAGAGCACGC	GTGCTCTCTCTCGCTGTCATA	24425	197
	vvi-miR535b	Un	25369688	25369818	-	TGACAACGAGAGAGAGCACGC	GTGCTCTCTCTCGCTGTCATA	24425	197
miR535	vvi-miR535c	7 random	1346353	1346483	-	TGACAACGAGAGAGAGCACGC	GTGCTCTCTGTCGCTGTCATA	24425	197

[a] Refers to the genomic localization on the GRAPE_IGGP12Xv1 genome sequence

[b] Sum of TP5M values from 68 libraries

- Missing nucleotides

Nucleotides that differ from the annotated sequence are shown in red.

Newly annotated miRNA are shown in blue.
