# Supplementary material for: miRVine: a microRNA expression atlas of grapevine based on small RNA sequencing
Source: BMC Genomics. 2015 May 16;16(1):393. doi: 10.1186/s12864-015-1610-5 (PMC4434875; doi:10.1186/s12864-015-1610-5)
Supplement: Additional file 4: — Novel vvi-miRNAs identified in Corvina-derived libraries. List of novel Vitis vinifera miRNAs identified in small RNA libraries of Corvina-derived tissues. A temporary name has been given to each miRNA sequence either using a sequential numbering associated with the abbreviation mirC (miRNA Candidate) for those similar to other known plant miRNAs or using “grape-m” with a random numbering for those completely new candidates. [file 12864_2015_1610_MOESM4_ESM.pdf]

miRNA name	chr[a]	start[a]	end[a]	strand[a]	5p sequence	nt[b]	3p sequence	nt[b]	Abundance 5p[c]	Abundanc 3p[c]
vvi-miRC1446*	14	24560621	24560731	-	TCTGAACTCTCTCCCTCATGGC	22			1134	
vvi-miRC159d	15	18461450	18461639	-			TTTGGTTTGAAGGGAGCTCT	20		125
vvi-miRC159e	15	18466298	18466488	-			TTTGGTTTGAAGGGAGCTCT	20		125
vvi-miRC159f	15	18473287	18473482	-			TTGGAGTGAAGGGAGCTCTA	20		2722
vvi-miRC166i*	7	19450158	19450352	+	GGAATGTTGTCTGGCTCGAGG	21	TCGGACCAGGCTTCATTCCCC	21	215923	24298680
vvi-miRC171j	18	1502294	1502428	+			TGATTGAGCCGTGCCAATATC	21		1390
vvi-miRC171h	Un	19112172	19112315	+	TATTGGCCCGGTTCACTCAGA	21	TGATTGAGCCGTGCCAATATC	21	48174	1390
vvi-miRC171h.1	Un	19112172	19112315	+			TTGATTTGATTGAGCCGTGCC	21		15468
vvi-miRC172e*	13	6181355	6181500	-	GTAGCATCATCAAGATTCACA	21	TGAGAATCTTGATGATGCTGC	21	161	2569
vvi-miRC172f*	6	17652397	17652534	-	GTAGCATCATCAAGATTCACA	21	TGAGAATCTTGATGATGCTGC	21	161	2569
vvi-miRC2275	1	11721643	11721752	-			TTTAGTTTCCTCCAATATCTCA	22		48
vvi-miRC2275a	14	29265081	29265177	-	AGAATTGGAGGATAGCAAACA	21			69	
vvi-miRC3627a	14	28619565	28619652	+	TTGCCGCACGAGAGATGGCACC	22			572	
vvi-miRC3629d	14	3031591	3031773	+	TGGCTGCTGAGAAAATGTAGG	21			1912	
vvi-miRC3629e	19	10543925	10544090	-	TGTTTGGTTGTCGAGAAAATG	21			162	
vvi-miRC390a*	8	9571397	9571544	+	AAGCTCAGGAGGGATAGCGCC	21	CGCTATCCATCCTGAGTTTC	20	38339	1096
vvi-miRC396e*	1	1997798	1998004	+	TTCCACGGCTTTCTTGAACTT	21	TTCAAGAAAGCCGTGGGAAAA	21	74082	1446
vvi-miRC477c*	19	12889963	12890080	+	TCCCTCAAAGGCTTCCAATTT	21	GTTGGAAGCCGGTGGGGGACC	21	409	27451
vvi-miRC477d*	19	13141671	13141788	+	TCCCTCAAAGGCTTCCAATTT	21	GTTGGAAGCCGGTGGGGGACC	21	409	27451
vvi-miRC477e*	19	13510093	13510210	+	TCCCTCAAAGGCTTCCAATTT	21	GTTGGAAGCCGGTGGGGGACC	21	409	27451
vvi-miRC477f*	19	18678338	18678455	+	TCCCTCAAAGGCTTCCAATTT	21	GTTGGAAGTCGGTGGGGGAAC	21	409	23526
vvi-miRC477g*	19	18881351	18881468	-	TCCCTCAAAGGCTTCCAATTT	21	GTTGGAAGCCGGTGGGGGACC	21	409	27451
vvi-miRC477h	Un	16672963	16673080	-	TCCCTCAAAGGCTTCCAATTT	21	GTTGGAAGCCGGTGGGGGACC	21	409	27451
vvi-miRC5225	17	5928104	5928188	+	TCCCAGGAGAGATGGCACCTGC	22			496	
vvi-miRC530*	6	17896112	17896288	+	TCTGCATTTGCACCTGCACCT	21	AGGTGCAGGTGAAGGTGCAGA	21	10586	567
vvi-miRC530a	8	18999725	18999889	-	TCTGCATTTGCACCTGCACCT	21			10586	
vvi-miRC535f	Un	10281947	10282060	-	TGACAAAGAGAGAGAGCACACT	22	GTGCTCTCTCTCGCTGTCATA	21	19	197
vvi-miRC535g	Un	11733968	11734081	+	TGACAAAGAGAGAGAGCACACT	22	GTGCTCTCTCTCGCTGTCATA	21	19	197
vvi-miRC7122	5	6820719	6820833	-	TTACACAGAGAGATGACGGTGG	22	ACCGTCTTTCTCTGTATAAGC	21	1617	1906
vvi-miRC827*	5	24742113	24742240	-	TTTTGTTGCTGGTCATCTAGTC	21	TTAGATGATCATCAACAAACA	21	6996	14564
grape-m0033	10	4660930	4661046	+			TAAGGACTAAATTGGTAGACC	21		1671
grape-m0073	10	9062839	9062920	+			TTAGGTTGCTGTTTGGAGAGT	21		348

miRNA name	chr[a]	start[a]	end[a]	strand[a]	5p sequence	nt[b]	3p sequence	nt[b]	Abundance 5p[c]	Abundanc 3p[c]
grape-m0256	10	11642347	11642413	-	GTTACAGAGTCCGATCGGTC	20			84	
grape-m0423	11	10360043	10360216	+	CAGCTCCAATAGCGTATATT	20			41	
grape-m0447	11	14373348	14373442	+			ATTGATTGGTTGGGATAGGTT	21		96
grape-m0477	11	16106464	16106625	+	TGAAGAGGTTGAGAGTGGAATG	22			468	
grape-m0479	11	16112952	16113113	+	TGAAGAGGTTGAGAGTGGAATG	22			468	
grape-m0593	11	9977583	9977656	-	AGCTGCTGAGATTGGAAGGTT	21			43	
grape-m0693	12	1561898	1561988	+	TTGGGATTTTTGAATTGAACA	21			88	
grape-m1119*	12	19456317	19456411	-			AGAAGAACAAGTAGACTGAGC	21		1067
grape-m1120	12	19459384	19459478	-			AGAAGAACAAGTAGACTGAGC	21		1067
grape-m1191	13	4841635	4841840	+	CTGAATGGATCAAGGAGTTGT	21			75	
grape-m1203	13	6511567	6511663	+			CACTTCCTTAACTTTTCGGTG	21		514
grape-m1354	13	20548804	20548919	+	TTCCTCTTTATTTTCTTTCCA	21			81	
grape-m1364*	13	21037998	21038153	+	TTGATTACAGTGGGATGGGACC	22			265	
grape-m1458	13	4835472	4835625	-			CGGGGTTTCGCCGGACGGTG	20		164
grape-m1556	13	15466712	15466871	-	AAATACTGATTATTCGGTTCAG	22			146	
grape-m1565	13	16723472	16723557	-	ACGGTCAATGATTTAGCAAGA	21			141	
grape-m1612	13	21698084	21698183	-	CAAGGCTGAATAGAAAACCAGT	22			92	
grape-m1662	13	24202107	24202197	-	TCGAGAAAGGGCAAGAATGGC	21			202	
grape-m1944	14	28011371	28011525	+			GTAAGGACTTTCAAAGACGGGA	22		81
grape-m2136	14	14721543	14721732	-	TACCAAGGTCGAGGATTGACT	21			1915	
grape-m2196	14	21179859	21179967	-	GAAGATCCGGAATTCTTAGAGC	22			43	
grape-m2399	15	8297135	8297221	+	GGTGGTTATGGAGTGGGCTTG	21			17531	
grape-m2483	15	16588282	16588444	+			TGATGAGGATGTAGCTGTTGCA	22		99
grape-m2616	15	5849845	5849981	-	TTGCTTCGGAAGGTTTACTTC	21			245	
grape-m2704	15	11940120	11940226	-	TATCATGAACGCTCGAGCGCC	21			36	
grape-m2830	16	3217509	3217617	+	TTAGGATGGTGAGAAAGAGTG	21			103	
grape-m2885	16	11236436	11236513	+	AAAGGATCATCGGGCTGTTGCT	22			92	
grape-m3054	16	7129520	7129733	-	CAAAGAAAGAAAGCTGGCAATC	22			34	
grape-m3147	16	15763862	15764097	-	TTTCACTCATCTTCCCTAAGG	21			83	
grape-m3245	17	5523009	5523155	+	AATTGGAGAGTAGGAAAGCTT	21	TCTTTCCTACTCCTCCCATTCC	22	528739	322286
grape-m3409	17	2609227	2609373	-	AGGGTCTTACTGGGTAGATT	20	TATCCAAGTTTAGATCCTTG	22	5452	3056
grape-m3453	17	6445059	6445223	-			TGAATGGAAGGCGAAGAGGA	20		89

miRNA name	chr[a]	start[a]	end[a]	strand[a]	5p sequence	nt[b]	3p sequence	nt[b]	Abundance 5p[c]	Abundanc 3p[c]
grape-m3469*	17	7265141	7265283	-	TGAGATAAGTCTGCTGCT	21	TGAGTAGTGGACTATCGCATG	21	134	1722
grape-m3618	18	4447466	4447594	+			ACCAATGGTGTTCTCAGGACA	21		184
grape-m3772	18	24220613	24220765	+			ATGGACTATGTCAAGGGACT	20		82
grape-m3778	18	24586743	24586858	+			CTTGAGAGCTCCAATGGATGG	21		1040
grape-m3816	18	27782605	27782800	+			TTGATGGTAGAGATTCTAAAG	21		740
grape-m3841	18	714470	714623	-			TGGCGCTGATTCTTCTTGTAG	21		231
grape-m3966	18	12957736	12957884	-	ATTAATAGATTGTCAAGTGT	20			82	
grape-m4051	18	23054619	23054804	-			TAAGATATCTCTATTGGCATG	21		172
grape-m4178	19	4078806	4078999	+			CGTGAAGATCATCAAGTTGGA	21		1822
grape-m4179	19	4080388	4080575	+			CGTGAAGATCATCAAGTTGGA	21		1822
grape-m4180	19	4104952	4105148	+			CGTGAAGATCATCAAGTTGGA	21		1822
grape-m4207	19	7695221	7695343	+			TTCACTTCAAGCTTGGACTAG	21		57
grape-m4241	19	11013461	11013568	+			TCGGAAAATGCACAAAATTT	20		621
grape-m4250	19	11833169	11833287	+	AGGGTGTGAGAAATGTGAAGA	21			119	
grape-m4269	19	13951057	13951244	+			TGTCTGAGAATTGCTTCAAGG	21		1029
grape-m4277	19	15131212	15131329	+	AGGGTGTGAGAAATGTGAAGA	21			119	
grape-m4281	19	15466582	15466713	+	CATGTCACTCGTCAAGCCAAG	21			2907	
grape-m4363	19	23202090	23202163	+			AGCGGACCGGTCCGGCGAAG	20		58
grape-m4455	19	7308948	7309138	-			GGTTGAGCACAAGGGGACAAG	21		88
grape-m4520	19	14726502	14726626	-	CGTGAAGATCATCAAGTTGGA	21			1822	
grape-m4553*	19	18872706	18872824	-	TCCCTCAAAGGCTTCCAATTT	21	GTTGGAAGTCGGTGGGGGACC	21	409	20202
grape-m4560	19	19300048	19300174	-	TTTGGCAGTGATTCTAGGAAA	21			120	
grape-m4757	1	14504569	14504688	+	TTGGAGATCGTCTTGAGGGGA	21			75	
grape-m4795	1	17956448	17956517	+	AACATGAATGAGCACTCTCCT	21			43	
grape-m4809	1	19488979	19489200	+	TTTCACTCATCTTCCCTAAGG	21			83	
grape-m4846	1	361208	361343	-	CCATCGGTTTGAAATTTCATC	21			68	
grape-m4908	1	7871756	7871876	-			AGGCAAAGGTAGGATTGGGTCA	22		37
grape-m4912	1	8218404	8218578	-			GATGAAGTGGGTGCTGTCTC	20		82
grape-m5176	Un	16227504	16227644	+	TTGAGAGCTAAAGGAAGGCCT	21			88	
grape-m5236	Un	23153364	23153458	+			ATTGATTGGTTGGGATAGGTT	21		96
grape-m5380	Un	38428043	38428125	+			GGAGATTATGTTGTGGATGG	20		84
grape-m5408	Un	40428338	40428432	+			ATTGATTGGTTGGGATAGGTT	21		96

miRNA name	chr[a]	start[a]	end[a]	strand[a]	5p sequence	nt[b]	3p sequence	nt[b]	Abundance 5p[c]	Abundanc 3p[c]
grape-m5638	Un	21377636	21377769	-	ATTGATTGGTTGGGATAGGTT	21			96	
grape-m5747	Un	33339005	33339208	-			CGTGAAGATCATCAAGTTGGA	21		1822
grape-m5836	Un	40321003	40321097	-			ATTGATTGGTTGGGATAGGTT	21		96
grape-m6251	2	13048510	13048691	-			ATTTTAGGTTTTTTGGTTTTA	21		65
grape-m6520	18 random	3407778	3407992	-			TTGGCTAAGTTCATTGTTGCAG	22		148
grape-m6580	3	4131893	4131994	+			TGTTCATAAGATGTGATGTG	20		36
grape-m6581	3	4154887	4155010	+	TATGAACTCCTGTATGTGCA	20			39	
grape-m6603	3	6148832	6148899	+	CCAAACGAAGCGTACTGAGG	20			37	
grape-m6710	3	15367124	15367400	+	ATAGATCAGATAGTCGACGCC	21			168	
grape-m6790	3	1771706	1771804	-			GTTAGTCTGGAATGGAGGGGC	21		92
grape-m6845	3	7752610	7752756	-			TAAGTTGATTTCGATTGTGGAT	22		84
grape-m6873	3	10530619	10530800	-	CGGGATTTTGGTAACTTAACC	21			1538	
grape-m6905	3	13241508	13241588	-	GCCGCGTTGGAGCAGGAGCTT	21			1513	
grape-m6953	3	17920034	17920105	-			GCTAGTACGTGTTGCAGGGGA	21		90
grape-m7008	4	3099245	3099408	+			TGTCCTTGACCTCGTTTTAAAG	22		645
grape-m7192	4	23525665	23525775	+			GGAAGAAGAGATGCATGCTC	20		43
grape-m7209*	4	1141381	1141521	-	ACAGTAGGAAATTGAAAGAGA	21			628	
grape-m7218	4	2265913	2266028	-	TGTAGGGAGTAGAATGCAGC	20	TACATTGGGCTCTCTTCTTC	20	16062	606
grape-m7388	4	20931852	20931953	-			GACTATTGCCGGTGTTGGAAGG	22		37
grape-m7484	5	2941942	2942051	+			TTGGCTGTTAAGTGTTCACT	20		33
grape-m7540	5	10459977	10460094	+	GGAAGATGTGTACGGATGGTGT	22			84	
grape-m7644*	5	20944390	20944559	+	AATATGGAGGACTGTGTTCTT	21			5765	
grape-m7670	5	22089877	22089947	+			TCTGAAGCTTCACCTAGAGACC	22		71
grape-m7692	5	24544386	24544456	+	AGCGAGTCGCTGGTCAAGCG	20			60	
grape-m7774	5	7401989	7402105	-			GGAGATTATGTTGTGGATGG	20		84
grape-m7843	5	15969599	15969708	-	ATGGGATGGAACATTATTCGT	21			564	
grape-m7969*	6	4485456	4485555	+			TCAAAAGAGAAAATGTGGATG	21		2770
grape-m8013	6	9147662	9147757	+	TTGACTTTGGTGTTTTGGACC	21			539	
grape-m8119	6	782864	782963	-	GGAGGATTGATCAGTGGGGC	20			153	
grape-m8278	6	19950730	19950847	-	TCCCTCAAAGGCTTCCAATTT	20	GCTGGAAGCCGATGGGGGACC	21	409	12122
grape-m8392	7	12636275	12636426	+			ATGGACTATGTCAAGGGACT	20		82
grape-m8399	7	13415540	13415652	+			AGATGTGGGGAATTGGAGCAAG	22		81

miRNA name	chr[a]	start[a]	end[a]	strand[a]	5p sequence	nt[b]	3p sequence	nt[b]	Abundance 5p[c]	Abundanc 3p[c]
grape-m8476	7	2176796	2176906	-			CATGATTAAAGAGCTCATGT	20		88
grape-m8937*	8	14593863	14594095	-	CCGAGGGAGAGAGCGAGAGGA	21	CTCTCACTCTGTCCCTCAGTC	21	1986	358
grape-m8943	8	15175935	15176139	-	TCAATGTAACCTTGGGAGCTG	21			90	
grape-m8960	8	17310763	17310986	-			TAAACGGAGATTGTTTGACT	20		84
grape-m8993	8	21905716	21905814	-	TCGTAGTGGCTGCGACAGCTCC	22			3823	
grape-m9163	9	16109677	16109880	+	TGAGATTGAAGACTTTGATGT	21			12142	
grape-m9201	9	20314929	20315048	+	TTGGAGATCGTCTTGAGGGGA	21			75	
grape-m9269	9	3184788	3184924	-			TTCTTCTCACCGTTTCTCAGC	21		1344
grape-m9381	9	14373742	14373950	-	CGTTTCCCACAGACGGCGCCA	21			9845	
grape-m9401	9	16648813	16648934	-	TTGGAGATCGTCTTGAGGGGA	21			75	
grape-m9410	9	18443427	18443584	-	TCAGCCAATAGTTCATCGAGGC	22	CTCATGAATTGTTGGCTGATA	21	4613	382

[a] Refers to the genomic localization on the GRAPE_IGGP12Xv1 genome sequence.
[b] nucleotide, sequence length of the microRNA.
[c] Sum of TP5M values from 68 libraries.
*These miRNAs were also identified by Wang et al., 2011; Han et al., 2014, and Wang et al., 2014.
