# Supplementary material for: miRVine: a microRNA expression atlas of grapevine based on small RNA sequencing
Source: BMC Genomics. 2015 May 16;16(1):393. doi: 10.1186/s12864-015-1610-5 (PMC4434875; doi:10.1186/s12864-015-1610-5)
Supplement: Additional file 5: — Novel vvi-miRNAs identified in PN40024-derived libraries. List of novel Vitis vinifera miRNAs identified in small RNA libraries from tissues of the PN40024 clone. A temporary name has been given to each miRNA sequence either using a sequential numbering associated with the abbreviation miRC (miRNA Candidate) for those similar to other known plant miRNAs or using “grape-m” with a random numbering for those completely new candidates. [file 12864_2015_1610_MOESM5_ESM.pdf]

miRNA	chr[a]	star[a]	end[a]	strand[a]	5p sequence	nt[b]	3p sequence	nt[b]	Abundance 5p[c]	Abundance 3p[c]
vvi-miRC2118	19	9739626	9739720	+	GGTATGGGTGGGCCGGGAAAA	21	TTGCCGACTCCACCCATACCTA	22	23	12643
vvi-miRC3629f	19	5056194	5056436	+	TTGGTTACCGAGAAAACGCAG	21			498	
vvi-miRC3635a	18	27417341	27417475	+			ATTATGTCCCACACATGCCTC	21		79
vvi-miRC391*	6	3976406	3976497	-	TTCGCAGGAGAGATGACGCCGT	22			58	
vvi-miRC399j	10	2978899	2979015	-			TGCCAAAGGAGATTTGCCCCG	21		45
vvi-miRC482a*	14	19755468	19755588	-	GGAATGGGCTGATTGGGATA	20	TTTTCCCAATGCCGCCCATTCC	22	7663	2722
vvi-miRC5225a*	14	28620631	28620733	+	TCTGTCGCAGGAGAGATGATGC	22			755	
vvi-miRC529*	5	19124470	19124728	-			AGAAGAGAGAGAGTACAGCTA	21		1111
vvi-miRC535d	Un	10256638	10256736	-	TGACAACGAGAGAGAGCATGC	21			58	
vvi-miRC535e	Un	10277197	10277295	-	TGACAACGAGAGAGAGCATGC	21			58	
vvi-miRC535h	Un	11738797	11739027	+			TGACAACGAGAGAGAGCATGC	21		58
vvi-miRC535i	Un	11799052	11799267	+			TGACAACGAGAGAGAGCATGC	21		58
vvi-miRC535j*	Un	11820795	11820908	+	TGACAAAGAGAGAGAGCACAC	21			1116	
vvi-miRC535k	Un	37604874	37604972	-	TGACAACGAGAGAGAGCATGC	21			58	
vvi-miRC771	14	17188645	17188772	-			TGAGTCTAATCGTCGTATGGC	21		124
grape-m0193	13	20234980	20235070	+			GTATCGGCAAAACTACTCTGG	21		176
grape-m0221	13	9274642	9274828	-	TGGTTATAGAGAAGCTGAGCG	21			1436	
grape-m0250	13	21869432	21869586	-			TGTAGAGACTCTAGTGATGGG	21		264
grape-m0281*	14	10959011	10959186	+	CTAGAGATTGTGGATTAGGCT	21			1614	
grape-m0283	14	12116273	12116391	+			TGGAGGAGAATCTTGAACTGC	21		178
grape-m0297	14	22335451	22335595	+	TTCTCAGCTACTAATATCAAG	21			60	
grape-m0534	17	5681196	5681433	-	GTGGAGTTTTGTGAAGCTTAT	21	TGGTTTCAGATCACTCCTCCC	21	19	49
grape-m0555	18	5500395	5500665	+	TGGTTATAGAGAAGCTGAGCG	21			1436	
grape-m0563	18	13887168	13887304	+			TGAGCTCTAACATTGGTGAAG	21		31
grape-m0640	19	3974695	3974841	+			CTTGATCTTTCTTTGACAGTG	21		112
grape-m0641	19	4034775	4034921	+			CTTGATCTTTCTTTGACAGTG	21		112
grape-m0642	19	4078944	4079090	+			CTTGATCTTTCTTTGACAGTG	21		112
grape-m0657*	19	12889975	12890065	+			GTTGGAAGCCGGTGGGGGACC	21		273
grape-m0721*	1	3865560	3865686	+	CATGGGCGGTTTGGTAAGAGG	21	TCTTACCAACACCTCCCATTCC	22	10905	7499
grape-m0738	1	7530676	7530798	+			TGGGGTACGAACTAGAGGTGG	21		102
grape-m0941	2	14372620	14372799	-	TGCAGACCGTTTTGGAGGAGG	21			91	
grape-m0954	13 random	2504393	2504483	+			GTATCGGCAAAACTACTCTGG	21		176

miRNA	chr[a]	star[a]	end[a]	strand[a]	5p sequence	nt[b]	3p sequence	nt[b]	Abundance 5p[c]	Abundance 3p[c]
grape-m1073*	4	378829	378904	-			GGAGTGAAATTGCAGTGACGG	21		73
grape-m1185	6	5662499	5662579	+			GTGGCTTCACATCATGGCTTTA	22		56
grape-m1209	6	13593086	13593171	-	TTGAGATTTCTGAATGCAGGC	21			1297	
grape-m1235	7	13327670	13327812	+			TAAGGTCCCGAGTTCGAATCC	21		148
grape-m1351	9	4633115	4633209	+	AAAGATTGTTGCTGAAGAGTG	21			221	
grape-m1398	9	12277787	12277891	-			TAAGATTGTTGTTGAAGAATG	21		559

[a] Refers to the genomic localization on the GRAPE_IGGP12Xv1 genome sequence.
[b] nucleotide, sequence length of the microRNA.
[c] Sum of TP5M values from 2 libraries.
*These miRNAs were also identified by Wang et al., 2011; Han et al., 2014, and Wang et al., 2014.
