# Supplementary material for: miRVine: a microRNA expression atlas of grapevine based on small RNA sequencing
Source: BMC Genomics. 2015 May 16;16(1):393. doi: 10.1186/s12864-015-1610-5 (PMC4434875; doi:10.1186/s12864-015-1610-5)

vvi-miRC1446

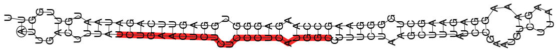

vvi-miRC159d

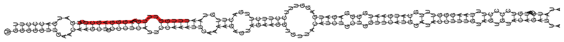

vvi-miRC159e

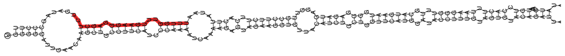

vvi-miRC159f

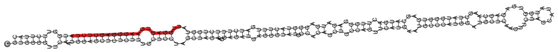

vvi-miRC166i

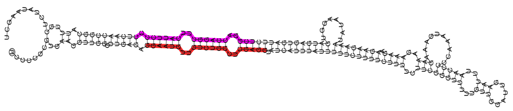

vvi-miRC171j

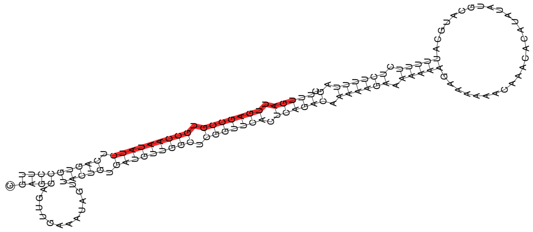

vvi-miRC172e

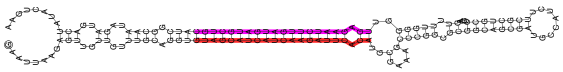

grape-m3966

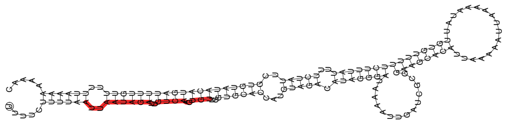

grape-m4051

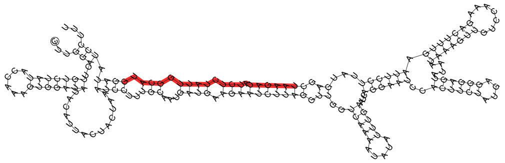

grape-m4178

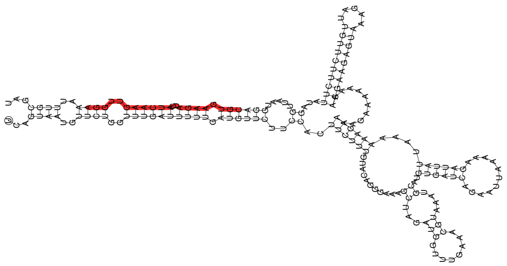

grape-m4179

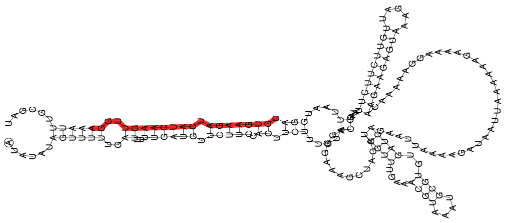

grape-m4180

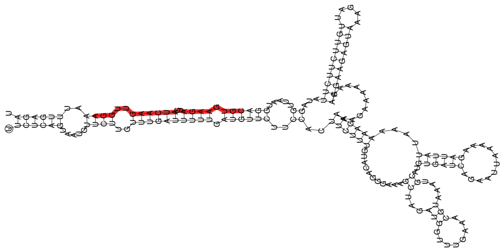

grape-m4207

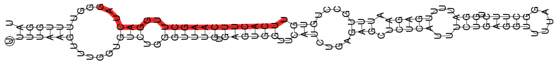

grape-m4241

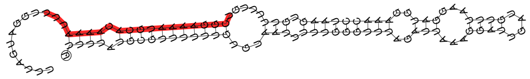

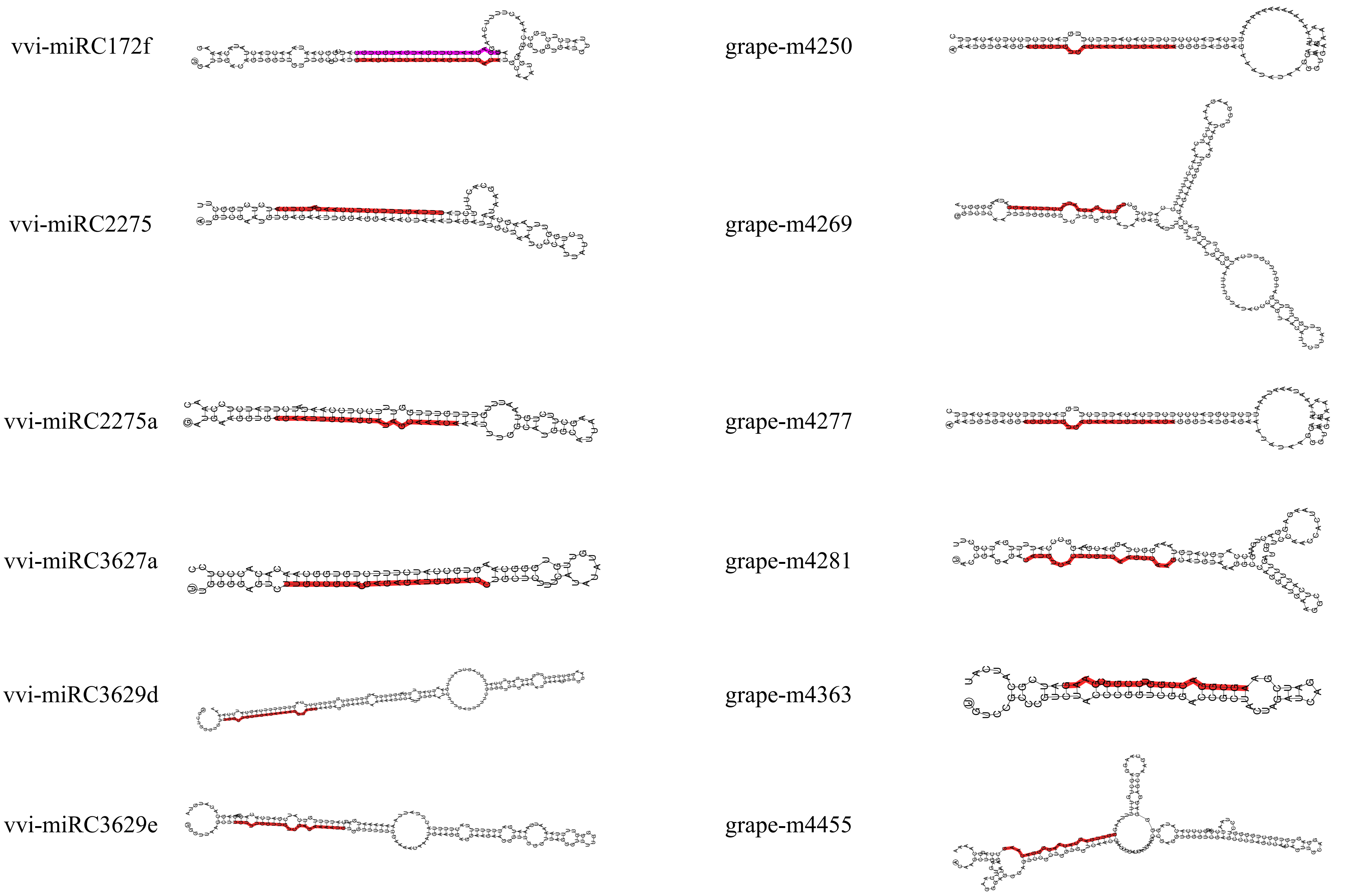
vvi-miRC172f
grape-m4250
vvi-miRC2275
grape-m4269
vvi-miRC2275a
grape-m4277
vvi-miRC3627a
grape-m4281
vvi-miRC3629d
grape-m4363
vvi-miRC3629e
grape-m4455
vvi-miRC390a
grape-m4520

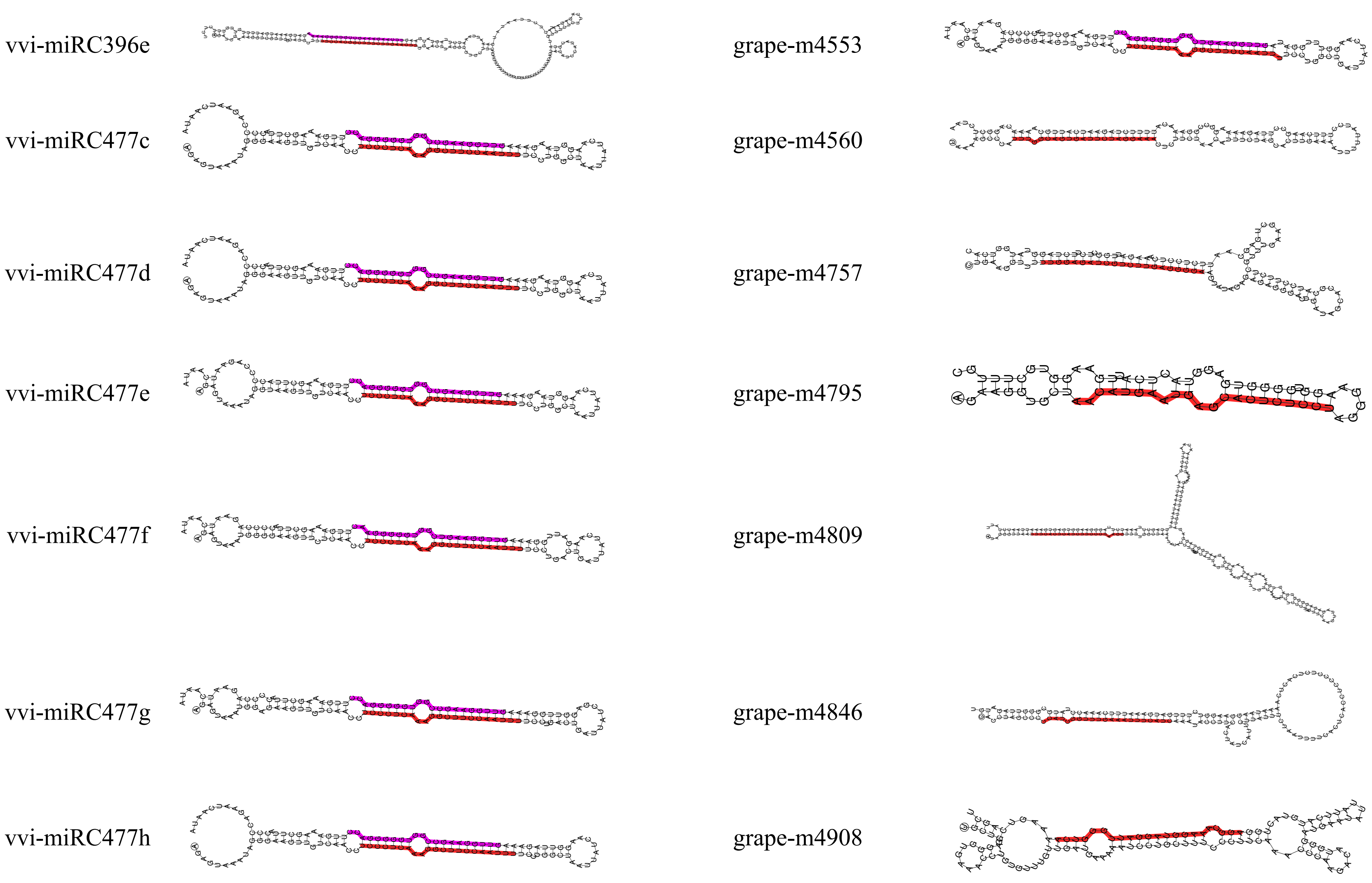
vvi-miRC396e
grape-m4553
vvi-miRC477c
grape-m4560
vvi-miRC477d
grape-m4757
vvi-miRC477e
grape-m4795
vvi-miRC477f
grape-m4809
vvi-miRC477g
grape-m4846
vvi-miRC477h
grape-m4908

vvi-miRC5225

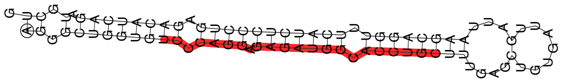

vvi-miRC530

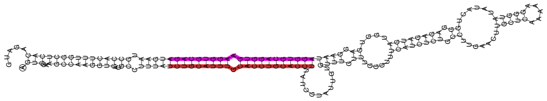

vvi-miRC530a

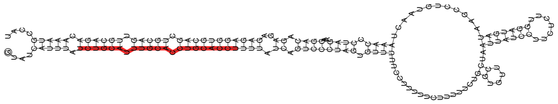

vvi-miRC535f

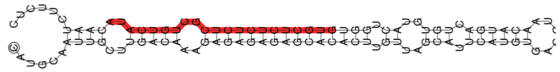

vvi-miRC535g

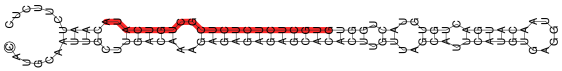

vvi-miRC7122

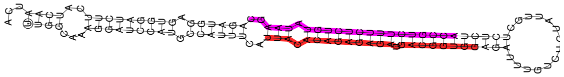

grape-m4912

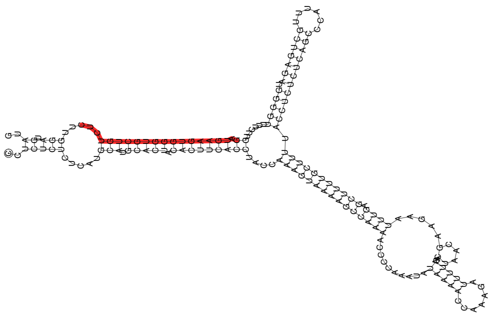

grape-m5176

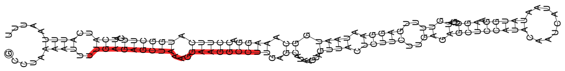

grape-m5236

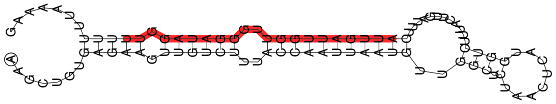

grape-m5380

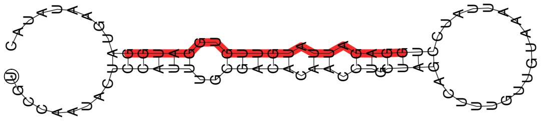

grape-m5408

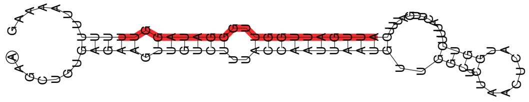

grape-m5638

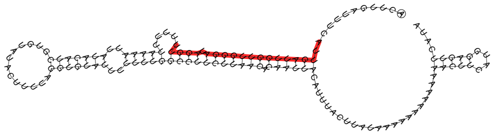

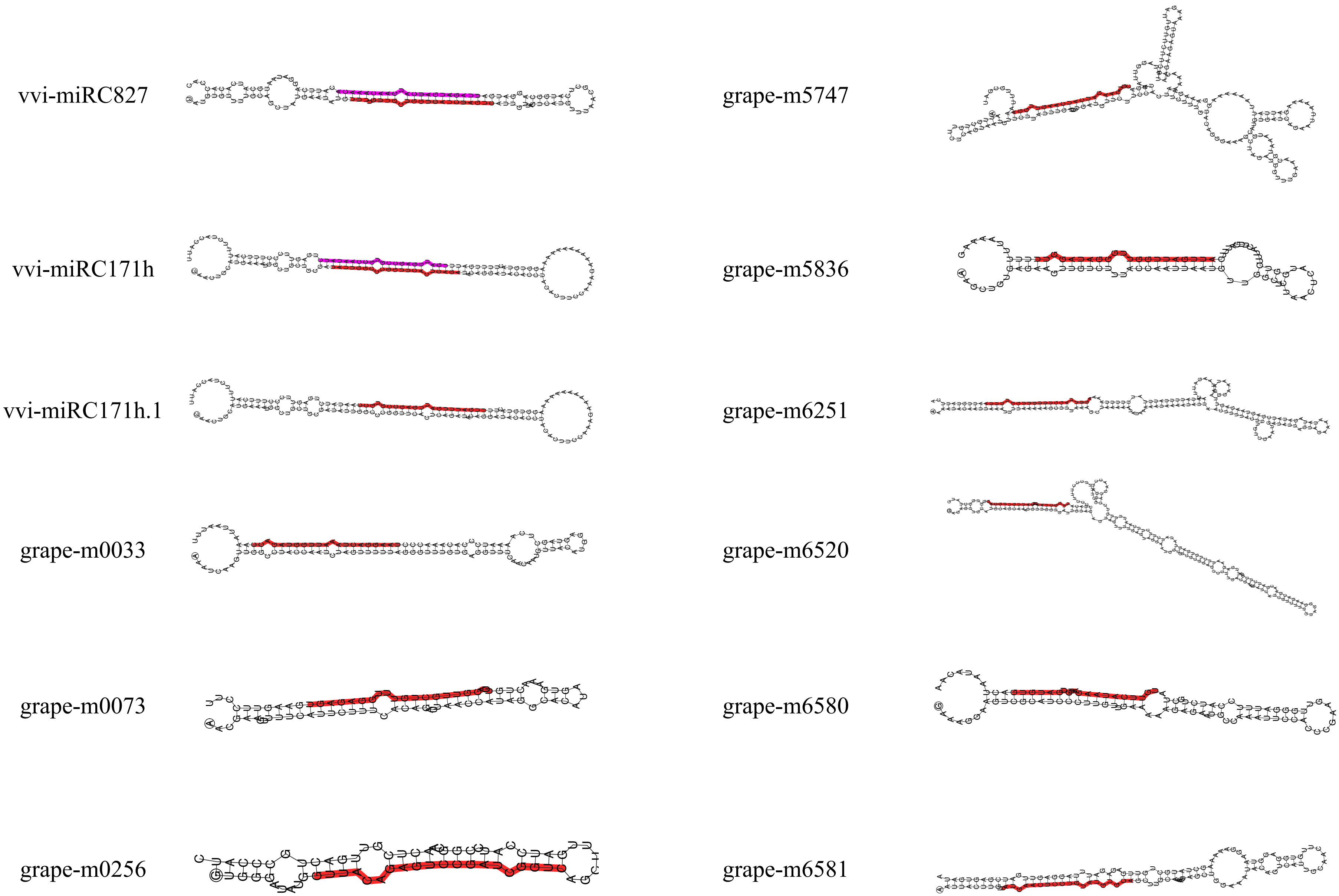
vvi-miRC827
grape-m5747
vvi-miRC171h
grape-m5836
vvi-miRC171h.1
grape-m6251
grape-m0033
grape-m6520
grape-m0073
grape-m6580
grape-m0256
grape-m6581

grape-m0423

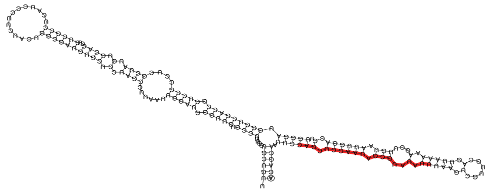

grape-m0447

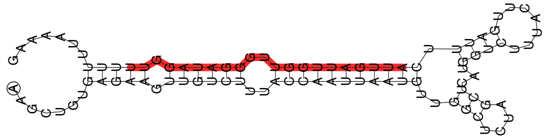

grape-m0477

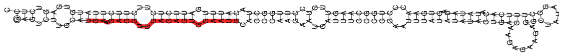

grape-m0479

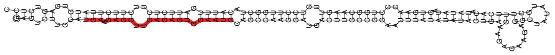

grape-m0593

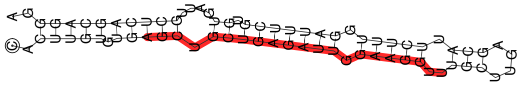

grape-m6603

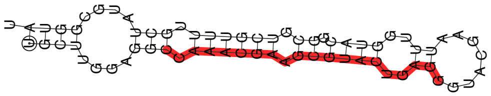

grape-m6710

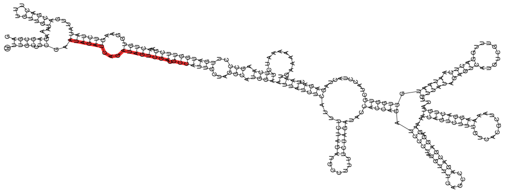

grape-m6790

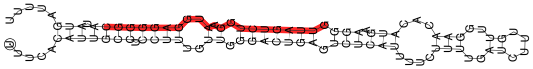

grape-m6845

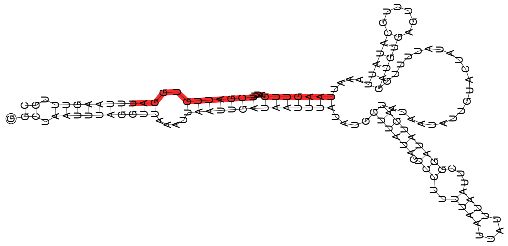

grape-m6873

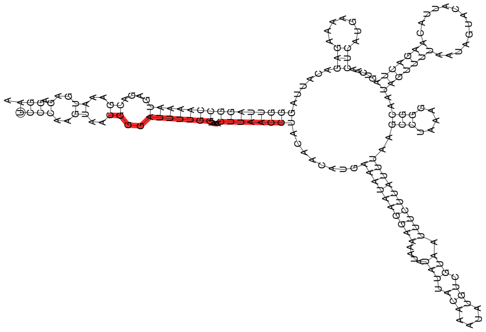

grape-m0693

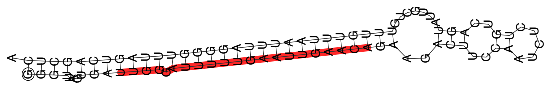

grape-m1119

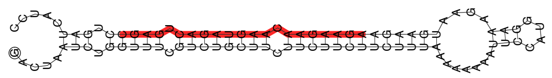

grape-m1120

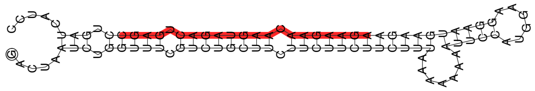

grape-m1191

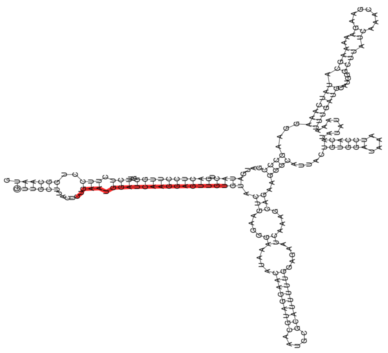

grape-m1203

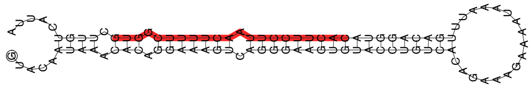

grape-m6905

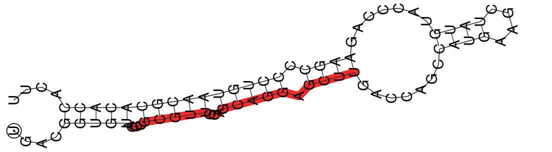

grape-m6953

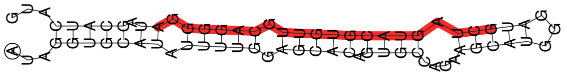

grape-m7008

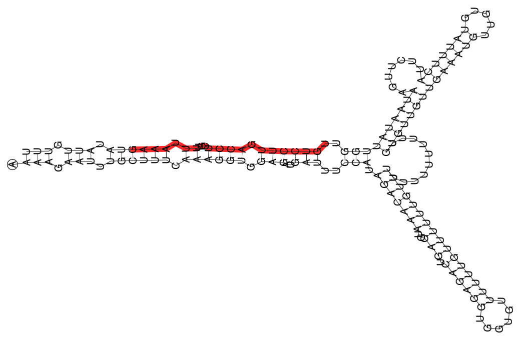

grape-m7192

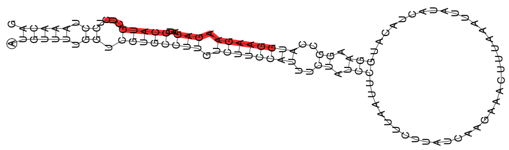

grape-m7209

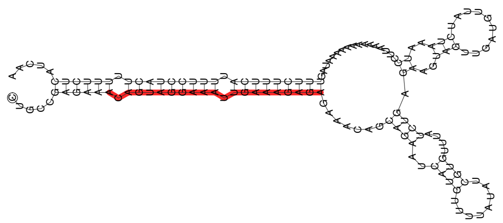

grape-m1354

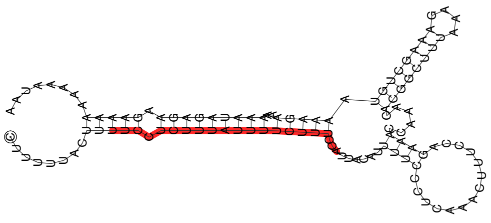

grape-m1364

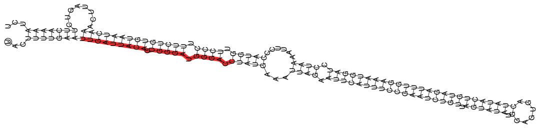

grape-m1458

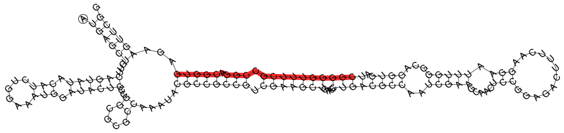

grape-m1556

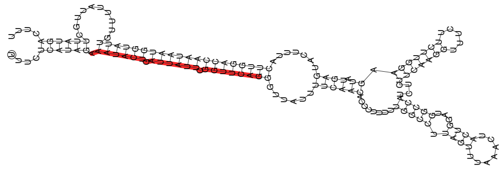

grape-m1565

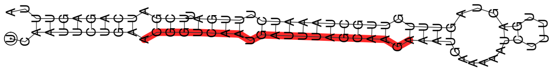

grape-m1612

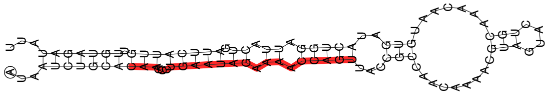

grape-m7218

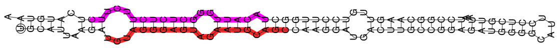

grape-m7388

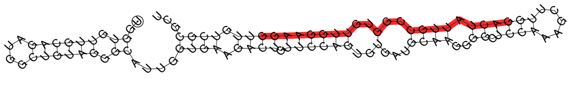

grape-m7484

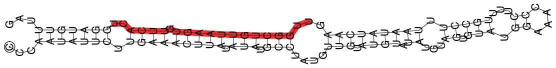

grape-m7540

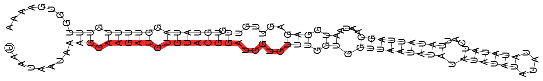

grape-m7644

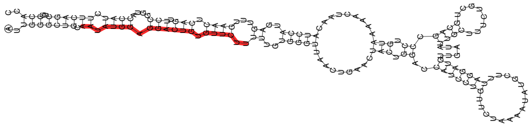

grape-m7670

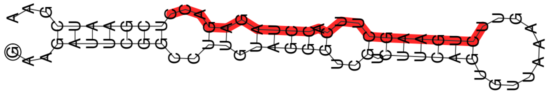

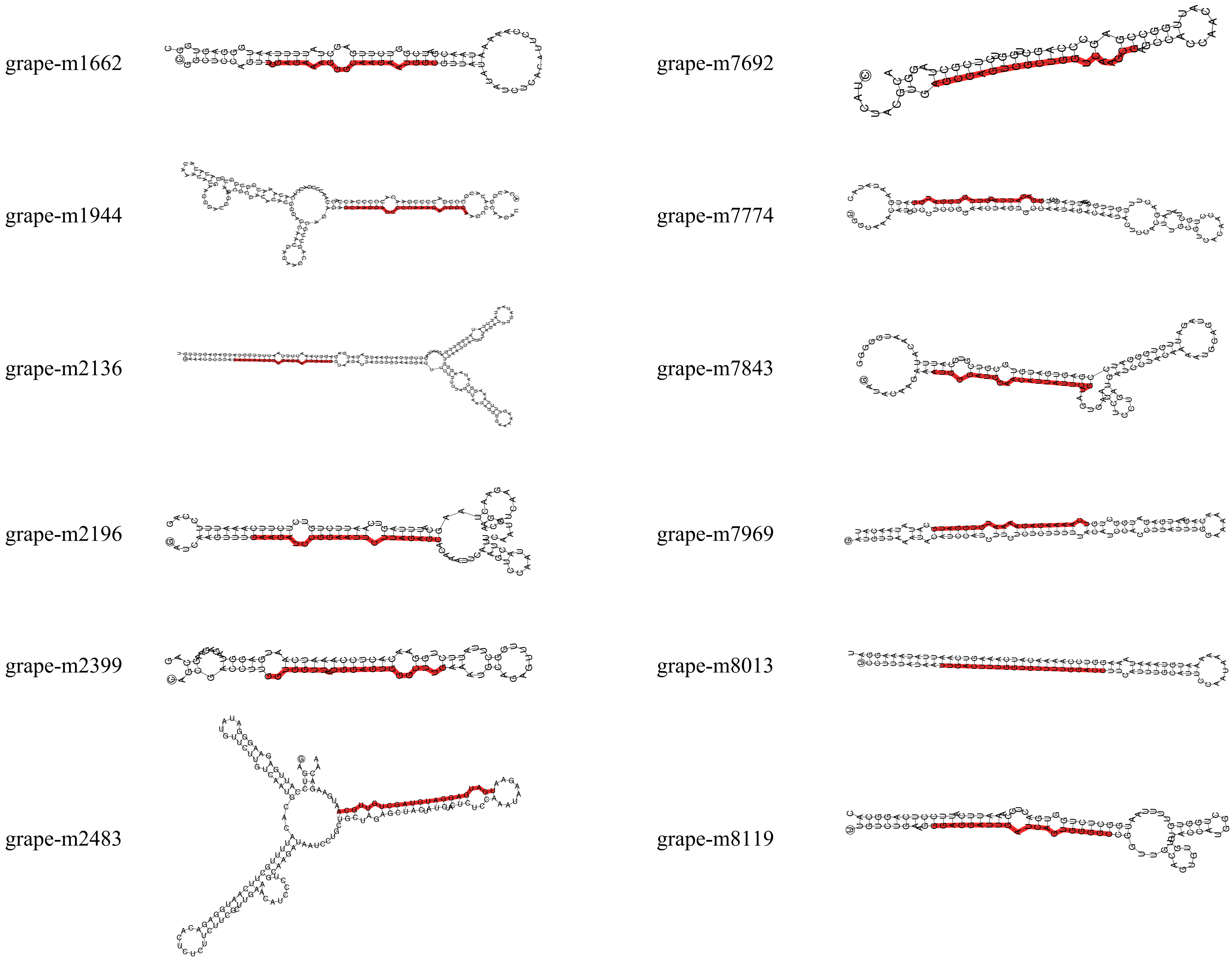
grape-m1662
grape-m7692
grape-m1944
grape-m7774
grape-m2136
grape-m7843
grape-m2196
grape-m7969
grape-m2399
grape-m8013
grape-m2483
grape-m8119

grape-m2616

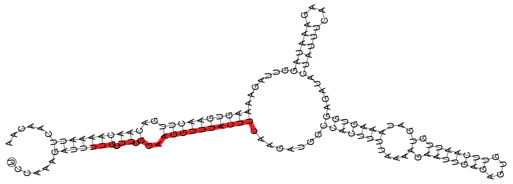

grape-m2704

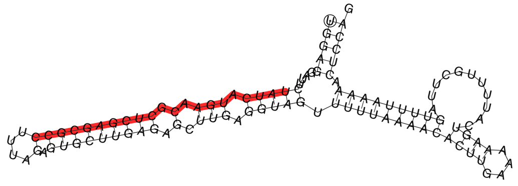

grape-m2830

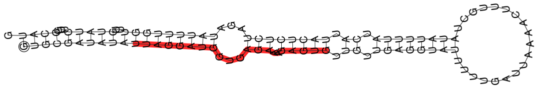

grape-m2885

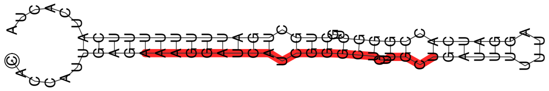

grape-m3054

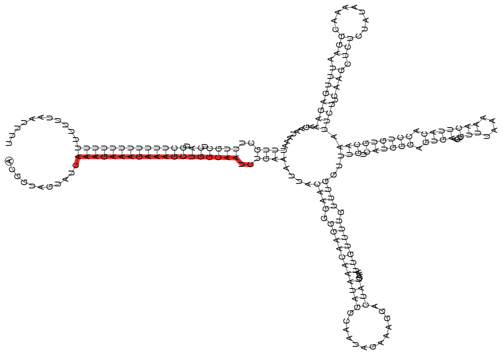

grape-m3147

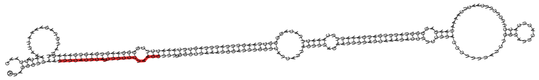

grape-m8278

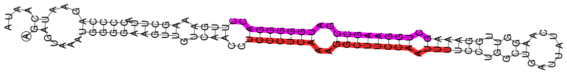

grape-m8392

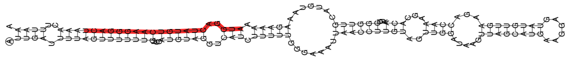

grape-m8399

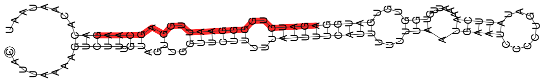

grape-m8476

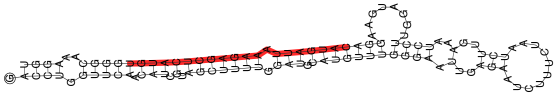

grape-m8937

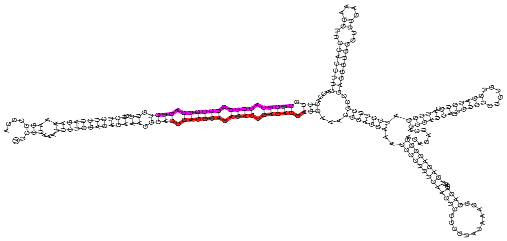

grape-m8943

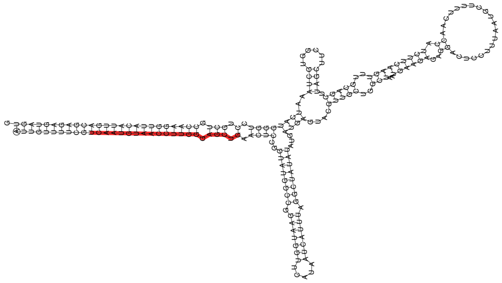

grape-m3245

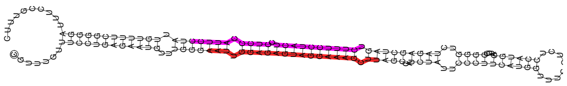

grape-m3409

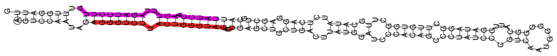

grape-m3453

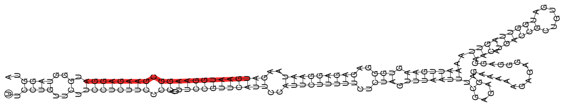

grape-m3469

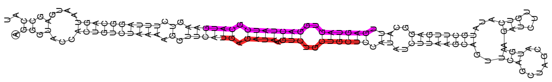

grape-m3618

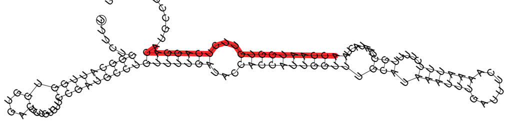

grape-m3772

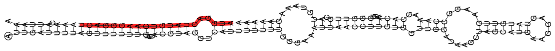

grape-m8960

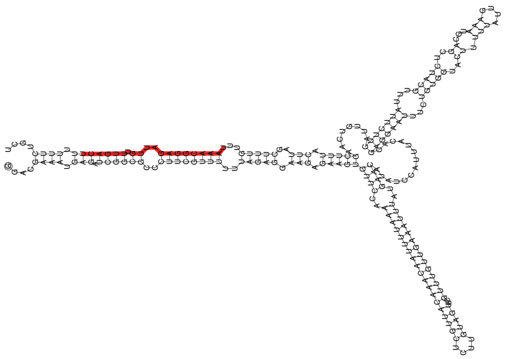

grape-m8993

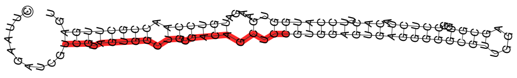

grape-m9163

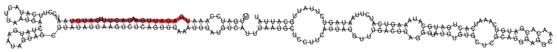

grape-m9201

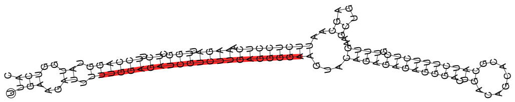

grape-m9269

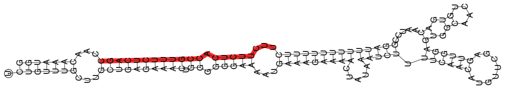

grape-m9381

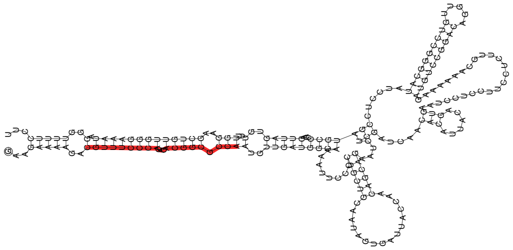

grape-m3778

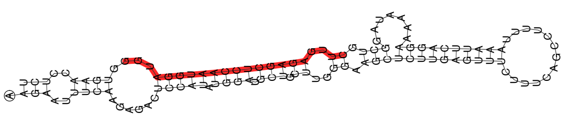

grape-m3816

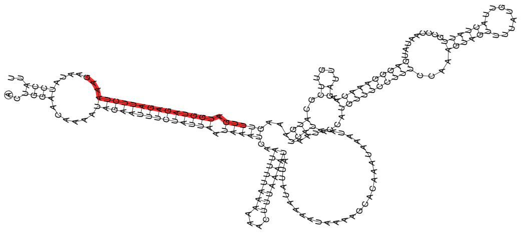

grape-m3841

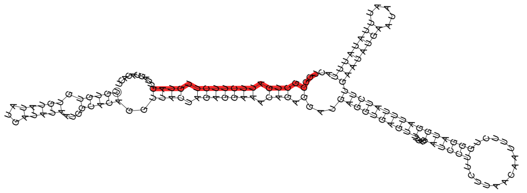

grape-m9401

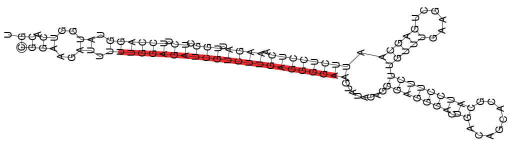

grape-m9410

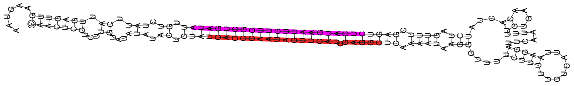

Supplement: Additional file 6: — Secondary structure of novel miRNAs identified in Corvina-derived libraries. List of all RNA secondary structures of novel miRNA precursors, predicted using the RNA folding tool of the UEA sRNA toolkit – Plant version [43]. The sequence of the mature miRNA is highlighted in red and the complementary sequence (miRNA*) is highlighted in pink when present. [file 12864_2015_1610_MOESM6_ESM.pdf]
