# Supplementary material for: miRVine: a microRNA expression atlas of grapevine based on small RNA sequencing
Source: BMC Genomics. 2015 May 16;16(1):393. doi: 10.1186/s12864-015-1610-5 (PMC4434875; doi:10.1186/s12864-015-1610-5)

vvi-miRC2118

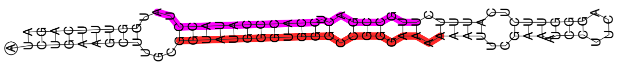

grape-m0283

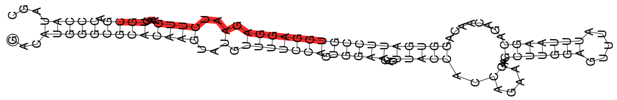

vvi-miRC3629f

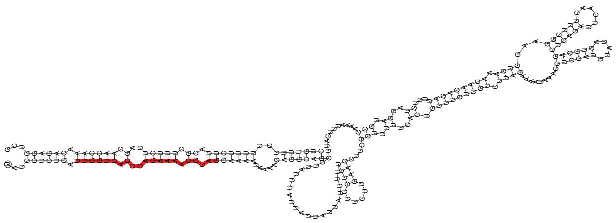

grape-m0297

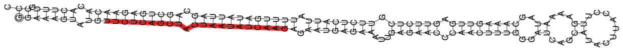

vvi-miRC3635a

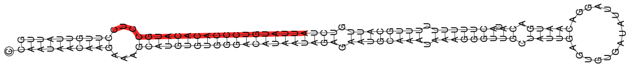

grape-m0534

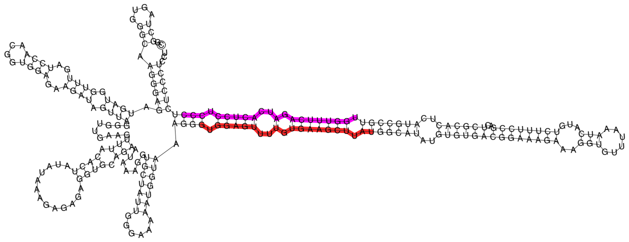

vvi-miRC391

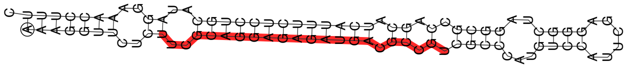

grape-m0555

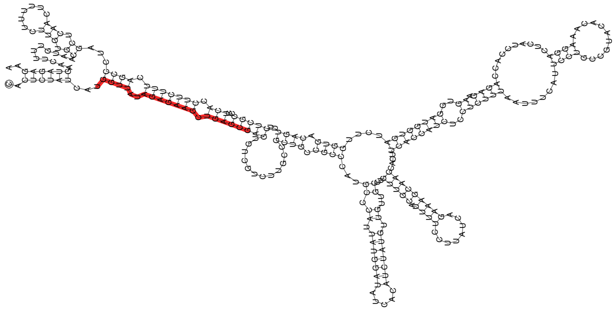

vvi-miRC399j

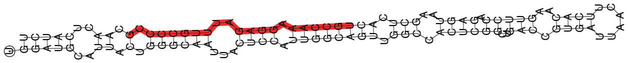

grape-m0563

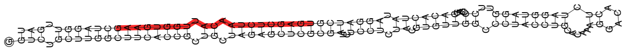

vvi-miRC482a

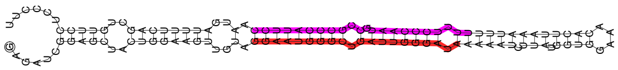

grape-m0640

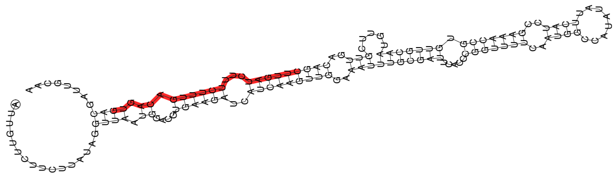

vvi-miRC5225a

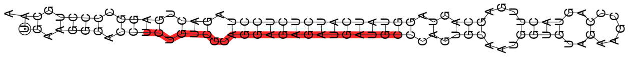

grape-m0641

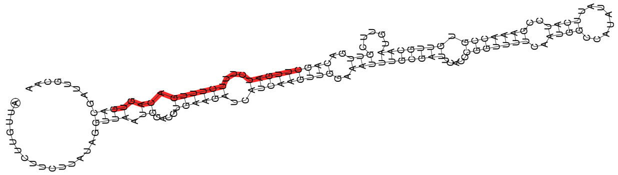

vvi-miRC529

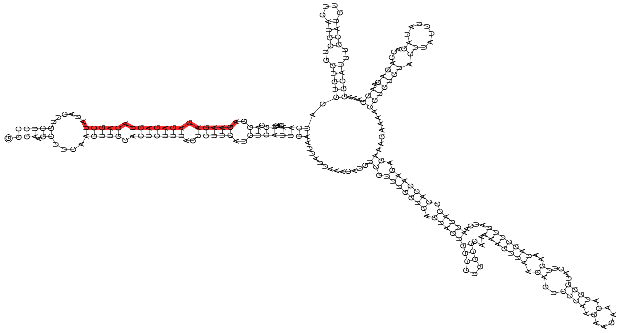

grape-m0642

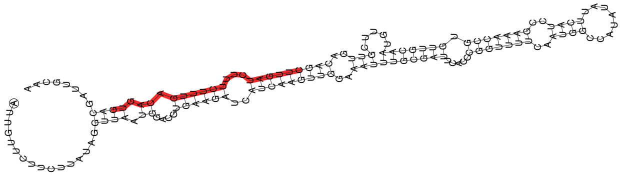

vvi-miRC535d

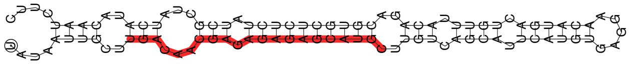

grape-m0657

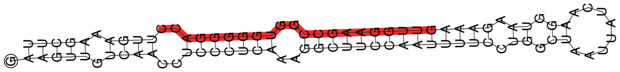

vvi-miRC535e

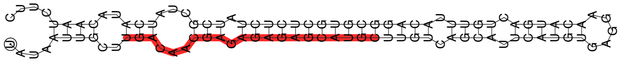

grape-m0721

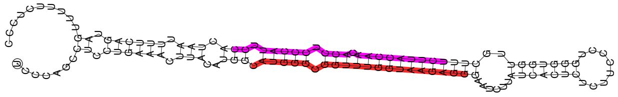

vvi-miRC535h

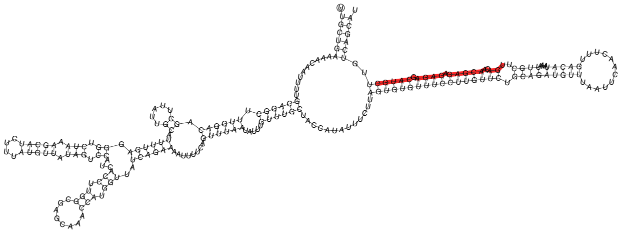

grape-m0738

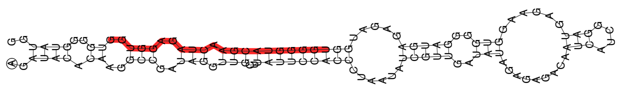

vvi-miRC535i

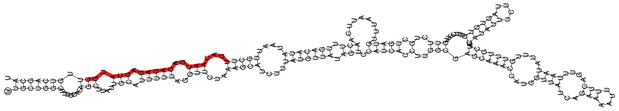

grape-m0941

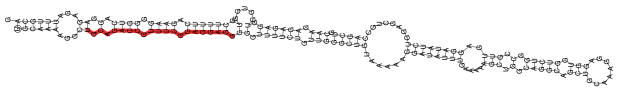

vvi-miRC535j

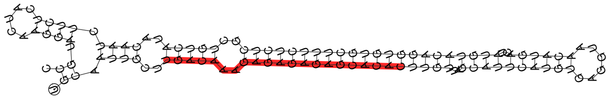

grape-m0954

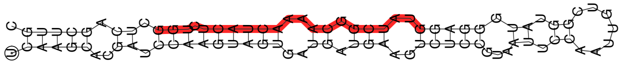

vvi-miRC535k

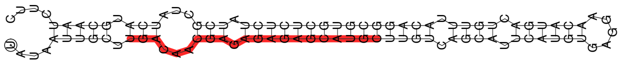

grape-m1073

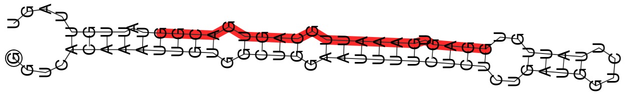

vvi-miRC771

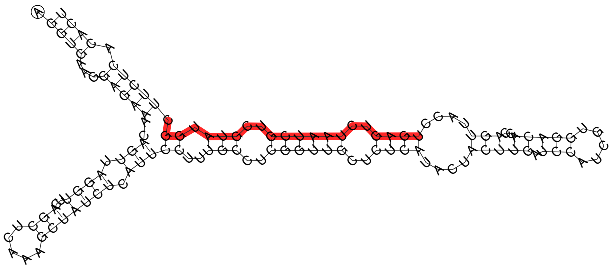

grape-m1185

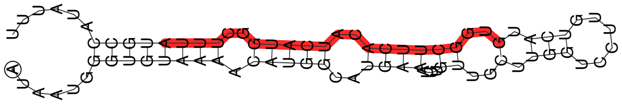

grape-m0193

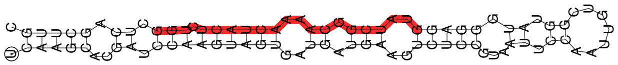

grape-m1209

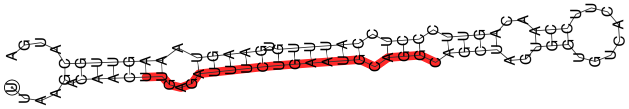

grape-m0221

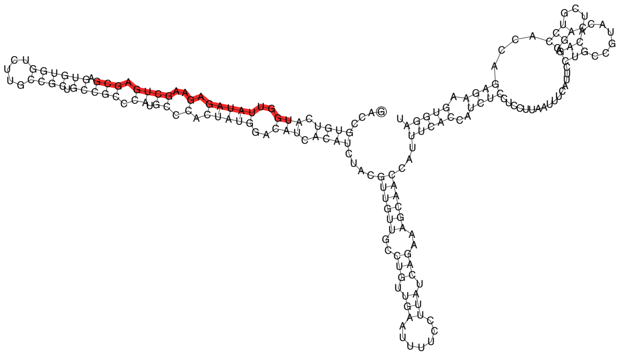

grape-m1235

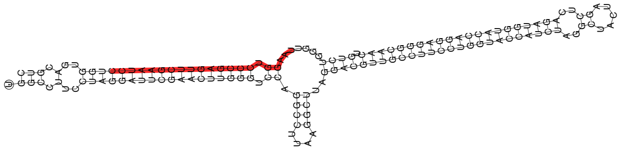

grape-m0250

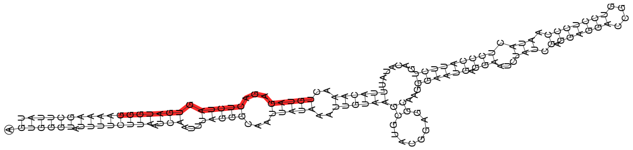

grape-m1351

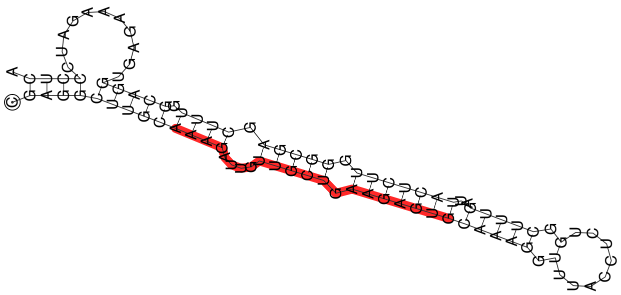

grape-m0281

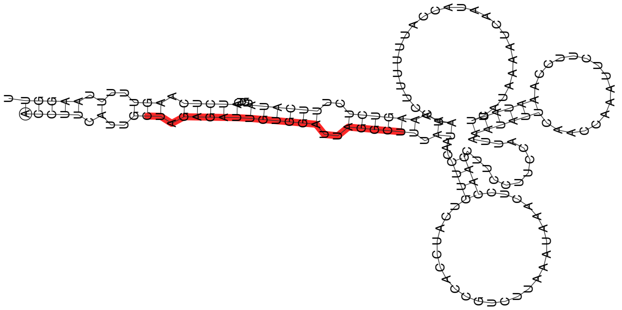

grape-m1398

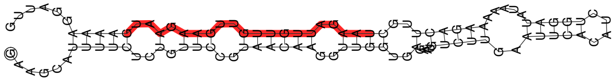

Supplement: Additional file 7: — Secondary structure of novel miRNAs identified in PN40024-derived libraries. List of all RNA secondary structures of novel miRNA precursors, predicted using the RNA folding tool of the UEA sRNA toolkit – Plant version [43]. The sequence of the mature miRNA is highlighted in red and the complementary sequence (miRNA*) is highlighted in pink when present. [file 12864_2015_1610_MOESM7_ESM.pdf]
