# Supplementary material for: miRVine: a microRNA expression atlas of grapevine based on small RNA sequencing
Source: BMC Genomics. 2015 May 16;16(1):393. doi: 10.1186/s12864-015-1610-5 (PMC4434875; doi:10.1186/s12864-015-1610-5)
Supplement: Additional file 8: — Putative targets of known and novel vvi-miRNAs. List of putative targets as identified by TARGET FINDER. Only targets with score ≥ 3 are listed. GRAPE_IGGP_v1 12X assembly of Vitis vinifera genome and the v2 annotation were used. [file 12864_2015_1610_MOESM8_ESM.pdf]

miRNA	Target id	Alignment	Score	Range	Target gene functional annotation
grape-m0033	VIT_212s0034g02556.1	target 5' GGTCTA-TAATTTAGTTCTTG 3'<br>:::::: .::::::::.:::.<br>miRNA 3' CCAGATGGTTAAATCAGGAAT 5'	3	222-241	Protein
grape-m0033	VIT_212s0035g00003.1	target 5' GGTCTA-TAATTTAGTTCTTG 3'<br>:::::: .::::::::.:::.<br>miRNA 3' CCAGATGGTTAAATCAGGAAT 5'	3	222-241	Protein
grape-m0073	VIT_204s0008g06230.1	target 5' ACTCTAGCAGACAGCAACCTAA 3'<br>::::: ::.::::::::::::<br>miRNA 3' TGAGA-GGTTTGTCGTTGGATT 5'	3	557-578	Large proline-rich protein bat3
grape-m0073	VIT_204s0008g06230.2	target 5' ACTCTAGCAGACAGCAACCTAA 3'<br>::::: ::.::::::::::::<br>miRNA 3' TGAGA-GGTTTGTCGTTGGATT 5'	3	509-530	Large proline-rich protein bat3
grape-m0073	VIT_204s0008g06230.3	target 5' ACTCTAGCAGACAGCAACCTAA 3'<br>::::: ::.::::::::::::<br>miRNA 3' TGAGA-GGTTTGTCGTTGGATT 5'	3	557-578	Large proline-rich protein bat3
grape-m0073	VIT_204s0008g06230.4	target 5' ACTCTAGCAGACAGCAACCTAA 3'<br>::::: ::.::::::::::::<br>miRNA 3' TGAGA-GGTTTGTCGTTGGATT 5'	3	557-578	Large proline-rich protein bat3
grape-m0256	VIT_200s0375g00020.1	target 5' GACTGGTCGGACTTTGTAAA 3'<br>:::.:.:::::::.:::::<br>miRNA 3' CTGGCTAGCCTGAGACATTG 5'	3	478-497	Tmv resistance protein n
grape-m0423	VIT_206s0004g06795.1	target 5' AAGATG-GCTATTGGAGCTG 3'<br>:: ::. :::::::::::::<br>miRNA 3' TTATATGCGATAACCTCGAC 5'	2.5	693-711	Polynucleotidyl ribonuclease h fold
grape-m0423	VIT_217s0000g08870.1	target 5' AAGAT-CGCTATTGGGGCTG 3'<br>:: :: :::::::::.::::<br>miRNA 3' TTATATGCGATAACCTCGAC 5'	3	934-952	Protein
grape-m0423	VIT_217s0000g08870.2	target 5' AAGAT-CGCTATTGGGGCTG 3'<br>:: :: :::::::::.::::<br>miRNA 3' TTATATGCGATAACCTCGAC 5'	3	934-952	Protein
grape-m0423	VIT_218s0001g03430.1	target 5' CAGATACGCTATTGGAGCTG 3'<br>: ::::::::::::::::::<br>miRNA 3' TTATATGCGATAACCTCGAC 5'	2	500-519	Flavonol synthase
grape-m0423	VIT_218s0001g03430.2	target 5' CAGATACGCTATTGGAGCTG 3'<br>: ::::::::::::::::::<br>miRNA 3' TTATATGCGATAACCTCGAC 5'	2	500-519	Flavonol synthase
grape-m0423	VIT_218s0001g04730.1	target 5' GGT-TGTGCTATTGGAGCTG 3'<br>..: :..:::::::::::::<br>miRNA 3' TTATATGCGATAACCTCGAC 5'	3	520-538	F-box kelch-repeat protein at1g22040-like
grape-m0693	VIT_206s0004g07800.1	target 5' TTTTCAAGATCAAAAATCCCAA 3'<br>: ::::: :::::::::::::<br>miRNA 3' ACAAGTT-AAGTTTTTAGGGTT 5'	3	16-37	Pentatricopeptide repeat-containing protein

miRNA	Target id	Alignment	Score	Range	Target gene functional annotation
grape-m1203	VIT_214s0060g00250.1	target 5' TACTG-AAAGTTGAGGAAGTG 3'<br>.::.: :::::.::::::::<br>miRNA 3' GTGGCTTTTCAATTCCTTCAC 5'	3	1682-1701	Grip and coiled-coil domain-containing
grape-m1354	VIT_205s0049g02250.1	target 5' TGGAAAGAAGAAAAAGAGGAA 3'<br>:::::::::.: :::::::::<br>miRNA 3' ACCTTTCTTTTATTTCTCCTT 5'	3	663-683	Surfeit locus protein 6
grape-m1354	VIT_209s0002g06590.1	target 5' TGGAGAAGAAAATAAAGAAGAA 3'<br>:::: ::::::::::::: :::<br>miRNA 3' ACCT-TTCTTTTATTTCTCCTT 5'	3	939-960	Udp-glycosyltransferase 75d1-like
grape-m1458	VIT_216s0100g00490.1	target 5' CAGTTTTCGGCGAAACCCCG 3'<br>:: . :.:::::::::::::<br>miRNA 3' GTGGCAGGCCGCTTTGGGGC 5'	3	24-43	Uncharacterized protein
grape-m1458	VIT_216s0100g00490.2	target 5' CAGTTTTCGGCGAAACCCCG 3'<br>:: . :.:::::::::::::<br>miRNA 3' GTGGCAGGCCGCTTTGGGGC 5'	3	24-43	Uncharacterized protein
grape-m1458	VIT_216s0100g00490.3	target 5' CAGTTTTCGGCGAAACCCCG 3'<br>:: . :.:::::::::::::<br>miRNA 3' GTGGCAGGCCGCTTTGGGGC 5'	3	24-43	Uncharacterized protein
grape-m1458	VIT_216s0100g00490.4	target 5' CAGTTTTCGGCGAAACCCCG 3'<br>:: . :.:::::::::::::<br>miRNA 3' GTGGCAGGCCGCTTTGGGGC 5'	3	24-43	Uncharacterized protein
grape-m1458	VIT_216s0100g00490.5	target 5' CAGTTTTCGGCGAAACCCCG 3'<br>:: . :.:::::::::::::<br>miRNA 3' GTGGCAGGCCGCTTTGGGGC 5'	3	24-43	Uncharacterized protein
grape-m1458	VIT_216s0100g00490.6	target 5' CAGTTTTCGGCGAAACCCCG 3'<br>:: . :.:::::::::::::<br>miRNA 3' GTGGCAGGCCGCTTTGGGGC 5'	3	24-43	Uncharacterized protein
grape-m1458	VIT_216s0100g00490.7	target 5' CAGTTTTCGGCGAAACCCCG 3'<br>:: . :.:::::::::::::<br>miRNA 3' GTGGCAGGCCGCTTTGGGGC 5'	3	24-43	Uncharacterized protein
grape-m1458	VIT_216s0100g00490.8	target 5' CAGTTTTCGGCGAAACCCCG 3'<br>:: . :.:::::::::::::<br>miRNA 3' GTGGCAGGCCGCTTTGGGGC 5'	3	24-43	Uncharacterized protein
grape-m1458	VIT_216s0100g00490.9	target 5' CAGTTTTCGGCGAAACCCCG 3'<br>:: . :.:::::::::::::<br>miRNA 3' GTGGCAGGCCGCTTTGGGGC 5'	3	24-43	Uncharacterized protein
grape-m2136	VIT_213s0067g01040.1	target 5' AGTCAATCCTCGACCTTGGTA 3'<br>:::::::::::::::::::::<br>miRNA 3' TCAGTTAGGAGCTGGAACCAT 5'	1	179-199	Hypothetical protein
grape-m2483	VIT_201s0011g02270.1	target 5' TTCAACATCTGCATCCTCATCA 3'<br>: ::::: ::.:::::::::::<br>miRNA 3' ACGTTGTCGATGTAGGAGTAGT 5'	3	1521-1542	Serine threonine-protein kinase sepa-like

miRNA	Target id	Alignment	Score	Range	Target gene functional annotation
grape-m2483	VIT_201s0011g02270.2	target 5' TTCAACATCTGCATCCTCATCA 3'<br>: ::::: ::.:::::::::::<br>miRNA 3' ACGTTGTCGATGTAGGAGTAGT 5'	3	945-966	Serine threonine-protein kinase sepa-like
grape-m2483	VIT_211s0016g00160.1	target 5' TGCCACAGCTACCTCCTCATCA 3'<br>::: :::::::: :::::::::<br>miRNA 3' ACGTTGTCGATGTAGGAGTAGT 5'	3	1793-1814	Formin 2b
grape-m2616	VIT_206s0061g00330.1	target 5' GAAGTAGACCTTCTGAAGCAA 3'<br>::::::.::::::.:::::::<br>miRNA 3' CTTCATTTGGAAGGCTTCGTT 5'	1.5	176-196	Uncharacterized protein
grape-m2616	VIT_206s0061g00330.2	target 5' GAAGTAGACCTTCTGAAGCAA 3'<br>::::::.::::::.:::::::<br>miRNA 3' CTTCATTTGGAAGGCTTCGTT 5'	1.5	269-289	Uncharacterized protein loc100258439
grape-m2616	VIT_206s0061g00330.3	target 5' GAAGTAGACCTTCTGAAGCAA 3'<br>::::::.::::::.:::::::<br>miRNA 3' CTTCATTTGGAAGGCTTCGTT 5'	1.5	269-289	Uncharacterized protein
grape-m3245-3p	VIT_218s0072g01090.1	target 5' GGAATAGGAGGGGTAGGAAAGA 3'<br>::::: :::::.::::::::::<br>miRNA 3' CCTTACCCTCCTCATCCTTTCT 5'	1	154-175	Hypothetical protein
grape-m3245-5p	VIT_211s0016g05880.1	target 5' GAGCAGTTCTACTCTCCAATT 3'<br>.::: :.:::::::::::::<br>miRNA 3' TTCGAAAGGATGAGAGGTTAA 5'	3	1728-1748	Cgs1 mrna stability
grape-m3453	VIT_213s0019g02810.1	target 5' CCCTCTTCACCTTCCATTCA 3'<br>::::::: :::::::::::<br>miRNA 3' AGGAGAAGCGGAAGGTAAGT 5'	3	179-198	Udp-glycosyltransferase 76g1
grape-m3453	VIT_213s0019g02860.1	target 5' CCCTCTTCACCTTCCATTCA 3'<br>::::::: :::::::::::<br>miRNA 3' AGGAGAAGCGGAAGGTAAGT 5'	3	1502-1521	Udp-glycosyltransferase-like protein
grape-m3778	VIT_205s0029g00750.1	target 5' CCATCTATTGGAGATCTCAAG 3'<br>:::::.::::::: :::::::<br>miRNA 3' GGTAGGTAACCTCGAGAGTTC 5'	2.5	1474-1494	Tmv resistance protein n-like
grape-m3778	VIT_205s0029g00750.2	target 5' CCATCTATTGGAGATCTCAAG 3'<br>:::::.::::::: :::::::<br>miRNA 3' GGTAGGTAACCTCGAGAGTTC 5'	2.5	1474-1494	Tmv resistance protein n-like
grape-m3778	VIT_205s0029g00750.3	target 5' CCATCTATTGGAGATCTCAAG 3'<br>:::::.::::::: :::::::<br>miRNA 3' GGTAGGTAACCTCGAGAGTTC 5'	2.5	436-456	Tmv resistance protein n-like
grape-m3778	VIT_205s0029g00870.1	target 5' CCATCTATTGGAGATCTCAAG 3'<br>:::::.::::::: :::::::<br>miRNA 3' GGTAGGTAACCTCGAGAGTTC 5'	2.5	2032-2052	Tmv resistance protein n-like
grape-m3778	VIT_212s0034g00280.1	target 5' CCATCTGTTGGAGTTCTCAAG 3'<br>:::::..::::::.:::::::<br>miRNA 3' GGTAGGTAACCTCGAGAGTTC 5'	2	631-651	Tmv resistance protein n-like

miRNA	Target id	Alignment	Score	Range	Target gene functional annotation
grape-m3778	VIT_212s0034g00850.1	target 5' CCATCTGTTGGAGATCTCAAG 3'<br>:::::..:::::: :::::::<br>miRNA 3' GGTAGGTAACCTCGAGAGTTC 5'	3	2146-2166	Tmv resistance protein n-like
grape-m3778	VIT_212s0142g00590.1	target 5' CCATCTCTTGGAGTTCTCAAG 3'<br>:::::. ::::::.:::::::<br>miRNA 3' GGTAGGTAACCTCGAGAGTTC 5'	2.5	1672-1692	Tmv resistance protein n-like
grape-m3778	VIT_215s0045g00440.1	target 5' CCATCCATTGGAGCTCTCCAG 3'<br>:::::::::::::::::: ::<br>miRNA 3' GGTAGGTAACCTCGAGAGTTC 5'	2	802-822	Tmv resistance protein n-like
grape-m3778	VIT_218s0041g00200.1	target 5' CCATCTATTGGAGCTCTCAAG 3'<br>:::::.:::::::::::::::<br>miRNA 3' GGTAGGTAACCTCGAGAGTTC 5'	0.5	1561-1581	Tmv resistance protein n-like
grape-m3778	VIT_218s0041g00250.1	target 5' CCATCTATTGGAGCTCTCAAG 3'<br>:::::.:::::::::::::::<br>miRNA 3' GGTAGGTAACCTCGAGAGTTC 5'	0.5	1630-1650	Tmv resistance protein n-like
grape-m3816	VIT_212s0034g00280.1	target 5' CTTTAGATTTTTTACCATCAA 3'<br>::::::: :.:.:::::::::<br>miRNA 3' GAAATCTTAGAGATGGTAGTT 5'	3	407-427	Tmv resistance protein n-like
grape-m3816	VIT_212s0034g00910.1	target 5' CTTTAGATTTTTTACCATCAA 3'<br>::::::: :.:.:::::::::<br>miRNA 3' GAAATCTTAGAGATGGTAGTT 5'	3	1865-1885	Tmv resistance protein n-like
grape-m3816	VIT_218s0001g05050.1	target 5' CTTTAGAATCTTTACCAACAA 3'<br>:::::::::::.::::: :::<br>miRNA 3' GAAATCTTAGAGATGGTAGTT 5'	3	1760-1780	Tmv resistance protein n-like
grape-m3816	VIT_218s0072g01230.1	target 5' CATTAGAATCTTTGCCATCAA 3'<br>: :::::::::.:.:::::::<br>miRNA 3' GAAATCTTAGAGATGGTAGTT 5'	3	1781-1801	Tmv resistance protein n-like
grape-m3816	VIT_218s0089g00060.1	target 5' CTTTAGAATCTCTACCATCAA 3'<br>:::::::::::::::::::::<br>miRNA 3' GAAATCTTAGAGATGGTAGTT 5'	0	1847-1867	Tmv resistance protein n-like
grape-m3816	VIT_218s0089g00060.2	target 5' CTTTAGAATCTCTACCATCAA 3'<br>:::::::::::::::::::::<br>miRNA 3' GAAATCTTAGAGATGGTAGTT 5'	0	1847-1867	Tmv resistance protein n-like
grape-m3816	VIT_218s0089g00060.3	target 5' CTTTAGAATCTCTACCATCAA 3'<br>:::::::::::::::::::::<br>miRNA 3' GAAATCTTAGAGATGGTAGTT 5'	0	1847-1867	Tmv resistance protein n-like
grape-m3816	VIT_218s0089g00090.1	target 5' CTTTAGAATCTCTACCATCAA 3'<br>:::::::::::::::::::::<br>miRNA 3' GAAATCTTAGAGATGGTAGTT 5'	0	1772-1792	Tmv resistance protein n-like
grape-m3816	VIT_218s0089g00090.2	target 5' CTTTAGAATCTCTACCATCAA 3'<br>:::::::::::::::::::::<br>miRNA 3' GAAATCTTAGAGATGGTAGTT 5'	0	1772-1792	Tmv resistance protein n-like

miRNA	Target id	Alignment	Score	Range	Target gene functional annotation
grape-m3816	VIT_218s0089g00090.4	target 5' CTTTAGAATCTCTACCATCAA 3'<br>:::::::::::::::::::::<br>miRNA 3' GAAATCTTAGAGATGGTAGTT 5'	0	1772-1792	Tmv resistance protein n-like
grape-m3816	VIT_218s0089g00090.5	target 5' CTTTAGAATCTCTACCATCAA 3'<br>:::::::::::::::::::::<br>miRNA 3' GAAATCTTAGAGATGGTAGTT 5'	0	1772-1792	Tmv resistance protein n-like
grape-m3816	VIT_218s0089g00100.1	target 5' CTTTAGAATCTCTACCACCAA 3'<br>::::::::::::::::: :::<br>miRNA 3' GAAATCTTAGAGATGGTAGTT 5'	2	1250-1270	Tmv resistance protein n-like
grape-m4051	VIT_204s0069g00560.1	target 5' TATGAAAATAGAGATATCTTA 3'<br>.::: :::::::::::::::<br>miRNA 3' GTACGGTTATCTCTATAGAAT 5'	2.5	2476-2496	Glutamate receptor -like
grape-m4250	VIT_211s0118g00150.1	target 5' TCTTC-CATTTCTCATACCCA 3'<br>::::: :::::::::.::::<br>miRNA 3' AGAAGTGTAAAGAGTGTGGGA 5'	1	37-56	Hypothetical protein
grape-m4269	VIT_204s0008g02560.1	target 5' CCTTGAAGCAATTTTCAG-CA 3'<br>:::::::::::::.:::: ::<br>miRNA 3' GGAACTTCGTTAAGAGTCTGT 5'	3	603-622	Uncharacterized aarf domain-containing protein kinase chloroplastic-like
grape-m4269	VIT_204s0008g02560.2	target 5' CCTTGAAGCAATTTTCAG-CA 3'<br>:::::::::::::.:::: ::<br>miRNA 3' GGAACTTCGTTAAGAGTCTGT 5'	3	237-256	Uncharacterized aarf domain-containing protein kinase chloroplastic-like
grape-m4277	VIT_211s0118g00150.1	target 5' TCTTC-CATTTCTCATACCCA 3'<br>::::: :::::::::.::::<br>miRNA 3' AGAAGTGTAAAGAGTGTGGGA 5'	1	37-56	Hypothetical protein
grape-m4553-5p	VIT_201s0010g02270.1	target 5' ATATTGGAAGCCTTTGAGGGA 3'<br>: :::::::::::::::::::<br>miRNA 3' TTTAACCTTCGGAAACTCCCT 5'	1	778-798	Gras family transcription factor
grape-m4553-5p	VIT_217s0000g10300.1	target 5' ATATTGGAAGCCTTTGAGGGA 3'<br>: :::::::::::::::::::<br>miRNA 3' TTTAACCTTCGGAAACTCCCT 5'	1	748-768	Gras family transcription factor
grape-m4560	VIT_203s0091g00850.1	target 5' TTTTTCAGAATCACTGCCAAG 3'<br>:::.. ::::::::::::::.<br>miRNA 3' AAAGGATCTTAGTGACGGTTT 5'	2.5	238-258	Protein
grape-m4560	VIT_203s0091g00850.2	target 5' TTTTTCAGAATCACTGCCAAG 3'<br>:::.. ::::::::::::::.<br>miRNA 3' AAAGGATCTTAGTGACGGTTT 5'	2.5	238-258	Protein
grape-m4560	VIT_214s0068g00250.1	target 5' TCTCC-AAAATCACTGCCAAA 3'<br>: ::: : :::::::::::::<br>miRNA 3' AAAGGATCTTAGTGACGGTTT 5'	3	1924-1943	Exocyst complex component 7
grape-m4560	VIT_214s0068g00250.2	target 5' TCTCC-AAAATCACTGCCAAA 3'<br>: ::: : :::::::::::::<br>miRNA 3' AAAGGATCTTAGTGACGGTTT 5'	3	1924-1943	Exocyst complex component 7

miRNA	Target id	Alignment	Score	Range	Target gene functional annotation
grape-m4846	VIT_213s0067g01490.1	target 5' GGTGGAATTTCAAATTGATGG 3'<br>:.::.:::::::::..:::::<br>miRNA 3' CTACTTTAAAGTTTGGCTACC 5'	3	1176-1196	Disease resistance protein at3g14460-like
grape-m4912	VIT_208s0007g00660.1	target 5' CAGAGAGCCACCCACTTCATC 3'<br>::: :: :::::::::::::<br>miRNA 3' CTCTGTC-GTGGGTGAAGTAG 5'	3	419-439	Laccase 110c
grape-m4912	VIT_218s0072g00510.1	target 5' GAAACTGACACCCACTTCATC 3'<br>:: :: : :::::::::::::<br>miRNA 3' CTCTGTC-GTGGGTGAAGTAG 5'	3	43-63	Kda class i heat shock protein
grape-m5176	VIT_205s0029g00780.1	target 5' CGGCCTTCCTTTAGCTCTAAA 3'<br>::::::::::::::::: ::<br>miRNA 3' TCCGGAAGGAAATCGAGAGTT 5'	3	741-761	Tmv resistance protein n-like
grape-m5176	VIT_205s0029g00870.1	target 5' TGGCCTTCCTTTAGCTCTAAA 3'<br>::::::::::::::::: ::<br>miRNA 3' TCCGGAAGGAAATCGAGAGTT 5'	3	1167-1187	Tmv resistance protein n-like
grape-m5176	VIT_208s0056g00770.1	target 5' AGTCCTTCCTTTAGCTCTCAA 3'<br>:: ::::::::::::::::::<br>miRNA 3' TCCGGAAGGAAATCGAGAGTT 5'	1	381-401	Regulation of nuclear pre-mrna domain-containing protein 1b
grape-m5176	VIT_208s0056g00770.2	target 5' AGTCCTTCCTTTAGCTCTCAA 3'<br>:: ::::::::::::::::::<br>miRNA 3' TCCGGAAGGAAATCGAGAGTT 5'	1	255-275	Regulation of nuclear pre-mrna domain-containing protein 1b
grape-m5176	VIT_208s0056g00770.3	target 5' AGTCCTTCCTTTAGCTCTCAA 3'<br>:: ::::::::::::::::::<br>miRNA 3' TCCGGAAGGAAATCGAGAGTT 5'	1	255-275	Regulation of nuclear pre-mrna domain-containing protein 1b
grape-m5176	VIT_208s0056g00770.4	target 5' AGTCCTTCCTTTAGCTCTCAA 3'<br>:: ::::::::::::::::::<br>miRNA 3' TCCGGAAGGAAATCGAGAGTT 5'	1	255-275	Regulation of nuclear pre-mrna domain-containing protein 1b
grape-m5176	VIT_208s0056g00770.5	target 5' AGTCCTTCCTTTAGCTCTCAA 3'<br>:: ::::::::::::::::::<br>miRNA 3' TCCGGAAGGAAATCGAGAGTT 5'	1	255-275	Regulation of nuclear pre-mrna domain-containing protein 1b
grape-m5176	VIT_214s0128g00050.1	target 5' AGCTCTATCTTTAGCTCTCAA 3'<br>:: .:: .:::::::::::::<br>miRNA 3' TCCGGAAGGAAATCGAGAGTT 5'	3	225-245	Thylakoid lumen kda protein
grape-m5176	VIT_214s0128g00050.2	target 5' AGCTCTATCTTTAGCTCTCAA 3'<br>:: .:: .:::::::::::::<br>miRNA 3' TCCGGAAGGAAATCGAGAGTT 5'	3	225-245	Thylakoid lumen kda protein
grape-m5176	VIT_214s0128g00050.3	target 5' AGCTCTATCTTTAGCTCTCAA 3'<br>:: .:: .:::::::::::::<br>miRNA 3' TCCGGAAGGAAATCGAGAGTT 5'	3	225-245	Protein
grape-m5176	VIT_214s0128g00050.4	target 5' AGCTCTATCTTTAGCTCTCAA 3'<br>:: .:: .:::::::::::::<br>miRNA 3' TCCGGAAGGAAATCGAGAGTT 5'	3	225-245	Thylakoid lumen kda protein

miRNA	Target id	Alignment	Score	Range	Target gene functional annotation
grape-m5176	VIT_218s0001g03900.1	target 5' GGGTCATCCTTTAGCTCTTAA 3'<br>.::.: ::::::::::::.::<br>miRNA 3' TCCGGAAGGAAATCGAGAGTT 5'	3	1104-1124	Tmv resistance protein n-like
grape-m5176	VIT_218s0001g10420.1	target 5' ATTCCTTCTCTTTAGCTCTCAA 3'<br>: ::::: :::::::::::::<br>miRNA 3' TCCGGAAG-GAAATCGAGAGTT 5'	3	236-257	Dna binding
grape-m5176	VIT_218s0001g11250.1	target 5' AGGGCTTCCTTTGGCTCTTAA 3'<br>::: ::::::::.:::::.::<br>miRNA 3' TCCGGAAGGAAATCGAGAGTT 5'	3	1101-1121	Nbs-lrr resistance protein
grape-m5380	VIT_200s1313g00010.1	target 5' CCATCCACAACATAATCTCC 3'<br>::::::::::::::::::::<br>miRNA 3' GGTAGGTGTTGTATTAGAGG 5'	0	240-259	1-aminocyclopropane-1-carboxylate oxidase homolog 1
grape-m5380	VIT_200s1313g00010.2	target 5' CCATCCACAACATAATCTCC 3'<br>::::::::::::::::::::<br>miRNA 3' GGTAGGTGTTGTATTAGAGG 5'	0	240-259	1-aminocyclopropane-1-carboxylate oxidase homolog 1
grape-m5380	VIT_205s0049g00330.1	target 5' CCATCCACAACATAATCTCC 3'<br>::::::::::::::::::::<br>miRNA 3' GGTAGGTGTTGTATTAGAGG 5'	0	111-130	1-aminocyclopropane-1-carboxylate oxidase homolog 1
grape-m5380	VIT_205s0049g00340.1	target 5' CCATCCACAACATAATCTCC 3'<br>::::::::::::::::::::<br>miRNA 3' GGTAGGTGTTGTATTAGAGG 5'	0	138-157	1-aminocyclopropane-1-carboxylate oxidase homolog 1
grape-m5380	VIT_205s0049g00340.2	target 5' CCATCCACAACATAATCTCC 3'<br>::::::::::::::::::::<br>miRNA 3' GGTAGGTGTTGTATTAGAGG 5'	0	138-157	1-aminocyclopropane-1-carboxylate oxidase homolog 1
grape-m5380	VIT_205s0049g00350.1	target 5' CCATCCACAGCATTATCTCC 3'<br>:::::::::.::: ::::::<br>miRNA 3' GGTAGGTGTTGTATTAGAGG 5'	3	132-151	1-aminocyclopropane-1-carboxylate oxidase homolog 1
grape-m5380	VIT_205s0049g00350.2	target 5' CCATCCACAGCATTATCTCC 3'<br>:::::::::.::: ::::::<br>miRNA 3' GGTAGGTGTTGTATTAGAGG 5'	3	132-151	1-aminocyclopropane-1-carboxylate oxidase homolog 1
grape-m5380	VIT_205s0049g00370.1	target 5' CCATCCACAACATAATCTCT 3'<br>:::::::::::::::::::.<br>miRNA 3' GGTAGGTGTTGTATTAGAGG 5'	0.5	132-151	1-aminocyclopropane-1-carboxylate oxidase homolog 1
grape-m5380	VIT_216s0022g02420.1	target 5' CCA-CTACAACATAATCTCT 3'<br>::: :.:::::::::::::.<br>miRNA 3' GGTAGGTGTTGTATTAGAGG 5'	2	1470-1488	Protein
grape-m5380	VIT_216s0022g02450.1	target 5' CCA-ATACAACATAATCTCC 3'<br>::: .::::::::::::::<br>miRNA 3' GGTAGGTGTTGTATTAGAGG 5'	2.5	2439-2457	Protein
grape-m5380	VIT_216s0100g00030.1	target 5' CCA-ATACAACATAATCTCC 3'<br>::: .::::::::::::::<br>miRNA 3' GGTAGGTGTTGTATTAGAGG 5'	2.5	1245-1263	Cucumisin-like

miRNA	Target id	Alignment	Score	Range	Target gene functional annotation
grape-m6251	VIT_211s0016g01760.1	target 5' TCGTACCAAAAAACCTAAAAT 3'<br>: . :::::::::::::::::<br>miRNA 3' ATTTTGGTTTTTTGGATTTTA 5'	2.5	743-763	Pre-mrna cleavage factor 25kd
grape-m6251	VIT_211s0016g01760.2	target 5' TCGTACCAAAAAACCTAAAAT 3'<br>: . :::::::::::::::::<br>miRNA 3' ATTTTGGTTTTTTGGATTTTA 5'	2.5	743-763	Pre-mrna cleavage factor 25kd
grape-m6580	VIT_201s0127g00500.1	target 5' AACA-AACATCTTATGAACA 3'<br>::: ::::::::::::::<br>miRNA 3' GTGTAGTGTAGAATACTTGT 5'	3	869-887	Uncharacterized protein
grape-m6580	VIT_201s0127g00500.2	target 5' AACA-AACATCTTATGAACA 3'<br>::: ::::::::::::::<br>miRNA 3' GTGTAGTGTAGAATACTTGT 5'	3	809-827	Uncharacterized protein
grape-m6580	VIT_205s0102g00460.1	target 5' TATATT-CATCTTATGAACA 3'<br>.:.::. :::::::::::::<br>miRNA 3' GTGTAGTGTAGAATACTTGT 5'	2.5	1066-1084	Ubiquitin carboxyl-terminal hydrolase-like protein
grape-m6580	VIT_205s0102g00460.2	target 5' TATATT-CATCTTATGAACA 3'<br>.:.::. :::::::::::::<br>miRNA 3' GTGTAGTGTAGAATACTTGT 5'	2.5	1066-1084	Ubiquitin carboxyl-terminal hydrolase-like protein
grape-m6580	VIT_209s0002g04070.1	target 5' CACAGAACATTTTATGAACA 3'<br>:::: ::::.:::::::::<br>miRNA 3' GTGTAGTGTAGAATACTTGT 5'	3	1041-1060	Uncharacterized protein
grape-m6580	VIT_216s0013g00774.1	target 5' TATATT-CATCTTATGAACA 3'<br>.:.::. :::::::::::::<br>miRNA 3' GTGTAGTGTAGAATACTTGT 5'	2.5	487-505	Ubiquitin carboxyl-terminal hydrolase-like protein
grape-m6905	VIT_203s0110g00350.1	target 5' AAGCTCCTGCTCCAACGCGGC 3'<br>:::::::::::::::::::::<br>miRNA 3' TTCGAGGACGAGGTTGCGCCG 5'	0	67-87	Cinnamoyl reductase-like protein
grape-m6905	VIT_213s0047g00540.1	target 5' AAGCTCCTTCTCCAACGCGGC 3'<br>:::::::: ::::::::::::<br>miRNA 3' TTCGAGGACGAGGTTGCGCCG 5'	2	67-87	Low quality protein: bifunctional dihydroflavonol 4-reductase flavanone 4-reductase-like
grape-m6905	VIT_213s0047g00940.1	target 5' AAGCTCCTGCTCCAACGCGGC 3'<br>:::::::::::::::::::::<br>miRNA 3' TTCGAGGACGAGGTTGCGCCG 5'	0	67-87	Low quality protein: bifunctional dihydroflavonol 4-reductase flavanone 4-reductase-like
grape-m6905	VIT_213s0047g00990.1	target 5' AAGCTCTTGCTCCAACGCGGC 3'<br>::::::.::::::::::::::<br>miRNA 3' TTCGAGGACGAGGTTGCGCCG 5'	0.5	67-87	Low quality protein: bifunctional dihydroflavonol 4-reductase flavanone 4-reductase-like
grape-m6905	VIT_213s0064g00290.1	target 5' AAGCTCCTGCTCCAACGCGGC 3'<br>:::::::::::::::::::::<br>miRNA 3' TTCGAGGACGAGGTTGCGCCG 5'	0	67-87	Low quality protein: bifunctional dihydroflavonol 4-reductase flavanone 4-reductase-like
grape-m6905	VIT_213s0064g00340.1	target 5' AAGCTCCTGCTCCAACGTGGC 3'<br>:::::::::::::::::.:::<br>miRNA 3' TTCGAGGACGAGGTTGCGCCG 5'	1	Nov-31	Phenylacetaldehyde reductase

miRNA	Target id	Alignment	Score	Range	Target gene functional annotation
grape-m6905	VIT_213s0067g00460.1	target 5' AAGCTCCTGCTCCAACGTGGT 3'<br>:::::::::::::::::.::.<br>miRNA 3' TTCGAGGACGAGGTTGCGCCG 5'	1.5	70-90	Low quality protein: bifunctional dihydroflavonol 4-reductase flavanone 4-reductase-like
grape-m6905	VIT_213s0067g00530.1	target 5' AAGCTCCTGCTCCAACGTGGT 3'<br>:::::::::::::::::.::.<br>miRNA 3' TTCGAGGACGAGGTTGCGCCG 5'	1.5	70-90	Low quality protein: bifunctional dihydroflavonol 4-reductase flavanone 4-reductase-like
grape-m7192	VIT_219s0090g01460.1	target 5' CAG-ATCCATCTCTTCTTCC 3'<br>:: :: :::::::::::::<br>miRNA 3' CTCGTACGTAGAGAAGAAGG 5'	3	151-169	Brg-1 associated
grape-m7484	VIT_205s0102g00030.1	target 5' AGATGGCACTTAACAGCCAA 3'<br>:: ..::::::::::::::<br>miRNA 3' TCACTTGTGAATTGTCGGTT 5'	3	1148-1167	Ankyrin repeat-containing protein at3g12360-like
grape-m7484	VIT_205s0165g00290.1	target 5' AGATGATACTTAACAGCCAA 3'<br>:: .:.:::::::::::::<br>miRNA 3' TCACTTGTGAATTGTCGGTT 5'	3	1172-1191	Ankyrin repeat-containing protein at3g12360-like
grape-m7644	VIT_203s0110g00030.1	target 5' AATGACACAGTCCTCCATATG 3'<br>:: .:::::::::::::::::<br>miRNA 3' TTCTTGTGTCAGGAGGTATAA 5'	2.5	139-159	Ankyrin repeat-containing protein at3g12360-like
grape-m7644	VIT_205s0124g00345.1	target 5' AAGAACACAGTCCTCCATATT 3'<br>:::::::::::::::::::::<br>miRNA 3' TTCTTGTGTCAGGAGGTATAA 5'	0	385-405	Ankyrin repeat-containing protein at3g12360-like
grape-m7644	VIT_205s0124g00697.1	target 5' GAGAACACAGTCCTCCACATT 3'<br>.:::::::::::::::: :::<br>miRNA 3' TTCTTGTGTCAGGAGGTATAA 5'	2.5	208-228	Ankyrin repeat-containing protein at3g12360-like
grape-m7644	VIT_205s0124g00720.1	target 5' AAGAACACAGTTCTCCATGTT 3'<br>:::::::::::.::::::.::<br>miRNA 3' TTCTTGTGTCAGGAGGTATAA 5'	2	220-240	Ankyrin repeat-containing protein at3g12360-like
grape-m7644	VIT_205s0124g00720.2	target 5' AAGAACACAGTTCTCCATGTT 3'<br>:::::::::::.::::::.::<br>miRNA 3' TTCTTGTGTCAGGAGGTATAA 5'	2	220-240	Ankyrin repeat-containing protein at3g12360-like
grape-m7644	VIT_205s0165g00080.1	target 5' CAGAACACTGTCCTCCATATT 3'<br>::::::: ::::::::::::<br>miRNA 3' TTCTTGTGTCAGGAGGTATAA 5'	3	220-240	Ankyrin repeat-containing protein at3g12360-like
grape-m7644	VIT_219s0014g05330.1	target 5' AAGAACACAGCCCTTCATATT 3'<br>:::::::::: :::.::::::<br>miRNA 3' TTCTTGTGTCAGGAGGTATAA 5'	3	133-153	Ankyrin repeat-containing
grape-m7644	VIT_219s0085g00470.1	target 5' AAGAACACAGTCCTCCATATT 3'<br>:::::::::::::::::::::<br>miRNA 3' TTCTTGTGTCAGGAGGTATAA 5'	0	179-199	Uncharacterized protein loc100854011
grape-m7774	VIT_200s1313g00010.1	target 5' CCATCCACAACATAATCTCC 3'<br>::::::::::::::::::::<br>miRNA 3' GGTAGGTGTTGTATTAGAGG 5'	0	240-259	1-aminocyclopropane-1-carboxylate oxidase homolog 1

miRNA	Target id	Alignment	Score	Range	Target gene functional annotation
grape-m7774	VIT_200s1313g00010.2	target 5' CCATCCACAACATAATCTCC 3'<br>::::::::::::::::::::<br>miRNA 3' GGTAGGTGTTGTATTAGAGG 5'	0	240-259	1-aminocyclopropane-1-carboxylate oxidase homolog 1
grape-m7774	VIT_205s0049g00330.1	target 5' CCATCCACAACATAATCTCC 3'<br>::::::::::::::::::::<br>miRNA 3' GGTAGGTGTTGTATTAGAGG 5'	0	111-130	1-aminocyclopropane-1-carboxylate oxidase homolog 1
grape-m7774	VIT_205s0049g00340.1	target 5' CCATCCACAACATAATCTCC 3'<br>::::::::::::::::::::<br>miRNA 3' GGTAGGTGTTGTATTAGAGG 5'	0	138-157	1-aminocyclopropane-1-carboxylate oxidase homolog 1
grape-m7774	VIT_205s0049g00340.2	target 5' CCATCCACAACATAATCTCC 3'<br>::::::::::::::::::::<br>miRNA 3' GGTAGGTGTTGTATTAGAGG 5'	0	138-157	1-aminocyclopropane-1-carboxylate oxidase homolog 1
grape-m7774	VIT_205s0049g00350.1	target 5' CCATCCACAGCATTATCTCC 3'<br>:::::::::.::: ::::::<br>miRNA 3' GGTAGGTGTTGTATTAGAGG 5'	3	132-151	1-aminocyclopropane-1-carboxylate oxidase homolog 1
grape-m7774	VIT_205s0049g00350.2	target 5' CCATCCACAGCATTATCTCC 3'<br>:::::::::.::: ::::::<br>miRNA 3' GGTAGGTGTTGTATTAGAGG 5'	3	132-151	1-aminocyclopropane-1-carboxylate oxidase homolog 1
grape-m7774	VIT_205s0049g00370.1	target 5' CCATCCACAACATAATCTCT 3'<br>:::::::::::::::::::.<br>miRNA 3' GGTAGGTGTTGTATTAGAGG 5'	0.5	132-151	1-aminocyclopropane-1-carboxylate oxidase homolog 1
grape-m7774	VIT_216s0022g02420.1	target 5' CCA-CTACAACATAATCTCT 3'<br>::: :.:::::::::::::.<br>miRNA 3' GGTAGGTGTTGTATTAGAGG 5'	2	1470-1488	Protein
grape-m7774	VIT_216s0022g02450.1	target 5' CCA-ATACAACATAATCTCC 3'<br>::: .::::::::::::::<br>miRNA 3' GGTAGGTGTTGTATTAGAGG 5'	2.5	2439-2457	Protein
grape-m7774	VIT_216s0100g00030.1	target 5' CCA-ATACAACATAATCTCC 3'<br>::: .::::::::::::::<br>miRNA 3' GGTAGGTGTTGTATTAGAGG 5'	2.5	1245-1263	Cucumisin-like
grape-m7969	VIT_212s0034g01000.1	target 5' GATCTATGTTTTCTCTTTTGA 3'<br>:::.:..:::::::::::::<br>miRNA 3' GTAGGTGTAAAAGAGAAAACT 5'	2.5	117-137	Mrna turnover protein 4
grape-m8278-5p	VIT_201s0010g02270.1	target 5' ATATTGGAAGCCTTTGAGGGA 3'<br>: :::::::::::::::::::<br>miRNA 3' TTTAACCTTCGGAAACTCCCT 5'	1	778-798	Gras family transcription factor
grape-m8278-5p	VIT_217s0000g10300.1	target 5' ATATTGGAAGCCTTTGAGGGA 3'<br>: :::::::::::::::::::<br>miRNA 3' TTTAACCTTCGGAAACTCCCT 5'	1	748-768	Gras family transcription factor
grape-m8937-5p	VIT_207s0005g00700.1	target 5' TCCTCTCCCTCTCCTCCCTCGG 3'<br>::::::: :::: :::::::::<br>miRNA 3' AGGAGAGCGAGA-GAGGGAGCC 5'	3	131-152	Epoxide hydrolase 2-like

miRNA	Target id	Alignment	Score	Range	Target gene functional annotation
grape-m8937-5p	VIT_209s0002g04913.1	target 5' TCCTCT-GCTCTTTCCCTCGC 3'<br>:::::: :::::.::::::::<br>miRNA 3' AGGAGAGCGAGAGAGGGAGCC 5'	3	2325-2344	Disease resistance protein rps5-like
grape-m8937-5p	VIT_209s0002g05000.2	target 5' TCCTCT-GCTCTTTCCCTCGC 3'<br>:::::: :::::.::::::::<br>miRNA 3' AGGAGAGCGAGAGAGGGAGCC 5'	3	2481-2500	Disease resistance protein rps5-like
grape-m8937-5p	VIT_209s0002g05070.1	target 5' TCCTCT-GCTCTTTCCCTCGC 3'<br>:::::: :::::.::::::::<br>miRNA 3' AGGAGAGCGAGAGAGGGAGCC 5'	3	2484-2503	Disease resistance protein rps5-like
grape-m8937-5p	VIT_218s0001g02870.1	target 5' TCCTCTCTCTCTCCCTCTG 3'<br>::::::: ::::::::::: :<br>miRNA 3' AGGAGAGCGAGAGAGGGAGCC 5'	3	92-112	Unnamed protein product [Vitis vinifera]
grape-m8937-5p	VIT_219s0085g00680.1	target 5' TCCTC-CTCTCTCTCTCTCGG 3'<br>::::: : :::::::.:::::<br>miRNA 3' AGGAGAGCGAGAGAGGGAGCC 5'	3	56-75	Uncharacterized protein
grape-m8943	VIT_210s0003g03070.1	target 5' CGACTCCCAAGGTTACATTGA 3'<br>:. ::::::::::::::::::<br>miRNA 3' GTCGAGGGTTCCAATGTAACT 5'	1.5	312-332	Beta-ketoacyl-acp synthase i
grape-m8960	VIT_214s0030g00650.1	target 5' AGTCAAACAATCTCC-TTTG 3'<br>::::::::::::::: :::.<br>miRNA 3' TCAGTTTGTTAGAGGCAAAT 5'	2.5	216-234	Bifunctional 3-dehydroquinate dehydratase shikimate chloroplastic-like
grape-m8960	VIT_214s0030g00650.2	target 5' AGTCAAACAATCTCC-TTTG 3'<br>::::::::::::::: :::.<br>miRNA 3' TCAGTTTGTTAGAGGCAAAT 5'	2.5	216-234	Bifunctional 3-dehydroquinate dehydratase shikimate chloroplastic-like
grape-m8993	VIT_208s0007g08500.1	target 5' GGAGCTGTCGCAGCCACTACGA 3'<br>::::::::::::::::::::::<br>miRNA 3' CCTCGACAGCGTCGGTGATGCT 5'	0	788-809	60s ribosomal export protein nmd3-like
grape-m9163	VIT_200s2579g00010.1	target 5' GCGTTAATGTCTTCAATCTCA 3'<br>.:.:.:: :::::::::::::<br>miRNA 3' TGTAGTTTCAGAAGTTAGAGT 5'	2.5	92-112	Phosphoserine aminotransferase
grape-m9163	VIT_206s0004g04000.1	target 5' ACATCAAAGCCTTCAATCTCA 3'<br>::::::::: :::::::::::<br>miRNA 3' TGTAGTTTCAGAAGTTAGAGT 5'	2	470-490	3-ketoacyl- synthase
grape-m9269	VIT_208s0007g05240.1	target 5' GTGGAGAAGACGGTGAGAAGAA 3'<br>:. ::::: :::::::::::::<br>miRNA 3' CGACTCTT-TGCCACTCTTCTT 5'	2.5	1762-1783	Phosphatidylinositol-4-phosphate 5-kinase
vvi-miR156a	VIT_201s0010g03710.1	target 5' GTGCTCTCTCTCTTCTGTCAA 3'<br>::::::.::::::::::::::<br>miRNA 3' CACGAGGGAGAGAAGACAGTT 5'	0.5	1145-1165	Squamosa promoter-binding-like protein 2
vvi-miR156a	VIT_201s0010g03710.2	target 5' GTGCTCTCTCTCTTCTGTCAA 3'<br>::::::.::::::::::::::<br>miRNA 3' CACGAGGGAGAGAAGACAGTT 5'	0.5	1208-1228	Squamosa promoter-binding-like protein 2

miRNA	Target id	Alignment	Score	Range	Target gene functional annotation
vvi-miR156a	VIT_201s0010g03710.3	target 5' GTGCTCTCTCTCTTCTGTCAA 3'<br>::::::.::::::::::::::<br>miRNA 3' CACGAGGGAGAGAAGACAGTT 5'	0.5	1145-1165	Squamosa promoter-binding-like protein 2
vvi-miR156a	VIT_201s0010g03710.4	target 5' GTGCTCTCTCTCTTCTGTCAA 3'<br>::::::.::::::::::::::<br>miRNA 3' CACGAGGGAGAGAAGACAGTT 5'	0.5	1145-1165	Squamosa promoter-binding-like protein 2
vvi-miR156a	VIT_201s0010g03710.5	target 5' GTGCTCTCTCTCTTCTGTCAA 3'<br>::::::.::::::::::::::<br>miRNA 3' CACGAGGGAGAGAAGACAGTT 5'	0.5	1208-1228	Squamosa promoter-binding-like protein 2
vvi-miR156a	VIT_201s0010g03710.6	target 5' GTGCTCTCTCTCTTCTGTCAA 3'<br>::::::.::::::::::::::<br>miRNA 3' CACGAGGGAGAGAAGACAGTT 5'	0.5	1145-1165	Squamosa promoter-binding-like protein 2
vvi-miR156a	VIT_201s0010g03910.1	target 5' GTGCTCTCTCTCTTCTGTCAT 3'<br>::::::.:::::::::::::<br>miRNA 3' CACGAGGGAGAGAAGACAGTT 5'	1.5	881-901	Squamosa promoter-binding-like protein 13-like
vvi-miR156a	VIT_201s0010g03910.2	target 5' GTGCTCTCTCTCTTCTGTCAT 3'<br>::::::.:::::::::::::<br>miRNA 3' CACGAGGGAGAGAAGACAGTT 5'	1.5	881-901	Squamosa promoter-binding-like protein 13-like
vvi-miR156a	VIT_201s0010g03910.3	target 5' GTGCTCTCTCTCTTCTGTCAT 3'<br>::::::.:::::::::::::<br>miRNA 3' CACGAGGGAGAGAAGACAGTT 5'	1.5	881-901	Squamosa promoter-binding-like protein 13-like
vvi-miR156a	VIT_201s0011g00130.1	target 5' GTGCTCTCTCTCTTCTGTCAG 3'<br>::::::.:::::::::::::.<br>miRNA 3' CACGAGGGAGAGAAGACAGTT 5'	1	1250-1270	Squamosa promoter-binding-like protein 6-like
vvi-miR156a	VIT_201s0011g00130.2	target 5' GTGCTCTCTCTCTTCTGTCAG 3'<br>::::::.:::::::::::::.<br>miRNA 3' CACGAGGGAGAGAAGACAGTT 5'	1	1250-1270	Squamosa promoter-binding-like protein 6-like
vvi-miR156a	VIT_208s0007g06270.1	target 5' GTGCTCTCTCTCTTCTGTCAA 3'<br>::::::.::::::::::::::<br>miRNA 3' CACGAGGGAGAGAAGACAGTT 5'	0.5	788-808	Squamosa promoter-binding-like protein 9-like
vvi-miR156a	VIT_211s0065g00170.1	target 5' GTGCTCTCTCTCTTCTGTCAA 3'<br>::::::.::::::::::::::<br>miRNA 3' CACGAGGGAGAGAAGACAGTT 5'	0.5	1124-1144	Squamosa promoter-binding-like protein 12-like
vvi-miR156a	VIT_211s0065g00170.2	target 5' GTGCTCTCTCTCTTCTGTCAA 3'<br>::::::.::::::::::::::<br>miRNA 3' CACGAGGGAGAGAAGACAGTT 5'	0.5	1124-1144	Squamosa promoter-binding-like protein 12-like
vvi-miR156a	VIT_211s0065g00170.3	target 5' GTGCTCTCTCTCTTCTGTCAA 3'<br>::::::.::::::::::::::<br>miRNA 3' CACGAGGGAGAGAAGACAGTT 5'	0.5	1124-1144	Squamosa promoter-binding-like protein 12-like
vvi-miR156a	VIT_211s0065g00170.4	target 5' GTGCTCTCTCTCTTCTGTCAA 3'<br>::::::.::::::::::::::<br>miRNA 3' CACGAGGGAGAGAAGACAGTT 5'	0.5	1124-1144	Squamosa promoter-binding-like protein 12-like

miRNA	Target id	Alignment	Score	Range	Target gene functional annotation
vvi-miR156a	VIT_211s0065g00170.5	target 5' GTGCTCTCTCTCTTCTGTCAA 3'<br>::::::.::::::::::::::<br>miRNA 3' CACGAGGGAGAGAAGACAGTT 5'	0.5	1124-1144	Squamosa promoter-binding-like protein 12-like
vvi-miR156a	VIT_211s0065g00170.7	target 5' GTGCTCTCTCTCTTCTGTCAA 3'<br>::::::.::::::::::::::<br>miRNA 3' CACGAGGGAGAGAAGACAGTT 5'	0.5	1124-1144	Squamosa promoter-binding-like protein 12-like
vvi-miR156a	VIT_214s0068g01780.1	target 5' GTGCTTTCTCTCTTCTGTCAT 3'<br>:::::..:::::::::::::<br>miRNA 3' CACGAGGGAGAGAAGACAGTT 5'	2	866-886	Squamosa promoter-binding-like protein 16-like
vvi-miR156a	VIT_215s0021g02290.1	target 5' GTGCTCTCTCTCTTCTGTCAT 3'<br>::::::.:::::::::::::<br>miRNA 3' CACGAGGGAGAGAAGACAGTT 5'	1.5	797-817	Squamosa promoter-binding-like protein 7-like
vvi-miR156a	VIT_217s0000g01260.1	target 5' GTGCTCTCTCTCTTCTGTCAT 3'<br>::::::.:::::::::::::<br>miRNA 3' CACGAGGGAGAGAAGACAGTT 5'	1.5	953-973	Squamosa promoter-binding-like protein 13
vvi-miR156a	VIT_217s0000g05020.1	target 5' GTGCTCTCTCTCTTCTGTCAT 3'<br>::::::.:::::::::::::<br>miRNA 3' CACGAGGGAGAGAAGACAGTT 5'	1.5	1232-1252	Squamosa promoter-binding-like protein 6-like
vvi-miR156a	VIT_217s0000g05020.2	target 5' GTGCTCTCTCTCTTCTGTCAT 3'<br>::::::.:::::::::::::<br>miRNA 3' CACGAGGGAGAGAAGACAGTT 5'	1.5	1232-1252	Squamosa promoter-binding-like protein 6-like
vvi-miR156a	VIT_217s0000g05020.3	target 5' GTGCTCTCTCTCTTCTGTCAT 3'<br>::::::.:::::::::::::<br>miRNA 3' CACGAGGGAGAGAAGACAGTT 5'	1.5	1232-1252	Squamosa promoter-binding-like protein 6-like
vvi-miR156b-5p	VIT_201s0010g03710.1	target 5' GTGCTCTCTCTCTTCTGTCA 3'<br>:::::: :::::::::::::<br>miRNA 3' CACGAGTGAGAGAAGACAGT 5'	1	1145-1164	Squamosa promoter-binding-like protein 2
vvi-miR156b-5p	VIT_201s0010g03710.2	target 5' GTGCTCTCTCTCTTCTGTCA 3'<br>:::::: :::::::::::::<br>miRNA 3' CACGAGTGAGAGAAGACAGT 5'	1	1208-1227	Squamosa promoter-binding-like protein 2
vvi-miR156b-5p	VIT_201s0010g03710.3	target 5' GTGCTCTCTCTCTTCTGTCA 3'<br>:::::: :::::::::::::<br>miRNA 3' CACGAGTGAGAGAAGACAGT 5'	1	1145-1164	Squamosa promoter-binding-like protein 2
vvi-miR156b-5p	VIT_201s0010g03710.4	target 5' GTGCTCTCTCTCTTCTGTCA 3'<br>:::::: :::::::::::::<br>miRNA 3' CACGAGTGAGAGAAGACAGT 5'	1	1145-1164	Squamosa promoter-binding-like protein 2
vvi-miR156b-5p	VIT_201s0010g03710.5	target 5' GTGCTCTCTCTCTTCTGTCA 3'<br>:::::: :::::::::::::<br>miRNA 3' CACGAGTGAGAGAAGACAGT 5'	1	1208-1227	Squamosa promoter-binding-like protein 2
vvi-miR156b-5p	VIT_201s0010g03710.6	target 5' GTGCTCTCTCTCTTCTGTCA 3'<br>:::::: :::::::::::::<br>miRNA 3' CACGAGTGAGAGAAGACAGT 5'	1	1145-1164	Squamosa promoter-binding-like protein 2

miRNA	Target id	Alignment	Score	Range	Target gene functional annotation
vvi-miR156b-5p	VIT_201s0010g03910.1	target 5' GTGCTCTCTCTCTTCTGTCA 3'<br>:::::: :::::::::::::<br>miRNA 3' CACGAGTGAGAGAAGACAGT 5'	1	881-900	Squamosa promoter-binding-like protein 13-like
vvi-miR156b-5p	VIT_201s0010g03910.2	target 5' GTGCTCTCTCTCTTCTGTCA 3'<br>:::::: :::::::::::::<br>miRNA 3' CACGAGTGAGAGAAGACAGT 5'	1	881-900	Squamosa promoter-binding-like protein 13-like
vvi-miR156b-5p	VIT_201s0010g03910.3	target 5' GTGCTCTCTCTCTTCTGTCA 3'<br>:::::: :::::::::::::<br>miRNA 3' CACGAGTGAGAGAAGACAGT 5'	1	881-900	Squamosa promoter-binding-like protein 13-like
vvi-miR156b-5p	VIT_201s0011g00130.1	target 5' GTGCTCTCTCTCTTCTGTCA 3'<br>:::::: :::::::::::::<br>miRNA 3' CACGAGTGAGAGAAGACAGT 5'	1	1250-1269	Squamosa promoter-binding-like protein 6-like
vvi-miR156b-5p	VIT_201s0011g00130.2	target 5' GTGCTCTCTCTCTTCTGTCA 3'<br>:::::: :::::::::::::<br>miRNA 3' CACGAGTGAGAGAAGACAGT 5'	1	1250-1269	Squamosa promoter-binding-like protein 6-like
vvi-miR156b-5p	VIT_208s0007g06270.1	target 5' GTGCTCTCTCTCTTCTGTCA 3'<br>:::::: :::::::::::::<br>miRNA 3' CACGAGTGAGAGAAGACAGT 5'	1	788-807	Squamosa promoter-binding-like protein 9-like
vvi-miR156b-5p	VIT_211s0065g00170.1	target 5' GTGCTCTCTCTCTTCTGTCA 3'<br>:::::: :::::::::::::<br>miRNA 3' CACGAGTGAGAGAAGACAGT 5'	1	1124-1143	Squamosa promoter-binding-like protein 12-like
vvi-miR156b-5p	VIT_211s0065g00170.2	target 5' GTGCTCTCTCTCTTCTGTCA 3'<br>:::::: :::::::::::::<br>miRNA 3' CACGAGTGAGAGAAGACAGT 5'	1	1124-1143	Squamosa promoter-binding-like protein 12-like
vvi-miR156b-5p	VIT_211s0065g00170.3	target 5' GTGCTCTCTCTCTTCTGTCA 3'<br>:::::: :::::::::::::<br>miRNA 3' CACGAGTGAGAGAAGACAGT 5'	1	1124-1143	Squamosa promoter-binding-like protein 12-like
vvi-miR156b-5p	VIT_211s0065g00170.4	target 5' GTGCTCTCTCTCTTCTGTCA 3'<br>:::::: :::::::::::::<br>miRNA 3' CACGAGTGAGAGAAGACAGT 5'	1	1124-1143	Squamosa promoter-binding-like protein 12-like
vvi-miR156b-5p	VIT_211s0065g00170.5	target 5' GTGCTCTCTCTCTTCTGTCA 3'<br>:::::: :::::::::::::<br>miRNA 3' CACGAGTGAGAGAAGACAGT 5'	1	1124-1143	Squamosa promoter-binding-like protein 12-like
vvi-miR156b-5p	VIT_211s0065g00170.7	target 5' GTGCTCTCTCTCTTCTGTCA 3'<br>:::::: :::::::::::::<br>miRNA 3' CACGAGTGAGAGAAGACAGT 5'	1	1124-1143	Squamosa promoter-binding-like protein 12-like
vvi-miR156b-5p	VIT_214s0068g01780.1	target 5' GTGCTTTCTCTCTTCTGTCA 3'<br>:::::. :::::::::::::<br>miRNA 3' CACGAGTGAGAGAAGACAGT 5'	1.5	866-885	Squamosa promoter-binding-like protein 16-like
vvi-miR156b-5p	VIT_215s0021g02290.1	target 5' GTGCTCTCTCTCTTCTGTCA 3'<br>:::::: :::::::::::::<br>miRNA 3' CACGAGTGAGAGAAGACAGT 5'	1	797-816	Squamosa promoter-binding-like protein 7-like

miRNA	Target id	Alignment	Score	Range	Target gene functional annotation
vvi-miR156b-5p	VIT_215s0046g01020.1	target 5' GTGATCAACTTTCTTCTGTCA 3'<br>::: :: :::.::::::::::<br>miRNA 3' CACGAG-TGAGAGAAGACAGT 5'	3	1781-1801	Uncharacterized protein loc100241322
vvi-miR156b-5p	VIT_215s0046g01020.2	target 5' GTGATCAACTTTCTTCTGTCA 3'<br>::: :: :::.::::::::::<br>miRNA 3' CACGAG-TGAGAGAAGACAGT 5'	3	1781-1801	Uncharacterized protein loc100241322
vvi-miR156b-5p	VIT_215s0046g01020.3	target 5' GTGATCAACTTTCTTCTGTCA 3'<br>::: :: :::.::::::::::<br>miRNA 3' CACGAG-TGAGAGAAGACAGT 5'	3	1781-1801	Uncharacterized protein loc100241322
vvi-miR156b-5p	VIT_215s0046g01020.4	target 5' GTGATCAACTTTCTTCTGTCA 3'<br>::: :: :::.::::::::::<br>miRNA 3' CACGAG-TGAGAGAAGACAGT 5'	3	1781-1801	Uncharacterized protein loc100241322
vvi-miR156b-5p	VIT_215s0046g01020.5	target 5' GTGATCAACTTTCTTCTGTCA 3'<br>::: :: :::.::::::::::<br>miRNA 3' CACGAG-TGAGAGAAGACAGT 5'	3	836-856	Uncharacterized protein loc100241322
vvi-miR156b-5p	VIT_217s0000g01260.1	target 5' GTGCTCTCTCTCTTCTGTCA 3'<br>:::::: :::::::::::::<br>miRNA 3' CACGAGTGAGAGAAGACAGT 5'	1	953-972	Squamosa promoter-binding-like protein 13
vvi-miR156b-5p	VIT_217s0000g05020.1	target 5' GTGCTCTCTCTCTTCTGTCA 3'<br>:::::: :::::::::::::<br>miRNA 3' CACGAGTGAGAGAAGACAGT 5'	1	1232-1251	Squamosa promoter-binding-like protein 6-like
vvi-miR156b-5p	VIT_217s0000g05020.2	target 5' GTGCTCTCTCTCTTCTGTCA 3'<br>:::::: :::::::::::::<br>miRNA 3' CACGAGTGAGAGAAGACAGT 5'	1	1232-1251	Squamosa promoter-binding-like protein 6-like
vvi-miR156b-5p	VIT_217s0000g05020.3	target 5' GTGCTCTCTCTCTTCTGTCA 3'<br>:::::: :::::::::::::<br>miRNA 3' CACGAGTGAGAGAAGACAGT 5'	1	1232-1251	Squamosa promoter-binding-like protein 6-like
vvi-miR156b-5p	VIT_219s0090g01180.4	target 5' ATGCTCACTCTTTTCTGTCA 3'<br>::::::::::.::::::::<br>miRNA 3' CACGAGTGAGAGAAGACAGT 5'	2	161-180	Uncharacterized protein
vvi-miR156b-5p	VIT_219s0090g01180.7	target 5' ATGCTCACTCTTTTCTGTCA 3'<br>::::::::::.::::::::<br>miRNA 3' CACGAGTGAGAGAAGACAGT 5'	2	161-180	Uncharacterized protein
vvi-miR156c-5p	VIT_201s0010g03710.1	target 5' GTGCTCTCTCTCTTCTGTCA 3'<br>:::::: :::::::::::::<br>miRNA 3' CACGAGTGAGAGAAGACAGT 5'	1	1145-1164	Squamosa promoter-binding-like protein 2
vvi-miR156c-5p	VIT_201s0010g03710.2	target 5' GTGCTCTCTCTCTTCTGTCA 3'<br>:::::: :::::::::::::<br>miRNA 3' CACGAGTGAGAGAAGACAGT 5'	1	1208-1227	Squamosa promoter-binding-like protein 2
vvi-miR156c-5p	VIT_201s0010g03710.3	target 5' GTGCTCTCTCTCTTCTGTCA 3'<br>:::::: :::::::::::::<br>miRNA 3' CACGAGTGAGAGAAGACAGT 5'	1	1145-1164	Squamosa promoter-binding-like protein 2

miRNA	Target id	Alignment	Score	Range	Target gene functional annotation
vvi-miR156c-5p	VIT_201s0010g03710.4	target 5' GTGCTCTCTCTCTTCTGTCA 3'<br>:::::: :::::::::::::<br>miRNA 3' CACGAGTGAGAGAAGACAGT 5'	1	1145-1164	Squamosa promoter-binding-like protein 2
vvi-miR156c-5p	VIT_201s0010g03710.5	target 5' GTGCTCTCTCTCTTCTGTCA 3'<br>:::::: :::::::::::::<br>miRNA 3' CACGAGTGAGAGAAGACAGT 5'	1	1208-1227	Squamosa promoter-binding-like protein 2
vvi-miR156c-5p	VIT_201s0010g03710.6	target 5' GTGCTCTCTCTCTTCTGTCA 3'<br>:::::: :::::::::::::<br>miRNA 3' CACGAGTGAGAGAAGACAGT 5'	1	1145-1164	Squamosa promoter-binding-like protein 2
vvi-miR156c-5p	VIT_201s0010g03910.1	target 5' GTGCTCTCTCTCTTCTGTCA 3'<br>:::::: :::::::::::::<br>miRNA 3' CACGAGTGAGAGAAGACAGT 5'	1	881-900	Squamosa promoter-binding-like protein 13-like
vvi-miR156c-5p	VIT_201s0010g03910.2	target 5' GTGCTCTCTCTCTTCTGTCA 3'<br>:::::: :::::::::::::<br>miRNA 3' CACGAGTGAGAGAAGACAGT 5'	1	881-900	Squamosa promoter-binding-like protein 13-like
vvi-miR156c-5p	VIT_201s0010g03910.3	target 5' GTGCTCTCTCTCTTCTGTCA 3'<br>:::::: :::::::::::::<br>miRNA 3' CACGAGTGAGAGAAGACAGT 5'	1	881-900	Squamosa promoter-binding-like protein 13-like
vvi-miR156c-5p	VIT_201s0011g00130.1	target 5' GTGCTCTCTCTCTTCTGTCA 3'<br>:::::: :::::::::::::<br>miRNA 3' CACGAGTGAGAGAAGACAGT 5'	1	1250-1269	Squamosa promoter-binding-like protein 6-like
vvi-miR156c-5p	VIT_201s0011g00130.2	target 5' GTGCTCTCTCTCTTCTGTCA 3'<br>:::::: :::::::::::::<br>miRNA 3' CACGAGTGAGAGAAGACAGT 5'	1	1250-1269	Squamosa promoter-binding-like protein 6-like
vvi-miR156c-5p	VIT_208s0007g06270.1	target 5' GTGCTCTCTCTCTTCTGTCA 3'<br>:::::: :::::::::::::<br>miRNA 3' CACGAGTGAGAGAAGACAGT 5'	1	788-807	Squamosa promoter-binding-like protein 9-like
vvi-miR156c-5p	VIT_211s0065g00170.1	target 5' GTGCTCTCTCTCTTCTGTCA 3'<br>:::::: :::::::::::::<br>miRNA 3' CACGAGTGAGAGAAGACAGT 5'	1	1124-1143	Squamosa promoter-binding-like protein 12-like
vvi-miR156c-5p	VIT_211s0065g00170.2	target 5' GTGCTCTCTCTCTTCTGTCA 3'<br>:::::: :::::::::::::<br>miRNA 3' CACGAGTGAGAGAAGACAGT 5'	1	1124-1143	Squamosa promoter-binding-like protein 12-like
vvi-miR156c-5p	VIT_211s0065g00170.3	target 5' GTGCTCTCTCTCTTCTGTCA 3'<br>:::::: :::::::::::::<br>miRNA 3' CACGAGTGAGAGAAGACAGT 5'	1	1124-1143	Squamosa promoter-binding-like protein 12-like
vvi-miR156c-5p	VIT_211s0065g00170.4	target 5' GTGCTCTCTCTCTTCTGTCA 3'<br>:::::: :::::::::::::<br>miRNA 3' CACGAGTGAGAGAAGACAGT 5'	1	1124-1143	Squamosa promoter-binding-like protein 12-like
vvi-miR156c-5p	VIT_211s0065g00170.5	target 5' GTGCTCTCTCTCTTCTGTCA 3'<br>:::::: :::::::::::::<br>miRNA 3' CACGAGTGAGAGAAGACAGT 5'	1	1124-1143	Squamosa promoter-binding-like protein 12-like

miRNA	Target id	Alignment	Score	Range	Target gene functional annotation
vvi-miR156c-5p	VIT_211s0065g00170.7	target 5' GTGCTCTCTCTCTTCTGTCA 3'<br>:::::: :::::::::::::<br>miRNA 3' CACGAGTGAGAGAAGACAGT 5'	1	1124-1143	Squamosa promoter-binding-like protein 12-like
vvi-miR156c-5p	VIT_214s0068g01780.1	target 5' GTGCTTTCTCTCTTCTGTCA 3'<br>:::::. :::::::::::::<br>miRNA 3' CACGAGTGAGAGAAGACAGT 5'	1.5	866-885	Squamosa promoter-binding-like protein 16-like
vvi-miR156c-5p	VIT_215s0021g02290.1	target 5' GTGCTCTCTCTCTTCTGTCA 3'<br>:::::: :::::::::::::<br>miRNA 3' CACGAGTGAGAGAAGACAGT 5'	1	797-816	Squamosa promoter-binding-like protein 7-like
vvi-miR156c-5p	VIT_215s0046g01020.1	target 5' GTGATCAACTTTCTTCTGTCA 3'<br>::: :: :::.::::::::::<br>miRNA 3' CACGAG-TGAGAGAAGACAGT 5'	3	1781-1801	Uncharacterized protein loc100241322
vvi-miR156c-5p	VIT_215s0046g01020.2	target 5' GTGATCAACTTTCTTCTGTCA 3'<br>::: :: :::.::::::::::<br>miRNA 3' CACGAG-TGAGAGAAGACAGT 5'	3	1781-1801	Uncharacterized protein loc100241322
vvi-miR156c-5p	VIT_215s0046g01020.3	target 5' GTGATCAACTTTCTTCTGTCA 3'<br>::: :: :::.::::::::::<br>miRNA 3' CACGAG-TGAGAGAAGACAGT 5'	3	1781-1801	Uncharacterized protein loc100241322
vvi-miR156c-5p	VIT_215s0046g01020.4	target 5' GTGATCAACTTTCTTCTGTCA 3'<br>::: :: :::.::::::::::<br>miRNA 3' CACGAG-TGAGAGAAGACAGT 5'	3	1781-1801	Uncharacterized protein loc100241322
vvi-miR156c-5p	VIT_215s0046g01020.5	target 5' GTGATCAACTTTCTTCTGTCA 3'<br>::: :: :::.::::::::::<br>miRNA 3' CACGAG-TGAGAGAAGACAGT 5'	3	836-856	Uncharacterized protein loc100241322
vvi-miR156c-5p	VIT_217s0000g01260.1	target 5' GTGCTCTCTCTCTTCTGTCA 3'<br>:::::: :::::::::::::<br>miRNA 3' CACGAGTGAGAGAAGACAGT 5'	1	953-972	Squamosa promoter-binding-like protein 13
vvi-miR156c-5p	VIT_217s0000g05020.1	target 5' GTGCTCTCTCTCTTCTGTCA 3'<br>:::::: :::::::::::::<br>miRNA 3' CACGAGTGAGAGAAGACAGT 5'	1	1232-1251	Squamosa promoter-binding-like protein 6-like
vvi-miR156c-5p	VIT_217s0000g05020.2	target 5' GTGCTCTCTCTCTTCTGTCA 3'<br>:::::: :::::::::::::<br>miRNA 3' CACGAGTGAGAGAAGACAGT 5'	1	1232-1251	Squamosa promoter-binding-like protein 6-like
vvi-miR156c-5p	VIT_217s0000g05020.3	target 5' GTGCTCTCTCTCTTCTGTCA 3'<br>:::::: :::::::::::::<br>miRNA 3' CACGAGTGAGAGAAGACAGT 5'	1	1232-1251	Squamosa promoter-binding-like protein 6-like
vvi-miR156c-5p	VIT_219s0090g01180.4	target 5' ATGCTCACTCTTTTCTGTCA 3'<br>:::::::::.:::::::::<br>miRNA 3' CACGAGTGAGAGAAGACAGT 5'	2	161-180	Uncharacterized protein
vvi-miR156c-5p	VIT_219s0090g01180.7	target 5' ATGCTCACTCTTTTCTGTCA 3'<br>:::::::::.:::::::::<br>miRNA 3' CACGAGTGAGAGAAGACAGT 5'	2	161-180	Uncharacterized protein

miRNA	Target id	Alignment	Score	Range	Target gene functional annotation
vvi-miR156d-5p	VIT_201s0010g03710.1	target 5' GTGCTCTCTCTCTTCTGTCA 3'<br>:::::: :::::::::::::<br>miRNA 3' CACGAGTGAGAGAAGACAGT 5'	1	1145-1164	Squamosa promoter-binding-like protein 2
vvi-miR156d-5p	VIT_201s0010g03710.2	target 5' GTGCTCTCTCTCTTCTGTCA 3'<br>:::::: :::::::::::::<br>miRNA 3' CACGAGTGAGAGAAGACAGT 5'	1	1208-1227	Squamosa promoter-binding-like protein 2
vvi-miR156d-5p	VIT_201s0010g03710.3	target 5' GTGCTCTCTCTCTTCTGTCA 3'<br>:::::: :::::::::::::<br>miRNA 3' CACGAGTGAGAGAAGACAGT 5'	1	1145-1164	Squamosa promoter-binding-like protein 2
vvi-miR156d-5p	VIT_201s0010g03710.4	target 5' GTGCTCTCTCTCTTCTGTCA 3'<br>:::::: :::::::::::::<br>miRNA 3' CACGAGTGAGAGAAGACAGT 5'	1	1145-1164	Squamosa promoter-binding-like protein 2
vvi-miR156d-5p	VIT_201s0010g03710.5	target 5' GTGCTCTCTCTCTTCTGTCA 3'<br>:::::: :::::::::::::<br>miRNA 3' CACGAGTGAGAGAAGACAGT 5'	1	1208-1227	Squamosa promoter-binding-like protein 2
vvi-miR156d-5p	VIT_201s0010g03710.6	target 5' GTGCTCTCTCTCTTCTGTCA 3'<br>:::::: :::::::::::::<br>miRNA 3' CACGAGTGAGAGAAGACAGT 5'	1	1145-1164	Squamosa promoter-binding-like protein 2
vvi-miR156d-5p	VIT_201s0010g03910.1	target 5' GTGCTCTCTCTCTTCTGTCA 3'<br>:::::: :::::::::::::<br>miRNA 3' CACGAGTGAGAGAAGACAGT 5'	1	881-900	Squamosa promoter-binding-like protein 13-like
vvi-miR156d-5p	VIT_201s0010g03910.2	target 5' GTGCTCTCTCTCTTCTGTCA 3'<br>:::::: :::::::::::::<br>miRNA 3' CACGAGTGAGAGAAGACAGT 5'	1	881-900	Squamosa promoter-binding-like protein 13-like
vvi-miR156d-5p	VIT_201s0010g03910.3	target 5' GTGCTCTCTCTCTTCTGTCA 3'<br>:::::: :::::::::::::<br>miRNA 3' CACGAGTGAGAGAAGACAGT 5'	1	881-900	Squamosa promoter-binding-like protein 13-like
vvi-miR156d-5p	VIT_201s0011g00130.1	target 5' GTGCTCTCTCTCTTCTGTCA 3'<br>:::::: :::::::::::::<br>miRNA 3' CACGAGTGAGAGAAGACAGT 5'	1	1250-1269	Squamosa promoter-binding-like protein 6-like
vvi-miR156d-5p	VIT_201s0011g00130.2	target 5' GTGCTCTCTCTCTTCTGTCA 3'<br>:::::: :::::::::::::<br>miRNA 3' CACGAGTGAGAGAAGACAGT 5'	1	1250-1269	Squamosa promoter-binding-like protein 6-like
vvi-miR156d-5p	VIT_208s0007g06270.1	target 5' GTGCTCTCTCTCTTCTGTCA 3'<br>:::::: :::::::::::::<br>miRNA 3' CACGAGTGAGAGAAGACAGT 5'	1	788-807	Squamosa promoter-binding-like protein 9-like
vvi-miR156d-5p	VIT_211s0065g00170.1	target 5' GTGCTCTCTCTCTTCTGTCA 3'<br>:::::: :::::::::::::<br>miRNA 3' CACGAGTGAGAGAAGACAGT 5'	1	1124-1143	Squamosa promoter-binding-like protein 12-like
vvi-miR156d-5p	VIT_211s0065g00170.2	target 5' GTGCTCTCTCTCTTCTGTCA 3'<br>:::::: :::::::::::::<br>miRNA 3' CACGAGTGAGAGAAGACAGT 5'	1	1124-1143	Squamosa promoter-binding-like protein 12-like

miRNA	Target id	Alignment	Score	Range	Target gene functional annotation
vvi-miR156d-5p	VIT_211s0065g00170.3	target 5' GTGCTCTCTCTCTTCTGTCA 3'<br>:::::: :::::::::::::<br>miRNA 3' CACGAGTGAGAGAAGACAGT 5'	1	1124-1143	Squamosa promoter-binding-like protein 12-like
vvi-miR156d-5p	VIT_211s0065g00170.4	target 5' GTGCTCTCTCTCTTCTGTCA 3'<br>:::::: :::::::::::::<br>miRNA 3' CACGAGTGAGAGAAGACAGT 5'	1	1124-1143	Squamosa promoter-binding-like protein 12-like
vvi-miR156d-5p	VIT_211s0065g00170.5	target 5' GTGCTCTCTCTCTTCTGTCA 3'<br>:::::: :::::::::::::<br>miRNA 3' CACGAGTGAGAGAAGACAGT 5'	1	1124-1143	Squamosa promoter-binding-like protein 12-like
vvi-miR156d-5p	VIT_211s0065g00170.7	target 5' GTGCTCTCTCTCTTCTGTCA 3'<br>:::::: :::::::::::::<br>miRNA 3' CACGAGTGAGAGAAGACAGT 5'	1	1124-1143	Squamosa promoter-binding-like protein 12-like
vvi-miR156d-5p	VIT_214s0068g01780.1	target 5' GTGCTTTCTCTCTTCTGTCA 3'<br>:::::. :::::::::::::<br>miRNA 3' CACGAGTGAGAGAAGACAGT 5'	1.5	866-885	Squamosa promoter-binding-like protein 16-like
vvi-miR156d-5p	VIT_215s0021g02290.1	target 5' GTGCTCTCTCTCTTCTGTCA 3'<br>:::::: :::::::::::::<br>miRNA 3' CACGAGTGAGAGAAGACAGT 5'	1	797-816	Squamosa promoter-binding-like protein 7-like
vvi-miR156d-5p	VIT_215s0046g01020.1	target 5' GTGATCAACTTTCTTCTGTCA 3'<br>::: :: :::.::::::::::<br>miRNA 3' CACGAG-TGAGAGAAGACAGT 5'	3	1781-1801	Uncharacterized protein loc100241322
vvi-miR156d-5p	VIT_215s0046g01020.2	target 5' GTGATCAACTTTCTTCTGTCA 3'<br>::: :: :::.::::::::::<br>miRNA 3' CACGAG-TGAGAGAAGACAGT 5'	3	1781-1801	Uncharacterized protein loc100241322
vvi-miR156d-5p	VIT_215s0046g01020.3	target 5' GTGATCAACTTTCTTCTGTCA 3'<br>::: :: :::.::::::::::<br>miRNA 3' CACGAG-TGAGAGAAGACAGT 5'	3	1781-1801	Uncharacterized protein loc100241322
vvi-miR156d-5p	VIT_215s0046g01020.4	target 5' GTGATCAACTTTCTTCTGTCA 3'<br>::: :: :::.::::::::::<br>miRNA 3' CACGAG-TGAGAGAAGACAGT 5'	3	1781-1801	Uncharacterized protein loc100241322
vvi-miR156d-5p	VIT_215s0046g01020.5	target 5' GTGATCAACTTTCTTCTGTCA 3'<br>::: :: :::.::::::::::<br>miRNA 3' CACGAG-TGAGAGAAGACAGT 5'	3	836-856	Uncharacterized protein loc100241322
vvi-miR156d-5p	VIT_217s0000g01260.1	target 5' GTGCTCTCTCTCTTCTGTCA 3'<br>:::::: :::::::::::::<br>miRNA 3' CACGAGTGAGAGAAGACAGT 5'	1	953-972	Squamosa promoter-binding-like protein 13
vvi-miR156d-5p	VIT_217s0000g05020.1	target 5' GTGCTCTCTCTCTTCTGTCA 3'<br>:::::: :::::::::::::<br>miRNA 3' CACGAGTGAGAGAAGACAGT 5'	1	1232-1251	Squamosa promoter-binding-like protein 6-like
vvi-miR156d-5p	VIT_217s0000g05020.2	target 5' GTGCTCTCTCTCTTCTGTCA 3'<br>:::::: :::::::::::::<br>miRNA 3' CACGAGTGAGAGAAGACAGT 5'	1	1232-1251	Squamosa promoter-binding-like protein 6-like

miRNA	Target id	Alignment	Score	Range	Target gene functional annotation
vvi-miR156d-5p	VIT_217s0000g05020.3	target 5' GTGCTCTCTCTCTTCTGTCA 3'<br>:::::: :::::::::::::<br>miRNA 3' CACGAGTGAGAGAAGACAGT 5'	1	1232-1251	Squamosa promoter-binding-like protein 6-like
vvi-miR156d-5p	VIT_219s0090g01180.4	target 5' ATGCTCACTCTTTTCTGTCA 3'<br>::::::::::.::::::::<br>miRNA 3' CACGAGTGAGAGAAGACAGT 5'	2	161-180	Uncharacterized protein
vvi-miR156d-5p	VIT_219s0090g01180.7	target 5' ATGCTCACTCTTTTCTGTCA 3'<br>::::::::::.::::::::<br>miRNA 3' CACGAGTGAGAGAAGACAGT 5'	2	161-180	Uncharacterized protein
vvi-miR156e	VIT_201s0010g03710.1	target 5' GTGCTCTCTCTCTTCTGTCA 3'<br>:::::: :::::.:::::::<br>miRNA 3' CACGAGTGAGAGGAGACAGT 5'	2	1145-1164	Squamosa promoter-binding-like protein 2
vvi-miR156e	VIT_201s0010g03710.2	target 5' GTGCTCTCTCTCTTCTGTCA 3'<br>:::::: :::::.:::::::<br>miRNA 3' CACGAGTGAGAGGAGACAGT 5'	2	1208-1227	Squamosa promoter-binding-like protein 2
vvi-miR156e	VIT_201s0010g03710.3	target 5' GTGCTCTCTCTCTTCTGTCA 3'<br>:::::: :::::.:::::::<br>miRNA 3' CACGAGTGAGAGGAGACAGT 5'	2	1145-1164	Squamosa promoter-binding-like protein 2
vvi-miR156e	VIT_201s0010g03710.4	target 5' GTGCTCTCTCTCTTCTGTCA 3'<br>:::::: :::::.:::::::<br>miRNA 3' CACGAGTGAGAGGAGACAGT 5'	2	1145-1164	Squamosa promoter-binding-like protein 2
vvi-miR156e	VIT_201s0010g03710.5	target 5' GTGCTCTCTCTCTTCTGTCA 3'<br>:::::: :::::.:::::::<br>miRNA 3' CACGAGTGAGAGGAGACAGT 5'	2	1208-1227	Squamosa promoter-binding-like protein 2
vvi-miR156e	VIT_201s0010g03710.6	target 5' GTGCTCTCTCTCTTCTGTCA 3'<br>:::::: :::::.:::::::<br>miRNA 3' CACGAGTGAGAGGAGACAGT 5'	2	1145-1164	Squamosa promoter-binding-like protein 2
vvi-miR156e	VIT_201s0010g03910.1	target 5' GTGCTCTCTCTCTTCTGTCA 3'<br>:::::: :::::.:::::::<br>miRNA 3' CACGAGTGAGAGGAGACAGT 5'	2	881-900	Squamosa promoter-binding-like protein 13-like
vvi-miR156e	VIT_201s0010g03910.2	target 5' GTGCTCTCTCTCTTCTGTCA 3'<br>:::::: :::::.:::::::<br>miRNA 3' CACGAGTGAGAGGAGACAGT 5'	2	881-900	Squamosa promoter-binding-like protein 13-like
vvi-miR156e	VIT_201s0010g03910.3	target 5' GTGCTCTCTCTCTTCTGTCA 3'<br>:::::: :::::.:::::::<br>miRNA 3' CACGAGTGAGAGGAGACAGT 5'	2	881-900	Squamosa promoter-binding-like protein 13-like
vvi-miR156e	VIT_201s0011g00130.1	target 5' GTGCTCTCTCTCTTCTGTCA 3'<br>:::::: :::::.:::::::<br>miRNA 3' CACGAGTGAGAGGAGACAGT 5'	2	1250-1269	Squamosa promoter-binding-like protein 6-like
vvi-miR156e	VIT_201s0011g00130.2	target 5' GTGCTCTCTCTCTTCTGTCA 3'<br>:::::: :::::.:::::::<br>miRNA 3' CACGAGTGAGAGGAGACAGT 5'	2	1250-1269	Squamosa promoter-binding-like protein 6-like

miRNA	Target id	Alignment	Score	Range	Target gene functional annotation
vvi-miR156e	VIT_204s0008g02640.1	target 5' GTGCTC-TTCTCCTCTGTCA 3'<br>:::::: .::::::::::::<br>miRNA 3' CACGAGTGAGAGGAGACAGT 5'	2	16-34	Plastid-lipid-associated protein 8
vvi-miR156e	VIT_204s0008g02640.2	target 5' GTGCTC-TTCTCCTCTGTCA 3'<br>:::::: .::::::::::::<br>miRNA 3' CACGAGTGAGAGGAGACAGT 5'	2	16-34	Plastid-lipid-associated protein 8
vvi-miR156e	VIT_208s0007g06270.1	target 5' GTGCTCTCTCTCTTCTGTCA 3'<br>:::::: :::::.:::::::<br>miRNA 3' CACGAGTGAGAGGAGACAGT 5'	2	788-807	Squamosa promoter-binding-like protein 9-like
vvi-miR156e	VIT_211s0065g00170.1	target 5' GTGCTCTCTCTCTTCTGTCA 3'<br>:::::: :::::.:::::::<br>miRNA 3' CACGAGTGAGAGGAGACAGT 5'	2	1124-1143	Squamosa promoter-binding-like protein 12-like
vvi-miR156e	VIT_211s0065g00170.2	target 5' GTGCTCTCTCTCTTCTGTCA 3'<br>:::::: :::::.:::::::<br>miRNA 3' CACGAGTGAGAGGAGACAGT 5'	2	1124-1143	Squamosa promoter-binding-like protein 12-like
vvi-miR156e	VIT_211s0065g00170.3	target 5' GTGCTCTCTCTCTTCTGTCA 3'<br>:::::: :::::.:::::::<br>miRNA 3' CACGAGTGAGAGGAGACAGT 5'	2	1124-1143	Squamosa promoter-binding-like protein 12-like
vvi-miR156e	VIT_211s0065g00170.4	target 5' GTGCTCTCTCTCTTCTGTCA 3'<br>:::::: :::::.:::::::<br>miRNA 3' CACGAGTGAGAGGAGACAGT 5'	2	1124-1143	Squamosa promoter-binding-like protein 12-like
vvi-miR156e	VIT_211s0065g00170.5	target 5' GTGCTCTCTCTCTTCTGTCA 3'<br>:::::: :::::.:::::::<br>miRNA 3' CACGAGTGAGAGGAGACAGT 5'	2	1124-1143	Squamosa promoter-binding-like protein 12-like
vvi-miR156e	VIT_211s0065g00170.7	target 5' GTGCTCTCTCTCTTCTGTCA 3'<br>:::::: :::::.:::::::<br>miRNA 3' CACGAGTGAGAGGAGACAGT 5'	2	1124-1143	Squamosa promoter-binding-like protein 12-like
vvi-miR156e	VIT_214s0068g01780.1	target 5' GTGCTTTCTCTCTTCTGTCA 3'<br>:::::. :::::.:::::::<br>miRNA 3' CACGAGTGAGAGGAGACAGT 5'	2.5	866-885	Squamosa promoter-binding-like protein 16-like
vvi-miR156e	VIT_215s0021g02290.1	target 5' GTGCTCTCTCTCTTCTGTCA 3'<br>:::::: :::::.:::::::<br>miRNA 3' CACGAGTGAGAGGAGACAGT 5'	2	797-816	Squamosa promoter-binding-like protein 7-like
vvi-miR156e	VIT_217s0000g01260.1	target 5' GTGCTCTCTCTCTTCTGTCA 3'<br>:::::: :::::.:::::::<br>miRNA 3' CACGAGTGAGAGGAGACAGT 5'	2	953-972	Squamosa promoter-binding-like protein 13
vvi-miR156e	VIT_217s0000g05020.1	target 5' GTGCTCTCTCTCTTCTGTCA 3'<br>:::::: :::::.:::::::<br>miRNA 3' CACGAGTGAGAGGAGACAGT 5'	2	1232-1251	Squamosa promoter-binding-like protein 6-like
vvi-miR156e	VIT_217s0000g05020.2	target 5' GTGCTCTCTCTCTTCTGTCA 3'<br>:::::: :::::.:::::::<br>miRNA 3' CACGAGTGAGAGGAGACAGT 5'	2	1232-1251	Squamosa promoter-binding-like protein 6-like

miRNA	Target id	Alignment	Score	Range	Target gene functional annotation
vvi-miR156e	VIT_217s0000g05020.3	target 5' GTGCTCTCTCTCTTCTGTCA 3'<br>:::::: :::::.:::::::<br>miRNA 3' CACGAGTGAGAGGAGACAGT 5'	2	1232-1251	Squamosa promoter-binding-like protein 6-like
vvi-miR156e	VIT_219s0090g01180.4	target 5' ATGCTCACTCTTTTCTGTCA 3'<br>::::::::::..:::::::<br>miRNA 3' CACGAGTGAGAGGAGACAGT 5'	3	161-180	Uncharacterized protein
vvi-miR156e	VIT_219s0090g01180.7	target 5' ATGCTCACTCTTTTCTGTCA 3'<br>::::::::::..:::::::<br>miRNA 3' CACGAGTGAGAGGAGACAGT 5'	3	161-180	Uncharacterized protein
vvi-miR156f	VIT_201s0010g03710.1	target 5' GTGCTCTCTCTCTTCTGTCAA 3'<br>::::::::: :::::::::::<br>miRNA 3' CACGAGAGATAGAAGACAGTT 5'	2	1145-1165	Squamosa promoter-binding-like protein 2
vvi-miR156f	VIT_201s0010g03710.2	target 5' GTGCTCTCTCTCTTCTGTCAA 3'<br>::::::::: :::::::::::<br>miRNA 3' CACGAGAGATAGAAGACAGTT 5'	2	1208-1228	Squamosa promoter-binding-like protein 2
vvi-miR156f	VIT_201s0010g03710.3	target 5' GTGCTCTCTCTCTTCTGTCAA 3'<br>::::::::: :::::::::::<br>miRNA 3' CACGAGAGATAGAAGACAGTT 5'	2	1145-1165	Squamosa promoter-binding-like protein 2
vvi-miR156f	VIT_201s0010g03710.4	target 5' GTGCTCTCTCTCTTCTGTCAA 3'<br>::::::::: :::::::::::<br>miRNA 3' CACGAGAGATAGAAGACAGTT 5'	2	1145-1165	Squamosa promoter-binding-like protein 2
vvi-miR156f	VIT_201s0010g03710.5	target 5' GTGCTCTCTCTCTTCTGTCAA 3'<br>::::::::: :::::::::::<br>miRNA 3' CACGAGAGATAGAAGACAGTT 5'	2	1208-1228	Squamosa promoter-binding-like protein 2
vvi-miR156f	VIT_201s0010g03710.6	target 5' GTGCTCTCTCTCTTCTGTCAA 3'<br>::::::::: :::::::::::<br>miRNA 3' CACGAGAGATAGAAGACAGTT 5'	2	1145-1165	Squamosa promoter-binding-like protein 2
vvi-miR156f	VIT_201s0010g03910.1	target 5' GTGCTCTCTCTCTTCTGTCAT 3'<br>::::::::: ::::::::::<br>miRNA 3' CACGAGAGATAGAAGACAGTT 5'	3	881-901	Squamosa promoter-binding-like protein 13-like
vvi-miR156f	VIT_201s0010g03910.2	target 5' GTGCTCTCTCTCTTCTGTCAT 3'<br>::::::::: ::::::::::<br>miRNA 3' CACGAGAGATAGAAGACAGTT 5'	3	881-901	Squamosa promoter-binding-like protein 13-like
vvi-miR156f	VIT_201s0010g03910.3	target 5' GTGCTCTCTCTCTTCTGTCAT 3'<br>::::::::: ::::::::::<br>miRNA 3' CACGAGAGATAGAAGACAGTT 5'	3	881-901	Squamosa promoter-binding-like protein 13-like
vvi-miR156f	VIT_201s0011g00130.1	target 5' GTGCTCTCTCTCTTCTGTCAG 3'<br>::::::::: ::::::::::.<br>miRNA 3' CACGAGAGATAGAAGACAGTT 5'	2.5	1250-1270	Squamosa promoter-binding-like protein 6-like
vvi-miR156f	VIT_201s0011g00130.2	target 5' GTGCTCTCTCTCTTCTGTCAG 3'<br>::::::::: ::::::::::.<br>miRNA 3' CACGAGAGATAGAAGACAGTT 5'	2.5	1250-1270	Squamosa promoter-binding-like protein 6-like

miRNA	Target id	Alignment	Score	Range	Target gene functional annotation
vvi-miR156f	VIT_208s0007g06270.1	target 5' GTGCTCTCTCTCTTCTGTCAA 3'<br>::::::::: :::::::::::<br>miRNA 3' CACGAGAGATAGAAGACAGTT 5'	2	788-808	Squamosa promoter-binding-like protein 9-like
vvi-miR156f	VIT_211s0065g00170.1	target 5' GTGCTCTCTCTCTTCTGTCAA 3'<br>::::::::: :::::::::::<br>miRNA 3' CACGAGAGATAGAAGACAGTT 5'	2	1124-1144	Squamosa promoter-binding-like protein 12-like
vvi-miR156f	VIT_211s0065g00170.2	target 5' GTGCTCTCTCTCTTCTGTCAA 3'<br>::::::::: :::::::::::<br>miRNA 3' CACGAGAGATAGAAGACAGTT 5'	2	1124-1144	Squamosa promoter-binding-like protein 12-like
vvi-miR156f	VIT_211s0065g00170.3	target 5' GTGCTCTCTCTCTTCTGTCAA 3'<br>::::::::: :::::::::::<br>miRNA 3' CACGAGAGATAGAAGACAGTT 5'	2	1124-1144	Squamosa promoter-binding-like protein 12-like
vvi-miR156f	VIT_211s0065g00170.4	target 5' GTGCTCTCTCTCTTCTGTCAA 3'<br>::::::::: :::::::::::<br>miRNA 3' CACGAGAGATAGAAGACAGTT 5'	2	1124-1144	Squamosa promoter-binding-like protein 12-like
vvi-miR156f	VIT_211s0065g00170.5	target 5' GTGCTCTCTCTCTTCTGTCAA 3'<br>::::::::: :::::::::::<br>miRNA 3' CACGAGAGATAGAAGACAGTT 5'	2	1124-1144	Squamosa promoter-binding-like protein 12-like
vvi-miR156f	VIT_211s0065g00170.7	target 5' GTGCTCTCTCTCTTCTGTCAA 3'<br>::::::::: :::::::::::<br>miRNA 3' CACGAGAGATAGAAGACAGTT 5'	2	1124-1144	Squamosa promoter-binding-like protein 12-like
vvi-miR156f	VIT_215s0021g02290.1	target 5' GTGCTCTCTCTCTTCTGTCAT 3'<br>::::::::: ::::::::::<br>miRNA 3' CACGAGAGATAGAAGACAGTT 5'	3	797-817	Squamosa promoter-binding-like protein 7-like
vvi-miR156f	VIT_217s0000g01260.1	target 5' GTGCTCTCTCTCTTCTGTCAT 3'<br>::::::::: ::::::::::<br>miRNA 3' CACGAGAGATAGAAGACAGTT 5'	3	953-973	Squamosa promoter-binding-like protein 13
vvi-miR156f	VIT_217s0000g05020.1	target 5' GTGCTCTCTCTCTTCTGTCAT 3'<br>::::::::: ::::::::::<br>miRNA 3' CACGAGAGATAGAAGACAGTT 5'	3	1232-1252	Squamosa promoter-binding-like protein 6-like
vvi-miR156f	VIT_217s0000g05020.2	target 5' GTGCTCTCTCTCTTCTGTCAT 3'<br>::::::::: ::::::::::<br>miRNA 3' CACGAGAGATAGAAGACAGTT 5'	3	1232-1252	Squamosa promoter-binding-like protein 6-like
vvi-miR156f	VIT_217s0000g05020.3	target 5' GTGCTCTCTCTCTTCTGTCAT 3'<br>::::::::: ::::::::::<br>miRNA 3' CACGAGAGATAGAAGACAGTT 5'	3	1232-1252	Squamosa promoter-binding-like protein 6-like
vvi-miR156g-5p	VIT_201s0010g03710.1	target 5' GTGCTCTCTCTCTTCTGTCAA 3'<br>::::::::: :::::::::::<br>miRNA 3' CACGAGAGATAGAAGACAGTT 5'	2	1145-1165	Squamosa promoter-binding-like protein 2
vvi-miR156g-5p	VIT_201s0010g03710.2	target 5' GTGCTCTCTCTCTTCTGTCAA 3'<br>::::::::: :::::::::::<br>miRNA 3' CACGAGAGATAGAAGACAGTT 5'	2	1208-1228	Squamosa promoter-binding-like protein 2

miRNA	Target id	Alignment	Score	Range	Target gene functional annotation
vvi-miR156g-5p	VIT_201s0010g03710.3	target 5' GTGCTCTCTCTCTTCTGTCAA 3'<br>:::::::: ::::::::::<br>miRNA 3' CACGAGAGATAGAAGACAGTT 5'	2	1145-1165	Squamosa promoter-binding-like protein 2
vvi-miR156g-5p	VIT_201s0010g03710.4	target 5' GTGCTCTCTCTCTTCTGTCAA 3'<br>:::::::: ::::::::::<br>miRNA 3' CACGAGAGATAGAAGACAGTT 5'	2	1145-1165	Squamosa promoter-binding-like protein 2
vvi-miR156g-5p	VIT_201s0010g03710.5	target 5' GTGCTCTCTCTCTTCTGTCAA 3'<br>:::::::: ::::::::::<br>miRNA 3' CACGAGAGATAGAAGACAGTT 5'	2	1208-1228	Squamosa promoter-binding-like protein 2
vvi-miR156g-5p	VIT_201s0010g03710.6	target 5' GTGCTCTCTCTCTTCTGTCAA 3'<br>:::::::: ::::::::::<br>miRNA 3' CACGAGAGATAGAAGACAGTT 5'	2	1145-1165	Squamosa promoter-binding-like protein 2
vvi-miR156g-5p	VIT_201s0010g03910.1	target 5' GTGCTCTCTCTCTTCTGTCAT 3'<br>:::::::: :::::::::<br>miRNA 3' CACGAGAGATAGAAGACAGTT 5'	3	881-901	Squamosa promoter-binding-like protein 13-like
vvi-miR156g-5p	VIT_201s0010g03910.2	target 5' GTGCTCTCTCTCTTCTGTCAT 3'<br>:::::::: :::::::::<br>miRNA 3' CACGAGAGATAGAAGACAGTT 5'	3	881-901	Squamosa promoter-binding-like protein 13-like
vvi-miR156g-5p	VIT_201s0010g03910.3	target 5' GTGCTCTCTCTCTTCTGTCAT 3'<br>:::::::: :::::::::<br>miRNA 3' CACGAGAGATAGAAGACAGTT 5'	3	881-901	Squamosa promoter-binding-like protein 13-like
vvi-miR156g-5p	VIT_201s0011g00130.1	target 5' GTGCTCTCTCTCTTCTGTCAG 3'<br>:::::::: :::::::::.<br>miRNA 3' CACGAGAGATAGAAGACAGTT 5'	2.5	1250-1270	Squamosa promoter-binding-like protein 6-like
vvi-miR156g-5p	VIT_201s0011g00130.2	target 5' GTGCTCTCTCTCTTCTGTCAG 3'<br>:::::::: :::::::::.<br>miRNA 3' CACGAGAGATAGAAGACAGTT 5'	2.5	1250-1270	Squamosa promoter-binding-like protein 6-like
vvi-miR156g-5p	VIT_208s0007g06270.1	target 5' GTGCTCTCTCTCTTCTGTCAA 3'<br>:::::::: ::::::::::<br>miRNA 3' CACGAGAGATAGAAGACAGTT 5'	2	788-808	Squamosa promoter-binding-like protein 9-like
vvi-miR156g-5p	VIT_211s0065g00170.1	target 5' GTGCTCTCTCTCTTCTGTCAA 3'<br>:::::::: ::::::::::<br>miRNA 3' CACGAGAGATAGAAGACAGTT 5'	2	1124-1144	Squamosa promoter-binding-like protein 12-like
vvi-miR156g-5p	VIT_211s0065g00170.2	target 5' GTGCTCTCTCTCTTCTGTCAA 3'<br>:::::::: ::::::::::<br>miRNA 3' CACGAGAGATAGAAGACAGTT 5'	2	1124-1144	Squamosa promoter-binding-like protein 12-like
vvi-miR156g-5p	VIT_211s0065g00170.3	target 5' GTGCTCTCTCTCTTCTGTCAA 3'<br>:::::::: ::::::::::<br>miRNA 3' CACGAGAGATAGAAGACAGTT 5'	2	1124-1144	Squamosa promoter-binding-like protein 12-like
vvi-miR156g-5p	VIT_211s0065g00170.4	target 5' GTGCTCTCTCTCTTCTGTCAA 3'<br>:::::::: ::::::::::<br>miRNA 3' CACGAGAGATAGAAGACAGTT 5'	2	1124-1144	Squamosa promoter-binding-like protein 12-like

miRNA	Target id	Alignment	Score	Range	Target gene functional annotation
vvi-miR156g-5p	VIT_211s0065g00170.5	target 5' GTGCTCTCTCTCTTCTGTCAA 3'<br>::::::::: :::::::::::<br>miRNA 3' CACGAGAGATAGAAGACAGTT 5'	2	1124-1144	Squamosa promoter-binding-like protein 12-like
vvi-miR156g-5p	VIT_211s0065g00170.7	target 5' GTGCTCTCTCTCTTCTGTCAA 3'<br>::::::::: :::::::::::<br>miRNA 3' CACGAGAGATAGAAGACAGTT 5'	2	1124-1144	Squamosa promoter-binding-like protein 12-like
vvi-miR156g-5p	VIT_215s0021g02290.1	target 5' GTGCTCTCTCTCTTCTGTCAT 3'<br>::::::::: ::::::::::<br>miRNA 3' CACGAGAGATAGAAGACAGTT 5'	3	797-817	Squamosa promoter-binding-like protein 7-like
vvi-miR156g-5p	VIT_217s0000g01260.1	target 5' GTGCTCTCTCTCTTCTGTCAT 3'<br>::::::::: ::::::::::<br>miRNA 3' CACGAGAGATAGAAGACAGTT 5'	3	953-973	Squamosa promoter-binding-like protein 13
vvi-miR156g-5p	VIT_217s0000g05020.1	target 5' GTGCTCTCTCTCTTCTGTCAT 3'<br>::::::::: ::::::::::<br>miRNA 3' CACGAGAGATAGAAGACAGTT 5'	3	1232-1252	Squamosa promoter-binding-like protein 6-like
vvi-miR156g-5p	VIT_217s0000g05020.2	target 5' GTGCTCTCTCTCTTCTGTCAT 3'<br>::::::::: ::::::::::<br>miRNA 3' CACGAGAGATAGAAGACAGTT 5'	3	1232-1252	Squamosa promoter-binding-like protein 6-like
vvi-miR156g-5p	VIT_217s0000g05020.3	target 5' GTGCTCTCTCTCTTCTGTCAT 3'<br>::::::::: ::::::::::<br>miRNA 3' CACGAGAGATAGAAGACAGTT 5'	3	1232-1252	Squamosa promoter-binding-like protein 6-like
vvi-miR156i	VIT_201s0010g03710.1	target 5' GTGCTCTCTCTCTTCTGTCAA 3'<br>::::::::: :::::::::::<br>miRNA 3' CACGAGAGATAGAAGACAGTT 5'	2	1145-1165	Squamosa promoter-binding-like protein 2
vvi-miR156i	VIT_201s0010g03710.2	target 5' GTGCTCTCTCTCTTCTGTCAA 3'<br>::::::::: :::::::::::<br>miRNA 3' CACGAGAGATAGAAGACAGTT 5'	2	1208-1228	Squamosa promoter-binding-like protein 2
vvi-miR156i	VIT_201s0010g03710.3	target 5' GTGCTCTCTCTCTTCTGTCAA 3'<br>::::::::: :::::::::::<br>miRNA 3' CACGAGAGATAGAAGACAGTT 5'	2	1145-1165	Squamosa promoter-binding-like protein 2
vvi-miR156i	VIT_201s0010g03710.4	target 5' GTGCTCTCTCTCTTCTGTCAA 3'<br>::::::::: :::::::::::<br>miRNA 3' CACGAGAGATAGAAGACAGTT 5'	2	1145-1165	Squamosa promoter-binding-like protein 2
vvi-miR156i	VIT_201s0010g03710.5	target 5' GTGCTCTCTCTCTTCTGTCAA 3'<br>::::::::: :::::::::::<br>miRNA 3' CACGAGAGATAGAAGACAGTT 5'	2	1208-1228	Squamosa promoter-binding-like protein 2
vvi-miR156i	VIT_201s0010g03710.6	target 5' GTGCTCTCTCTCTTCTGTCAA 3'<br>::::::::: :::::::::::<br>miRNA 3' CACGAGAGATAGAAGACAGTT 5'	2	1145-1165	Squamosa promoter-binding-like protein 2
vvi-miR156i	VIT_201s0010g03910.1	target 5' GTGCTCTCTCTCTTCTGTCAT 3'<br>::::::::: ::::::::::<br>miRNA 3' CACGAGAGATAGAAGACAGTT 5'	3	881-901	Squamosa promoter-binding-like protein 13-like

miRNA	Target id	Alignment	Score	Range	Target gene functional annotation
vvi-miR156i	VIT_201s0010g03910.2	target 5' GTGCTCTCTCTCTTCTGTCAT 3'<br>::::::::: ::::::::::<br>miRNA 3' CACGAGAGATAGAAGACAGTT 5'	3	881-901	Squamosa promoter-binding-like protein 13-like
vvi-miR156i	VIT_201s0010g03910.3	target 5' GTGCTCTCTCTCTTCTGTCAT 3'<br>::::::::: ::::::::::<br>miRNA 3' CACGAGAGATAGAAGACAGTT 5'	3	881-901	Squamosa promoter-binding-like protein 13-like
vvi-miR156i	VIT_201s0011g00130.1	target 5' GTGCTCTCTCTCTTCTGTCAG 3'<br>::::::::: ::::::::::.<br>miRNA 3' CACGAGAGATAGAAGACAGTT 5'	2.5	1250-1270	Squamosa promoter-binding-like protein 6-like
vvi-miR156i	VIT_201s0011g00130.2	target 5' GTGCTCTCTCTCTTCTGTCAG 3'<br>::::::::: ::::::::::.<br>miRNA 3' CACGAGAGATAGAAGACAGTT 5'	2.5	1250-1270	Squamosa promoter-binding-like protein 6-like
vvi-miR156i	VIT_208s0007g06270.1	target 5' GTGCTCTCTCTCTTCTGTCAA 3'<br>::::::::: :::::::::::<br>miRNA 3' CACGAGAGATAGAAGACAGTT 5'	2	788-808	Squamosa promoter-binding-like protein 9-like
vvi-miR156i	VIT_211s0065g00170.1	target 5' GTGCTCTCTCTCTTCTGTCAA 3'<br>::::::::: :::::::::::<br>miRNA 3' CACGAGAGATAGAAGACAGTT 5'	2	1124-1144	Squamosa promoter-binding-like protein 12-like
vvi-miR156i	VIT_211s0065g00170.2	target 5' GTGCTCTCTCTCTTCTGTCAA 3'<br>::::::::: :::::::::::<br>miRNA 3' CACGAGAGATAGAAGACAGTT 5'	2	1124-1144	Squamosa promoter-binding-like protein 12-like
vvi-miR156i	VIT_211s0065g00170.3	target 5' GTGCTCTCTCTCTTCTGTCAA 3'<br>::::::::: :::::::::::<br>miRNA 3' CACGAGAGATAGAAGACAGTT 5'	2	1124-1144	Squamosa promoter-binding-like protein 12-like
vvi-miR156i	VIT_211s0065g00170.4	target 5' GTGCTCTCTCTCTTCTGTCAA 3'<br>::::::::: :::::::::::<br>miRNA 3' CACGAGAGATAGAAGACAGTT 5'	2	1124-1144	Squamosa promoter-binding-like protein 12-like
vvi-miR156i	VIT_211s0065g00170.5	target 5' GTGCTCTCTCTCTTCTGTCAA 3'<br>::::::::: :::::::::::<br>miRNA 3' CACGAGAGATAGAAGACAGTT 5'	2	1124-1144	Squamosa promoter-binding-like protein 12-like
vvi-miR156i	VIT_211s0065g00170.7	target 5' GTGCTCTCTCTCTTCTGTCAA 3'<br>::::::::: :::::::::::<br>miRNA 3' CACGAGAGATAGAAGACAGTT 5'	2	1124-1144	Squamosa promoter-binding-like protein 12-like
vvi-miR156i	VIT_215s0021g02290.1	target 5' GTGCTCTCTCTCTTCTGTCAT 3'<br>::::::::: ::::::::::<br>miRNA 3' CACGAGAGATAGAAGACAGTT 5'	3	797-817	Squamosa promoter-binding-like protein 7-like
vvi-miR156i	VIT_217s0000g01260.1	target 5' GTGCTCTCTCTCTTCTGTCAT 3'<br>::::::::: ::::::::::<br>miRNA 3' CACGAGAGATAGAAGACAGTT 5'	3	953-973	Squamosa promoter-binding-like protein 13
vvi-miR156i	VIT_217s0000g05020.1	target 5' GTGCTCTCTCTCTTCTGTCAT 3'<br>::::::::: ::::::::::<br>miRNA 3' CACGAGAGATAGAAGACAGTT 5'	3	1232-1252	Squamosa promoter-binding-like protein 6-like

miRNA	Target id	Alignment	Score	Range	Target gene functional annotation
vvi-miR156i	VIT_217s0000g05020.2	target 5' GTGCTCTCTCTCTTCTGTCAT 3'<br>::::::::: ::::::::::<br>miRNA 3' CACGAGAGATAGAAGACAGTT 5'	3	1232-1252	Squamosa promoter-binding-like protein 6-like
vvi-miR156i	VIT_217s0000g05020.3	target 5' GTGCTCTCTCTCTTCTGTCAT 3'<br>::::::::: ::::::::::<br>miRNA 3' CACGAGAGATAGAAGACAGTT 5'	3	1232-1252	Squamosa promoter-binding-like protein 6-like
vvi-miR159a	VIT_200s0226g00070.1	target 5' CAGAACTCTCTTCACTCCAA 3'<br>::: :::.:::::::::::<br>miRNA 3' CTCTCGAGGGAAGTGAGGTT 5'	3	25-Jun	Gras family transcription factor
vvi-miR159a	VIT_202s0025g04120.1	target 5' GAGAGGTCCCTTCACTCCCA 3'<br>::::: :::::::::::: :<br>miRNA 3' CTCTCGAGGGAAGTGAGGTT 5'	3	1143-1162	Calmodulin binding
vvi-miR159a	VIT_203s0038g01150.1	target 5' GA-AGCACCCTTCATTCCAA 3'<br>:: ::: :::::::.:::::<br>miRNA 3' CTCTCGAGGGAAGTGAGGTT 5'	3	153-171	Auxin-induced protein 10a5
vvi-miR159a	VIT_213s0067g01630.1	target 5' TGGAGCTCCCTTCACTCCAA 3'<br>.::::::::::::::::::<br>miRNA 3' CTCTCGAGGGAAGTGAGGTT 5'	1.5	956-975	Transcription factor gamyb-like
vvi-miR159c.1-3p	VIT_219s0014g01700.1	target 5' TAGAGCCCCCTTCAAACCAAA 3'<br>:::::: :::::::: :::::<br>miRNA 3' ATCTCGAGGGAAGTTAGGTTT 5'	3	659-679	Uncharacterized protein loc100505457
vvi-miR159c.1-5p	VIT_201s0011g06470.1	target 5' CTATTGGCCTACAAGGAGCTC 3'<br>::::::: :: ::::::::::<br>miRNA 3' GATAACCTGAAGTTCCTCGAG 5'	3	336-356	Probable nad h-dependent oxidoreductase 1
vvi-miR159c.1-5p	VIT_201s0011g06470.2	target 5' CTATTGGCCTACAAGGAGCTC 3'<br>::::::: :: ::::::::::<br>miRNA 3' GATAACCTGAAGTTCCTCGAG 5'	3	336-356	Probable nad h-dependent oxidoreductase 1
vvi-miR159c.1-5p	VIT_201s0011g06470.3	target 5' CTATTGGCCTACAAGGAGCTC 3'<br>::::::: :: ::::::::::<br>miRNA 3' GATAACCTGAAGTTCCTCGAG 5'	3	336-356	Probable nad h-dependent oxidoreductase 1
vvi-miR159c.1-5p	VIT_201s0011g06470.4	target 5' CTATTGGCCTACAAGGAGCTC 3'<br>::::::: :: ::::::::::<br>miRNA 3' GATAACCTGAAGTTCCTCGAG 5'	3	336-356	Probable nad h-dependent oxidoreductase 1
vvi-miR159c.1-5p	VIT_201s0011g06470.5	target 5' CTATTGGCCTACAAGGAGCTC 3'<br>::::::: :: ::::::::::<br>miRNA 3' GATAACCTGAAGTTCCTCGAG 5'	3	336-356	Probable nad h-dependent oxidoreductase 1
vvi-miR159c.2-3p	VIT_207s0031g00480.1	target 5' GTAGCTCCTGA-ATATGCAAG 3'<br>:.::::::::: :::::::::<br>miRNA 3' CGTCGAGGACTCTATACGTTC 5'	2.5	1027-1046	Probable serine threonine-protein kinase at1g01540-like
vvi-miR160c	VIT_206s0004g02750.1	target 5' AGGCATACAGGGAGCCAGGCA 3'<br>::::::::::::::::::::<br>miRNA 3' ACCGTATGTCCCTCGGTCCGT 5'	1	1320-1340	Auxin response factor 18-like

miRNA	Target id	Alignment	Score	Range	Target gene functional annotation
vvi-miR160c	VIT_206s0004g02750.2	target 5' AGGCATACAGGGAGCCAGGCA 3'<br>:::::::::::::::::::: miRNA 3' ACCGTATGTCCCTCGGTCCGT 5'	1	1320-1340	Auxin response factor 18-like
vvi-miR160c	VIT_206s0004g02750.3	target 5' AGGCATACAGGGAGCCAGGCA 3'<br>:::::::::::::::::::: miRNA 3' ACCGTATGTCCCTCGGTCCGT 5'	1	1320-1340	Auxin response factor 18-like
vvi-miR160c	VIT_206s0004g02750.4	target 5' AGGCATACAGGGAGCCAGGCA 3'<br>:::::::::::::::::::: miRNA 3' ACCGTATGTCCCTCGGTCCGT 5'	1	1320-1340	Auxin response factor 18-like
vvi-miR160c	VIT_206s0004g02750.5	target 5' AGGCATACAGGGAGCCAGGCA 3'<br>:::::::::::::::::::: miRNA 3' ACCGTATGTCCCTCGGTCCGT 5'	1	1320-1340	Auxin response factor 18-like
vvi-miR160c	VIT_208s0040g01810.1	target 5' TGGCATGCAGGGAGCCAGGCA 3'<br>::::::.:::::::::::::: miRNA 3' ACCGTATGTCCCTCGGTCCGT 5'	0.5	1353-1373	Auxin response factor 18-like
vvi-miR160c	VIT_208s0040g01810.2	target 5' TGGCATGCAGGGAGCCAGGCA 3'<br>::::::.:::::::::::::: miRNA 3' ACCGTATGTCCCTCGGTCCGT 5'	0.5	1353-1373	Auxin response factor 18-like
vvi-miR160c	VIT_208s0040g01810.3	target 5' TGGCATGCAGGGAGCCAGGCA 3'<br>::::::.:::::::::::::: miRNA 3' ACCGTATGTCCCTCGGTCCGT 5'	0.5	1353-1373	Auxin response factor 18-like
vvi-miR160c	VIT_208s0040g01810.4	target 5' TGGCATGCAGGGAGCCAGGCA 3'<br>::::::.:::::::::::::: miRNA 3' ACCGTATGTCCCTCGGTCCGT 5'	0.5	1353-1373	Auxin response factor 18-like
vvi-miR160c	VIT_213s0019g04380.1	target 5' AGGCATACAGGGAGCCAGGCA 3'<br>:::::::::::::::::::: miRNA 3' ACCGTATGTCCCTCGGTCCGT 5'	1	1326-1346	Protein
vvi-miR160c	VIT_213s0019g04380.2	target 5' AGGCATACAGGGAGCCAGGCA 3'<br>:::::::::::::::::::: miRNA 3' ACCGTATGTCCCTCGGTCCGT 5'	1	1326-1346	Protein
vvi-miR160c	VIT_213s0019g04380.3	target 5' AGGCATACAGGGAGCCAGGCA 3'<br>:::::::::::::::::::: miRNA 3' ACCGTATGTCCCTCGGTCCGT 5'	1	1326-1346	Protein
vvi-miR160c	VIT_213s0019g04380.4	target 5' AGGCATACAGGGAGCCAGGCA 3'<br>:::::::::::::::::::: miRNA 3' ACCGTATGTCCCTCGGTCCGT 5'	1	1326-1346	Protein
vvi-miR160c	VIT_218s0001g04180.1	target 5' TGGCATGCAGGGAGCCAGGCA 3'<br>::::::.:::::::::::::: miRNA 3' ACCGTATGTCCCTCGGTCCGT 5'	0.5	1293-1313	Auxin response
vvi-miR160c	VIT_218s0001g04180.2	target 5' TGGCATGCAGGGAGCCAGGCA 3'<br>::::::.:::::::::::::: miRNA 3' ACCGTATGTCCCTCGGTCCGT 5'	0.5	1293-1313	Auxin response

miRNA	Target id	Alignment	Score	Range	Target gene functional annotation
vvi-miR160d	VIT_206s0004g02750.1	target 5' AGGCATACAGGGAGCCAGGCA 3'<br>::::::::::::::::::::<br>miRNA 3' ACCGTATGTCCCTCGGTCCGT 5'	1	1320-1340	Auxin response factor 18-like
vvi-miR160d	VIT_206s0004g02750.2	target 5' AGGCATACAGGGAGCCAGGCA 3'<br>::::::::::::::::::::<br>miRNA 3' ACCGTATGTCCCTCGGTCCGT 5'	1	1320-1340	Auxin response factor 18-like
vvi-miR160d	VIT_206s0004g02750.3	target 5' AGGCATACAGGGAGCCAGGCA 3'<br>::::::::::::::::::::<br>miRNA 3' ACCGTATGTCCCTCGGTCCGT 5'	1	1320-1340	Auxin response factor 18-like
vvi-miR160d	VIT_206s0004g02750.4	target 5' AGGCATACAGGGAGCCAGGCA 3'<br>::::::::::::::::::::<br>miRNA 3' ACCGTATGTCCCTCGGTCCGT 5'	1	1320-1340	Auxin response factor 18-like
vvi-miR160d	VIT_206s0004g02750.5	target 5' AGGCATACAGGGAGCCAGGCA 3'<br>::::::::::::::::::::<br>miRNA 3' ACCGTATGTCCCTCGGTCCGT 5'	1	1320-1340	Auxin response factor 18-like
vvi-miR160d	VIT_208s0040g01810.1	target 5' TGGCATGCAGGGAGCCAGGCA 3'<br>::::::.::::::::::::::<br>miRNA 3' ACCGTATGTCCCTCGGTCCGT 5'	0.5	1353-1373	Auxin response factor 18-like
vvi-miR160d	VIT_208s0040g01810.2	target 5' TGGCATGCAGGGAGCCAGGCA 3'<br>::::::.::::::::::::::<br>miRNA 3' ACCGTATGTCCCTCGGTCCGT 5'	0.5	1353-1373	Auxin response factor 18-like
vvi-miR160d	VIT_208s0040g01810.3	target 5' TGGCATGCAGGGAGCCAGGCA 3'<br>::::::.::::::::::::::<br>miRNA 3' ACCGTATGTCCCTCGGTCCGT 5'	0.5	1353-1373	Auxin response factor 18-like
vvi-miR160d	VIT_208s0040g01810.4	target 5' TGGCATGCAGGGAGCCAGGCA 3'<br>::::::.::::::::::::::<br>miRNA 3' ACCGTATGTCCCTCGGTCCGT 5'	0.5	1353-1373	Auxin response factor 18-like
vvi-miR160d	VIT_213s0019g04380.1	target 5' AGGCATACAGGGAGCCAGGCA 3'<br>::::::::::::::::::::<br>miRNA 3' ACCGTATGTCCCTCGGTCCGT 5'	1	1326-1346	Protein
vvi-miR160d	VIT_213s0019g04380.2	target 5' AGGCATACAGGGAGCCAGGCA 3'<br>::::::::::::::::::::<br>miRNA 3' ACCGTATGTCCCTCGGTCCGT 5'	1	1326-1346	Protein
vvi-miR160d	VIT_213s0019g04380.3	target 5' AGGCATACAGGGAGCCAGGCA 3'<br>::::::::::::::::::::<br>miRNA 3' ACCGTATGTCCCTCGGTCCGT 5'	1	1326-1346	Protein
vvi-miR160d	VIT_213s0019g04380.4	target 5' AGGCATACAGGGAGCCAGGCA 3'<br>::::::::::::::::::::<br>miRNA 3' ACCGTATGTCCCTCGGTCCGT 5'	1	1326-1346	Protein
vvi-miR160d	VIT_218s0001g04180.1	target 5' TGGCATGCAGGGAGCCAGGCA 3'<br>::::::.::::::::::::::<br>miRNA 3' ACCGTATGTCCCTCGGTCCGT 5'	0.5	1293-1313	Auxin response

miRNA	Target id	Alignment	Score	Range	Target gene functional annotation
vvi-miR160d	VIT_218s0001g04180.2	target 5' TGGCATGCAGGGAGCCAGGCA 3'<br>::::::.::::::::::::::<br>miRNA 3' ACCGTATGTCCCTCGGTCCGT 5'	0.5	1293-1313	Auxin response
vvi-miR160e	VIT_206s0004g02750.1	target 5' AGGCATACAGGGAGCCAGGCA 3'<br>::::::::::::::::::::<br>miRNA 3' ACCGTATGTCCCTCGGTCCGT 5'	1	1320-1340	Auxin response factor 18-like
vvi-miR160e	VIT_206s0004g02750.2	target 5' AGGCATACAGGGAGCCAGGCA 3'<br>::::::::::::::::::::<br>miRNA 3' ACCGTATGTCCCTCGGTCCGT 5'	1	1320-1340	Auxin response factor 18-like
vvi-miR160e	VIT_206s0004g02750.3	target 5' AGGCATACAGGGAGCCAGGCA 3'<br>::::::::::::::::::::<br>miRNA 3' ACCGTATGTCCCTCGGTCCGT 5'	1	1320-1340	Auxin response factor 18-like
vvi-miR160e	VIT_206s0004g02750.4	target 5' AGGCATACAGGGAGCCAGGCA 3'<br>::::::::::::::::::::<br>miRNA 3' ACCGTATGTCCCTCGGTCCGT 5'	1	1320-1340	Auxin response factor 18-like
vvi-miR160e	VIT_206s0004g02750.5	target 5' AGGCATACAGGGAGCCAGGCA 3'<br>::::::::::::::::::::<br>miRNA 3' ACCGTATGTCCCTCGGTCCGT 5'	1	1320-1340	Auxin response factor 18-like
vvi-miR160e	VIT_208s0040g01810.1	target 5' TGGCATGCAGGGAGCCAGGCA 3'<br>::::::.::::::::::::::<br>miRNA 3' ACCGTATGTCCCTCGGTCCGT 5'	0.5	1353-1373	Auxin response factor 18-like
vvi-miR160e	VIT_208s0040g01810.2	target 5' TGGCATGCAGGGAGCCAGGCA 3'<br>::::::.::::::::::::::<br>miRNA 3' ACCGTATGTCCCTCGGTCCGT 5'	0.5	1353-1373	Auxin response factor 18-like
vvi-miR160e	VIT_208s0040g01810.3	target 5' TGGCATGCAGGGAGCCAGGCA 3'<br>::::::.::::::::::::::<br>miRNA 3' ACCGTATGTCCCTCGGTCCGT 5'	0.5	1353-1373	Auxin response factor 18-like
vvi-miR160e	VIT_208s0040g01810.4	target 5' TGGCATGCAGGGAGCCAGGCA 3'<br>::::::.::::::::::::::<br>miRNA 3' ACCGTATGTCCCTCGGTCCGT 5'	0.5	1353-1373	Auxin response factor 18-like
vvi-miR160e	VIT_213s0019g04380.1	target 5' AGGCATACAGGGAGCCAGGCA 3'<br>::::::::::::::::::::<br>miRNA 3' ACCGTATGTCCCTCGGTCCGT 5'	1	1326-1346	Protein
vvi-miR160e	VIT_213s0019g04380.2	target 5' AGGCATACAGGGAGCCAGGCA 3'<br>::::::::::::::::::::<br>miRNA 3' ACCGTATGTCCCTCGGTCCGT 5'	1	1326-1346	Protein
vvi-miR160e	VIT_213s0019g04380.3	target 5' AGGCATACAGGGAGCCAGGCA 3'<br>::::::::::::::::::::<br>miRNA 3' ACCGTATGTCCCTCGGTCCGT 5'	1	1326-1346	Protein
vvi-miR160e	VIT_213s0019g04380.4	target 5' AGGCATACAGGGAGCCAGGCA 3'<br>::::::::::::::::::::<br>miRNA 3' ACCGTATGTCCCTCGGTCCGT 5'	1	1326-1346	Protein

miRNA	Target id	Alignment	Score	Range	Target gene functional annotation
vvi-miR160e	VIT_218s0001g04180.1	target 5' TGGCATGCAGGGAGCCAGGCA 3'<br>::::::.::::::::::::::<br>miRNA 3' ACCGTATGTCCCTCGGTCCGT 5'	0.5	1293-1313	Auxin response
vvi-miR160e	VIT_218s0001g04180.2	target 5' TGGCATGCAGGGAGCCAGGCA 3'<br>::::::.::::::::::::::<br>miRNA 3' ACCGTATGTCCCTCGGTCCGT 5'	0.5	1293-1313	Auxin response
vvi-miR162-3p	VIT_206s0009g03510.1	target 5' TTGGGT-CAGAGGTTTATTGA 3'<br>.:::.: :::::::::::.::<br>miRNA 3' GACCTACGTCTCCAAATAGCT 5'	3	337-356	Pentatricopeptide repeat-containing protein
vvi-miR162-3p	VIT_215s0048g02380.1	target 5' CTGGATGCAGAGGTGTTATCGA 3'<br>:::::::::::::: :::::::<br>miRNA 3' GACCTACGTCTCCA-AATAGCT 5'	2	3250-3271	Endoribonuclease dicer homolog 1-like
vvi-miR162-3p	VIT_215s0048g02380.2	target 5' CTGGATGCAGAGGTGTTATCGA 3'<br>:::::::::::::: :::::::<br>miRNA 3' GACCTACGTCTCCA-AATAGCT 5'	2	3250-3271	Endoribonuclease dicer homolog 1-like
vvi-miR162-3p	VIT_215s0048g02380.3	target 5' CTGGATGCAGAGGTGTTATCGA 3'<br>:::::::::::::: :::::::<br>miRNA 3' GACCTACGTCTCCA-AATAGCT 5'	2	2005-2026	Endoribonuclease dicer homolog 1-like
vvi-miR164a-5p	VIT_217s0000g06400.1	target 5' CTCACGTGCCCTGCTTCTCCA 3'<br>:::::::::::::::::::<br>miRNA 3' ACGTGCACGGGACGAAGAGGT 5'	2	644-664	Nac domain ipr003441
vvi-miR164a-5p	VIT_217s0000g06400.2	target 5' CTCACGTGCCCTGCTTCTCCA 3'<br>:::::::::::::::::::<br>miRNA 3' ACGTGCACGGGACGAAGAGGT 5'	2	740-760	Nac domain ipr003441
vvi-miR164a-5p	VIT_217s0000g06400.3	target 5' CTCACGTGCCCTGCTTCTCCA 3'<br>:::::::::::::::::::<br>miRNA 3' ACGTGCACGGGACGAAGAGGT 5'	2	467-487	Nac domain ipr003441
vvi-miR164a-5p	VIT_219s0014g02200.1	target 5' AGCACGTGCCCTGGTTCTCCA 3'<br>:::::::::::: :::::::<br>miRNA 3' ACGTGCACGGGACGAAGAGGT 5'	3	722-742	No apical meristem
vvi-miR164a-5p	VIT_219s0027g00230.1	target 5' AGCAAGTGCCCTGCTTCTCCG 3'<br>::: :::::::::::::::.<br>miRNA 3' ACGTGCACGGGACGAAGAGGT 5'	2.5	623-643	Nac domain protein
vvi-miR164a-5p	VIT_219s0027g00230.2	target 5' AGCAAGTGCCCTGCTTCTCCG 3'<br>::: :::::::::::::::.<br>miRNA 3' ACGTGCACGGGACGAAGAGGT 5'	2.5	488-508	Nac domain protein
vvi-miR164c-3p	VIT_214s0036g01400.1	target 5' GAAGGAGAAGAGGGGCACATC 3'<br>:: :: ::::::::::::::<br>miRNA 3' CTACCCCTTCTCCCCGTGTAC 5'	3	36-56	Protein
vvi-miR164c-5p	VIT_217s0000g06400.1	target 5' CTCACGTGCCCTGCTTCTCCA 3'<br>:::::::::::::::::::<br>miRNA 3' ACGTGCACGGGACGAAGAGGT 5'	2	644-664	Nac domain ipr003441

miRNA	Target id	Alignment	Score	Range	Target gene functional annotation
vvi-miR164c-5p	VIT_217s0000g06400.2	target 5' CTCACGTGCCCTGCTTCTCCA 3'<br>::::::::::::::::::: miRNA 3' ACGTGCACGGGACGAAGAGGT 5'	2	740-760	Nac domain ipr003441
vvi-miR164c-5p	VIT_217s0000g06400.3	target 5' CTCACGTGCCCTGCTTCTCCA 3'<br>:::::::::::::::::::<br>miRNA 3' ACGTGCACGGGACGAAGAGGT 5'	2	467-487	Nac domain ipr003441
vvi-miR164c-5p	VIT_219s0014g02200.1	target 5' AGCACGTGCCCTGGTTCTCCA 3'<br>:::::::::::: :::::::<br>miRNA 3' ACGTGCACGGGACGAAGAGGT 5'	3	722-742	No apical meristem
vvi-miR164c-5p	VIT_219s0027g00230.1	target 5' AGCAAGTGCCCTGCTTCTCCG 3'<br>::: :::::::::::::::.<br>miRNA 3' ACGTGCACGGGACGAAGAGGT 5'	2.5	623-643	Nac domain protein
vvi-miR164c-5p	VIT_219s0027g00230.2	target 5' AGCAAGTGCCCTGCTTCTCCG 3'<br>::: :::::::::::::::.<br>miRNA 3' ACGTGCACGGGACGAAGAGGT 5'	2.5	488-508	Nac domain protein
vvi-miR164d-5p	VIT_217s0000g06400.1	target 5' CTCACGTGCCCTGCTTCTCCA 3'<br>:::::::::::::::::::<br>miRNA 3' ACGTGCACGGGACGAAGAGGT 5'	2	644-664	Nac domain ipr003441
vvi-miR164d-5p	VIT_217s0000g06400.2	target 5' CTCACGTGCCCTGCTTCTCCA 3'<br>:::::::::::::::::::<br>miRNA 3' ACGTGCACGGGACGAAGAGGT 5'	2	740-760	Nac domain ipr003441
vvi-miR164d-5p	VIT_217s0000g06400.3	target 5' CTCACGTGCCCTGCTTCTCCA 3'<br>:::::::::::::::::::<br>miRNA 3' ACGTGCACGGGACGAAGAGGT 5'	2	467-487	Nac domain ipr003441
vvi-miR164d-5p	VIT_219s0014g02200.1	target 5' AGCACGTGCCCTGGTTCTCCA 3'<br>:::::::::::: :::::::<br>miRNA 3' ACGTGCACGGGACGAAGAGGT 5'	3	722-742	No apical meristem
vvi-miR164d-5p	VIT_219s0027g00230.1	target 5' AGCAAGTGCCCTGCTTCTCCG 3'<br>::: :::::::::::::::.<br>miRNA 3' ACGTGCACGGGACGAAGAGGT 5'	2.5	623-643	Nac domain protein
vvi-miR164d-5p	VIT_219s0027g00230.2	target 5' AGCAAGTGCCCTGCTTCTCCG 3'<br>::: :::::::::::::::.<br>miRNA 3' ACGTGCACGGGACGAAGAGGT 5'	2.5	488-508	Nac domain protein
vvi-miR166a-3p	VIT_218s0075g00480.1	target 5' GGAATGAAGTATGGTCCGAGA 3'<br>:::::::::. ::::::::::<br>miRNA 3' CCTTACTTCGGACCAGGCTCT 5'	3	2612-2632	Tmv resistance protein n-like
vvi-miR166a-3p	VIT_218s0075g00480.2	target 5' GGAATGAAGTATGGTCCGAGA 3'<br>:::::::::. ::::::::::<br>miRNA 3' CCTTACTTCGGACCAGGCTCT 5'	3	2612-2632	Tmv resistance protein n-like
vvi-miR166a-3p	VIT_218s0075g00480.3	target 5' GGAATGAAGTATGGTCCGAGA 3'<br>:::::::::. ::::::::::<br>miRNA 3' CCTTACTTCGGACCAGGCTCT 5'	3	1988-2008	Tmv resistance protein n-like

miRNA	Target id	Alignment	Score	Range	Target gene functional annotation
vvi-miR166a-3p	VIT_218s0075g00480.4	target 5' GGAATGAAGTATGGTCCGAGA 3'<br>:::::::::. ::::::::::<br>miRNA 3' CCTTACTTCGGACCAGGCTCT 5'	3	950-970	Tmv resistance protein n-like
vvi-miR166a-3p	VIT_218s0075g00480.5	target 5' GGAATGAAGTATGGTCCGAGA 3'<br>:::::::::. ::::::::::<br>miRNA 3' CCTTACTTCGGACCAGGCTCT 5'	3	2612-2632	Tmv resistance protein n-like
vvi-miR166a-3p	VIT_218s0075g00480.6	target 5' GGAATGAAGTATGGTCCGAGA 3'<br>:::::::::. ::::::::::<br>miRNA 3' CCTTACTTCGGACCAGGCTCT 5'	3	479-499	Tmv resistance protein n-like
vvi-miR166a-3p	VIT_218s0075g00480.7	target 5' GGAATGAAGTATGGTCCGAGA 3'<br>:::::::::. ::::::::::<br>miRNA 3' CCTTACTTCGGACCAGGCTCT 5'	3	950-970	Tmv resistance protein n-like
vvi-miR166b-3p	VIT_204s0008g03250.1	target 5' CTGGGATGAAGCCTGGTCCGG 3'<br>::.:::::::::::::::.<br>miRNA 3' CTCCTTACTTCGGACCAGGCT 5'	3	557-577	Protein
vvi-miR166b-3p	VIT_204s0008g03250.2	target 5' CTGGGATGAAGCCTGGTCCGG 3'<br>::.:::::::::::::::.<br>miRNA 3' CTCCTTACTTCGGACCAGGCT 5'	3	557-577	Class iii hd-zip protein 8
vvi-miR166b-3p	VIT_206s0004g02800.1	target 5' CTGGGATGAAGCCTGGTCCGG 3'<br>::.:::::::::::::::.<br>miRNA 3' CTCCTTACTTCGGACCAGGCT 5'	3	563-583	Homeobox leucine-zipper protein
vvi-miR166b-3p	VIT_206s0004g02800.2	target 5' CTGGGATGAAGCCTGGTCCGG 3'<br>::.:::::::::::::::.<br>miRNA 3' CTCCTTACTTCGGACCAGGCT 5'	3	563-583	Homeobox leucine-zipper protein
vvi-miR166b-3p	VIT_206s0004g02800.3	target 5' CTGGGATGAAGCCTGGTCCGG 3'<br>::.:::::::::::::::.<br>miRNA 3' CTCCTTACTTCGGACCAGGCT 5'	3	563-583	Homeobox leucine-zipper protein
vvi-miR166b-3p	VIT_209s0002g03740.1	target 5' CTGGAATGAAGCCTGGTCCGG 3'<br>::::::::::::::::::.<br>miRNA 3' CTCCTTACTTCGGACCAGGCT 5'	2.5	551-571	Homeobox-leucine zipper protein athb-15
vvi-miR166b-3p	VIT_210s0003g04670.1	target 5' TTGGGATGAAGCCTGGTCCGG 3'<br>::.:::::::::::::::.<br>miRNA 3' CTCCTTACTTCGGACCAGGCT 5'	3	566-586	Homeobox-leucine zipper protein hox32-like
vvi-miR166b-3p	VIT_210s0003g04670.2	target 5' TTGGGATGAAGCCTGGTCCGG 3'<br>::.:::::::::::::::.<br>miRNA 3' CTCCTTACTTCGGACCAGGCT 5'	3	293-313	Homeobox-leucine zipper protein hox32-like
vvi-miR166b-3p	VIT_210s0003g04670.3	target 5' TTGGGATGAAGCCTGGTCCGG 3'<br>::.:::::::::::::::.<br>miRNA 3' CTCCTTACTTCGGACCAGGCT 5'	3	25-May	Homeobox-leucine zipper protein hox32-like
vvi-miR166b-3p	VIT_213s0019g04320.1	target 5' CTGGGATGAAGCCTGGTCCGG 3'<br>::.:::::::::::::::.<br>miRNA 3' CTCCTTACTTCGGACCAGGCT 5'	3	263-283	Homeobox-leucine zipper protein revoluta

miRNA	Target id	Alignment	Score	Range	Target gene functional annotation
vvi-miR166b-3p	VIT_213s0019g04320.2	target 5' CTGGGATGAAGCCTGGTCCGG 3'<br>::.:::::::::::::::.<br>miRNA 3' CTCCTTACTTCGGACCAGGCT 5'	3	263-283	Homeobox-leucine zipper protein revoluta
vvi-miR166b-3p	VIT_213s0019g04320.3	target 5' CTGGGATGAAGCCTGGTCCGG 3'<br>::.:::::::::::::::.<br>miRNA 3' CTCCTTACTTCGGACCAGGCT 5'	3	263-283	Homeobox-leucine zipper protein revoluta
vvi-miR166b-3p	VIT_213s0019g04320.4	target 5' CTGGGATGAAGCCTGGTCCGG 3'<br>::.:::::::::::::::.<br>miRNA 3' CTCCTTACTTCGGACCAGGCT 5'	3	263-283	Homeobox-leucine zipper protein revoluta
vvi-miR166b-3p	VIT_213s0019g04320.5	target 5' CTGGGATGAAGCCTGGTCCGG 3'<br>::.:::::::::::::::.<br>miRNA 3' CTCCTTACTTCGGACCAGGCT 5'	3	173-193	Homeobox-leucine zipper protein revoluta
vvi-miR166b-3p	VIT_213s0019g04320.6	target 5' CTGGGATGAAGCCTGGTCCGG 3'<br>::.:::::::::::::::.<br>miRNA 3' CTCCTTACTTCGGACCAGGCT 5'	3	263-283	Homeobox-leucine zipper protein revoluta
vvi-miR166b-3p	VIT_213s0019g04320.7	target 5' CTGGGATGAAGCCTGGTCCGG 3'<br>::.:::::::::::::::.<br>miRNA 3' CTCCTTACTTCGGACCAGGCT 5'	3	263-283	Homeobox-leucine zipper protein revoluta
vvi-miR166c-3p	VIT_204s0008g03250.1	target 5' CTGGGATGAAGCCTGGTCCGG 3'<br>::.:::::::::::::::.<br>miRNA 3' CCCCTTACTTCGGACCAGGCT 5'	3	557-577	Protein
vvi-miR166c-3p	VIT_204s0008g03250.2	target 5' CTGGGATGAAGCCTGGTCCGG 3'<br>::.:::::::::::::::.<br>miRNA 3' CCCCTTACTTCGGACCAGGCT 5'	3	557-577	Class iii hd-zip protein 8
vvi-miR166c-3p	VIT_206s0004g02800.1	target 5' CTGGGATGAAGCCTGGTCCGG 3'<br>::.:::::::::::::::.<br>miRNA 3' CCCCTTACTTCGGACCAGGCT 5'	3	563-583	Homeobox leucine-zipper protein
vvi-miR166c-3p	VIT_206s0004g02800.2	target 5' CTGGGATGAAGCCTGGTCCGG 3'<br>::.:::::::::::::::.<br>miRNA 3' CCCCTTACTTCGGACCAGGCT 5'	3	563-583	Homeobox leucine-zipper protein
vvi-miR166c-3p	VIT_206s0004g02800.3	target 5' CTGGGATGAAGCCTGGTCCGG 3'<br>::.:::::::::::::::.<br>miRNA 3' CCCCTTACTTCGGACCAGGCT 5'	3	563-583	Homeobox leucine-zipper protein
vvi-miR166c-3p	VIT_209s0002g03740.1	target 5' CTGGAATGAAGCCTGGTCCGG 3'<br>::::::::::::::::::.<br>miRNA 3' CCCCTTACTTCGGACCAGGCT 5'	2.5	551-571	Homeobox-leucine zipper protein athb-15
vvi-miR166c-3p	VIT_210s0003g04670.1	target 5' TTGGGATGAAGCCTGGTCCGG 3'<br>::.:::::::::::::::.<br>miRNA 3' CCCCTTACTTCGGACCAGGCT 5'	3	566-586	Homeobox-leucine zipper protein hox32-like
vvi-miR166c-3p	VIT_210s0003g04670.2	target 5' TTGGGATGAAGCCTGGTCCGG 3'<br>::.:::::::::::::::.<br>miRNA 3' CCCCTTACTTCGGACCAGGCT 5'	3	293-313	Homeobox-leucine zipper protein hox32-like

miRNA	Target id	Alignment	Score	Range	Target gene functional annotation
vvi-miR166c-3p	VIT_210s0003g04670.3	target 5' TTGGGATGAAGCCTGGTCCGG 3'<br>::.:::::::::::::::::.<br>miRNA 3' CCCCTTACTTCGGACCAGGCT 5'	3	25-May	Homeobox-leucine zipper protein hox32-like
vvi-miR166c-3p	VIT_213s0019g04320.1	target 5' CTGGGATGAAGCCTGGTCCGG 3'<br>::.:::::::::::::::::.<br>miRNA 3' CCCCTTACTTCGGACCAGGCT 5'	3	263-283	Homeobox-leucine zipper protein revoluta
vvi-miR166c-3p	VIT_213s0019g04320.2	target 5' CTGGGATGAAGCCTGGTCCGG 3'<br>::.:::::::::::::::::.<br>miRNA 3' CCCCTTACTTCGGACCAGGCT 5'	3	263-283	Homeobox-leucine zipper protein revoluta
vvi-miR166c-3p	VIT_213s0019g04320.3	target 5' CTGGGATGAAGCCTGGTCCGG 3'<br>::.:::::::::::::::::.<br>miRNA 3' CCCCTTACTTCGGACCAGGCT 5'	3	263-283	Homeobox-leucine zipper protein revoluta
vvi-miR166c-3p	VIT_213s0019g04320.4	target 5' CTGGGATGAAGCCTGGTCCGG 3'<br>::.:::::::::::::::::.<br>miRNA 3' CCCCTTACTTCGGACCAGGCT 5'	3	263-283	Homeobox-leucine zipper protein revoluta
vvi-miR166c-3p	VIT_213s0019g04320.5	target 5' CTGGGATGAAGCCTGGTCCGG 3'<br>::.:::::::::::::::::.<br>miRNA 3' CCCCTTACTTCGGACCAGGCT 5'	3	173-193	Homeobox-leucine zipper protein revoluta
vvi-miR166c-3p	VIT_213s0019g04320.6	target 5' CTGGGATGAAGCCTGGTCCGG 3'<br>::.:::::::::::::::::.<br>miRNA 3' CCCCTTACTTCGGACCAGGCT 5'	3	263-283	Homeobox-leucine zipper protein revoluta
vvi-miR166c-3p	VIT_213s0019g04320.7	target 5' CTGGGATGAAGCCTGGTCCGG 3'<br>::.:::::::::::::::::.<br>miRNA 3' CCCCTTACTTCGGACCAGGCT 5'	3	263-283	Homeobox-leucine zipper protein revoluta
vvi-miR166d-3p	VIT_204s0008g03250.1	target 5' CTGGGATGAAGCCTGGTCCGG 3'<br>::.:::::::::::::::::.<br>miRNA 3' CCCCTTACTTCGGACCAGGCT 5'	3	557-577	Protein
vvi-miR166d-3p	VIT_204s0008g03250.2	target 5' CTGGGATGAAGCCTGGTCCGG 3'<br>::.:::::::::::::::::.<br>miRNA 3' CCCCTTACTTCGGACCAGGCT 5'	3	557-577	Class iii hd-zip protein 8
vvi-miR166d-3p	VIT_206s0004g02800.1	target 5' CTGGGATGAAGCCTGGTCCGG 3'<br>::.:::::::::::::::::.<br>miRNA 3' CCCCTTACTTCGGACCAGGCT 5'	3	563-583	Homeobox leucine-zipper protein
vvi-miR166d-3p	VIT_206s0004g02800.2	target 5' CTGGGATGAAGCCTGGTCCGG 3'<br>::.:::::::::::::::::.<br>miRNA 3' CCCCTTACTTCGGACCAGGCT 5'	3	563-583	Homeobox leucine-zipper protein
vvi-miR166d-3p	VIT_206s0004g02800.3	target 5' CTGGGATGAAGCCTGGTCCGG 3'<br>::.:::::::::::::::::.<br>miRNA 3' CCCCTTACTTCGGACCAGGCT 5'	3	563-583	Homeobox leucine-zipper protein
vvi-miR166d-3p	VIT_209s0002g03740.1	target 5' CTGGAATGAAGCCTGGTCCGG 3'<br>:::::::::::::::::::.<br>miRNA 3' CCCCTTACTTCGGACCAGGCT 5'	2.5	551-571	Homeobox-leucine zipper protein athb-15

miRNA	Target id	Alignment	Score	Range	Target gene functional annotation
vvi-miR166d-3p	VIT_210s0003g04670.1	target 5' TTGGGATGAAGCCTGGTCCGG 3'<br>::.:::::::::::::::::.<br>miRNA 3' CCCCTTACTTCGGACCAGGCT 5'	3	566-586	Homeobox-leucine zipper protein hox32-like
vvi-miR166d-3p	VIT_210s0003g04670.2	target 5' TTGGGATGAAGCCTGGTCCGG 3'<br>::.:::::::::::::::::.<br>miRNA 3' CCCCTTACTTCGGACCAGGCT 5'	3	293-313	Homeobox-leucine zipper protein hox32-like
vvi-miR166d-3p	VIT_210s0003g04670.3	target 5' TTGGGATGAAGCCTGGTCCGG 3'<br>::.:::::::::::::::::.<br>miRNA 3' CCCCTTACTTCGGACCAGGCT 5'	3	25-May	Homeobox-leucine zipper protein hox32-like
vvi-miR166d-3p	VIT_213s0019g04320.1	target 5' CTGGGATGAAGCCTGGTCCGG 3'<br>::.:::::::::::::::::.<br>miRNA 3' CCCCTTACTTCGGACCAGGCT 5'	3	263-283	Homeobox-leucine zipper protein revoluta
vvi-miR166d-3p	VIT_213s0019g04320.2	target 5' CTGGGATGAAGCCTGGTCCGG 3'<br>::.:::::::::::::::::.<br>miRNA 3' CCCCTTACTTCGGACCAGGCT 5'	3	263-283	Homeobox-leucine zipper protein revoluta
vvi-miR166d-3p	VIT_213s0019g04320.3	target 5' CTGGGATGAAGCCTGGTCCGG 3'<br>::.:::::::::::::::::.<br>miRNA 3' CCCCTTACTTCGGACCAGGCT 5'	3	263-283	Homeobox-leucine zipper protein revoluta
vvi-miR166d-3p	VIT_213s0019g04320.4	target 5' CTGGGATGAAGCCTGGTCCGG 3'<br>::.:::::::::::::::::.<br>miRNA 3' CCCCTTACTTCGGACCAGGCT 5'	3	263-283	Homeobox-leucine zipper protein revoluta
vvi-miR166d-3p	VIT_213s0019g04320.5	target 5' CTGGGATGAAGCCTGGTCCGG 3'<br>::.:::::::::::::::::.<br>miRNA 3' CCCCTTACTTCGGACCAGGCT 5'	3	173-193	Homeobox-leucine zipper protein revoluta
vvi-miR166d-3p	VIT_213s0019g04320.6	target 5' CTGGGATGAAGCCTGGTCCGG 3'<br>::.:::::::::::::::::.<br>miRNA 3' CCCCTTACTTCGGACCAGGCT 5'	3	263-283	Homeobox-leucine zipper protein revoluta
vvi-miR166d-3p	VIT_213s0019g04320.7	target 5' CTGGGATGAAGCCTGGTCCGG 3'<br>::.:::::::::::::::::.<br>miRNA 3' CCCCTTACTTCGGACCAGGCT 5'	3	263-283	Homeobox-leucine zipper protein revoluta
vvi-miR166d-5p	VIT_208s0056g01350.1	target 5' GCCTCAGACCAGACAACAATC 3'<br>::::: . :::::::::::::<br>miRNA 3' CGGAGCTCGGTCTGTTGTTAG 5'	2.5	1750-1770	Protein
vvi-miR166e-3p	VIT_204s0008g03250.1	target 5' CTGGGATGAAGCCTGGTCCGG 3'<br>::.:::::::::::::::::.<br>miRNA 3' CCCCTTACTTCGGACCAGGCT 5'	3	557-577	Protein
vvi-miR166e-3p	VIT_204s0008g03250.2	target 5' CTGGGATGAAGCCTGGTCCGG 3'<br>::.:::::::::::::::::.<br>miRNA 3' CCCCTTACTTCGGACCAGGCT 5'	3	557-577	Class iii hd-zip protein 8
vvi-miR166e-3p	VIT_206s0004g02800.1	target 5' CTGGGATGAAGCCTGGTCCGG 3'<br>::.:::::::::::::::::.<br>miRNA 3' CCCCTTACTTCGGACCAGGCT 5'	3	563-583	Homeobox leucine-zipper protein

miRNA	Target id	Alignment	Score	Range	Target gene functional annotation
vvi-miR166e-3p	VIT_206s0004g02800.2	target 5' CTGGGATGAAGCCTGGTCCGG 3'<br>::.:::::::::::::::.<br>miRNA 3' CCCCTTACTTCGGACCAGGCT 5'	3	563-583	Homeobox leucine-zipper protein
vvi-miR166e-3p	VIT_206s0004g02800.3	target 5' CTGGGATGAAGCCTGGTCCGG 3'<br>::.:::::::::::::::.<br>miRNA 3' CCCCTTACTTCGGACCAGGCT 5'	3	563-583	Homeobox leucine-zipper protein
vvi-miR166e-3p	VIT_209s0002g03740.1	target 5' CTGGAATGAAGCCTGGTCCGG 3'<br>::::::::::::::::::.<br>miRNA 3' CCCCTTACTTCGGACCAGGCT 5'	2.5	551-571	Homeobox-leucine zipper protein athb-15
vvi-miR166e-3p	VIT_210s0003g04670.1	target 5' TTGGGATGAAGCCTGGTCCGG 3'<br>::.:::::::::::::::.<br>miRNA 3' CCCCTTACTTCGGACCAGGCT 5'	3	566-586	Homeobox-leucine zipper protein hox32-like
vvi-miR166e-3p	VIT_210s0003g04670.2	target 5' TTGGGATGAAGCCTGGTCCGG 3'<br>::.:::::::::::::::.<br>miRNA 3' CCCCTTACTTCGGACCAGGCT 5'	3	293-313	Homeobox-leucine zipper protein hox32-like
vvi-miR166e-3p	VIT_210s0003g04670.3	target 5' TTGGGATGAAGCCTGGTCCGG 3'<br>::.:::::::::::::::.<br>miRNA 3' CCCCTTACTTCGGACCAGGCT 5'	3	25-May	Homeobox-leucine zipper protein hox32-like
vvi-miR166e-3p	VIT_213s0019g04320.1	target 5' CTGGGATGAAGCCTGGTCCGG 3'<br>::.:::::::::::::::.<br>miRNA 3' CCCCTTACTTCGGACCAGGCT 5'	3	263-283	Homeobox-leucine zipper protein revoluta
vvi-miR166e-3p	VIT_213s0019g04320.2	target 5' CTGGGATGAAGCCTGGTCCGG 3'<br>::.:::::::::::::::.<br>miRNA 3' CCCCTTACTTCGGACCAGGCT 5'	3	263-283	Homeobox-leucine zipper protein revoluta
vvi-miR166e-3p	VIT_213s0019g04320.3	target 5' CTGGGATGAAGCCTGGTCCGG 3'<br>::.:::::::::::::::.<br>miRNA 3' CCCCTTACTTCGGACCAGGCT 5'	3	263-283	Homeobox-leucine zipper protein revoluta
vvi-miR166e-3p	VIT_213s0019g04320.4	target 5' CTGGGATGAAGCCTGGTCCGG 3'<br>::.:::::::::::::::.<br>miRNA 3' CCCCTTACTTCGGACCAGGCT 5'	3	263-283	Homeobox-leucine zipper protein revoluta
vvi-miR166e-3p	VIT_213s0019g04320.5	target 5' CTGGGATGAAGCCTGGTCCGG 3'<br>::.:::::::::::::::.<br>miRNA 3' CCCCTTACTTCGGACCAGGCT 5'	3	173-193	Homeobox-leucine zipper protein revoluta
vvi-miR166e-3p	VIT_213s0019g04320.6	target 5' CTGGGATGAAGCCTGGTCCGG 3'<br>::.:::::::::::::::.<br>miRNA 3' CCCCTTACTTCGGACCAGGCT 5'	3	263-283	Homeobox-leucine zipper protein revoluta
vvi-miR166e-3p	VIT_213s0019g04320.7	target 5' CTGGGATGAAGCCTGGTCCGG 3'<br>::.:::::::::::::::.<br>miRNA 3' CCCCTTACTTCGGACCAGGCT 5'	3	263-283	Homeobox-leucine zipper protein revoluta
vvi-miR166f-3p	VIT_204s0008g03250.1	target 5' CTGGGATGAAGCCTGGTCCGG 3'<br>::.:::::::::::::::.<br>miRNA 3' CCCCTTACTTCGGACCAGGCT 5'	3	557-577	Protein

miRNA	Target id	Alignment	Score	Range	Target gene functional annotation
vvi-miR166f-3p	VIT_204s0008g03250.2	target 5' CTGGGATGAAGCCTGGTCCGG 3'<br>::.:::::::::::::::::.<br>miRNA 3' CCCCTTACTTCGGACCAGGCT 5'	3	557-577	Class iii hd-zip protein 8
vvi-miR166f-3p	VIT_206s0004g02800.1	target 5' CTGGGATGAAGCCTGGTCCGG 3'<br>::.:::::::::::::::::.<br>miRNA 3' CCCCTTACTTCGGACCAGGCT 5'	3	563-583	Homeobox leucine-zipper protein
vvi-miR166f-3p	VIT_206s0004g02800.2	target 5' CTGGGATGAAGCCTGGTCCGG 3'<br>::.:::::::::::::::::.<br>miRNA 3' CCCCTTACTTCGGACCAGGCT 5'	3	563-583	Homeobox leucine-zipper protein
vvi-miR166f-3p	VIT_206s0004g02800.3	target 5' CTGGGATGAAGCCTGGTCCGG 3'<br>::.:::::::::::::::::.<br>miRNA 3' CCCCTTACTTCGGACCAGGCT 5'	3	563-583	Homeobox leucine-zipper protein
vvi-miR166f-3p	VIT_209s0002g03740.1	target 5' CTGGAATGAAGCCTGGTCCGG 3'<br>::::::::::::::::::::.<br>miRNA 3' CCCCTTACTTCGGACCAGGCT 5'	2.5	551-571	Homeobox-leucine zipper protein athb-15
vvi-miR166f-3p	VIT_210s0003g04670.1	target 5' TTGGGATGAAGCCTGGTCCGG 3'<br>::.:::::::::::::::::.<br>miRNA 3' CCCCTTACTTCGGACCAGGCT 5'	3	566-586	Homeobox-leucine zipper protein hox32-like
vvi-miR166f-3p	VIT_210s0003g04670.2	target 5' TTGGGATGAAGCCTGGTCCGG 3'<br>::.:::::::::::::::::.<br>miRNA 3' CCCCTTACTTCGGACCAGGCT 5'	3	293-313	Homeobox-leucine zipper protein hox32-like
vvi-miR166f-3p	VIT_210s0003g04670.3	target 5' TTGGGATGAAGCCTGGTCCGG 3'<br>::.:::::::::::::::::.<br>miRNA 3' CCCCTTACTTCGGACCAGGCT 5'	3	25-May	Homeobox-leucine zipper protein hox32-like
vvi-miR166f-3p	VIT_213s0019g04320.1	target 5' CTGGGATGAAGCCTGGTCCGG 3'<br>::.:::::::::::::::::.<br>miRNA 3' CCCCTTACTTCGGACCAGGCT 5'	3	263-283	Homeobox-leucine zipper protein revoluta
vvi-miR166f-3p	VIT_213s0019g04320.2	target 5' CTGGGATGAAGCCTGGTCCGG 3'<br>::.:::::::::::::::::.<br>miRNA 3' CCCCTTACTTCGGACCAGGCT 5'	3	263-283	Homeobox-leucine zipper protein revoluta
vvi-miR166f-3p	VIT_213s0019g04320.3	target 5' CTGGGATGAAGCCTGGTCCGG 3'<br>::.:::::::::::::::::.<br>miRNA 3' CCCCTTACTTCGGACCAGGCT 5'	3	263-283	Homeobox-leucine zipper protein revoluta
vvi-miR166f-3p	VIT_213s0019g04320.4	target 5' CTGGGATGAAGCCTGGTCCGG 3'<br>::.:::::::::::::::::.<br>miRNA 3' CCCCTTACTTCGGACCAGGCT 5'	3	263-283	Homeobox-leucine zipper protein revoluta
vvi-miR166f-3p	VIT_213s0019g04320.5	target 5' CTGGGATGAAGCCTGGTCCGG 3'<br>::.:::::::::::::::::.<br>miRNA 3' CCCCTTACTTCGGACCAGGCT 5'	3	173-193	Homeobox-leucine zipper protein revoluta
vvi-miR166f-3p	VIT_213s0019g04320.6	target 5' CTGGGATGAAGCCTGGTCCGG 3'<br>::.:::::::::::::::::.<br>miRNA 3' CCCCTTACTTCGGACCAGGCT 5'	3	263-283	Homeobox-leucine zipper protein revoluta

miRNA	Target id	Alignment	Score	Range	Target gene functional annotation
vvi-miR166f-3p	VIT_213s0019g04320.7	target 5' CTGGGATGAAGCCTGGTCCGG 3'<br>::.:::::::::::::::::.<br>miRNA 3' CCCCTTACTTCGGACCAGGCT 5'	3	263-283	Homeobox-leucine zipper protein revoluta
vvi-miR166g-3p	VIT_204s0008g03250.1	target 5' CTGGGATGAAGCCTGGTCCGG 3'<br>::.:::::::::::::::::.<br>miRNA 3' CCCCTTACTTCGGACCAGGCT 5'	3	557-577	Protein
vvi-miR166g-3p	VIT_204s0008g03250.2	target 5' CTGGGATGAAGCCTGGTCCGG 3'<br>::.:::::::::::::::::.<br>miRNA 3' CCCCTTACTTCGGACCAGGCT 5'	3	557-577	Class iii hd-zip protein 8
vvi-miR166g-3p	VIT_206s0004g02800.1	target 5' CTGGGATGAAGCCTGGTCCGG 3'<br>::.:::::::::::::::::.<br>miRNA 3' CCCCTTACTTCGGACCAGGCT 5'	3	563-583	Homeobox leucine-zipper protein
vvi-miR166g-3p	VIT_206s0004g02800.2	target 5' CTGGGATGAAGCCTGGTCCGG 3'<br>::.:::::::::::::::::.<br>miRNA 3' CCCCTTACTTCGGACCAGGCT 5'	3	563-583	Homeobox leucine-zipper protein
vvi-miR166g-3p	VIT_206s0004g02800.3	target 5' CTGGGATGAAGCCTGGTCCGG 3'<br>::.:::::::::::::::::.<br>miRNA 3' CCCCTTACTTCGGACCAGGCT 5'	3	563-583	Homeobox leucine-zipper protein
vvi-miR166g-3p	VIT_209s0002g03740.1	target 5' CTGGAATGAAGCCTGGTCCGG 3'<br>::::::::::::::::::.<br>miRNA 3' CCCCTTACTTCGGACCAGGCT 5'	2.5	551-571	Homeobox-leucine zipper protein athb-15
vvi-miR166g-3p	VIT_210s0003g04670.1	target 5' TTGGGATGAAGCCTGGTCCGG 3'<br>::.:::::::::::::::::.<br>miRNA 3' CCCCTTACTTCGGACCAGGCT 5'	3	566-586	Homeobox-leucine zipper protein hox32-like
vvi-miR166g-3p	VIT_210s0003g04670.2	target 5' TTGGGATGAAGCCTGGTCCGG 3'<br>::.:::::::::::::::::.<br>miRNA 3' CCCCTTACTTCGGACCAGGCT 5'	3	293-313	Homeobox-leucine zipper protein hox32-like
vvi-miR166g-3p	VIT_210s0003g04670.3	target 5' TTGGGATGAAGCCTGGTCCGG 3'<br>::.:::::::::::::::::.<br>miRNA 3' CCCCTTACTTCGGACCAGGCT 5'	3	25-May	Homeobox-leucine zipper protein hox32-like
vvi-miR166g-3p	VIT_213s0019g04320.1	target 5' CTGGGATGAAGCCTGGTCCGG 3'<br>::.:::::::::::::::::.<br>miRNA 3' CCCCTTACTTCGGACCAGGCT 5'	3	263-283	Homeobox-leucine zipper protein revoluta
vvi-miR166g-3p	VIT_213s0019g04320.2	target 5' CTGGGATGAAGCCTGGTCCGG 3'<br>::.:::::::::::::::::.<br>miRNA 3' CCCCTTACTTCGGACCAGGCT 5'	3	263-283	Homeobox-leucine zipper protein revoluta
vvi-miR166g-3p	VIT_213s0019g04320.3	target 5' CTGGGATGAAGCCTGGTCCGG 3'<br>::.:::::::::::::::::.<br>miRNA 3' CCCCTTACTTCGGACCAGGCT 5'	3	263-283	Homeobox-leucine zipper protein revoluta
vvi-miR166g-3p	VIT_213s0019g04320.4	target 5' CTGGGATGAAGCCTGGTCCGG 3'<br>::.:::::::::::::::::.<br>miRNA 3' CCCCTTACTTCGGACCAGGCT 5'	3	263-283	Homeobox-leucine zipper protein revoluta

miRNA	Target id	Alignment	Score	Range	Target gene functional annotation
vvi-miR166g-3p	VIT_213s0019g04320.5	target 5' CTGGGATGAAGCCTGGTCCGG 3'<br>::.:::::::::::::::.<br>miRNA 3' CCCCTTACTTCGGACCAGGCT 5'	3	173-193	Homeobox-leucine zipper protein revoluta
vvi-miR166g-3p	VIT_213s0019g04320.6	target 5' CTGGGATGAAGCCTGGTCCGG 3'<br>::.:::::::::::::::.<br>miRNA 3' CCCCTTACTTCGGACCAGGCT 5'	3	263-283	Homeobox-leucine zipper protein revoluta
vvi-miR166g-3p	VIT_213s0019g04320.7	target 5' CTGGGATGAAGCCTGGTCCGG 3'<br>::.:::::::::::::::.<br>miRNA 3' CCCCTTACTTCGGACCAGGCT 5'	3	263-283	Homeobox-leucine zipper protein revoluta
vvi-miR166h-3p	VIT_204s0008g03250.1	target 5' CTGGGATGAAGCCTGGTCCGG 3'<br>::.:::::::::::::::.<br>miRNA 3' CCCCTTACTTCGGACCAGGCT 5'	3	557-577	Protein
vvi-miR166h-3p	VIT_204s0008g03250.2	target 5' CTGGGATGAAGCCTGGTCCGG 3'<br>::.:::::::::::::::.<br>miRNA 3' CCCCTTACTTCGGACCAGGCT 5'	3	557-577	Class iii hd-zip protein 8
vvi-miR166h-3p	VIT_206s0004g02800.1	target 5' CTGGGATGAAGCCTGGTCCGG 3'<br>::.:::::::::::::::.<br>miRNA 3' CCCCTTACTTCGGACCAGGCT 5'	3	563-583	Homeobox leucine-zipper protein
vvi-miR166h-3p	VIT_206s0004g02800.2	target 5' CTGGGATGAAGCCTGGTCCGG 3'<br>::.:::::::::::::::.<br>miRNA 3' CCCCTTACTTCGGACCAGGCT 5'	3	563-583	Homeobox leucine-zipper protein
vvi-miR166h-3p	VIT_206s0004g02800.3	target 5' CTGGGATGAAGCCTGGTCCGG 3'<br>::.:::::::::::::::.<br>miRNA 3' CCCCTTACTTCGGACCAGGCT 5'	3	563-583	Homeobox leucine-zipper protein
vvi-miR166h-3p	VIT_209s0002g03740.1	target 5' CTGGAATGAAGCCTGGTCCGG 3'<br>::::::::::::::::::.<br>miRNA 3' CCCCTTACTTCGGACCAGGCT 5'	2.5	551-571	Homeobox-leucine zipper protein athb-15
vvi-miR166h-3p	VIT_210s0003g04670.1	target 5' TTGGGATGAAGCCTGGTCCGG 3'<br>::.:::::::::::::::.<br>miRNA 3' CCCCTTACTTCGGACCAGGCT 5'	3	566-586	Homeobox-leucine zipper protein hox32-like
vvi-miR166h-3p	VIT_210s0003g04670.2	target 5' TTGGGATGAAGCCTGGTCCGG 3'<br>::.:::::::::::::::.<br>miRNA 3' CCCCTTACTTCGGACCAGGCT 5'	3	293-313	Homeobox-leucine zipper protein hox32-like
vvi-miR166h-3p	VIT_210s0003g04670.3	target 5' TTGGGATGAAGCCTGGTCCGG 3'<br>::.:::::::::::::::.<br>miRNA 3' CCCCTTACTTCGGACCAGGCT 5'	3	25-May	Homeobox-leucine zipper protein hox32-like
vvi-miR166h-3p	VIT_213s0019g04320.1	target 5' CTGGGATGAAGCCTGGTCCGG 3'<br>::.:::::::::::::::.<br>miRNA 3' CCCCTTACTTCGGACCAGGCT 5'	3	263-283	Homeobox-leucine zipper protein revoluta
vvi-miR166h-3p	VIT_213s0019g04320.2	target 5' CTGGGATGAAGCCTGGTCCGG 3'<br>::.:::::::::::::::.<br>miRNA 3' CCCCTTACTTCGGACCAGGCT 5'	3	263-283	Homeobox-leucine zipper protein revoluta

miRNA	Target id	Alignment	Score	Range	Target gene functional annotation
vvi-miR166h-3p	VIT_213s0019g04320.3	target 5' CTGGGATGAAGCCTGGTCCGG 3'<br>::.:::::::::::::::.<br>miRNA 3' CCCCTTACTTCGGACCAGGCT 5'	3	263-283	Homeobox-leucine zipper protein revoluta
vvi-miR166h-3p	VIT_213s0019g04320.4	target 5' CTGGGATGAAGCCTGGTCCGG 3'<br>::.:::::::::::::::.<br>miRNA 3' CCCCTTACTTCGGACCAGGCT 5'	3	263-283	Homeobox-leucine zipper protein revoluta
vvi-miR166h-3p	VIT_213s0019g04320.5	target 5' CTGGGATGAAGCCTGGTCCGG 3'<br>::.:::::::::::::::.<br>miRNA 3' CCCCTTACTTCGGACCAGGCT 5'	3	173-193	Homeobox-leucine zipper protein revoluta
vvi-miR166h-3p	VIT_213s0019g04320.6	target 5' CTGGGATGAAGCCTGGTCCGG 3'<br>::.:::::::::::::::.<br>miRNA 3' CCCCTTACTTCGGACCAGGCT 5'	3	263-283	Homeobox-leucine zipper protein revoluta
vvi-miR166h-3p	VIT_213s0019g04320.7	target 5' CTGGGATGAAGCCTGGTCCGG 3'<br>::.:::::::::::::::.<br>miRNA 3' CCCCTTACTTCGGACCAGGCT 5'	3	263-283	Homeobox-leucine zipper protein revoluta
vvi-miR168-3p	VIT_208s0007g07890.1	target 5' GTTTAGGTGATGCAAGGCGGG 3'<br>.::.:: ::::::::::::::<br>miRNA 3' TAAGTCAACTACGTTCCGCCC 5'	2	347-367	Pentatricopeptide repeat-containing protein mitochondrial-like
vvi-miR169k-3p	VIT_206s0004g00450.1	target 5' GGTGGTCTAGGACAACCTGCT 3'<br>:::.:.: ::::::::::::.<br>miRNA 3' CCATCGGTTCCTGTTGGACGG 5'	2.5	5188-5208	Rna polymerase ii transcription mediator
vvi-miR169k-3p	VIT_206s0004g00450.2	target 5' GGTGGTCTAGGACAACCTGCT 3'<br>:::.:.: ::::::::::::.<br>miRNA 3' CCATCGGTTCCTGTTGGACGG 5'	2.5	5188-5208	Rna polymerase ii transcription mediator
vvi-miR169w-3p	VIT_204s0008g03660.1	target 5' GATGAGAAGAATGAGGCAGAAA 3'<br>:::::::: ::::::::::::<br>miRNA 3' GTACTCTTC-TACTCCGTCTTT 5'	3	118-139	Embyo flowering 1-like protein
vvi-miR169w-3p	VIT_204s0008g03660.2	target 5' GATGAGAAGAATGAGGCAGAAA 3'<br>:::::::: ::::::::::::<br>miRNA 3' GTACTCTTC-TACTCCGTCTTT 5'	3	118-139	Embyo flowering 1-like protein
vvi-miR169w-3p	VIT_212s0028g03940.1	target 5' TATG-GAGGATGAGGCAGAAC 3'<br>.::: ::.::::::::::::<br>miRNA 3' GTACTCTTCTACTCCGTCTTT 5'	3	933-952	Histone acetyltransferase
vvi-miR169w-3p	VIT_212s0028g03940.2	target 5' TATG-GAGGATGAGGCAGAAC 3'<br>.::: ::.::::::::::::<br>miRNA 3' GTACTCTTCTACTCCGTCTTT 5'	3	933-952	Histone acetyltransferase
vvi-miR169w-3p	VIT_212s0028g03940.3	target 5' TATG-GAGGATGAGGCAGAAC 3'<br>.::: ::.::::::::::::<br>miRNA 3' GTACTCTTCTACTCCGTCTTT 5'	3	933-952	Histone acetyltransferase
vvi-miR169w-3p	VIT_212s0028g03940.4	target 5' TATG-GAGGATGAGGCAGAAC 3'<br>.::: ::.::::::::::::<br>miRNA 3' GTACTCTTCTACTCCGTCTTT 5'	3	834-853	Histone acetyltransferase

miRNA	Target id	Alignment	Score	Range	Target gene functional annotation
vvi-miR169w-3p	VIT_212s0028g03940.5	target 5' TATG-GAGGATGAGGCAGAAC 3'<br>.::: ::.::::::::::::<br>miRNA 3' GTACTCTTCTACTCCGTCTTT 5'	3	738-757	Histone acetyltransferase
vvi-miR169w-3p	VIT_212s0028g03940.6	target 5' TATG-GAGGATGAGGCAGAAC 3'<br>.::: ::.::::::::::::<br>miRNA 3' GTACTCTTCTACTCCGTCTTT 5'	3	465-484	Histone acetyltransferase
vvi-miR169w-3p	VIT_217s0053g00583.1	target 5' CTTGGAAGGATGAGGCAGAAA 3'<br>: ::. :.:::::::::::::<br>miRNA 3' GTACTCTTCTACTCCGTCTTT 5'	3	41-61	Cullin 3-like protein
vvi-miR171b	VIT_202s0154g00400.1	target 5' AGGGATATTGGCGCGGCTCAA 3'<br>:.:::::::.::::::::::<br>miRNA 3' CCTCTATAACTGCGCCGAGTT 5'	2.5	1218-1238	Gras family transcription factor
vvi-miR171b	VIT_204s0023g01380.1	target 5' GGCGATATTGGCGCGGCTCAA 3'<br>:: :::::::.::::::::::<br>miRNA 3' CCTCTATAACTGCGCCGAGTT 5'	2	585-605	Gras family transcription factor
vvi-miR171b	VIT_215s0048g00270.1	target 5' AGGGATATTGGCGCGGCTCAA 3'<br>:.:::::::.::::::::::<br>miRNA 3' CCTCTATAACTGCGCCGAGTT 5'	2.5	1230-1250	Gras family transcription factor
vvi-miR171e-3p	VIT_202s0154g00400.1	target 5' AGGGATATTGGCGCGGCTCAA 3'<br>:: ::::::::::::::::::<br>miRNA 3' TCACTATAACCGCGCCGAGTT 5'	1	1218-1238	Gras family transcription factor
vvi-miR171e-3p	VIT_204s0023g01380.1	target 5' GGCGATATTGGCGCGGCTCAA 3'<br>.: ::::::::::::::::::<br>miRNA 3' TCACTATAACCGCGCCGAGTT 5'	1.5	585-605	Gras family transcription factor
vvi-miR171e-3p	VIT_215s0048g00270.1	target 5' AGGGATATTGGCGCGGCTCAA 3'<br>:: ::::::::::::::::::<br>miRNA 3' TCACTATAACCGCGCCGAGTT 5'	1	1230-1250	Gras family transcription factor
vvi-miR171f-3p	VIT_202s0154g00400.1	target 5' AGGGATATTGGCGCGGCTCAA 3'<br>:: ::::::::::::::::::<br>miRNA 3' TCACTATAACCGCGCCGAGTT 5'	1	1218-1238	Gras family transcription factor
vvi-miR171f-3p	VIT_204s0023g01380.1	target 5' GGCGATATTGGCGCGGCTCAA 3'<br>.: ::::::::::::::::::<br>miRNA 3' TCACTATAACCGCGCCGAGTT 5'	1.5	585-605	Gras family transcription factor
vvi-miR171f-3p	VIT_215s0048g00270.1	target 5' AGGGATATTGGCGCGGCTCAA 3'<br>:: ::::::::::::::::::<br>miRNA 3' TCACTATAACCGCGCCGAGTT 5'	1	1230-1250	Gras family transcription factor
vvi-miR171h	VIT_202s0154g00400.1	target 5' AGGGATATTGGCGCGGCTCAA 3'<br>::::::::::::::::::::<br>miRNA 3' GCCCTATAACCGCGCCGAGTT 5'	1	1218-1238	Gras family transcription factor
vvi-miR171h	VIT_204s0023g01380.1	target 5' GGCGATATTGGCGCGGCTCAA 3'<br>: ::::::::::::::::::<br>miRNA 3' GCCCTATAACCGCGCCGAGTT 5'	2	585-605	Gras family transcription factor

miRNA	Target id	Alignment	Score	Range	Target gene functional annotation
vvi-miR171h	VIT_212s0059g00650.1	target 5' CGGGATATTGGTGCGGTTCAA 3'<br>:::::::::::.::::.::::<br>miRNA 3' GCCCTATAACCGCGCCGAGTT 5'	1	142-166	Hypothetical protein
vvi-miR171h	VIT_215s0048g00270.1	target 5' AGGGATATTGGCGCGGCTCAA 3'<br>::::::::::::::::::::<br>miRNA 3' GCCCTATAACCGCGCCGAGTT 5'	1	1230-1250	Gras family transcription factor
vvi-miR172c-3p	VIT_206s0004g03590.1	target 5' CTGCAGCATCATCAGGATTCT 3'<br>::::::::::::::.:::::.<br>miRNA 3' GACGTCGTAGTAGTTCTAAGG 5'	1.5	1334-1354	Ethylene-responsive transcription factor rap2-7-like
vvi-miR172c-3p	VIT_206s0004g03590.3	target 5' CTGCAGCATCATCAGGATTCT 3'<br>::::::::::::::.:::::.<br>miRNA 3' GACGTCGTAGTAGTTCTAAGG 5'	1.5	1289-1309	Ethylene-responsive transcription factor rap2-7-like
vvi-miR172c-3p	VIT_207s0031g00220.1	target 5' CTGCAGCATCATCAGGATTCC 3'<br>::::::::::::::.::::::<br>miRNA 3' GACGTCGTAGTAGTTCTAAGG 5'	1	1430-1450	Transcription factor apetala2
vvi-miR172c-3p	VIT_207s0031g00220.2	target 5' CTGCAGCATCATCAGGATTCC 3'<br>::::::::::::::.::::::<br>miRNA 3' GACGTCGTAGTAGTTCTAAGG 5'	1	1442-1462	Transcription factor apetala2
vvi-miR172c-3p	VIT_208s0040g03180.1	target 5' CTGCAGCATCATCAGGATTCT 3'<br>::::::::::::::.:::::.<br>miRNA 3' GACGTCGTAGTAGTTCTAAGG 5'	1.5	1331-1351	Ap2 domain-containing transcription factor
vvi-miR172c-3p	VIT_213s0019g03550.1	target 5' CTGCAGCATCATCAGGATTCC 3'<br>::::::::::::::.::::::<br>miRNA 3' GACGTCGTAGTAGTTCTAAGG 5'	1	1304-1324	Ap2 domain-containing transcription factor
vvi-miR172c-3p	VIT_213s0019g03550.2	target 5' CTGCAGCATCATCAGGATTCC 3'<br>::::::::::::::.::::::<br>miRNA 3' GACGTCGTAGTAGTTCTAAGG 5'	1	1259-1279	Ap2 domain-containing transcription factor
vvi-miR172c-5p	VIT_207s0191g00170.1	target 5' TGTAAAACTTGATGATGCTCA 3'<br>::: :: :::::::::::::<br>miRNA 3' ACACTTAGAACTACTACGAGG 5'	3	573-593	Lmbr1 domain-containing protein 2 homolog a-like
vvi-miR172c-5p	VIT_207s0191g00170.2	target 5' TGTAAAACTTGATGATGCTCA 3'<br>::: :: :::::::::::::<br>miRNA 3' ACACTTAGAACTACTACGAGG 5'	3	183-203	Lmbr1 domain-containing protein 2 homolog a-like
vvi-miR172c-5p	VIT_207s0191g00170.3	target 5' TGTAAAACTTGATGATGCTCA 3'<br>::: :: :::::::::::::<br>miRNA 3' ACACTTAGAACTACTACGAGG 5'	3	573-593	Lmbr1 domain-containing protein 2 homolog a-like
vvi-miR172c-5p	VIT_207s0191g00170.6	target 5' TGTAAAACTTGATGATGCTCA 3'<br>::: :: :::::::::::::<br>miRNA 3' ACACTTAGAACTACTACGAGG 5'	3	573-593	Lmbr1 domain-containing protein 2 homolog a-like
vvi-miR172c-5p	VIT_207s0191g00170.7	target 5' TGTAAAACTTGATGATGCTCA 3'<br>::: :: :::::::::::::<br>miRNA 3' ACACTTAGAACTACTACGAGG 5'	3	573-593	Lmbr1 domain-containing protein 2 homolog a-like

miRNA	Target id	Alignment	Score	Range	Target gene functional annotation
vvi-miR172c-5p	VIT_207s0191g00170.8	target 5' TGTAAAACTTGATGATGCTCA 3'<br>::: :: :::::::::::::<br>miRNA 3' ACACTTAGAACTACTACGAGG 5'	3	183-203	Lmbr1 domain-containing protein 2 homolog a-like
vvi-miR172c-5p	VIT_208s0007g05895.1	target 5' TGTGAGTGTTGATGATGCTCT 3'<br>:::::.: ::::::::::::.<br>miRNA 3' ACACTTAGAACTACTACGAGG 5'	2	702-722	F-box family protein
vvi-miR172d	VIT_200s0184g00102.1	target 5' GCATTCATGATCAAGATTCTCA 3'<br>::: ::: :::::::::::::<br>miRNA 3' CGT-CGTAGTAGTTCTAAGAGT 5'	3	215-236	Ap2 erf and b3 domain-containing transcription factor rav2
vvi-miR172d	VIT_206s0004g03590.1	target 5' GCAGCATCATCAGGATTCTCA 3'<br>:::::::::::::.:::::::<br>miRNA 3' CGTCGTAGTAGTTCTAAGAGT 5'	1	1336-1356	Ethylene-responsive transcription factor rap2-7-like
vvi-miR172d	VIT_206s0004g03590.3	target 5' GCAGCATCATCAGGATTCTCA 3'<br>:::::::::::::.:::::::<br>miRNA 3' CGTCGTAGTAGTTCTAAGAGT 5'	1	1291-1311	Ethylene-responsive transcription factor rap2-7-like
vvi-miR172d	VIT_207s0031g00220.1	target 5' GCAGCATCATCAGGATTCCCA 3'<br>:::::::::::::.:::: ::<br>miRNA 3' CGTCGTAGTAGTTCTAAGAGT 5'	3	1432-1452	Transcription factor apetala2
vvi-miR172d	VIT_207s0031g00220.2	target 5' GCAGCATCATCAGGATTCCCA 3'<br>:::::::::::::.:::: ::<br>miRNA 3' CGTCGTAGTAGTTCTAAGAGT 5'	3	1444-1464	Transcription factor apetala2
vvi-miR172d	VIT_208s0040g03180.1	target 5' GCAGCATCATCAGGATTCTTG 3'<br>:::::::::::::.:::::..<br>miRNA 3' CGTCGTAGTAGTTCTAAGAGT 5'	2.5	1333-1353	Ap2 domain-containing transcription factor
vvi-miR2111-3p	VIT_209s0002g02550.1	target 5' AGTAAT-TGCAACCAGAGGAT 3'<br>:::::: :::::::::::::.<br>miRNA 3' TCATTAGACGTTGGTCTCCTG 5'	1.5	955-974	Tudor pwwp mbt superfamily protein
vvi-miR2111-3p	VIT_209s0002g02550.2	target 5' AGTAAT-TGCAACCAGAGGAT 3'<br>:::::: :::::::::::::.<br>miRNA 3' TCATTAGACGTTGGTCTCCTG 5'	1.5	1399-1418	Tudor pwwp mbt superfamily protein
vvi-miR2111-3p	VIT_209s0002g02550.3	target 5' AGTAAT-TGCAACCAGAGGAT 3'<br>:::::: :::::::::::::.<br>miRNA 3' TCATTAGACGTTGGTCTCCTG 5'	1.5	955-974	Tudor pwwp mbt superfamily protein
vvi-miR2111-3p	VIT_209s0002g02550.4	target 5' AGTAAT-TGCAACCAGAGGAT 3'<br>:::::: :::::::::::::.<br>miRNA 3' TCATTAGACGTTGGTCTCCTG 5'	1.5	955-974	Tudor pwwp mbt superfamily protein
vvi-miR2111-3p	VIT_209s0002g02550.5	target 5' AGTAAT-TGCAACCAGAGGAT 3'<br>:::::: :::::::::::::.<br>miRNA 3' TCATTAGACGTTGGTCTCCTG 5'	1.5	955-974	Tudor pwwp mbt superfamily protein
vvi-miR2111-3p	VIT_209s0002g02550.6	target 5' AGTAAT-TGCAACCAGAGGAT 3'<br>:::::: :::::::::::::.<br>miRNA 3' TCATTAGACGTTGGTCTCCTG 5'	1.5	955-974	Tudor pwwp mbt superfamily protein

miRNA	Target id	Alignment	Score	Range	Target gene functional annotation
vvi-miR2111-5p	VIT_214s0068g02150.1	target 5' TAGATACTCAGGATGCAGATTA 3'<br>::::. ::::::::::::::::<br>miRNA 3' ATCTG-GAGTCCTACGTCTAAT 5'	1.5	239-260	F-box kelch-repeat protein at3g27150-like
vvi-miR2111-5p	VIT_214s0068g02150.2	target 5' TAGATACTCAGGATGCAGATTA 3'<br>::::. ::::::::::::::::<br>miRNA 3' ATCTG-GAGTCCTACGTCTAAT 5'	1.5	239-260	F-box kelch-repeat protein at3g27150-like
vvi-miR2111-5p	VIT_214s0068g02150.3	target 5' TAGATACTCAGGATGCAGATTA 3'<br>::::. ::::::::::::::::<br>miRNA 3' ATCTG-GAGTCCTACGTCTAAT 5'	1.5	410-431	F-box kelch-repeat protein at3g27150-like
vvi-miR2111-5p	VIT_214s0068g02150.4	target 5' TAGATACTCAGGATGCAGATTA 3'<br>::::. ::::::::::::::::<br>miRNA 3' ATCTG-GAGTCCTACGTCTAAT 5'	1.5	239-260	F-box kelch-repeat protein at3g27150-like
vvi-miR2111-5p	VIT_214s0068g02150.5	target 5' TAGATACTCAGGATGCAGATTA 3'<br>::::. ::::::::::::::::<br>miRNA 3' ATCTG-GAGTCCTACGTCTAAT 5'	1.5	239-260	F-box kelch-repeat protein at3g27150-like
vvi-miR2950-5p	VIT_209s0002g01600.1	target 5' CAACTTTGCAAGAGATGGAAT 3'<br>: : : :::::::::::::::<br>miRNA 3' GGTCACACGTTCTCTACCTTA 5'	3	234-254	Dna binding
vvi-miR319b-3p	VIT_210s0003g00870.1	target 5' GGGAAC-CCCTTCAGTCCAC 3'<br>:::: : :::::::::::<br>miRNA 3' CCCTCGAGGGAAGTCAGGTT 5'	3	926-944	Transcription factor tcp4-like
vvi-miR319b-3p	VIT_213s0067g01630.1	target 5' TGGAGCTCCCTTCACTCCAA 3'<br>::::::::::::: :::::<br>miRNA 3' CCCTCGAGGGAAGTCAGGTT 5'	3	956-975	Transcription factor gamyb-like
vvi-miR319c-3p	VIT_210s0003g00870.1	target 5' GGGAAC-CCCTTCAGTCCAC 3'<br>:::: : :::::::::::<br>miRNA 3' CCCTCGAGGGAAGTCAGGTT 5'	3	926-944	Transcription factor tcp4-like
vvi-miR319c-3p	VIT_213s0067g01630.1	target 5' TGGAGCTCCCTTCACTCCAA 3'<br>::::::::::::: :::::<br>miRNA 3' CCCTCGAGGGAAGTCAGGTT 5'	3	956-975	Transcription factor gamyb-like
vvi-miR319c-5p	VIT_214s0081g00480.1	target 5' GAATGCACTGAAGAAAGCTCA 3'<br>:: :: ::::::::::::::<br>miRNA 3' CTCACCTGACTTCTTTCGAGA 5'	3	322-342	Protein
vvi-miR319f-3p	VIT_210s0003g00870.1	target 5' GGGAAC-CCCTTCAGTCCAC 3'<br>:::: : :::::::::::<br>miRNA 3' CCCTCGAGGGAAGTCAGGTT 5'	3	926-944	Transcription factor tcp4-like
vvi-miR319f-3p	VIT_213s0067g01630.1	target 5' TGGAGCTCCCTTCACTCCAA 3'<br>::::::::::::: :::::<br>miRNA 3' CCCTCGAGGGAAGTCAGGTT 5'	3	956-975	Transcription factor gamyb-like
vvi-miR319g	VIT_210s0003g00870.1	target 5' GGGAAC-CCCTTCAGTCCAC 3'<br>:::: : :::::::::::<br>miRNA 3' CCCTCGAGGGAAGTCAGGTT 5'	3	926-944	Transcription factor tcp4-like

miRNA	Target id	Alignment	Score	Range	Target gene functional annotation
vvi-miR319g	VIT_213s0067g01630.1	target 5' TGGAGCTCCCTTCACTCCAA 3'<br>::::::::::::: :::::<br>miRNA 3' CCCTCGAGGGAAGTCAGGTT 5'	3	956-975	Transcription factor gamyb-like
vvi-miR3623-5p	VIT_218s0041g02180.2	target 5' TGGTGCTTGGAGGAGCTTGTGA 3'<br>::::::::::: ::.:::::::<br>miRNA 3' ACCACGAACCTACTTGAACACT 5'	3	115-136	Protein da1-related 1-like
vvi-miR3623-5p	VIT_218s0041g02180.3	target 5' TGGTGCTTGGAGGAGCTTGTGA 3'<br>::::::::::: ::.:::::::<br>miRNA 3' ACCACGAACCTACTTGAACACT 5'	3	271-292	Protein da1-related 1-like
vvi-miR3624-3p	VIT_200s0194g00300.1	target 5' AGTAGTATGCTGCTGCCCTGA 3'<br>:::::::::::::::::::::<br>miRNA 3' TCATCATACGACGACGGGACT 5'	0	141-161	Uncharacterized protein loc100260893
vvi-miR3624-3p	VIT_200s0194g00340.1	target 5' AGTAGTATGCTGCTGCCCTGA 3'<br>:::::::::::::::::::::<br>miRNA 3' TCATCATACGACGACGGGACT 5'	0	29-Sep	Uncharacterized protein loc100852467
vvi-miR3624-3p	VIT_200s0194g00340.2	target 5' AGTAGTATGCTGCTGCCCTGA 3'<br>:::::::::::::::::::::<br>miRNA 3' TCATCATACGACGACGGGACT 5'	0	123-143	Metal ion binding protein
vvi-miR3629a-5p	VIT_208s0040g01510.1	target 5' TTTGGTTGCTGAGAAAAT-CG 3'<br>.::::.:::::::::::: ::<br>miRNA 3' GAACCGACGACTCTTTTACGC 5'	3	48-67	Uncharacterized protein loc100853283
vvi-miR3629a-5p	VIT_212s0028g01350.1	target 5' ATTGG-TGCTGAGAAAATGCT 3'<br>:::: ::::::::::::::<br>miRNA 3' GAACCGACGACTCTTTTACGC 5'	3	834-853	Lrr receptor-like serine threonine-protein kinase
vvi-miR3629a-5p	VIT_212s0028g01350.2	target 5' ATTGG-TGCTGAGAAAATGCT 3'<br>:::: ::::::::::::::<br>miRNA 3' GAACCGACGACTCTTTTACGC 5'	3	834-853	Lrr receptor-like serine threonine-protein kinase
vvi-miR3629a-5p	VIT_212s0028g01350.3	target 5' ATTGG-TGCTGAGAAAATGCT 3'<br>:::: ::::::::::::::<br>miRNA 3' GAACCGACGACTCTTTTACGC 5'	3	447-466	Lrr receptor-like serine threonine-protein kinase
vvi-miR3629a-5p	VIT_212s0028g01350.4	target 5' ATTGG-TGCTGAGAAAATGCT 3'<br>:::: ::::::::::::::<br>miRNA 3' GAACCGACGACTCTTTTACGC 5'	3	621-640	Lrr receptor-like serine threonine-protein kinase
vvi-miR3629a-5p	VIT_212s0028g01350.5	target 5' ATTGG-TGCTGAGAAAATGCT 3'<br>:::: ::::::::::::::<br>miRNA 3' GAACCGACGACTCTTTTACGC 5'	3	834-853	Lrr receptor-like serine threonine-protein kinase
vvi-miR3632-3p	VIT_213s0067g00790.1	target 5' ATGGTATGGGGGGTCTGGGAAA 3'<br>:::::: ::::::::::::::<br>miRNA 3' AACCATAACCCCCAGACCCTTT 5'	2	575-596	Disease resistance protein
vvi-miR3632-3p	VIT_213s0067g00830.1	target 5' ATGGTATGGGGGGTCTGGGAAA 3'<br>:::::: ::::::::::::::<br>miRNA 3' AACCATAACCCCCAGACCCTTT 5'	2	578-599	Probable disease resistance rpp8-like protein 2-like

miRNA	Target id	Alignment	Score	Range	Target gene functional annotation
vvi-miR3632-3p	VIT_213s0067g00830.2	target 5' ATGGTATGGGGGGTCTGGGAAA 3'<br>:::::: ::::::::::::::<br>miRNA 3' AACCATAACCCCCAGACCCTTT 5'	2	578-599	Probable disease resistance rpp8-like protein 2-like
vvi-miR3632-3p	VIT_213s0067g00830.3	target 5' ATGGTATGGGGGGTCTGGGAAA 3'<br>:::::: ::::::::::::::<br>miRNA 3' AACCATAACCCCCAGACCCTTT 5'	2	578-599	Probable disease resistance rpp8-like protein 2-like
vvi-miR3632-3p	VIT_213s0067g00830.4	target 5' ATGGTATGGGGGGTCTGGGAAA 3'<br>:::::: ::::::::::::::<br>miRNA 3' AACCATAACCCCCAGACCCTTT 5'	2	578-599	Probable disease resistance rpp8-like protein 2-like
vvi-miR3633b-5p	VIT_203s0180g00280.1	target 5' GGAT-CCAGCCATCCATTCT 3'<br>.::: :::::::.::::::.<br>miRNA 3' TCTAGGGTCGGTGGGTAAGG 5'	3	431-449	Limonoid udp-glucosyltransferase
vvi-miR3633b-5p	VIT_210s0003g03540.1	target 5' AGATTTCAGCCACCCATTAC 3'<br>::::..:::::::::::: :<br>miRNA 3' TCTAGGGTCGGTGGGTAAGG 5'	3	633-652	Beta-amyrin synthase
vvi-miR3633b-5p	VIT_212s0034g02470.1	target 5' AGATCCCA-CCATCCATTCC 3'<br>:::::::: :::.:::::::<br>miRNA 3' TCTAGGGTCGGTGGGTAAGG 5'	3	1081-1099	Disease resistance protein at3g14460-like
vvi-miR3634-3p	VIT_219s0085g00430.1	target 5' ATGGCATGAGTG-GAGTCGGAAA 3'<br>:.:::::::::: ::::::::::<br>miRNA 3' TGCCGTACTCACGCTCAGCCTTT 5'	2.5	86-107	Uncharacterized protein loc100855287
vvi-miR3634-3p	VIT_219s0085g00470.1	target 5' ATGGCATGAGTG-GAGTCGGAAA 3'<br>:.:::::::::: ::::::::::<br>miRNA 3' TGCCGTACTCACGCTCAGCCTTT 5'	2.5	116-137	Uncharacterized protein loc100854011
vvi-miR3635-3p	VIT_217s0000g05850.1	target 5' GAGGCATGTGTGGGACATAAT 3'<br>:::::::::::::::::::::<br>miRNA 3' CTCCGTACACACCCTGTATTA 5'	0	2097-2117	Abc transporter retinal flippase subfamily
vvi-miR3635-3p	VIT_217s0000g05850.2	target 5' GAGGCATGTGTGGGACATAAT 3'<br>:::::::::::::::::::::<br>miRNA 3' CTCCGTACACACCCTGTATTA 5'	0	2097-2117	Abc transporter retinal flippase subfamily
vvi-miR3635-3p	VIT_217s0000g05850.3	target 5' GAGGCATGTGTGGGACATAAT 3'<br>:::::::::::::::::::::<br>miRNA 3' CTCCGTACACACCCTGTATTA 5'	0	2175-2195	Abc transporter retinal flippase subfamily
vvi-miR3635-3p	VIT_217s0000g05850.4	target 5' GAGGCATGTGTGGGACATAAT 3'<br>:::::::::::::::::::::<br>miRNA 3' CTCCGTACACACCCTGTATTA 5'	0	2175-2195	Abc transporter retinal flippase subfamily
vvi-miR3635-3p	VIT_217s0000g05850.5	target 5' GAGGCATGTGTGGGACATAAT 3'<br>:::::::::::::::::::::<br>miRNA 3' CTCCGTACACACCCTGTATTA 5'	0	2175-2195	Abc transporter retinal flippase subfamily
vvi-miR3635-5p	VIT_219s0015g00010.1	target 5' CCGCTATGCCCCACACATGCC 3'<br>: . :::::::::::::::::<br>miRNA 3' GATAATACGGGGTGTGTACGG 5'	2.5	3417-3437	Abc transporter c family member 3-like

miRNA	Target id	Alignment	Score	Range	Target gene functional annotation
vvi-miR3635-5p	VIT_219s0015g00020.1	target 5' CCGCTATGCCCCACACATGCC 3'<br>: . :::::::::::::::::<br>miRNA 3' GATAATACGGGGTGTGTACGG 5'	2.5	2208-2228	Abc transporter c family member 3-like
vvi-miR3635-5p	VIT_219s0015g00050.1	target 5' CCGCTATGCCCCACACATGCC 3'<br>: . :::::::::::::::::<br>miRNA 3' GATAATACGGGGTGTGTACGG 5'	2.5	3753-3773	Abc transporter c family member 3-like
vvi-miR3635-5p	VIT_219s0015g00060.1	target 5' CCGCTATGCCCCACACATGCC 3'<br>: . :::::::::::::::::<br>miRNA 3' GATAATACGGGGTGTGTACGG 5'	2.5	3486-3506	Multidrug resistance protein abc transporter family
vvi-miR390-5p	VIT_210s0003g01890.1	target 5' GGCGTTCTTCCTCCTGAGCTT 3'<br>::::.: :.::::::::::::<br>miRNA 3' CCGCGATAGGGAGGACTCGAA 5'	2.5	319-339	Lrr receptor-like serine threonine-protein kinase rfk1
vvi-miR390-5p	VIT_216s0098g01090.1	target 5' GGCTCTATACCTCCTGAGCTT 3'<br>::: :::: ::::::::::::<br>miRNA 3' CCGCGATAGGGAGGACTCGAA 5'	3	1621-1641	Leucine-rich repeat receptor-like protein kinase pepr1
vvi-miR393a-5p	VIT_207s0104g01320.1	target 5' GA-CAATGCGATCCCTTTGGA 3'<br>:: ::::::::::::::::::<br>miRNA 3' CTAGTTACGCTAGGGAAACCT 5'	1	1506-1525	Transport inhibitor response 1
vvi-miR393a-5p	VIT_207s0104g01320.2	target 5' GA-CAATGCGATCCCTTTGGA 3'<br>:: ::::::::::::::::::<br>miRNA 3' CTAGTTACGCTAGGGAAACCT 5'	1	1506-1525	Transport inhibitor response 1
vvi-miR393a-5p	VIT_214s0030g01240.1	target 5' GA-CAATGCGATCCCTTTGGA 3'<br>:: ::::::::::::::::::<br>miRNA 3' CTAGTTACGCTAGGGAAACCT 5'	1	1506-1525	Transport inhibitor response 1
vvi-miR393a-5p	VIT_214s0030g01240.2	target 5' GA-CAATGCGATCCCTTTGGA 3'<br>:: ::::::::::::::::::<br>miRNA 3' CTAGTTACGCTAGGGAAACCT 5'	1	1506-1525	Transport inhibitor response 1
vvi-miR393a-5p	VIT_214s0030g01240.3	target 5' GA-CAATGCGATCCCTTTGGA 3'<br>:: ::::::::::::::::::<br>miRNA 3' CTAGTTACGCTAGGGAAACCT 5'	1	1506-1525	Transport inhibitor response 1
vvi-miR393a-5p	VIT_214s0030g01240.4	target 5' GA-CAATGCGATCCCTTTGGA 3'<br>:: ::::::::::::::::::<br>miRNA 3' CTAGTTACGCTAGGGAAACCT 5'	1	1506-1525	Transport inhibitor response 1
vvi-miR393a-5p	VIT_214s0068g01330.1	target 5' GA-CAATGCGATCCCTTTGGA 3'<br>:: ::::::::::::::::::<br>miRNA 3' CTAGTTACGCTAGGGAAACCT 5'	1	1503-1522	F-box family protein
vvi-miR393b	VIT_207s0104g01320.1	target 5' GA-CAATGCGATCCCTTTGGA 3'<br>:: ::::::::::::::::::<br>miRNA 3' CTAGTTACGCTAGGGAAACCT 5'	1	1506-1525	Transport inhibitor response 1
vvi-miR393b	VIT_207s0104g01320.2	target 5' GA-CAATGCGATCCCTTTGGA 3'<br>:: ::::::::::::::::::<br>miRNA 3' CTAGTTACGCTAGGGAAACCT 5'	1	1506-1525	Transport inhibitor response 1

miRNA	Target id	Alignment	Score	Range	Target gene functional annotation
vvi-miR393b	VIT_214s0030g01240.1	target 5' GA-CAATGCGATCCCTTTGGA 3'<br>:: :::::::::::::::::<br>miRNA 3' CTAGTTACGCTAGGGAAACCT 5'	1	1506-1525	Transport inhibitor response 1
vvi-miR393b	VIT_214s0030g01240.2	target 5' GA-CAATGCGATCCCTTTGGA 3'<br>:: :::::::::::::::::<br>miRNA 3' CTAGTTACGCTAGGGAAACCT 5'	1	1506-1525	Transport inhibitor response 1
vvi-miR393b	VIT_214s0030g01240.3	target 5' GA-CAATGCGATCCCTTTGGA 3'<br>:: :::::::::::::::::<br>miRNA 3' CTAGTTACGCTAGGGAAACCT 5'	1	1506-1525	Transport inhibitor response 1
vvi-miR393b	VIT_214s0030g01240.4	target 5' GA-CAATGCGATCCCTTTGGA 3'<br>:: :::::::::::::::::<br>miRNA 3' CTAGTTACGCTAGGGAAACCT 5'	1	1506-1525	Transport inhibitor response 1
vvi-miR393b	VIT_214s0068g01330.1	target 5' GA-CAATGCGATCCCTTTGGA 3'<br>:: :::::::::::::::::<br>miRNA 3' CTAGTTACGCTAGGGAAACCT 5'	1	1503-1522	F-box family protein
vvi-miR394c	VIT_201s0010g03730.1	target 5' GGAGGTTGACAGAATGCCAA 3'<br>:::::: :::::::::::::<br>miRNA 3' CCTCCACCTGTCTTACGGTT 5'	1	939-958	F-box family protein
vvi-miR394c	VIT_201s0010g03730.2	target 5' GGAGGTTGACAGAATGCCAA 3'<br>:::::: :::::::::::::<br>miRNA 3' CCTCCACCTGTCTTACGGTT 5'	1	1131-1150	F-box family protein
vvi-miR395a	VIT_207s0031g00940.1	target 5' AAGTTCTCCCAAACACTTCAA 3'<br>:::::.::::::::::::::<br>miRNA 3' CTCAAGGGGGTTTGTGAAGTC 5'	2.5	37-57	Sulfate bicarbonate oxalate exchanger and transporter sat-1
vvi-miR395a	VIT_207s0031g00940.2	target 5' AAGTTCTCCCAAACACTTCAA 3'<br>:::::.::::::::::::::<br>miRNA 3' CTCAAGGGGGTTTGTGAAGTC 5'	2.5	37-57	Sulfate bicarbonate oxalate exchanger and transporter sat-1
vvi-miR395c-3p	VIT_207s0031g00940.1	target 5' AAGTTCTCCCAAACACTTCAA 3'<br>:::::.::::::::::::::<br>miRNA 3' CTCAAGGGGGTTTGTGAAGTC 5'	2.5	37-57	Sulfate bicarbonate oxalate exchanger and transporter sat-1
vvi-miR395c-3p	VIT_207s0031g00940.2	target 5' AAGTTCTCCCAAACACTTCAA 3'<br>:::::.::::::::::::::<br>miRNA 3' CTCAAGGGGGTTTGTGAAGTC 5'	2.5	37-57	Sulfate bicarbonate oxalate exchanger and transporter sat-1
vvi-miR395c-5p	VIT_214s0066g00910.1	target 5' TGGTGAAGTGTTCAAGGGAAC 3'<br>..:::::::: :::::::::<br>miRNA 3' GTCACTTCACCAGTTCCCTTG 5'	3	1101-1121	Protein
vvi-miR395d	VIT_207s0031g00940.1	target 5' AAGTTCTCCCAAACACTTCAA 3'<br>:::::.::::::::::::::<br>miRNA 3' CTCAAGGGGGTTTGTGAAGTC 5'	2.5	37-57	Sulfate bicarbonate oxalate exchanger and transporter sat-1
vvi-miR395d	VIT_207s0031g00940.2	target 5' AAGTTCTCCCAAACACTTCAA 3'<br>:::::.::::::::::::::<br>miRNA 3' CTCAAGGGGGTTTGTGAAGTC 5'	2.5	37-57	Sulfate bicarbonate oxalate exchanger and transporter sat-1

miRNA	Target id	Alignment	Score	Range	Target gene functional annotation
vvi-miR395e-3p	VIT_207s0031g00940.1	target 5' AAGTTCTCCCAAACACTTCAA 3'<br>:::::.:::::::::::::<br>miRNA 3' CTCAAGGGGGTTTGTGAAGTC 5'	2.5	37-57	Sulfate bicarbonate oxalate exchanger and transporter sat-1
vvi-miR395e-3p	VIT_207s0031g00940.2	target 5' AAGTTCTCCCAAACACTTCAA 3'<br>:::::.:::::::::::::<br>miRNA 3' CTCAAGGGGGTTTGTGAAGTC 5'	2.5	37-57	Sulfate bicarbonate oxalate exchanger and transporter sat-1
vvi-miR395e-5p	VIT_214s0066g00910.1	target 5' TGGTGAAGTGTTCAAGGGAAC 3'<br>..:::::::: ::::::::::<br>miRNA 3' GTCACTTCACCAGTTCCCTTG 5'	3	1101-1121	Protein
vvi-miR395f-3p	VIT_207s0031g00940.1	target 5' AAGTTCTCCCAAACACTTCAA 3'<br>:::::.:::::::::::::<br>miRNA 3' CTCAAGGGGGTTTGTGAAGTC 5'	2.5	37-57	Sulfate bicarbonate oxalate exchanger and transporter sat-1
vvi-miR395f-3p	VIT_207s0031g00940.2	target 5' AAGTTCTCCCAAACACTTCAA 3'<br>:::::.:::::::::::::<br>miRNA 3' CTCAAGGGGGTTTGTGAAGTC 5'	2.5	37-57	Sulfate bicarbonate oxalate exchanger and transporter sat-1
vvi-miR395g-3p	VIT_207s0031g00940.1	target 5' AAGTTCTCCCAAACACTTCAA 3'<br>:::::.:::::::::::::<br>miRNA 3' CTCAAGGGGGTTTGTGAAGTC 5'	2.5	37-57	Sulfate bicarbonate oxalate exchanger and transporter sat-1
vvi-miR395g-3p	VIT_207s0031g00940.2	target 5' AAGTTCTCCCAAACACTTCAA 3'<br>:::::.:::::::::::::<br>miRNA 3' CTCAAGGGGGTTTGTGAAGTC 5'	2.5	37-57	Sulfate bicarbonate oxalate exchanger and transporter sat-1
vvi-miR395h-3p	VIT_207s0031g00940.1	target 5' AAGTTCTCCCAAACACTTCAA 3'<br>:::::.:::::::::::::<br>miRNA 3' CTCAAGGGGGTTTGTGAAGTC 5'	2.5	37-57	Sulfate bicarbonate oxalate exchanger and transporter sat-1
vvi-miR395h-3p	VIT_207s0031g00940.2	target 5' AAGTTCTCCCAAACACTTCAA 3'<br>:::::.:::::::::::::<br>miRNA 3' CTCAAGGGGGTTTGTGAAGTC 5'	2.5	37-57	Sulfate bicarbonate oxalate exchanger and transporter sat-1
vvi-miR395h-5p	VIT_214s0066g00910.1	target 5' TGGTGAAGTGTTCAAGGGAAC 3'<br>..:::::::: ::::::::::<br>miRNA 3' GTCACTTCACCAGTTCCCTTG 5'	3	1101-1121	Protein
vvi-miR395i-3p	VIT_207s0031g00940.1	target 5' AAGTTCTCCCAAACACTTCAA 3'<br>:::::.:::::::::::::<br>miRNA 3' CTCAAGGGGGTTTGTGAAGTC 5'	2.5	37-57	Sulfate bicarbonate oxalate exchanger and transporter sat-1
vvi-miR395i-3p	VIT_207s0031g00940.2	target 5' AAGTTCTCCCAAACACTTCAA 3'<br>:::::.:::::::::::::<br>miRNA 3' CTCAAGGGGGTTTGTGAAGTC 5'	2.5	37-57	Sulfate bicarbonate oxalate exchanger and transporter sat-1
vvi-miR395j-3p	VIT_207s0031g00940.1	target 5' AAGTTCTCCCAAACACTTCAA 3'<br>:::::.:::::::::::::<br>miRNA 3' CTCAAGGGGGTTTGTGAAGTC 5'	2.5	37-57	Sulfate bicarbonate oxalate exchanger and transporter sat-1
vvi-miR395j-3p	VIT_207s0031g00940.2	target 5' AAGTTCTCCCAAACACTTCAA 3'<br>:::::.:::::::::::::<br>miRNA 3' CTCAAGGGGGTTTGTGAAGTC 5'	2.5	37-57	Sulfate bicarbonate oxalate exchanger and transporter sat-1

miRNA	Target id	Alignment	Score	Range	Target gene functional annotation
vvi-miR395k-3p	VIT_207s0031g00940.1	target 5' AAGTTCTCCCAAACACTTCAA 3'<br>:::::.:::::::::::::<br>miRNA 3' CTCAAGGGGGTTTGTGAAGTC 5'	2.5	37-57	Sulfate bicarbonate oxalate exchanger and transporter sat-1
vvi-miR395k-3p	VIT_207s0031g00940.2	target 5' AAGTTCTCCCAAACACTTCAA 3'<br>:::::.:::::::::::::<br>miRNA 3' CTCAAGGGGGTTTGTGAAGTC 5'	2.5	37-57	Sulfate bicarbonate oxalate exchanger and transporter sat-1
vvi-miR395k-5p	VIT_214s0066g00910.1	target 5' TGGTGAAGTGTTCAAGGGAAC 3'<br>..:::::::: ::::::::::<br>miRNA 3' GTCACTTCACCAGTTCCCTTG 5'	3	1101-1121	Protein
vvi-miR395l-3p	VIT_207s0031g00940.1	target 5' AAGTTCTCCCAAACACTTCAA 3'<br>:::::.:::::::::::::<br>miRNA 3' CTCAAGGGGGTTTGTGAAGTC 5'	2.5	37-57	Sulfate bicarbonate oxalate exchanger and transporter sat-1
vvi-miR395l-3p	VIT_207s0031g00940.2	target 5' AAGTTCTCCCAAACACTTCAA 3'<br>:::::.:::::::::::::<br>miRNA 3' CTCAAGGGGGTTTGTGAAGTC 5'	2.5	37-57	Sulfate bicarbonate oxalate exchanger and transporter sat-1
vvi-miR395n	VIT_205s0020g04210.1	target 5' CGAGTTCCTCCAAACTCTTCA 3'<br>::::::::::: ::::::::<br>miRNA 3' TCTCAAGGAGGTCTGAGAAGT 5'	3	339-359	Atp sulfurylase
vvi-miR396a-3p	VIT_200s0208g00090.1	target 5' CTGTCCACCAGCTTTCTTGAGC 3'<br>:. .::: ::::::::::::::<br>miRNA 3' GGAGGGT-GTCGAAAGAACTCG 5'	3	Dec-33	Protein trichome birefringence-like 16
vvi-miR396a-3p	VIT_207s0031g02320.1	target 5' CCGTCCACCAGCTTTCTTGAGC 3'<br>:: .::: ::::::::::::::<br>miRNA 3' GGAGGGT-GTCGAAAGAACTCG 5'	2.5	837-858	Protein
vvi-miR396a-3p	VIT_207s0031g02320.2	target 5' CCGTCCACCAGCTTTCTTGAGC 3'<br>:: .::: ::::::::::::::<br>miRNA 3' GGAGGGT-GTCGAAAGAACTCG 5'	2.5	648-669	Duf231 domain containing family protein
vvi-miR396a-3p	VIT_207s0031g02320.3	target 5' CCGTCCACCAGCTTTCTTGAGC 3'<br>:: .::: ::::::::::::::<br>miRNA 3' GGAGGGT-GTCGAAAGAACTCG 5'	2.5	501-522	Protein trichome birefringence-like 16
vvi-miR396a-5p	VIT_200s0494g00010.1	target 5' CGTTCAAGAAAGCCTGTGGAA 3'<br>::::::::::: ::::::::<br>miRNA 3' TCAAGTTCTTTC-GACACCTT 5'	3	781-801	Transcription activator
vvi-miR396a-5p	VIT_202s0025g02680.1	target 5' CGTTCAAGAAAGCCTGTGGAA 3'<br>::::::::::: ::::::::<br>miRNA 3' TCAAGTTCTTTC-GACACCTT 5'	3	307-327	Uncharacterized protein loc100258227
vvi-miR396a-5p	VIT_202s0025g04910.1	target 5' CGTTCAAGAAAGCATGTGGAA 3'<br>:::::::::::: :::::::<br>miRNA 3' TCAAGTTCTTTCG-ACACCTT 5'	3	445-465	Growth-regulating factor
vvi-miR396a-5p	VIT_207s0191g00220.1	target 5' AACTCAAGGAAGCTGTGGAA 3'<br>: :::::.:::::::::::<br>miRNA 3' TCAAGTTCTTTCGACACCTT 5'	3	494-513	Uncharacterized protein loc100251922

miRNA	Target id	Alignment	Score	Range	Target gene functional annotation
vvi-miR396a-5p	VIT_207s0191g00220.2	target 5' AACTCAAGGAAGCTGTGGAA 3'<br>: :::::.:::::::::::<br>miRNA 3' TCAAGTTCTTTCGACACCTT 5'	3	494-513	Uncharacterized protein loc100251922
vvi-miR396a-5p	VIT_208s0007g03760.1	target 5' CGTTCAAGAAAGCCTGTGGAA 3'<br>::::::::::: ::::::::<br>miRNA 3' TCAAGTTCTTTC-GACACCTT 5'	3	538-558	Growth-regulating factor 4
vvi-miR396a-5p	VIT_209s0002g01350.1	target 5' CGTTCAAGAAAGCCTGTGGAA 3'<br>::::::::::: ::::::::<br>miRNA 3' TCAAGTTCTTTC-GACACCTT 5'	3	334-354	Growth-regulating factor 5
vvi-miR396a-5p	VIT_210s0597g00040.1	target 5' AGATGGAGAAAGCTGTGGAG 3'<br>:: : .:::::::::::::.<br>miRNA 3' TCAAGTTCTTTCGACACCTT 5'	3	1202-1221	Pentatricopeptide repeat-containing protein
vvi-miR396a-5p	VIT_211s0016g01250.1	target 5' CGTTCAAGAAAGCCTGTGGAA 3'<br>::::::::::: ::::::::<br>miRNA 3' TCAAGTTCTTTC-GACACCTT 5'	3	343-363	Growth-regulating factor 5
vvi-miR396a-5p	VIT_215s0048g01740.1	target 5' CGTTCAAGAAAGCCTGTGGAA 3'<br>::::::::::: ::::::::<br>miRNA 3' TCAAGTTCTTTC-GACACCTT 5'	3	439-459	Growth-regulating factor
vvi-miR396a-5p	VIT_215s0048g01740.2	target 5' CGTTCAAGAAAGCCTGTGGAA 3'<br>::::::::::: ::::::::<br>miRNA 3' TCAAGTTCTTTC-GACACCTT 5'	3	460-480	Growth-regulating factor
vvi-miR396a-5p	VIT_215s0048g01740.4	target 5' CGTTCAAGAAAGCCTGTGGAA 3'<br>::::::::::: ::::::::<br>miRNA 3' TCAAGTTCTTTC-GACACCTT 5'	3	115-135	Growth-regulating factor
vvi-miR396a-5p	VIT_215s0048g01740.5	target 5' CGTTCAAGAAAGCCTGTGGAA 3'<br>::::::::::: ::::::::<br>miRNA 3' TCAAGTTCTTTC-GACACCTT 5'	3	115-135	Growth-regulating factor
vvi-miR396a-5p	VIT_215s0048g01740.7	target 5' CGTTCAAGAAAGCCTGTGGAA 3'<br>::::::::::: ::::::::<br>miRNA 3' TCAAGTTCTTTC-GACACCTT 5'	3	115-135	Growth-regulating factor
vvi-miR396a-5p	VIT_216s0039g01450.1	target 5' CGTTCAAGAAAGCCTGTGGAA 3'<br>::::::::::: ::::::::<br>miRNA 3' TCAAGTTCTTTC-GACACCTT 5'	3	361-381	Growth-regulating factor 1
vvi-miR396a-5p	VIT_216s0039g01450.2	target 5' CGTTCAAGAAAGCCTGTGGAA 3'<br>::::::::::: ::::::::<br>miRNA 3' TCAAGTTCTTTC-GACACCTT 5'	3	361-381	Growth-regulating factor 1
vvi-miR396a-5p	VIT_216s0039g01450.3	target 5' CGTTCAAGAAAGCCTGTGGAA 3'<br>::::::::::: ::::::::<br>miRNA 3' TCAAGTTCTTTC-GACACCTT 5'	3	361-381	Growth-regulating factor 1
vvi-miR396a-5p	VIT_216s0039g01450.4	target 5' CGTTCAAGAAAGCCTGTGGAA 3'<br>::::::::::: ::::::::<br>miRNA 3' TCAAGTTCTTTC-GACACCTT 5'	3	172-192	Growth-regulating factor 1

miRNA	Target id	Alignment	Score	Range	Target gene functional annotation
vvi-miR396a-5p	VIT_216s0039g01450.5	target 5' CGTTCAAGAAAGCCTGTGGAA 3'<br>::::::::::: ::::::::<br>miRNA 3' TCAAGTTCTTTC-GACACCTT 5'	3	172-192	Growth-regulating factor 1
vvi-miR396a-5p	VIT_216s0098g01080.1	target 5' CGTTCAAGAAAGCCTGTGGAA 3'<br>::::::::::: ::::::::<br>miRNA 3' TCAAGTTCTTTC-GACACCTT 5'	3	322-342	Protein
vvi-miR396a-5p	VIT_218s0001g08650.1	target 5' CGTTCAAGAAAGCCTGTGGAA 3'<br>::::::::::: ::::::::<br>miRNA 3' TCAAGTTCTTTC-GACACCTT 5'	3	766-786	Uncharacterized protein loc100260890
vvi-miR396b-3p	VIT_201s0011g01220.1	target 5' TTTCCAGCAGCTTTCTTGAAT 3'<br>::::: .:::::::::::::.<br>miRNA 3' AAAGGGTGTCGAAAGAACTTG 5'	2	230-250	Double top
vvi-miR396b-5p	VIT_207s0191g00220.1	target 5' AAACTCAAGGAAGCTGTGGAA 3'<br>:: :::::.:::::::::::<br>miRNA 3' TTCAAGTTCTTTCGACACCTT 5'	3	493-513	Uncharacterized protein loc100251922
vvi-miR396b-5p	VIT_207s0191g00220.2	target 5' AAACTCAAGGAAGCTGTGGAA 3'<br>:: :::::.:::::::::::<br>miRNA 3' TTCAAGTTCTTTCGACACCTT 5'	3	493-513	Uncharacterized protein loc100251922
vvi-miR396c-3p	VIT_205s0077g02140.1	target 5' TGTT-CAGAATTTTCCTGAGC 3'<br>:::: :: ::.:::::::::::<br>miRNA 3' ACAAGGTGTTGAAAGGACTCG 5'	3	1659-1678	Glyoxysomal fatty acid beta-oxidation multifunctional protein mfp-a
vvi-miR396c-5p	VIT_202s0025g02680.1	target 5' CCGTTCAAGAAAGCCTGTGGAA 3'<br>: ::::::::::: ::::::::<br>miRNA 3' GTCAAGTTCTTTC-GACACCTT 5'	3	306-327	Uncharacterized protein loc100258227
vvi-miR396c-5p	VIT_208s0007g03760.1	target 5' CCGTTCAAGAAAGCCTGTGGAA 3'<br>: ::::::::::: ::::::::<br>miRNA 3' GTCAAGTTCTTTC-GACACCTT 5'	3	537-558	Growth-regulating factor 4
vvi-miR396c-5p	VIT_209s0002g01350.1	target 5' CCGTTCAAGAAAGCCTGTGGAA 3'<br>: ::::::::::: ::::::::<br>miRNA 3' GTCAAGTTCTTTC-GACACCTT 5'	3	333-354	Growth-regulating factor 5
vvi-miR396c-5p	VIT_211s0016g01250.1	target 5' CCGTTCAAGAAAGCCTGTGGAA 3'<br>: ::::::::::: ::::::::<br>miRNA 3' GTCAAGTTCTTTC-GACACCTT 5'	3	342-363	Growth-regulating factor 5
vvi-miR396c-5p	VIT_216s0039g01450.1	target 5' CCGTTCAAGAAAGCCTGTGGAA 3'<br>: ::::::::::: ::::::::<br>miRNA 3' GTCAAGTTCTTTC-GACACCTT 5'	3	360-381	Growth-regulating factor 1
vvi-miR396c-5p	VIT_216s0039g01450.2	target 5' CCGTTCAAGAAAGCCTGTGGAA 3'<br>: ::::::::::: ::::::::<br>miRNA 3' GTCAAGTTCTTTC-GACACCTT 5'	3	360-381	Growth-regulating factor 1
vvi-miR396c-5p	VIT_216s0039g01450.3	target 5' CCGTTCAAGAAAGCCTGTGGAA 3'<br>: ::::::::::: ::::::::<br>miRNA 3' GTCAAGTTCTTTC-GACACCTT 5'	3	360-381	Growth-regulating factor 1

miRNA	Target id	Alignment	Score	Range	Target gene functional annotation
vvi-miR396c-5p	VIT_216s0039g01450.4	target 5' CCGTTCAAGAAAGCCTGTGGAA 3'<br>: ::::::::::: ::::::::<br>miRNA 3' GTCAAGTTCTTTC-GACACCTT 5'	3	171-192	Growth-regulating factor 1
vvi-miR396c-5p	VIT_216s0039g01450.5	target 5' CCGTTCAAGAAAGCCTGTGGAA 3'<br>: ::::::::::: ::::::::<br>miRNA 3' GTCAAGTTCTTTC-GACACCTT 5'	3	171-192	Growth-regulating factor 1
vvi-miR396c-5p	VIT_216s0098g01080.1	target 5' CCGTTCAAGAAAGCCTGTGGAA 3'<br>: ::::::::::: ::::::::<br>miRNA 3' GTCAAGTTCTTTC-GACACCTT 5'	3	321-342	Protein
vvi-miR396d-3p	VIT_215s0024g00350.1	target 5' CTTTCCATAGTTTTGTTGAAC 3'<br>:::.:::.::.:::.::::::<br>miRNA 3' GAAGGGTGTCGAAATAACTTG 5'	3	617-637	Tata box binding protein associated factor-like protein
vvi-miR396d-5p	VIT_202s0025g02680.1	target 5' CCGTTCAAGAAAGCCTGTGGAA 3'<br>: ::::::::::: ::::::::<br>miRNA 3' GTCAAGTTCTTTC-GACACCTT 5'	3	306-327	Uncharacterized protein loc100258227
vvi-miR396d-5p	VIT_208s0007g03760.1	target 5' CCGTTCAAGAAAGCCTGTGGAA 3'<br>: ::::::::::: ::::::::<br>miRNA 3' GTCAAGTTCTTTC-GACACCTT 5'	3	537-558	Growth-regulating factor 4
vvi-miR396d-5p	VIT_209s0002g01350.1	target 5' CCGTTCAAGAAAGCCTGTGGAA 3'<br>: ::::::::::: ::::::::<br>miRNA 3' GTCAAGTTCTTTC-GACACCTT 5'	3	333-354	Growth-regulating factor 5
vvi-miR396d-5p	VIT_211s0016g01250.1	target 5' CCGTTCAAGAAAGCCTGTGGAA 3'<br>: ::::::::::: ::::::::<br>miRNA 3' GTCAAGTTCTTTC-GACACCTT 5'	3	342-363	Growth-regulating factor 5
vvi-miR396d-5p	VIT_216s0039g01450.1	target 5' CCGTTCAAGAAAGCCTGTGGAA 3'<br>: ::::::::::: ::::::::<br>miRNA 3' GTCAAGTTCTTTC-GACACCTT 5'	3	360-381	Growth-regulating factor 1
vvi-miR396d-5p	VIT_216s0039g01450.2	target 5' CCGTTCAAGAAAGCCTGTGGAA 3'<br>: ::::::::::: ::::::::<br>miRNA 3' GTCAAGTTCTTTC-GACACCTT 5'	3	360-381	Growth-regulating factor 1
vvi-miR396d-5p	VIT_216s0039g01450.3	target 5' CCGTTCAAGAAAGCCTGTGGAA 3'<br>: ::::::::::: ::::::::<br>miRNA 3' GTCAAGTTCTTTC-GACACCTT 5'	3	360-381	Growth-regulating factor 1
vvi-miR396d-5p	VIT_216s0039g01450.4	target 5' CCGTTCAAGAAAGCCTGTGGAA 3'<br>: ::::::::::: ::::::::<br>miRNA 3' GTCAAGTTCTTTC-GACACCTT 5'	3	171-192	Growth-regulating factor 1
vvi-miR396d-5p	VIT_216s0039g01450.5	target 5' CCGTTCAAGAAAGCCTGTGGAA 3'<br>: ::::::::::: ::::::::<br>miRNA 3' GTCAAGTTCTTTC-GACACCTT 5'	3	171-192	Growth-regulating factor 1
vvi-miR396d-5p	VIT_216s0098g01080.1	target 5' CCGTTCAAGAAAGCCTGTGGAA 3'<br>: ::::::::::: ::::::::<br>miRNA 3' GTCAAGTTCTTTC-GACACCTT 5'	3	321-342	Protein

miRNA	Target id	Alignment	Score	Range	Target gene functional annotation
vvi-miR397a-3p	VIT_204s0008g06470.1	target 5' CAT-ATTGAGTGCAGTGCCAT 3'<br>::: :::::::::::.::::<br>miRNA 3' GTACTAACTCACGTCGCGGTT 5'	3	541-560	Cysteinyl-trna synthetase
vvi-miR397a-3p	VIT_204s0008g06470.2	target 5' CAT-ATTGAGTGCAGTGCCAT 3'<br>::: :::::::::::.::::<br>miRNA 3' GTACTAACTCACGTCGCGGTT 5'	3	832-851	Cysteinyl-trna synthetase
vvi-miR397a-3p	VIT_204s0008g06470.3	target 5' CAT-ATTGAGTGCAGTGCCAT 3'<br>::: :::::::::::.::::<br>miRNA 3' GTACTAACTCACGTCGCGGTT 5'	3	373-392	Cysteinyl-trna synthetase
vvi-miR397a-5p	VIT_204s0069g00950.1	target 5' TGTCAATGCTGCACTCAATGA 3'<br>..::::.::::::::::::::<br>miRNA 3' GTAGTTGCGACGTGAGTTACT 5'	1.5	243-263	Laccase (diphenol oxidase)-like protein
vvi-miR397a-5p	VIT_204s0069g00950.2	target 5' TGTCAATGCTGCACTCAATGA 3'<br>..::::.::::::::::::::<br>miRNA 3' GTAGTTGCGACGTGAGTTACT 5'	1.5	243-263	Laccase (diphenol oxidase)-like protein
vvi-miR397a-5p	VIT_206s0004g03760.1	target 5' CATCAATGCGGCACTCAATGA 3'<br>::::::.:: :::::::::::<br>miRNA 3' GTAGTTGCGACGTGAGTTACT 5'	2.5	705-725	Protein
vvi-miR397a-5p	VIT_206s0004g03760.2	target 5' CATCAATGCGGCACTCAATGA 3'<br>::::::.:: :::::::::::<br>miRNA 3' GTAGTTGCGACGTGAGTTACT 5'	2.5	705-725	Protein
vvi-miR397a-5p	VIT_206s0004g06090.1	target 5' CATCAACGCTGCGCTGAATGA 3'<br>::::::::::::.:: :::::<br>miRNA 3' GTAGTTGCGACGTGAGTTACT 5'	3	663-683	Laccase 1a
vvi-miR397a-5p	VIT_208s0007g00600.1	target 5' GATCAACGCTGCACTCAACGA 3'<br>::::::::::::::::: ::<br>miRNA 3' GTAGTTGCGACGTGAGTTACT 5'	3	693-713	Laccase 110b
vvi-miR397a-5p	VIT_208s0007g00600.2	target 5' GATCAACGCTGCACTCAACGA 3'<br>::::::::::::::::: ::<br>miRNA 3' GTAGTTGCGACGTGAGTTACT 5'	3	192-212	Laccase 110b
vvi-miR397a-5p	VIT_208s0007g00670.1	target 5' AATCAATGCTGCACTCAATGA 3'<br>:::::.::::::::::::::<br>miRNA 3' GTAGTTGCGACGTGAGTTACT 5'	1.5	687-707	Laccase 110c
vvi-miR397a-5p	VIT_208s0007g01910.1	target 5' CATTAACGCTGCACTCAATGA 3'<br>:::.:::::::::::::::::<br>miRNA 3' GTAGTTGCGACGTGAGTTACT 5'	0.5	666-686	Laccase 1a
vvi-miR397a-5p	VIT_208s0007g01910.2	target 5' CATTAACGCTGCACTCAATGA 3'<br>:::.:::::::::::::::::<br>miRNA 3' GTAGTTGCGACGTGAGTTACT 5'	0.5	666-686	Laccase 1a
vvi-miR397a-5p	VIT_208s0040g01790.1	target 5' CGTCAATGCTGCACTCAATGA 3'<br>:.::::.::::::::::::::<br>miRNA 3' GTAGTTGCGACGTGAGTTACT 5'	1	684-704	Laccase (diphenol oxidase)-like protein

miRNA	Target id	Alignment	Score	Range	Target gene functional annotation
vvi-miR397a-5p	VIT_208s0040g01790.2	`target  5' CGTCAATGCTGCACTCAATGA 3'`<br>`            :.::::.::::::::::::::`<br>`miRNA   3' GTAGTTGCGACGTGAGTTACT 5'`	1	198-218	Laccase (diphenol oxidase)-like protein
vvi-miR397a-5p	VIT_208s0040g01790.3	`target  5' CGTCAATGCTGCACTCAATGA 3'`<br>`            :.::::.::::::::::::::`<br>`miRNA   3' GTAGTTGCGACGTGAGTTACT 5'`	1	684-704	Laccase (diphenol oxidase)-like protein
vvi-miR397a-5p	VIT_208s0040g01790.4	`target  5' CGTCAATGCTGCACTCAATGA 3'`<br>`            :.::::.::::::::::::::`<br>`miRNA   3' GTAGTTGCGACGTGAGTTACT 5'`	1	447-467	Laccase (diphenol oxidase)-like protein
vvi-miR397a-5p	VIT_208s0040g01790.5	`target  5' CGTCAATGCTGCACTCAATGA 3'`<br>`            :.::::.::::::::::::::`<br>`miRNA   3' GTAGTTGCGACGTGAGTTACT 5'`	1	39-59	Laccase (diphenol oxidase)-like protein
vvi-miR397a-5p	VIT_213s0019g01930.1	`target  5' GATCAACGCTGCACTCAATGA 3'`<br>`             ::::::::::::::::::::`<br>`miRNA   3' GTAGTTGCGACGTGAGTTACT 5'`	1	696-716	PREDICTED: laccase-17-like [Vitis vinifera]
vvi-miR397a-5p	VIT_213s0019g01940.1	`target  5' GATCAATGCTGCACTCAATGA 3'`<br>`             :::::.::::::::::::::`<br>`miRNA   3' GTAGTTGCGACGTGAGTTACT 5'`	1.5	699-719	Laccase-17
vvi-miR397a-5p	VIT_213s0019g02150.1	`target  5' GATCAATGCTGCACTCAATGA 3'`<br>`             :::::.::::::::::::::`<br>`miRNA   3' GTAGTTGCGACGTGAGTTACT 5'`	1.5	699-719	Laccase-17
vvi-miR397a-5p	VIT_213s0019g02160.1	`target  5' GATCAATGCTGCACTCAATGA 3'`<br>`             :::::.::::::::::::::`<br>`miRNA   3' GTAGTTGCGACGTGAGTTACT 5'`	1.5	699-719	Laccase-17
vvi-miR398a-3p	VIT_201s0127g00520.1	`target  5' GGCAAAGGGGTGGCCTGAGAA 3'`<br>`              ::::::::::.::::::::`<br>`miRNA   3' GGGTTTCCCCACTGGACTCTT 5'`	3	1458-1478	Probable nucleoredoxin 1-like
vvi-miR398a-3p	VIT_201s0127g00540.1	`target  5' GGCAAAGGGGTGGCCTGAGAA 3'`<br>`              ::::::::::.::::::::`<br>`miRNA   3' GGGTTTCCCCACTGGACTCTT 5'`	3	213-233	Probable nucleoredoxin 1-like
vvi-miR398a-3p	VIT_201s0127g00560.1	`target  5' GGCAAAGGGGTGGCCTGAGAA 3'`<br>`              ::::::::::.::::::::`<br>`miRNA   3' GGGTTTCCCCACTGGACTCTT 5'`	3	1461-1481	Probable nucleoredoxin 1-like
vvi-miR399b-3p	VIT_213s0067g03280.1	`target  5' CTGGGCAACTCTTCTTTGGCT 3'`<br>`            : ::::::::::.:::::::`<br>`miRNA   3' GTCCCGTTGAGAGGAAACCGT 5'`	3	248-268	High affinity inorganic phosphate transporter
vvi-miR399c	VIT_213s0067g03280.1	`target  5' CTGGGCAACTCTTCTTTGGCT 3'`<br>`            : ::::::::::.:::::::`<br>`miRNA   3' GTCCCGTTGAGAGGAAACCGT 5'`	3	248-268	High affinity inorganic phosphate transporter
vvi-miR399i	VIT_213s0067g03280.1	`target  5' CTGGGCAACTCTTCTTTGGCT 3'`<br>`            : ::::::::::.:::::::`<br>`miRNA   3' GTCCCGTTGAGAGGAAACCGC 5'`	3	248-268	High affinity inorganic phosphate transporter

miRNA	Target id	Alignment	Score	Range	Target gene functional annotation
vvi-miR403a-3p	VIT_207s0005g01690.1	target 5' GGAGTTTGTGCGTGACTCTAA 3'<br>:::::::::::::: :::::<br>miRNA 3' GCTCAAACACGCACTTAGATT 5'	3	149-169	Unnamed protein product [Vitis vinifera]
vvi-miR403b	VIT_207s0005g01690.1	target 5' GGAGTTTGTGCGTGACTCTAA 3'<br>:::::::::::::: :::::<br>miRNA 3' GCTCAAACACGCACTTAGATT 5'	3	149-169	Unnamed protein product [Vitis vinifera]
vvi-miR403d	VIT_207s0005g01690.1	target 5' GGAGTTTGTGCGTGACTCTAA 3'<br>:::::::::::::: :::::<br>miRNA 3' GCTCAAACACGCACTTAGATT 5'	3	149-169	Unnamed protein product [Vitis vinifera]
vvi-miR403e-3p	VIT_207s0005g01690.1	target 5' GGAGTTTGTGCGTGACTCTAA 3'<br>:::::::::::::: :::::<br>miRNA 3' GCTCAAACACGCACTTAGATT 5'	3	149-169	Unnamed protein product [Vitis vinifera]
vvi-miR403f-3p	VIT_207s0005g01690.1	target 5' GGAGTTTGTGCGTGACTCTAA 3'<br>:::::::::::::: :::::<br>miRNA 3' GCTCAAACACGCACTTAGATT 5'	3	149-169	Unnamed protein product [Vitis vinifera]
vvi-miR403f-5p	VIT_207s0005g01690.1	target 5' TCTTAGATTCACGCACAAACT 3'<br>: ::::: :::::::::::::<br>miRNA 3' AAAATCTCAGTGCGTGTTTGA 5'	2	216-236	Unnamed protein product [Vitis vinifera]
vvi-miR408-3p	VIT_212s0034g01140.1	target 5' GCTGTGGGAAGAGGCAGTGCAG 3'<br>::.. ::::::::::::::::<br>miRNA 3' CGGT-CCCTTCTCCGTCACGTA 5'	3	25-Apr	Basic blue protein
vvi-miR408-3p	VIT_218s0001g15240.1	target 5' GTGAGGGAAGAGGCAGTGCAG 3'<br>:. :::::::::::::::::<br>miRNA 3' CGGTCCCTTCTCCGTCACGTA 5'	2.5	146-166	Basic blue protein
vvi-miR477	VIT_201s0010g02270.1	target 5' ATATTGGAAGCCTTTGAGGGA 3'<br>: :::::::::::::::::::<br>miRNA 3' TTTAACCTTCGGAAACTCCCT 5'	1	778-798	Gras family transcription factor
vvi-miR477	VIT_217s0000g10300.1	target 5' ATATTGGAAGCCTTTGAGGGA 3'<br>: :::::::::::::::::::<br>miRNA 3' TTTAACCTTCGGAAACTCCCT 5'	1	748-768	Gras family transcription factor
vvi-miR477b-5p	VIT_201s0010g02270.1	target 5' TGGAAGCCTTTGAGGGAGAGA 3'<br>..:::::::.::::::::::<br>miRNA 3' GTCTTCGGGAACTCCCTCTCA 5'	3	782-802	Gras family transcription factor
vvi-miR477b-5p	VIT_217s0000g10300.1	target 5' TGGAAGCCTTTGAGGGAGAGA 3'<br>..:::::::.::::::::::<br>miRNA 3' GTCTTCGGGAACTCCCTCTCA 5'	3	752-772	Gras family transcription factor
vvi-miR479-3p	VIT_218s0001g03310.1	target 5' GGGTGATATTGGTTCGGCTCA 3'<br>:.::::::::::::::::::<br>miRNA 3' CTCACTATAACCAAGCCGAGC 5'	1.5	374-394	Gras family protein
vvi-miR535a-3p	VIT_200s0358g00010.1	target 5' CTTGACAACGAGAGAGAGCAC 3'<br>::::: :::::::::::::<br>miRNA 3' ATACTGTCGCTCTCTCTCGTG 5'	3	72-92	Unnamed protein product [Vitis vinifera]

miRNA	Target id	Alignment	Score	Range	Target gene functional annotation
vvi-miR535a-3p	VIT_207s0104g00030.1	target 5' CTTGACAACGAGAGAGAGCAC 3'<br>::::: :::::::::::::<br>miRNA 3' ATACTGTCGCTCTCTCTCGTG 5'	3	72-92	Unnamed protein product [Vitis vinifera]
vvi-miR535a-5p	VIT_200s0358g00010.1	target 5' GCGTGCTCTCTCTCGCTGTCA 3'<br>::::::::::::::: :::::<br>miRNA 3' CGCACGAGAGAGAGCAACAGT 5'	2	132-152	Unnamed protein product [Vitis vinifera]
vvi-miR535b-3p	VIT_200s0358g00010.1	target 5' CTTGACAACGAGAGAGAGCAC 3'<br>::::: :::::::::::::<br>miRNA 3' ATACTGTCGCTCTCTCTCGTG 5'	3	72-92	Unnamed protein product [Vitis vinifera]
vvi-miR535b-3p	VIT_207s0104g00030.1	target 5' CTTGACAACGAGAGAGAGCAC 3'<br>::::: :::::::::::::<br>miRNA 3' ATACTGTCGCTCTCTCTCGTG 5'	3	72-92	Unnamed protein product [Vitis vinifera]
vvi-miR535b-5p	VIT_200s0358g00010.1	target 5' GCGTGCTCTCTCTCGCTGTCA 3'<br>::::::::::::::: :::::<br>miRNA 3' CGCACGAGAGAGAGCAACAGT 5'	2	132-152	Unnamed protein product [Vitis vinifera]
vvi-miR535c-5p	VIT_200s0358g00010.1	target 5' GCGTGCTCTCTCTCGCTGTCA 3'<br>::::::::::::::: :::::<br>miRNA 3' CGCACGAGAGAGAGCAACAGT 5'	2	132-152	Unnamed protein product [Vitis vinifera]
vvi-miRC159d	VIT_201s0127g00480.1	target 5' AGAGCTACCTTCAAAACCAAA 3'<br>:::::: ::::: ::::::::<br>miRNA 3' TCTCGAGGGAAG-TTTGGTTT 5'	3	261-281	Uncharacterized protein
vvi-miRC159d	VIT_201s0127g00480.2	target 5' AGAGCTACCTTCAAAACCAAA 3'<br>:::::: ::::: ::::::::<br>miRNA 3' TCTCGAGGGAAG-TTTGGTTT 5'	3	261-281	Uncharacterized protein
vvi-miRC159d	VIT_201s0127g00480.3	target 5' AGAGCTACCTTCAAAACCAAA 3'<br>:::::: ::::: ::::::::<br>miRNA 3' TCTCGAGGGAAG-TTTGGTTT 5'	3	261-281	Uncharacterized protein
vvi-miRC159d	VIT_210s0003g00840.1	target 5' AACGCACCCTTCAAACCAAA 3'<br>: :: ::::::::::::::<br>miRNA 3' TCTCGAGGGAAGTTTGGTTT 5'	3	588-607	Unnamed protein product [Vitis vinifera]
vvi-miRC159d	VIT_219s0014g01700.1	target 5' AGAGCCCCCTTCAAACCAAA 3'<br>::::: ::::::::::::::<br>miRNA 3' TCTCGAGGGAAGTTTGGTTT 5'	1	660-679	Uncharacterized protein loc100505457
vvi-miRC159d	VIT_219s0090g00590.1	target 5' AGAGCTCCCTTCAAGCCAAT 3'<br>::::::::::::::.::::<br>miRNA 3' TCTCGAGGGAAGTTTGGTTT 5'	2	846-865	R2r3-myb transcription
vvi-miRC159e	VIT_201s0127g00480.1	target 5' AGAGCTACCTTCAAAACCAAA 3'<br>:::::: ::::: ::::::::<br>miRNA 3' TCTCGAGGGAAG-TTTGGTTT 5'	3	261-281	Uncharacterized protein
vvi-miRC159e	VIT_201s0127g00480.2	target 5' AGAGCTACCTTCAAAACCAAA 3'<br>:::::: ::::: ::::::::<br>miRNA 3' TCTCGAGGGAAG-TTTGGTTT 5'	3	261-281	Uncharacterized protein

miRNA	Target id	Alignment	Score	Range	Target gene functional annotation
vvi-miRC159e	VIT_201s0127g00480.3	target 5' AGAGCTACCTTCAAAACCAAA 3'<br>:::::: ::::: ::::::::<br>miRNA 3' TCTCGAGGGAAG-TTTGGTTT 5'	3	261-281	Uncharacterized protein
vvi-miRC159e	VIT_210s0003g00840.1	target 5' AACGCACCCTTCAAACCAAA 3'<br>: :: ::::::::::::::<br>miRNA 3' TCTCGAGGGAAGTTTGGTTT 5'	3	588-607	Unnamed protein product [Vitis vinifera]
vvi-miRC159e	VIT_219s0014g01700.1	target 5' AGAGCCCCCTTCAAACCAAA 3'<br>::::: ::::::::::::::<br>miRNA 3' TCTCGAGGGAAGTTTGGTTT 5'	1	660-679	Uncharacterized protein loc100505457
vvi-miRC159e	VIT_219s0090g00590.1	target 5' AGAGCTCCCTTCAAGCCAAT 3'<br>::::::::::::::.::::<br>miRNA 3' TCTCGAGGGAAGTTTGGTTT 5'	2	846-865	R2r3-myb transcription
vvi-miRC159f	VIT_200s0226g00070.1	target 5' CAGAACTCTCTTCACTCCAA 3'<br>::: :::.::::::::::<br>miRNA 3' ATCTCGAGGGAAGTGAGGTT 5'	3	25-Jun	Gras family transcription factor
vvi-miRC159f	VIT_206s0009g02480.1	target 5' TGGAGCTCCCTTCTCTCCAA 3'<br>:.:::::::::: ::::::<br>miRNA 3' ATCTCGAGGGAAGTGAGGTT 5'	2.5	959-978	Transcription factor gamyb-like
vvi-miRC159f	VIT_206s0009g02480.2	target 5' TGGAGCTCCCTTCTCTCCAA 3'<br>:.:::::::::: ::::::<br>miRNA 3' ATCTCGAGGGAAGTGAGGTT 5'	2.5	959-978	Transcription factor gamyb-like
vvi-miRC159f	VIT_206s0009g02480.3	target 5' TGGAGCTCCCTTCTCTCCAA 3'<br>:.:::::::::: ::::::<br>miRNA 3' ATCTCGAGGGAAGTGAGGTT 5'	2.5	524-543	Transcription factor gamyb-like
vvi-miRC159f	VIT_206s0009g02480.4	target 5' TGGAGCTCCCTTCTCTCCAA 3'<br>:.:::::::::: ::::::<br>miRNA 3' ATCTCGAGGGAAGTGAGGTT 5'	2.5	959-978	Transcription factor gamyb-like
vvi-miRC159f	VIT_213s0067g01630.1	target 5' TGGAGCTCCCTTCACTCCAA 3'<br>:.:::::::::::::::::<br>miRNA 3' ATCTCGAGGGAAGTGAGGTT 5'	0.5	956-975	Transcription factor gamyb-like
vvi-miRC166i-3p	VIT_204s0008g03250.1	target 5' CTGGGATGAAGCCTGGTCCGG 3'<br>::.:::::::::::::::.<br>miRNA 3' CCCCTTACTTCGGACCAGGCT 5'	3	557-577	Protein
vvi-miRC166i-3p	VIT_204s0008g03250.2	target 5' CTGGGATGAAGCCTGGTCCGG 3'<br>::.:::::::::::::::.<br>miRNA 3' CCCCTTACTTCGGACCAGGCT 5'	3	557-577	Class iii hd-zip protein 8
vvi-miRC166i-3p	VIT_206s0004g02800.1	target 5' CTGGGATGAAGCCTGGTCCGG 3'<br>::.:::::::::::::::.<br>miRNA 3' CCCCTTACTTCGGACCAGGCT 5'	3	563-583	Homeobox leucine-zipper protein
vvi-miRC166i-3p	VIT_206s0004g02800.2	target 5' CTGGGATGAAGCCTGGTCCGG 3'<br>::.:::::::::::::::.<br>miRNA 3' CCCCTTACTTCGGACCAGGCT 5'	3	563-583	Homeobox leucine-zipper protein

miRNA	Target id	Alignment	Score	Range	Target gene functional annotation
vvi-miRC166i-3p	VIT_206s0004g02800.3	target 5' CTGGGATGAAGCCTGGTCCGG 3'<br>::.:::::::::::::::.<br>miRNA 3' CCCCTTACTTCGGACCAGGCT 5'	3	563-583	Homeobox leucine-zipper protein
vvi-miRC166i-3p	VIT_209s0002g03740.1	target 5' CTGGAATGAAGCCTGGTCCGG 3'<br>::::::::::::::::::.<br>miRNA 3' CCCCTTACTTCGGACCAGGCT 5'	2.5	551-571	Homeobox-leucine zipper protein athb-15
vvi-miRC166i-3p	VIT_210s0003g04670.1	target 5' TTGGGATGAAGCCTGGTCCGG 3'<br>::.:::::::::::::::.<br>miRNA 3' CCCCTTACTTCGGACCAGGCT 5'	3	566-586	Homeobox-leucine zipper protein hox32-like
vvi-miRC166i-3p	VIT_210s0003g04670.2	target 5' TTGGGATGAAGCCTGGTCCGG 3'<br>::.:::::::::::::::.<br>miRNA 3' CCCCTTACTTCGGACCAGGCT 5'	3	293-313	Homeobox-leucine zipper protein hox32-like
vvi-miRC166i-3p	VIT_210s0003g04670.3	target 5' TTGGGATGAAGCCTGGTCCGG 3'<br>::.:::::::::::::::.<br>miRNA 3' CCCCTTACTTCGGACCAGGCT 5'	3	25-May	Homeobox-leucine zipper protein hox32-like
vvi-miRC166i-3p	VIT_213s0019g04320.1	target 5' CTGGGATGAAGCCTGGTCCGG 3'<br>::.:::::::::::::::.<br>miRNA 3' CCCCTTACTTCGGACCAGGCT 5'	3	263-283	Homeobox-leucine zipper protein revoluta
vvi-miRC166i-3p	VIT_213s0019g04320.2	target 5' CTGGGATGAAGCCTGGTCCGG 3'<br>::.:::::::::::::::.<br>miRNA 3' CCCCTTACTTCGGACCAGGCT 5'	3	263-283	Homeobox-leucine zipper protein revoluta
vvi-miRC166i-3p	VIT_213s0019g04320.3	target 5' CTGGGATGAAGCCTGGTCCGG 3'<br>::.:::::::::::::::.<br>miRNA 3' CCCCTTACTTCGGACCAGGCT 5'	3	263-283	Homeobox-leucine zipper protein revoluta
vvi-miRC166i-3p	VIT_213s0019g04320.4	target 5' CTGGGATGAAGCCTGGTCCGG 3'<br>::.:::::::::::::::.<br>miRNA 3' CCCCTTACTTCGGACCAGGCT 5'	3	263-283	Homeobox-leucine zipper protein revoluta
vvi-miRC166i-3p	VIT_213s0019g04320.5	target 5' CTGGGATGAAGCCTGGTCCGG 3'<br>::.:::::::::::::::.<br>miRNA 3' CCCCTTACTTCGGACCAGGCT 5'	3	173-193	Homeobox-leucine zipper protein revoluta
vvi-miRC166i-3p	VIT_213s0019g04320.6	target 5' CTGGGATGAAGCCTGGTCCGG 3'<br>::.:::::::::::::::.<br>miRNA 3' CCCCTTACTTCGGACCAGGCT 5'	3	263-283	Homeobox-leucine zipper protein revoluta
vvi-miRC166i-3p	VIT_213s0019g04320.7	target 5' CTGGGATGAAGCCTGGTCCGG 3'<br>::.:::::::::::::::.<br>miRNA 3' CCCCTTACTTCGGACCAGGCT 5'	3	263-283	Homeobox-leucine zipper protein revoluta
vvi-miRC171h-3p	VIT_202s0154g00400.1	target 5' GATATTGGCGCGGCTCAATCA 3'<br>:::::::::.:::::::::::<br>miRNA 3' CTATAACCGTGCCGAGTTAGT 5'	1	1221-1241	Gras family transcription factor
vvi-miRC171h-3p	VIT_204s0023g01380.1	target 5' GATATTGGCGCGGCTCAATCA 3'<br>:::::::::.:::::::::::<br>miRNA 3' CTATAACCGTGCCGAGTTAGT 5'	1	588-608	Gras family transcription factor

miRNA	Target id	Alignment	Score	Range	Target gene functional annotation
vvi-miRC171h-3p	VIT_215s0048g00270.1	target 5' GATATTGGCGCGGCTCAATCA 3'<br>:::::::::.:::::::::::<br>miRNA 3' CTATAACCGTGCCGAGTTAGT 5'	1	1233-1253	Gras family transcription factor
vvi-miRC171j	VIT_202s0154g00400.1	target 5' GATATTGGCGCGGCTCAATCA 3'<br>:::::::::.:::::::::::<br>miRNA 3' CTATAACCGTGCCGAGTTAGT 5'	1	1221-1241	Gras family transcription factor
vvi-miRC171j	VIT_204s0023g01380.1	target 5' GATATTGGCGCGGCTCAATCA 3'<br>:::::::::.:::::::::::<br>miRNA 3' CTATAACCGTGCCGAGTTAGT 5'	1	588-608	Gras family transcription factor
vvi-miRC171j	VIT_215s0048g00270.1	target 5' GATATTGGCGCGGCTCAATCA 3'<br>:::::::::.:::::::::::<br>miRNA 3' CTATAACCGTGCCGAGTTAGT 5'	1	1233-1253	Gras family transcription factor
vvi-miRC172e-3p	VIT_200s0184g00102.1	target 5' GCATTCATGATCAAGATTCTCA 3'<br>::: ::: :::::::::::::<br>miRNA 3' CGT-CGTAGTAGTTCTAAGAGT 5'	3	215-236	Ap2 erf and b3 domain-containing transcription factor rav2
vvi-miRC172e-3p	VIT_206s0004g03590.1	target 5' GCAGCATCATCAGGATTCTCA 3'<br>::::::::::::.::::::::<br>miRNA 3' CGTCGTAGTAGTTCTAAGAGT 5'	1	1336-1356	Ethylene-responsive transcription factor rap2-7-like
vvi-miRC172e-3p	VIT_206s0004g03590.3	target 5' GCAGCATCATCAGGATTCTCA 3'<br>::::::::::::.::::::::<br>miRNA 3' CGTCGTAGTAGTTCTAAGAGT 5'	1	1291-1311	Ethylene-responsive transcription factor rap2-7-like
vvi-miRC172e-3p	VIT_207s0031g00220.1	target 5' GCAGCATCATCAGGATTCCCA 3'<br>::::::::::::.::::: ::<br>miRNA 3' CGTCGTAGTAGTTCTAAGAGT 5'	3	1432-1452	Transcription factor apetala2
vvi-miRC172e-3p	VIT_207s0031g00220.2	target 5' GCAGCATCATCAGGATTCCCA 3'<br>::::::::::::.::::: ::<br>miRNA 3' CGTCGTAGTAGTTCTAAGAGT 5'	3	1444-1464	Transcription factor apetala2
vvi-miRC172e-3p	VIT_208s0040g03180.1	target 5' GCAGCATCATCAGGATTCTTG 3'<br>::::::::::::.::::::..<br>miRNA 3' CGTCGTAGTAGTTCTAAGAGT 5'	2.5	1333-1353	Ap2 domain-containing transcription factor
vvi-miRC172e-5p	VIT_216s0050g00455.1	target 5' TATGAAGCTTGATGATGCTAT 3'<br>: :::: :::::::::::::.<br>miRNA 3' ACACTTAGAACTACTACGATG 5'	2.5	249-269	Rna polymerase iii subunit rpc82 family protein
vvi-miRC172f-3p	VIT_200s0184g00102.1	target 5' GCATTCATGATCAAGATTCTCA 3'<br>::: ::: :::::::::::::<br>miRNA 3' CGT-CGTAGTAGTTCTAAGAGT 5'	3	215-236	Ap2 erf and b3 domain-containing transcription factor rav2
vvi-miRC172f-3p	VIT_206s0004g03590.1	target 5' GCAGCATCATCAGGATTCTCA 3'<br>::::::::::::.::::::::<br>miRNA 3' CGTCGTAGTAGTTCTAAGAGT 5'	1	1336-1356	Ethylene-responsive transcription factor rap2-7-like
vvi-miRC172f-3p	VIT_206s0004g03590.3	target 5' GCAGCATCATCAGGATTCTCA 3'<br>::::::::::::.::::::::<br>miRNA 3' CGTCGTAGTAGTTCTAAGAGT 5'	1	1291-1311	Ethylene-responsive transcription factor rap2-7-like

miRNA	Target id	Alignment	Score	Range	Target gene functional annotation
vvi-miRC172f-3p	VIT_207s0031g00220.1	target 5' GCAGCATCATCAGGATTCCCA 3'<br>:::::::::::.:::::: ::<br>miRNA 3' CGTCGTAGTAGTTCTAAGAGT 5'	3	1432-1452	Transcription factor apetala2
vvi-miRC172f-3p	VIT_207s0031g00220.2	target 5' GCAGCATCATCAGGATTCCCA 3'<br>:::::::::::.:::::: ::<br>miRNA 3' CGTCGTAGTAGTTCTAAGAGT 5'	3	1444-1464	Transcription factor apetala2
vvi-miRC172f-3p	VIT_208s0040g03180.1	target 5' GCAGCATCATCAGGATTCTTG 3'<br>:::::::::::.:::::::..<br>miRNA 3' CGTCGTAGTAGTTCTAAGAGT 5'	2.5	1333-1353	Ap2 domain-containing transcription factor
vvi-miRC172f-5p	VIT_216s0050g00455.1	target 5' TATGAAGCTTGATGATGCTAT 3'<br>: :::: :::::::::::::.<br>miRNA 3' ACACTTAGAACTACTACGATG 5'	2.5	249-269	Rna polymerase iii subunit rpc82 family protein
vvi-miRC2275	VIT_210s0003g02830.1	target 5' GGAGAAATTGGAGGAAACTGAA 3'<br>:::: :::::::::::::.::<br>miRNA 3' ACTCTATAACCTCCTTTGATTT 5'	3	531-552	O-acetyltransferase-like protein
vvi-miRC2275	VIT_210s0003g02830.2	target 5' GGAGAAATTGGAGGAAACTGAA 3'<br>:::: :::::::::::::.::<br>miRNA 3' ACTCTATAACCTCCTTTGATTT 5'	3	531-552	O-acetyltransferase-like protein
vvi-miRC2275	VIT_210s0003g02830.3	target 5' GGAGAAATTGGAGGAAACTGAA 3'<br>:::: :::::::::::::.::<br>miRNA 3' ACTCTATAACCTCCTTTGATTT 5'	3	399-420	Cas1 domain-containing protein 1-like
vvi-miRC3627a	VIT_209s0018g02130.1	target 5' GGCGCCATCTCTCGTGCGGCAG 3'<br>:: ::::::::::::::::::.<br>miRNA 3' CCACGGTAGAGAGCACGCCGTT 5'	1.5	149-170	Calcium-transporting atpase plasma membrane-type-like
vvi-miRC3629e	VIT_200s0524g00010.1	target 5' CAGTTTCTCGGCAACCAAACA 3'<br>:: :::::::.::::::::::<br>miRNA 3' GTAAAAGAGCTGTTGGTTTGT 5'	2	280-300	Protein
vvi-miRC3629e	VIT_208s0007g00890.1	target 5' CATGTTCTCG-CAACCAAACA 3'<br>::: :::::: ::::::::::<br>miRNA 3' GTAAAAGAGCTGTTGGTTTGT 5'	3	86-105	Tropinone reductase homolog at1g07440
vvi-miRC3629e	VIT_215s0048g02420.1	target 5' AGTTTTCTCGGCAACCAAACG 3'<br>.::::::::.:::::::::.<br>miRNA 3' GTAAAAGAGCTGTTGGTTTGT 5'	1	62-82	Hypothetical protein
vvi-miRC3629e	VIT_217s0000g07510.3	target 5' TATTTCCTCGGCAACCAAACG 3'<br>.:::: ::::.:::::::::.<br>miRNA 3' GTAAAAGAGCTGTTGGTTTGT 5'	3	598-618	Myb transcription factor
vvi-miRC390a-5p	VIT_210s0003g01890.1	target 5' GGCGTTCTTCCTCCTGAGCTT 3'<br>::::.: :.::::::::::::<br>miRNA 3' CCGCGATAGGGAGGACTCGAA 5'	2.5	319-339	Lrr receptor-like serine threonine-protein kinase rfk1
vvi-miRC390a-5p	VIT_216s0098g01090.1	target 5' GGCTCTATACCTCCTGAGCTT 3'<br>::: :::: ::::::::::::<br>miRNA 3' CCGCGATAGGGAGGACTCGAA 5'	3	1621-1641	Leucine-rich repeat receptor-like protein kinase pepr1

miRNA	Target id	Alignment	Score	Range	Target gene functional annotation
vvi-miRC396e-5p	VIT_200s0179g00260.1	target 5' GGGGTCAAGAGAGCCGTGGAA 3'<br>..: ::::::.::::::::::<br>miRNA 3' TTCAAGTTCTTTCGGCACCTT 5'	3	1978-1998	Calcium-transporting atpase plasma membrane-type-like
vvi-miRC477c-3p	VIT_217s0000g02070.1	target 5' GGT-CTCCACCGGCTTCCAAG 3'<br>::: :.::::::::::::::<br>miRNA 3' CCAGGGGGTGGCCGAAGGTTG 5'	2.5	135-154	Deoxyhypusine synthase
vvi-miRC477c-3p	VIT_217s0000g02070.2	target 5' GGT-CTCCACCGGCTTCCAAG 3'<br>::: :.::::::::::::::<br>miRNA 3' CCAGGGGGTGGCCGAAGGTTG 5'	2.5	135-154	Deoxyhypusine synthase
vvi-miRC477c-3p	VIT_217s0000g02070.3	target 5' GGT-CTCCACCGGCTTCCAAG 3'<br>::: :.::::::::::::::<br>miRNA 3' CCAGGGGGTGGCCGAAGGTTG 5'	2.5	135-154	Deoxyhypusine synthase
vvi-miRC477c-5p	VIT_201s0010g02270.1	target 5' ATATTGGAAGCCTTTGAGGGA 3'<br>: :::::::::::::::::::<br>miRNA 3' TTTAACCTTCGGAAACTCCCT 5'	1	778-798	Gras family transcription factor
vvi-miRC477c-5p	VIT_217s0000g10300.1	target 5' ATATTGGAAGCCTTTGAGGGA 3'<br>: :::::::::::::::::::<br>miRNA 3' TTTAACCTTCGGAAACTCCCT 5'	1	748-768	Gras family transcription factor
vvi-miRC477d-3p	VIT_217s0000g02070.1	target 5' GGT-CTCCACCGGCTTCCAAG 3'<br>::: :.::::::::::::::<br>miRNA 3' CCAGGGGGTGGCCGAAGGTTG 5'	2.5	135-154	Deoxyhypusine synthase
vvi-miRC477d-3p	VIT_217s0000g02070.2	target 5' GGT-CTCCACCGGCTTCCAAG 3'<br>::: :.::::::::::::::<br>miRNA 3' CCAGGGGGTGGCCGAAGGTTG 5'	2.5	135-154	Deoxyhypusine synthase
vvi-miRC477d-3p	VIT_217s0000g02070.3	target 5' GGT-CTCCACCGGCTTCCAAG 3'<br>::: :.::::::::::::::<br>miRNA 3' CCAGGGGGTGGCCGAAGGTTG 5'	2.5	135-154	Deoxyhypusine synthase
vvi-miRC477d-5p	VIT_201s0010g02270.1	target 5' ATATTGGAAGCCTTTGAGGGA 3'<br>: :::::::::::::::::::<br>miRNA 3' TTTAACCTTCGGAAACTCCCT 5'	1	778-798	Gras family transcription factor
vvi-miRC477d-5p	VIT_217s0000g10300.1	target 5' ATATTGGAAGCCTTTGAGGGA 3'<br>: :::::::::::::::::::<br>miRNA 3' TTTAACCTTCGGAAACTCCCT 5'	1	748-768	Gras family transcription factor
vvi-miRC477e-3p	VIT_217s0000g02070.1	target 5' GGT-CTCCACCGGCTTCCAAG 3'<br>::: :.::::::::::::::<br>miRNA 3' CCAGGGGGTGGCCGAAGGTTG 5'	2.5	135-154	Deoxyhypusine synthase
vvi-miRC477e-3p	VIT_217s0000g02070.2	target 5' GGT-CTCCACCGGCTTCCAAG 3'<br>::: :.::::::::::::::<br>miRNA 3' CCAGGGGGTGGCCGAAGGTTG 5'	2.5	135-154	Deoxyhypusine synthase
vvi-miRC477e-3p	VIT_217s0000g02070.3	target 5' GGT-CTCCACCGGCTTCCAAG 3'<br>::: :.::::::::::::::<br>miRNA 3' CCAGGGGGTGGCCGAAGGTTG 5'	2.5	135-154	Deoxyhypusine synthase

miRNA	Target id	Alignment	Score	Range	Target gene functional annotation
vvi-miRC477e-5p	VIT_201s0010g02270.1	target 5' ATATTGGAAGCCTTTGAGGGA 3'<br>: :::::::::::::::::::<br>miRNA 3' TTTAACCTTCGGAAACTCCCT 5'	1	778-798	Gras family transcription factor
vvi-miRC477e-5p	VIT_217s0000g10300.1	target 5' ATATTGGAAGCCTTTGAGGGA 3'<br>: :::::::::::::::::::<br>miRNA 3' TTTAACCTTCGGAAACTCCCT 5'	1	748-768	Gras family transcription factor
vvi-miRC477f-5p	VIT_201s0010g02270.1	target 5' ATATTGGAAGCCTTTGAGGGA 3'<br>: :::::::::::::::::::<br>miRNA 3' TTTAACCTTCGGAAACTCCCT 5'	1	778-798	Gras family transcription factor
vvi-miRC477f-5p	VIT_217s0000g10300.1	target 5' ATATTGGAAGCCTTTGAGGGA 3'<br>: :::::::::::::::::::<br>miRNA 3' TTTAACCTTCGGAAACTCCCT 5'	1	748-768	Gras family transcription factor
vvi-miRC477g-3p	VIT_217s0000g02070.1	target 5' GGT-CTCCACCGGCTTCCAAG 3'<br>::: :.::::::::::::::<br>miRNA 3' CCAGGGGGTGGCCGAAGGTTG 5'	2.5	135-154	Deoxyhypusine synthase
vvi-miRC477g-3p	VIT_217s0000g02070.2	target 5' GGT-CTCCACCGGCTTCCAAG 3'<br>::: :.::::::::::::::<br>miRNA 3' CCAGGGGGTGGCCGAAGGTTG 5'	2.5	135-154	Deoxyhypusine synthase
vvi-miRC477g-3p	VIT_217s0000g02070.3	target 5' GGT-CTCCACCGGCTTCCAAG 3'<br>::: :.::::::::::::::<br>miRNA 3' CCAGGGGGTGGCCGAAGGTTG 5'	2.5	135-154	Deoxyhypusine synthase
vvi-miRC477g-5p	VIT_201s0010g02270.1	target 5' ATATTGGAAGCCTTTGAGGGA 3'<br>: :::::::::::::::::::<br>miRNA 3' TTTAACCTTCGGAAACTCCCT 5'	1	778-798	Gras family transcription factor
vvi-miRC477g-5p	VIT_217s0000g10300.1	target 5' ATATTGGAAGCCTTTGAGGGA 3'<br>: :::::::::::::::::::<br>miRNA 3' TTTAACCTTCGGAAACTCCCT 5'	1	748-768	Gras family transcription factor
vvi-miRC477h-3p	VIT_217s0000g02070.1	target 5' GGT-CTCCACCGGCTTCCAAG 3'<br>::: :.::::::::::::::<br>miRNA 3' CCAGGGGGTGGCCGAAGGTTG 5'	2.5	135-154	Deoxyhypusine synthase
vvi-miRC477h-3p	VIT_217s0000g02070.2	target 5' GGT-CTCCACCGGCTTCCAAG 3'<br>::: :.::::::::::::::<br>miRNA 3' CCAGGGGGTGGCCGAAGGTTG 5'	2.5	135-154	Deoxyhypusine synthase
vvi-miRC477h-3p	VIT_217s0000g02070.3	target 5' GGT-CTCCACCGGCTTCCAAG 3'<br>::: :.::::::::::::::<br>miRNA 3' CCAGGGGGTGGCCGAAGGTTG 5'	2.5	135-154	Deoxyhypusine synthase
vvi-miRC477h-5p	VIT_201s0010g02270.1	target 5' ATATTGGAAGCCTTTGAGGGA 3'<br>: :::::::::::::::::::<br>miRNA 3' TTTAACCTTCGGAAACTCCCT 5'	1	778-798	Gras family transcription factor
vvi-miRC477h-5p	VIT_217s0000g10300.1	target 5' ATATTGGAAGCCTTTGAGGGA 3'<br>: :::::::::::::::::::<br>miRNA 3' TTTAACCTTCGGAAACTCCCT 5'	1	748-768	Gras family transcription factor

miRNA	Target id	Alignment	Score	Range	Target gene functional annotation
vvi-miRC530-3p	VIT_200s0267g00060.1	target 5' TCTTCACCTTCACCT-CACCT 3'<br>::: ::::::::::: :::::<br>miRNA 3' AGACGTGGAAGTGGACGTGGA 5'	3	31-Dec	Unnamed protein product [Vitis vinifera]
vvi-miRC530-3p	VIT_201s0011g03410.1	target 5' ACTGCACCTGCACCTGCACCT 3'<br>:::::::: ::::::::::<br>miRNA 3' AGACGTGGAAGTGGACGTGGA 5'	3	262-282	Uv excision repair protein
vvi-miRC530-3p	VIT_201s0011g03410.2	target 5' ACTGCACCTGCACCTGCACCT 3'<br>:::::::: ::::::::::<br>miRNA 3' AGACGTGGAAGTGGACGTGGA 5'	3	262-282	Dna repair protein rad23-3
vvi-miRC530-3p	VIT_201s0011g03410.3	target 5' ACTGCACCTGCACCTGCACCT 3'<br>:::::::: ::::::::::<br>miRNA 3' AGACGTGGAAGTGGACGTGGA 5'	3	340-360	Dna repair protein rad23-3
vvi-miRC530-3p	VIT_201s0011g03410.4	target 5' ACTGCACCTGCACCTGCACCT 3'<br>:::::::: ::::::::::<br>miRNA 3' AGACGTGGAAGTGGACGTGGA 5'	3	340-360	Dna repair protein rad23-3
vvi-miRC530-3p	VIT_202s0025g01250.1	target 5' TCTTCATCCTTCACTTGCACCT 3'<br>::: :: ::::::.:::::::<br>miRNA 3' AGACGT-GGAAGTGGACGTGGA 5'	3	69-90	Nucleoporin seh1-like
vvi-miRC530-3p	VIT_202s0025g01250.2	target 5' TCTTCATCCTTCACTTGCACCT 3'<br>::: :: ::::::.:::::::<br>miRNA 3' AGACGT-GGAAGTGGACGTGGA 5'	3	127-148	Nucleoporin seh1-like
vvi-miRC530-3p	VIT_213s0067g01550.1	target 5' TCTTCACCTTCACCTTCACCT 3'<br>::: ::::::::::: :::::<br>miRNA 3' AGACGTGGAAGTGGACGTGGA 5'	3	670-690	Probable lrr receptor-like serine threonine-protein kinase mrh1-like
vvi-miRC530-5p	VIT_202s0012g02510.1	target 5' AGGT-CAGGTGCAAATGCAGG 3'<br>:::: :::::::::::::::.<br>miRNA 3' TCCACGTCCACGTTTACGTCT 5'	1.5	268-287	Protein
vvi-miRC530-5p	VIT_205s0020g04860.1	target 5' AGGTGCAGGTGCAAATGCAGG 3'<br>::::::::::::::::::::.<br>miRNA 3' TCCACGTCCACGTTTACGTCT 5'	0.5	99-119	Zinc knuckle (cchc-type) family protein
vvi-miRC530-5p	VIT_205s0020g04860.2	target 5' AGGTGCAGGTGCAAATGCAGG 3'<br>::::::::::::::::::::.<br>miRNA 3' TCCACGTCCACGTTTACGTCT 5'	0.5	258-278	Zinc knuckle (cchc-type) family protein
vvi-miRC530-5p	VIT_205s0020g04860.3	target 5' AGGTGCAGGTGCAAATGCAGG 3'<br>::::::::::::::::::::.<br>miRNA 3' TCCACGTCCACGTTTACGTCT 5'	0.5	168-188	Zinc knuckle (cchc-type) family protein
vvi-miRC530-5p	VIT_205s0020g04860.4	target 5' AGGTGCAGGTGCAAATGCAGG 3'<br>::::::::::::::::::::.<br>miRNA 3' TCCACGTCCACGTTTACGTCT 5'	0.5	258-278	Zinc knuckle (cchc-type) family protein
vvi-miRC530-5p	VIT_205s0020g04860.6	target 5' AGGTGCAGGTGCAAATGCAGG 3'<br>::::::::::::::::::::.<br>miRNA 3' TCCACGTCCACGTTTACGTCT 5'	0.5	258-278	Zinc knuckle (cchc-type) family protein

miRNA	Target id	Alignment	Score	Range	Target gene functional annotation
vvi-miRC530-5p	VIT_205s0020g04860.7	target 5' AGGTGCAGGTGCAAATGCAGG 3'<br>::::::::::::::::::::.<br>miRNA 3' TCCACGTCCACGTTTACGTCT 5'	0.5	258-278	Zinc knuckle (cchc-type) family protein
vvi-miRC530-5p	VIT_206s0080g00400.1	target 5' AGGTGCAGGTGCAGATGCAGG 3'<br>:::::::::::::.::::::.<br>miRNA 3' TCCACGTCCACGTTTACGTCT 5'	1.5	143-163	Ribosomal protein l1
vvi-miRC530a	VIT_202s0012g02510.1	target 5' AGGT-CAGGTGCAAATGCAGG 3'<br>:::: :::::::::::::::.<br>miRNA 3' TCCACGTCCACGTTTACGTCT 5'	1.5	268-287	Protein
vvi-miRC530a	VIT_205s0020g04860.1	target 5' AGGTGCAGGTGCAAATGCAGG 3'<br>::::::::::::::::::::.<br>miRNA 3' TCCACGTCCACGTTTACGTCT 5'	0.5	99-119	Zinc knuckle (cchc-type) family protein
vvi-miRC530a	VIT_205s0020g04860.2	target 5' AGGTGCAGGTGCAAATGCAGG 3'<br>::::::::::::::::::::.<br>miRNA 3' TCCACGTCCACGTTTACGTCT 5'	0.5	258-278	Zinc knuckle (cchc-type) family protein
vvi-miRC530a	VIT_205s0020g04860.3	target 5' AGGTGCAGGTGCAAATGCAGG 3'<br>::::::::::::::::::::.<br>miRNA 3' TCCACGTCCACGTTTACGTCT 5'	0.5	168-188	Zinc knuckle (cchc-type) family protein
vvi-miRC530a	VIT_205s0020g04860.4	target 5' AGGTGCAGGTGCAAATGCAGG 3'<br>::::::::::::::::::::.<br>miRNA 3' TCCACGTCCACGTTTACGTCT 5'	0.5	258-278	Zinc knuckle (cchc-type) family protein
vvi-miRC530a	VIT_205s0020g04860.6	target 5' AGGTGCAGGTGCAAATGCAGG 3'<br>::::::::::::::::::::.<br>miRNA 3' TCCACGTCCACGTTTACGTCT 5'	0.5	258-278	Zinc knuckle (cchc-type) family protein
vvi-miRC530a	VIT_205s0020g04860.7	target 5' AGGTGCAGGTGCAAATGCAGG 3'<br>::::::::::::::::::::.<br>miRNA 3' TCCACGTCCACGTTTACGTCT 5'	0.5	258-278	Zinc knuckle (cchc-type) family protein
vvi-miRC530a	VIT_206s0080g00400.1	target 5' AGGTGCAGGTGCAGATGCAGG 3'<br>:::::::::::::.::::::.<br>miRNA 3' TCCACGTCCACGTTTACGTCT 5'	1.5	143-163	Ribosomal protein l1
vvi-miRC535f-3p	VIT_200s0358g00010.1	target 5' CTTGACAACGAGAGAGAGCAC 3'<br>::::: :::::::::::::<br>miRNA 3' ATACTGTCGCTCTCTCTCGTG 5'	3	72-92	Unnamed protein product [Vitis vinifera]
vvi-miRC535f-3p	VIT_207s0104g00030.1	target 5' CTTGACAACGAGAGAGAGCAC 3'<br>::::: :::::::::::::<br>miRNA 3' ATACTGTCGCTCTCTCTCGTG 5'	3	72-92	Unnamed protein product [Vitis vinifera]
vvi-miRC535f-5p	VIT_201s0011g00130.1	target 5' ATTGTGCTCTCTCTCTTCTGTCA 3'<br>: ::::::::::::::: :::::<br>miRNA 3' TCACACGAGAGAGAGAA-ACAGT 5'	3	1247-1269	Squamosa promoter-binding-like protein 6-like
vvi-miRC535f-5p	VIT_201s0011g00130.2	target 5' ATTGTGCTCTCTCTCTTCTGTCA 3'<br>: ::::::::::::::: :::::<br>miRNA 3' TCACACGAGAGAGAGAA-ACAGT 5'	3	1247-1269	Squamosa promoter-binding-like protein 6-like

miRNA	Target id	Alignment	Score	Range	Target gene functional annotation
vvi-miRC535f-5p	VIT_217s0000g01260.1	target 5' ATTGTGCTCTCTCTCTTCTGTCA 3'<br>: ::::::::::::::: :::::<br>miRNA 3' TCACACGAGAGAGAGAA-ACAGT 5'	3	950-972	Squamosa promoter-binding-like protein 13
vvi-miRC535g-3p	VIT_200s0358g00010.1	target 5' CTTGACAACGAGAGAGAGCAC 3'<br>::::: ::::::::::::<br>miRNA 3' ATACTGTCGCTCTCTCTCGTG 5'	3	72-92	Unnamed protein product [Vitis vinifera]
vvi-miRC535g-3p	VIT_207s0104g00030.1	target 5' CTTGACAACGAGAGAGAGCAC 3'<br>::::: ::::::::::::<br>miRNA 3' ATACTGTCGCTCTCTCTCGTG 5'	3	72-92	Unnamed protein product [Vitis vinifera]
vvi-miRC535g-5p	VIT_201s0011g00130.1	target 5' ATTGTGCTCTCTCTCTTCTGTCA 3'<br>: ::::::::::::::: :::::<br>miRNA 3' TCACACGAGAGAGAGAA-ACAGT 5'	3	1247-1269	Squamosa promoter-binding-like protein 6-like
vvi-miRC535g-5p	VIT_201s0011g00130.2	target 5' ATTGTGCTCTCTCTCTTCTGTCA 3'<br>: ::::::::::::::: :::::<br>miRNA 3' TCACACGAGAGAGAGAA-ACAGT 5'	3	1247-1269	Squamosa promoter-binding-like protein 6-like
vvi-miRC535g-5p	VIT_217s0000g01260.1	target 5' ATTGTGCTCTCTCTCTTCTGTCA 3'<br>: ::::::::::::::: :::::<br>miRNA 3' TCACACGAGAGAGAGAA-ACAGT 5'	3	950-972	Squamosa promoter-binding-like protein 13
vvi-miRC827-3p	VIT_204s0008g05050.1	target 5' TGTTT-TTGTTGATCATCTAA 3'<br>::::: ::: ::::::::::<br>miRNA 3' ACAAACAACTACTAGTAGATT 5'	3	393-412	Pentatricopeptide repeat-containing protein
vvi-miRC827-5p	VIT_201s0026g01490.1	target 5' CAC-AGAAGACCAGCAACAAAA 3'<br>:: ::: ::::::::::::::<br>miRNA 3' CTGATCTACTGGTCGTTGTTTT 5'	3	465-485	Nitrate transporter
vvi-miRC827-5p	VIT_201s0026g01490.2	target 5' CAC-AGAAGACCAGCAACAAAA 3'<br>:: ::: ::::::::::::::<br>miRNA 3' CTGATCTACTGGTCGTTGTTTT 5'	3	54-74	Nitrate transporter

[a] Score refers to values given by Target Finder using a plant-based scoring metric.
[b] Complementary site of the microRNA with the target gene.
