# Supplementary figures and images for: miRVine: a microRNA expression atlas of grapevine based on small RNA sequencing
Source: BMC Genomics. 2015 May 16;16(1):393. doi: 10.1186/s12864-015-1610-5 (PMC4434875; doi:10.1186/s12864-015-1610-5)

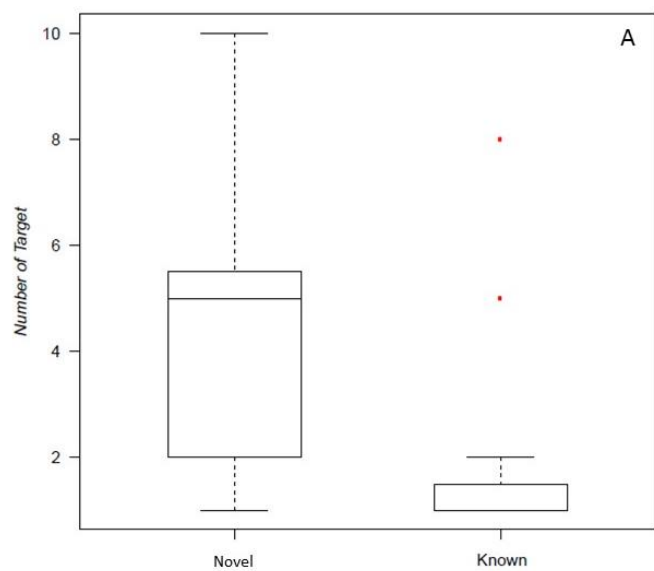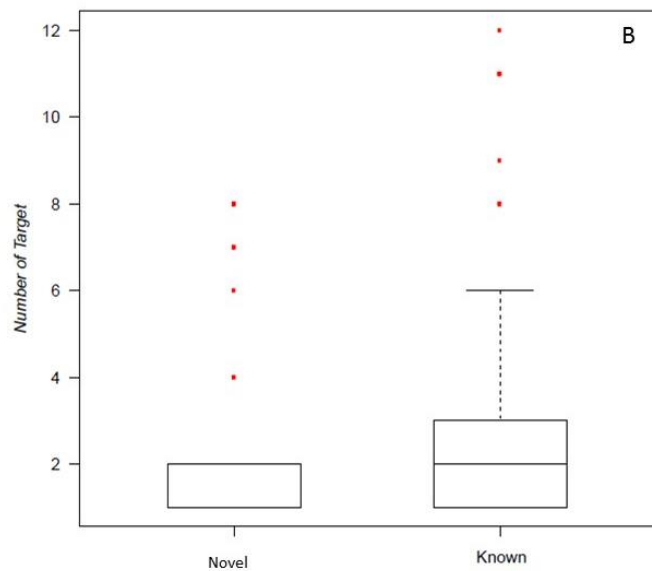

Supplement: Additional file 9: — Number of putative miRNA targets. Number of putative miRNA target within the (A) PN40024 and (B) Corvina data sets, identified using the TargetFinder (release 1.6). Novel and known refer to the different classes of miRNAs. [file 12864_2015_1610_MOESM9_ESM.pdf]

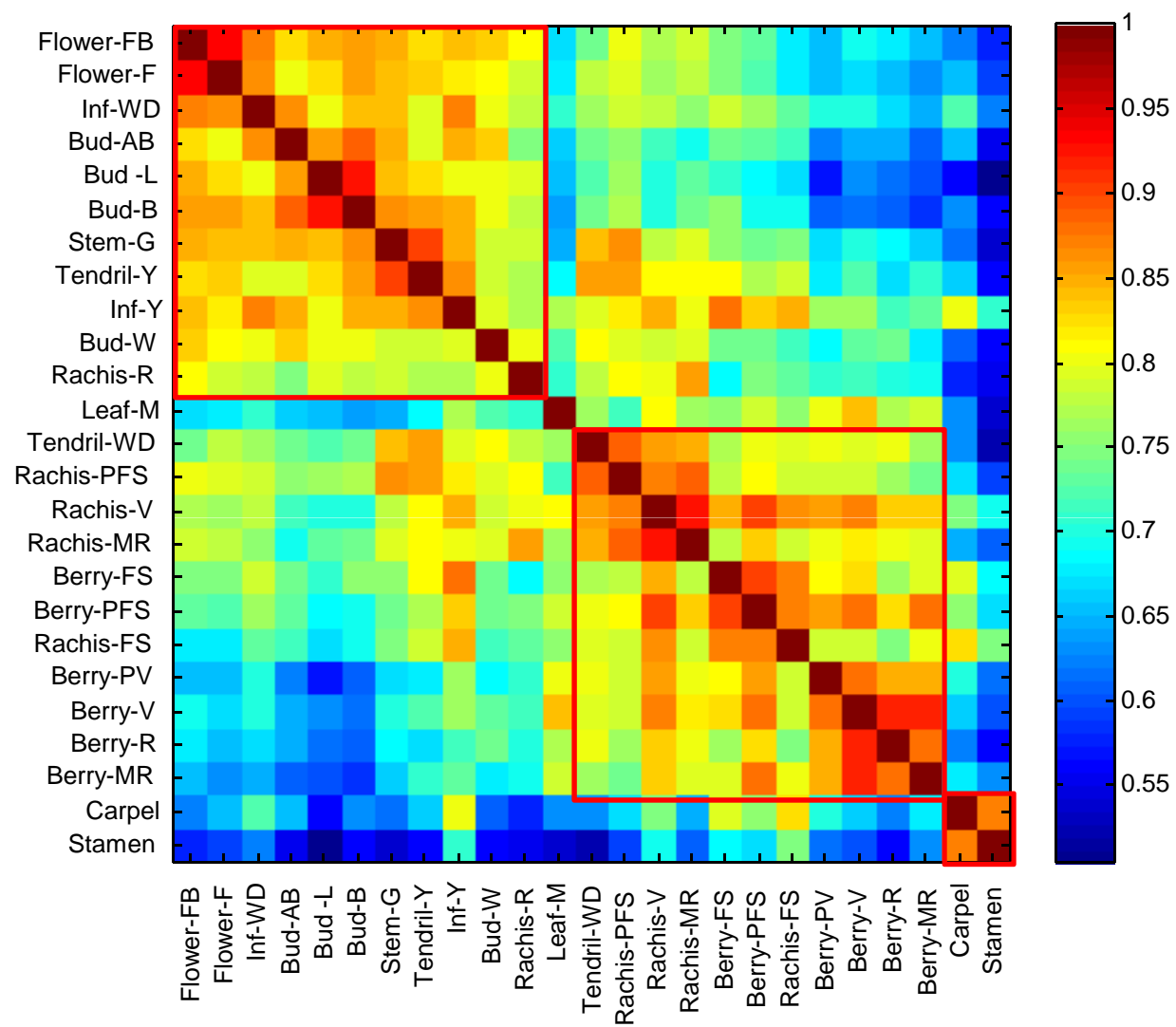

Supplement: Additional file 12: — Correlation matrix of the 25 grapevine samples used to define the miRNA atlas. One minus Pearson correlation was used as a metric distance. Main clusters identified are highlighted with a red box. [file 12864_2015_1610_MOESM12_ESM.pdf]
