# Supplementary material for: miRVine: a microRNA expression atlas of grapevine based on small RNA sequencing
Source: BMC Genomics. 2015 May 16;16(1):393. doi: 10.1186/s12864-015-1610-5 (PMC4434875; doi:10.1186/s12864-015-1610-5)
Supplement: Additional file 10: — miRNA - target correlations. From a list of 933 predicted miRNA - target pairs we could find 815 pairs for which the target gene expression values are available from a public dataset [4]. The table reports the Pearson correlations of the pairs for which the P value is less than 0.05. The P value is computed for testing the hypothesis of no correlation against the alternative that there is a non-zero correlation. The annotations reported are the same as in Additional file 8. [file 12864_2015_1610_MOESM10_ESM.pdf]

miRNA	Target id	Correlation	P value	Annotation
grape-m5176	VIT_218s0001g11250	-0.46	2.50E-02	NBS-LRR resistance protein
grape-m6905	VIT_213s0064g00290	-0.44	3.11E-02	bifunctional dihydroflavonol 4-reductase flavanone 4-reductase-like
vvi-miR156b-5p	VIT_201s0011g00130	-0.52	8.90E-03	squamosa promoter-binding-like protein 6-like
vvi-miR156b-5p	VIT_208s0007g06270	-0.47	1.92E-02	squamosa promoter-binding-like protein 9-like
vvi-miR156b-5p	VIT_211s0065g00170	-0.45	2.71E-02	squamosa promoter-binding-like protein 12-like
vvi-miR156b-5p	VIT_215s0021g02290	-0.44	3.14E-02	squamosa promoter-binding-like protein 7-like
vvi-miR156b-5p	VIT_214s0068g01780	-0.42	4.03E-02	squamosa promoter-binding-like protein 16-like
vvi-miR156c-5p	VIT_201s0011g00130	-0.52	8.90E-03	squamosa promoter-binding-like protein 6-like
vvi-miR156c-5p	VIT_208s0007g06270	-0.47	1.92E-02	squamosa promoter-binding-like protein 9-like
vvi-miR156c-5p	VIT_211s0065g00170	-0.45	2.71E-02	squamosa promoter-binding-like protein 12-like
vvi-miR156c-5p	VIT_215s0021g02290	-0.44	3.14E-02	squamosa promoter-binding-like protein 7-like
vvi-miR156c-5p	VIT_214s0068g01780	-0.42	4.03E-02	squamosa promoter-binding-like protein 16-like
vvi-miR156d-5p	VIT_201s0011g00130	-0.52	8.90E-03	squamosa promoter-binding-like protein 6-like
vvi-miR156d-5p	VIT_208s0007g06270	-0.47	1.92E-02	squamosa promoter-binding-like protein 9-like
vvi-miR156d-5p	VIT_211s0065g00170	-0.45	2.71E-02	squamosa promoter-binding-like protein 12-like
vvi-miR156d-5p	VIT_215s0021g02290	-0.44	3.14E-02	squamosa promoter-binding-like protein 7-like
vvi-miR156d-5p	VIT_214s0068g01780	-0.42	4.03E-02	squamosa promoter-binding-like protein 16-like
vvi-miR156f	VIT_211s0065g00170	-0.53	7.18E-03	squamosa promoter-binding-like protein 12-like
vvi-miR156f	VIT_217s0000g05020	-0.44	3.07E-02	squamosa promoter-binding-like protein 6-like
vvi-miR156g-5p	VIT_211s0065g00170	-0.53	7.18E-03	squamosa promoter-binding-like protein 12-like
vvi-miR156g-5p	VIT_217s0000g05020	-0.44	3.07E-02	squamosa promoter-binding-like protein 6-like
vvi-miR156i	VIT_211s0065g00170	-0.53	7.18E-03	squamosa promoter-binding-like protein 12-like
vvi-miR156i	VIT_217s0000g05020	-0.44	3.07E-02	squamosa promoter-binding-like protein 6-like
vvi-miR166b-3p	VIT_204s0008g03250	-0.51	1.09E-02	class III HD-Zip protein 8
vvi-miR166d-5p	VIT_208s0056g01350	-0.46	2.54E-02	uncharacterized protein
vvi-miR3635-3p	VIT_217s0000g05850	-0.52	8.69E-03	ABC transporter retinal flippase subfamily
vvi-miR394c	VIT_201s0010g03730	-0.60	1.85E-03	F-box family protein
vvi-miR394c	VIT_201s0010g03730	-0.60	1.85E-03	F-box family protein
vvi-miR396a-5p	VIT_202s0025g02680	-0.42	4.36E-02	uncharacterized protein loc100258227
vvi-miR396c-5p	VIT_202s0025g02680	-0.48	1.85E-02	uncharacterized protein loc100258227
vvi-miR396d-5p	VIT_202s0025g02680	-0.48	1.85E-02	uncharacterized protein loc100258227
vvi-miR397a-5p	VIT_208s0040g01790	-0.43	3.83E-02	laccase (diphenol oxidase)-like protein

vvi-miR397a-5p	VIT_208s0040g01790	-0.43	3.83E-02	laccase (diphenol oxidase)-like protein
vvi-miR408-3p	VIT_212s0034g01140	-0.48	1.86E-02	basic blue protein
vvi-miR408-3p	VIT_218s0001g15240	-0.42	4.17E-02	basic blue protein
vvi-miRC171h-3p	VIT_202s0154g00400	-0.48	1.69E-02	gras family transcription factor
vvi-miRC171j	VIT_202s0154g00400	-0.48	1.69E-02	gras family transcription factor
vvi-miRC477c-3p	VIT_217s0000g02070	-0.44	3.19E-02	deoxyhypusine synthase
vvi-miRC477d-3p	VIT_217s0000g02070	-0.44	3.19E-02	deoxyhypusine synthase
vvi-miRC477e-3p	VIT_217s0000g02070	-0.44	3.19E-02	deoxyhypusine synthase
vvi-miRC477g-3p	VIT_217s0000g02070	-0.44	3.19E-02	deoxyhypusine synthase
vvi-miRC477h-3p	VIT_217s0000g02070	-0.44	3.19E-02	deoxyhypusine synthase
