# Supplementary material for: miRVine: a microRNA expression atlas of grapevine based on small RNA sequencing
Source: BMC Genomics. 2015 May 16;16(1):393. doi: 10.1186/s12864-015-1610-5 (PMC4434875; doi:10.1186/s12864-015-1610-5)
Supplement: Additional file 11: — Top 20 expressed miRNAs per tissue/developmental stage. Top 20 expressed miRNAs in each tissue/developmental stage studied. Reads refers to the average of TP5M of both replicates. [file 12864_2015_1610_MOESM11_ESM.pdf]

**Leaf_M**			**Bud_AB**			**Bud_W**			**Bud_L**		
S.No	miRNAs	Reads	S.No	miRNAs	Reads	S.No	miRNAs	Reads	S.No	miRNAs	Reads
1	vvi-miR166c-3p	1411846	1	vvi-miR166c-3p	1346029	1	vvi-miR166c-3p	555991	1	vvi-miR166c-3p	92987
2	vvi-miR166d-3p	1411846	2	vvi-miR166d-3p	1346029	2	vvi-miR166d-3p	555991	2	vvi-miR166d-3p	92987
3	vvi-miR166e-3p	1411846	3	vvi-miR166e-3p	1346029	3	vvi-miR166e-3p	555991	3	vvi-miR166e-3p	92987
4	vvi-miR166f-3p	1411846	4	vvi-miR166f-3p	1346029	4	vvi-miR166f-3p	555991	4	vvi-miR166f-3p	92987
5	vvi-miR166g-3p	1411846	5	vvi-miR166g-3p	1346029	5	vvi-miR166g-3p	555991	5	vvi-miR166g-3p	92987
6	vvi-miR166h-3p	1411846	6	vvi-miR166h-3p	1346029	6	vvi-miR166h-3p	555991	6	vvi-miR166h-3p	92987
7	vvi-miRC166i-3p	1411846	7	vvi-miRC166i-3p	1346029	7	vvi-miRC166i-3p	555991	7	vvi-miRC166i-3p	92987
8	vvi-miR3634-3p	873783	8	vvi-miR3634-3p	151813	8	vvi-miR166a-3p	364999	8	vvi-miR166a-3p	48087.5
9	vvi-miR166a-3p	269926	9	vvi-miR166a-3p	145257	9	vvi-miR3634-3p	153711	9	vvi-miR3634-3p	15087
10	vvi-miR396c-5p	20442.5	10	vvi-miR159c.1-3p	27701.5	10	vvi-miR159c.1-3p	80921	10	vvi-miR159c.2-3p	12475.5
11	vvi-miR396d-5p	20442.5	11	grape-m3245-5p	22767	11	vvi-miR162-3p	18238	11	vvi-miR482-5p	7835
12	vvi-miR162-3p	7812	12	vvi-miR159c.2-3p	10538	12	grape-m3245-3p	17219	12	grape-m3245-3p	6280
13	vvi-miR396b-5p	5868.5	13	vvi-miR166b-3p	7501	13	vvi-miR3623-5p	16886	13	grape-m3245-5p	6120
14	vvi-miR159c.1-3p	5538	14	vvi-miR319b-3p	6495	14	vvi-miR403a-3p	12730.5	14	vvi-miR162-3p	5489
15	vvi-miR403a-3p	2389	15	vvi-miR319c-3p	6495	15	vvi-miR403b	12730.5	15	vvi-miR166c-5p	4634
16	vvi-miR403b	2389	16	vvi-miR319f-3p	6495	16	vvi-miR403d	12730.5	16	vvi-miR166e-5p	4634
17	vvi-miR403d	2389	17	vvi-miR319g	6495	17	vvi-miR403e-3p	12730.5	17	vvi-miRC166i-5p	4634
18	vvi-miR403e-3p	2389	18	vvi-miR159c.2-5p	4646.5	18	vvi-miR403f-3p	12730.5	18	vvi-miR482-3p	4012.5
19	vvi-miR403f-3p	2389	19	vvi-miR482-5p	4146.5	19	vvi-miR479-3p	11886	19	vvi-miR403a-3p	3584.5
20	vvi-miR3624-3p	2351	20	vvi-miR482-3p	3137	20	vvi-miR319b-3p	10155	20	vvi-miR403b	3584.5

Bud_B			Rachis-FS			Rachis-PFS			Rachis-V		
S.No	miRNAs	Reads	S.No	miRNAs	Reads	S.No	miRNAs	Reads	S.No	miRNAs	Reads
1	vvi-miR3634-3p	166556	1	vvi-miR166c-3p	203009	1	vvi-miR166c-3p	138097	1	vvi-miR166c-3p	376710
2	vvi-miR166c-3p	147487	2	vvi-miR166d-3p	203009	2	vvi-miR166d-3p	138097	2	vvi-miR166d-3p	376710
3	vvi-miR166d-3p	147487	3	vvi-miR166e-3p	203009	3	vvi-miR166e-3p	138097	3	vvi-miR166e-3p	376710
4	vvi-miR166e-3p	147487	4	vvi-miR166f-3p	203009	4	vvi-miR166f-3p	138097	4	vvi-miR166f-3p	376710
5	vvi-miR166f-3p	147487	5	vvi-miR166g-3p	203009	5	vvi-miR166g-3p	138097	5	vvi-miR166g-3p	376710
6	vvi-miR166g-3p	147487	6	vvi-miR166h-3p	203009	6	vvi-miR166h-3p	138097	6	vvi-miR166h-3p	376710
7	vvi-miR166h-3p	147487	7	vvi-miRC166i-3p	203009	7	vvi-miRC166i-3p	138097	7	vvi-miRC166i-3p	376710
8	vvi-miRC166i-3p	147487	8	vvi-miR159c.1-3p	92341.5	8	vvi-miR166a-3p	75475.5	8	vvi-miR166a-3p	85632
9	vvi-miR166a-3p	132012	9	vvi-miR166a-3p	38556	9	vvi-miR3634-3p	54838	9	vvi-miR159c.1-3p	57627

10	vvi-miR159c.1-3p	19145.5	10	grape-m3245-5p	15702	10	vvi-miR482-5p	30074	10	vvi-miR3634-3p	53037
11	vvi-miR159c.2-3p	11873	11	vvi-miR319b-3p	15008.5	11	vvi-miR159c.1-3p	17441	11	grape-m3245-5p	10836.5
12	vvi-miR482-5p	8991	12	vvi-miR319c-3p	15008.5	12	vvi-miR319b-3p	13780	12	vvi-miR319b-3p	7697
13	grape-m3245-5p	8441.5	13	vvi-miR319f-3p	15008.5	13	vvi-miR319c-3p	13780	13	vvi-miR319c-3p	7697
14	grape-m3245-3p	7087	14	vvi-miR319g	15008.5	14	vvi-miR319f-3p	13780	14	vvi-miR319f-3p	7697
15	vvi-miR166b-5p	6810.5	15	vvi-miR3634-3p	8488.5	15	vvi-miR319g	13780	15	vvi-miR319g	7697
16	vvi-miR166f-5p	6810.5	16	vvi-miR3633a-5p	2451.5	16	grape-m3245-5p	9973.5	16	vvi-miR482-5p	6126
17	vvi-miR166c-5p	4577	17	vvi-miR482-5p	2064.5	17	vvi-miR166c-5p	8773.5	17	grape-m3245-3p	3692.5
18	vvi-miR166e-5p	4577	18	vvi-miRC396e-5p	1817.5	18	vvi-miR166e-5p	8773.5	18	vvi-miR162-3p	3369
19	vvi-miRC166i-5p	4577	19	grape-m2399	1682	19	vvi-miRC166i-5p	8773.5	19	vvi-miR319e	3184.5
20	vvi-miR403a-3p	4511.5	20	grape-m7218-5p	1430.5	20	vvi-miR3633a-5p	8456	20	vvi-miR166c-5p	2504

Rachis-MR			Rachis-R			Stem-G			Tendril-Y		
S.No	miRNAs	Reads	S.No	miRNAs	Reads	S.No	miRNAs	Reads	S.No	miRNAs	Reads
1	vvi-miR166c-3p	76117	1	vvi-miR166a-3p	41422.5	1	vvi-miR3634-3p	336382	1	vvi-miR166a-3p	374005
2	vvi-miR166d-3p	76117	2	vvi-miR3634-3p	40550	2	vvi-miR166a-3p	259763	2	vvi-miR3634-3p	255677
3	vvi-miR166e-3p	76117	3	vvi-miR166c-3p	32917.5	3	vvi-miR166c-3p	154311	3	vvi-miR166c-3p	110023
4	vvi-miR166f-3p	76117	4	vvi-miR166d-3p	32917.5	4	vvi-miR166d-3p	154311	4	vvi-miR166d-3p	110023
5	vvi-miR166g-3p	76117	5	vvi-miR166e-3p	32917.5	5	vvi-miR166e-3p	154311	5	vvi-miR166e-3p	110023
6	vvi-miR166h-3p	76117	6	vvi-miR166f-3p	32917.5	6	vvi-miR166f-3p	154311	6	vvi-miR166f-3p	110023
7	vvi-miRC166i-3p	76117	7	vvi-miR166g-3p	32917.5	7	vvi-miR166g-3p	154311	7	vvi-miR166g-3p	110023
8	vvi-miR3634-3p	49577	8	vvi-miR166h-3p	32917.5	8	vvi-miR166h-3p	154311	8	vvi-miR166h-3p	110023
9	vvi-miR166a-3p	43691	9	vvi-miRC166i-3p	32917.5	9	vvi-miRC166i-3p	154311	9	vvi-miRC166i-3p	110023
10	vvi-miR482-5p	14858.5	10	vvi-miR162-3p	10113	10	vvi-miR159c.1-3p	30370.5	10	vvi-miR159c.1-3p	24273.5
11	vvi-miR159c.1-3p	10484.5	11	vvi-miR482-5p	9525.5	11	vvi-miR319b-3p	26134	11	vvi-miR482-5p	21938
12	grape-m3245-5p	7221	12	grape-m3245-5p	8008.5	12	vvi-miR319c-3p	26134	12	vvi-miR162-3p	13413
13	vvi-miR162-3p	5766.5	13	grape-m3245-3p	4968.5	13	vvi-miR319f-3p	26134	13	grape-m3245-3p	12087
14	grape-m3245-3p	5631	14	vvi-miR159c.1-3p	3732	14	vvi-miR319g	26134	14	vvi-miR166c-5p	8793.5
15	vvi-miR166c-5p	3889.5	15	vvi-miR3633a-5p	3336	15	vvi-miR482-5p	9238	15	vvi-miR166e-5p	8793.5
16	vvi-miR166e-5p	3889.5	16	vvi-miR3623-3p	2922.5	16	vvi-miR166c-5p	8830	16	vvi-miRC166i-5p	8793.5
17	vvi-miRC166i-5p	3889.5	17	vvi-miR403a-3p	2297	17	vvi-miR166e-5p	8830	17	grape-m3245-5p	7886
18	vvi-miR3633a-5p	2678.5	18	vvi-miR403b	2297	18	vvi-miRC166i-5p	8830	18	vvi-miR319b-3p	5806.5
19	vvi-miR403a-3p	2184	19	vvi-miR403d	2297	19	grape-m3245-5p	6694.5	19	vvi-miR319c-3p	5806.5
20	vvi-miR403b	2184	20	vvi-miR403e-3p	2297	20	vvi-miR396c-5p	5793	20	vvi-miR319f-3p	5806.5

Tendril-WD			Carpel			Stamen			Inf-Y		
S.No	miRNAs	Reads	S.No	miRNAs	Reads	S.No	miRNAs	Reads	S.No	miRNAs	Reads
1	vvi-miR166a-3p	468851	1	vvi-miR159c.1-3p	690175	1	vvi-miR159c.1-3p	310221	1	vvi-miR166c-3p	708838
2	vvi-miR166c-3p	234990	2	vvi-miR319b-3p	59597.5	2	vvi-miR319b-3p	79153	2	vvi-miR166d-3p	708838
3	vvi-miR166d-3p	234990	3	vvi-miR319c-3p	59597.5	3	vvi-miR319c-3p	79153	3	vvi-miR166e-3p	708838
4	vvi-miR166e-3p	234990	4	vvi-miR319f-3p	59597.5	4	vvi-miR319f-3p	79153	4	vvi-miR166f-3p	708838
5	vvi-miR166f-3p	234990	5	vvi-miR319g	59597.5	5	vvi-miR319g	79153	5	vvi-miR166g-3p	708838
6	vvi-miR166g-3p	234990	6	grape-m3245-5p	46488	6	vvi-miR166c-3p	25691.5	6	vvi-miR166h-3p	708838
7	vvi-miR166h-3p	234990	7	vvi-miR166c-3p	31766.5	7	vvi-miR166d-3p	25691.5	7	vvi-miRC166i-3p	708838
8	vvi-miRC166i-3p	234990	8	vvi-miR166d-3p	31766.5	8	vvi-miR166e-3p	25691.5	8	vvi-miR3634-3p	305165
9	vvi-miR3634-3p	208780	9	vvi-miR166e-3p	31766.5	9	vvi-miR166f-3p	25691.5	9	vvi-miR166a-3p	201462
10	vvi-miR159c.1-3p	60522	10	vvi-miR166f-3p	31766.5	10	vvi-miR166g-3p	25691.5	10	vvi-miR159c.1-3p	40004.5
11	vvi-miR482-5p	13486.5	11	vvi-miR166g-3p	31766.5	11	vvi-miR166h-3p	25691.5	11	vvi-miR166c-5p	17148.5
12	grape-m3245-3p	10735	12	vvi-miR166h-3p	31766.5	12	vvi-miRC166i-3p	25691.5	12	vvi-miR166e-5p	17148.5
13	vvi-miR162-3p	9730.5	13	vvi-miRC166i-3p	31766.5	13	grape-m3245-5p	17045	13	vvi-miRC166i-5p	17148.5
14	vvi-miR396c-5p	9553	14	vvi-miR319e	9382.5	14	vvi-miR398b-3p	9609	14	grape-m3245-5p	10227.5
15	vvi-miR396d-5p	9553	15	vvi-miRC477f-3p	6237.5	15	vvi-miR398c-3p	9609	15	vvi-miR319b-3p	8539.5
16	vvi-miR403a-3p	7712	16	vvi-miR166b-3p	3367.5	16	vvi-miR482-5p	6454.5	16	vvi-miR319c-3p	8539.5
17	vvi-miR403b	7712	17	vvi-miR482-5p	3362	17	vvi-miR156f	5625.5	17	vvi-miR319f-3p	8539.5
18	vvi-miR403d	7712	18	vvi-miR398b-3p	3157	18	vvi-miR156g-5p	5625.5	18	vvi-miR319g	8539.5
19	vvi-miR403e-3p	7712	19	vvi-miR398c-3p	3157	19	vvi-miR156i	5625.5	19	vvi-miR166b-5p	8489
20	vvi-miR403f-3p	7712	20	vvi-miR156f	2639.5	20	vvi-miR319e	5018.5	20	vvi-miR166f-5p	8489

Inf-WD			Flower-FB			Flower-F			Berry-FS		
S.No	miRNAs	Reads	S.No	miRNAs	Reads	S.No	miRNAs	Reads	S.No	miRNAs	Reads
1	vvi-miR166c-3p	1305947	1	vvi-miR166c-3p	513460	1	vvi-miR3634-3p	140452	1	vvi-miR166c-3p	908707
2	vvi-miR166d-3p	1305947	2	vvi-miR166d-3p	513460	2	vvi-miR166c-3p	122023	2	vvi-miR166d-3p	908707
3	vvi-miR166e-3p	1305947	3	vvi-miR166e-3p	513460	3	vvi-miR166d-3p	122023	3	vvi-miR166e-3p	908707
4	vvi-miR166f-3p	1305947	4	vvi-miR166f-3p	513460	4	vvi-miR166e-3p	122023	4	vvi-miR166f-3p	908707
5	vvi-miR166g-3p	1305947	5	vvi-miR166g-3p	513460	5	vvi-miR166f-3p	122023	5	vvi-miR166g-3p	908707
6	vvi-miR166h-3p	1305947	6	vvi-miR166h-3p	513460	6	vvi-miR166g-3p	122023	6	vvi-miR166h-3p	908707
7	vvi-miRC166i-3p	1305947	7	vvi-miRC166i-3p	513460	7	vvi-miR166h-3p	122023	7	vvi-miRC166i-3p	908707
8	vvi-miR3634-3p	284537	8	vvi-miR3634-3p	171663	8	vvi-miRC166i-3p	122023	8	vvi-miR166a-3p	129452
9	vvi-miR166a-3p	130906	9	vvi-miR166a-3p	93820.5	9	vvi-miR166a-3p	98738	9	vvi-miR3634-3p	70207.5

10	vvi-miR159c.1-3p	16951.5	10	vvi-miR162-3p	43447.5	10	vvi-miR162-3p	42048	10	vvi-miR159c.1-3p	19575
11	vvi-miR162-3p	8046	11	grape-m3245-3p	15089.5	11	grape-m3245-3p	26505	11	grape-m3245-5p	15664
12	grape-m3245-3p	6565	12	vvi-miR159c.1-3p	9989.5	12	vvi-miR482-5p	16877.5	12	vvi-miR166b-3p	8734.5
13	vvi-miR319b-3p	5973.5	13	vvi-miR403a-3p	8983	13	vvi-miR159c.1-3p	11100	13	vvi-miR166c-5p	6054
14	vvi-miR319c-3p	5973.5	14	vvi-miR403b	8983	14	vvi-miR168-5p	6218	14	vvi-miR166e-5p	6054
15	vvi-miR319f-3p	5973.5	15	vvi-miR403d	8983	15	vvi-miR403a-3p	6110	15	vvi-miRC166i-5p	6054
16	vvi-miR319g	5973.5	16	vvi-miR403e-3p	8983	16	vvi-miR403b	6110	16	vvi-miR482-5p	3635.5
17	vvi-miR166c-5p	5198.5	17	vvi-miR403f-3p	8983	17	vvi-miR403d	6110	17	vvi-miRC396e-5p	3384.5
18	vvi-miR166e-5p	5198.5	18	vvi-miR168-3p	8016.5	18	vvi-miR403e-3p	6110	18	grape-m3245-3p	2101.5
19	vvi-miRC166i-5p	5198.5	19	vvi-miR166c-5p	6980.5	19	vvi-miR403f-3p	6110	19	vvi-miR162-3p	1969
20	vvi-miR482-5p	5182	20	vvi-miR166e-5p	6980.5	20	vvi-miR166c-5p	5834.5	20	vvi-miR166b-5p	1772.5

Berry-PFS			Berry-PV			Berry-V			Berry-MR		
S.No	miRNAs	Reads	S.No	miRNAs	Reads	S.No	miRNAs	Reads	S.No	miRNAs	Reads
1	vvi-miR166c-3p	347960	1	vvi-miR166c-3p	997373	1	vvi-miR166c-3p	713119	1	vvi-miR166c-3p	166018
2	vvi-miR166d-3p	347960	2	vvi-miR166d-3p	997373	2	vvi-miR166d-3p	713119	2	vvi-miR166d-3p	166018
3	vvi-miR166e-3p	347960	3	vvi-miR166e-3p	997373	3	vvi-miR166e-3p	713119	3	vvi-miR166e-3p	166018
4	vvi-miR166f-3p	347960	4	vvi-miR166f-3p	997373	4	vvi-miR166f-3p	713119	4	vvi-miR166f-3p	166018
5	vvi-miR166g-3p	347960	5	vvi-miR166g-3p	997373	5	vvi-miR166g-3p	713119	5	vvi-miR166g-3p	166018
6	vvi-miR166h-3p	347960	6	vvi-miR166h-3p	997373	6	vvi-miR166h-3p	713119	6	vvi-miR166h-3p	166018
7	vvi-miRC166i-3p	347960	7	vvi-miRC166i-3p	997373	7	vvi-miRC166i-3p	713119	7	vvi-miRC166i-3p	166018
8	vvi-miR166a-3p	50292.5	8	vvi-miR166a-3p	93482.5	8	vvi-miR166a-3p	73075	8	vvi-miR166a-3p	45213.5
9	vvi-miR3634-3p	8865.5	9	vvi-miR3634-3p	27356.5	9	vvi-miR3634-3p	37580.5	9	vvi-miR3634-3p	19130
10	grape-m3245-5p	5726.5	10	vvi-miR159c.1-3p	23248	10	vvi-miR162-3p	5996	10	vvi-miR159c.1-3p	6237.5
11	vvi-miR159c.1-3p	4774.5	11	vvi-miR396b-5p	10637.5	11	vvi-miR159c.1-3p	5115.5	11	vvi-miR162-3p	4608.5
12	vvi-miR166c-5p	3567.5	12	vvi-miR3623-5p	10522	12	vvi-miR396b-5p	4629	12	grape-m3245-5p	3436.5
13	vvi-miR166e-5p	3567.5	13	grape-m3245-5p	10349	13	vvi-miR403a-3p	3930.5	13	vvi-miR319e	2721
14	vvi-miRC166i-5p	3567.5	14	vvi-miR319e	7075.5	14	vvi-miR403b	3930.5	14	vvi-miR403a-3p	2197.5
15	vvi-miR396b-5p	1839	15	vvi-miR482-5p	5956.5	15	vvi-miR403d	3930.5	15	vvi-miR403b	2197.5
16	grape-m3245-3p	1780.5	16	vvi-miR166c-5p	5338.5	16	vvi-miR403e-3p	3930.5	16	vvi-miR403d	2197.5
17	vvi-miRC396e-5p	1722	17	vvi-miR166e-5p	5338.5	17	vvi-miR403f-3p	3930.5	17	vvi-miR403e-3p	2197.5
18	vvi-miR482-3p	1367.5	18	vvi-miRC166i-5p	5338.5	18	grape-m3245-5p	2309	18	vvi-miR403f-3p	2197.5
19	vvi-miR162-3p	1329.5	19	vvi-miRC396e-5p	5209	19	vvi-miR3623-5p	1687.5	19	vvi-miR482-5p	1708.5
20	vvi-miR319e	974	20	vvi-miR162-3p	4475	20	vvi-miRC477c-3p	1635	20	vvi-miRC396e-5p	1641

	Berry-R	
S.No	miRNAs	Reads
1	vvi-miR166c-3p	456797
2	vvi-miR166d-3p	456797
3	vvi-miR166e-3p	456797
4	vvi-miR166f-3p	456797
5	vvi-miR166g-3p	456797
6	vvi-miR166h-3p	456797
7	vvi-miRC166i-3p	456797
8	vvi-miR166a-3p	27529.5
9	vvi-miR3634-3p	22796
10	vvi-miR159c.1-3p	13861
11	vvi-miR162-3p	9241
12	vvi-miR403a-3p	8974.5
13	vvi-miR403b	8974.5
14	vvi-miR403d	8974.5
15	vvi-miR403e-3p	8974.5
16	vvi-miR403f-3p	8974.5
17	vvi-miRC477c-3p	4859.5
18	vvi-miRC477d-3p	4859.5
19	vvi-miRC477e-3p	4859.5
20	vvi-miRC477g-3p	4859.5
