# Supplementary material for: miRVine: a microRNA expression atlas of grapevine based on small RNA sequencing
Source: BMC Genomics. 2015 May 16;16(1):393. doi: 10.1186/s12864-015-1610-5 (PMC4434875; doi:10.1186/s12864-015-1610-5)
Supplement: Additional file 13: — Additional plant material details. [file 12864_2015_1610_MOESM13_ESM.pdf]

Plant material details

Buds were collected at five developmental stages: namely first season latent bud (E-L 23), winter dormant bud (E-L 1), bud, burst showing green tip (E-L 4) and final bud stage after burst, (E-L 5). Inflorescences were collected at two developmental stages: young inflorescence with single flower in compact groups (E-L 14), and well-developed inflorescence with single flower separated (E-L 17). Flowers were collected at the beginning of flowering (E-L 20) and flowering phase (E-L 23); flower organs were collected from undisclosed flowers. Pollen was collected from disclosed flowers at 80% caps off (E-L25).

Tendrils were collected from two developmental stages: the first when the shoot bears seven separate leaves (E-L 14) and the second corresponding to a pool of well-developed tendrils collected when the shoot bears 12 separate leaves (E-L 17). Leaves were collected at three developmental stages: young leaves when the shoot contains about five well-separated leaves (EL14), mature leaves when the berry size was about 4 mm in diameter (E-L 29), and leaves in senescence, collected before the beginning of leaf fall (E-L43).

Berries were collected at six developmental time points by freezing the whole berry and removing the seeds. The six stages are fruit set (FS), 15 days after flowering (DAF; E-L29), post fruit set (PFS), when berries reach >7 mm diam. (E-L 31), pre-veraison (E-L 34), veraison (V), when berries change color (10.4 °Brix,70 DAF; E-L35), mid-ripening (MR) stage (84 DAF; E-L 36,15.5 °Brix) and the last stage ripening (R) (115 DAF; E-L 38, 20 °Brix). For post-harvest samples, bunches were placed for three months in single layers in naturally ventilated rooms, without any artificial control of temperature and humidity. Samples were collected every month. Withering stage I was characterized by a weight reduction of 23.6% and 24.5 °Brix, the second stage by weight reduction of 30.3% and 25.9 °Brix and the last stage by a weight reduction of 32.7% and 26.7 °Brix.
