# Supplementary material for: miRVine: a microRNA expression atlas of grapevine based on small RNA sequencing
Source: BMC Genomics. 2015 May 16;16(1):393. doi: 10.1186/s12864-015-1610-5 (PMC4434875; doi:10.1186/s12864-015-1610-5)
Supplement: Additional file 14: — Pearson correlation between replicates. Values of Pearson correlation between replicates using log2 transformed data. [file 12864_2015_1610_MOESM14_ESM.pdf]

Sample	Pearson coeff.	P-value
Berry_FS	0.97	2.02E-218
Berry_MR	0.83	6.78E-90
Berry_PFS	0.88	1.34E-115
Berry_PV	0.96	6.73E-193
Berry_R	0.97	7.73E-217
Berry_V	0.96	8.07E-193
Bud_AB	0.96	1.03E-197
Bud_B	0.95	1.09E-173
Bud_L	0.95	1.43E-183
Bud_W	0.98	9.14E-246
Carpel	0.94	2.01E-162
Flower_F	0.96	2.60E-190
Flower_FB	0.97	6.81E-212
Inf_WD	0.99	1.14E-275
Inf_Y	0.96	2.86E-197
Leaf_FS	0.98	5.94E-239
Rachis_FS	0.92	5.88E-147
Rachis_MR	0.96	1.87E-190
Rachis_PFS	0.95	2.62E-177
Rachis_R	0.86	1.26E-105
Rachis_V	0.96	2.54E-194
Stamen	0.89	1.43E-118
Stem_G	0.97	1.41E-219
Tendril_WD	0.99	2.43E-290
Tendril_Y	0.95	2.67E-181
