# Supplementary material for: miRVine: a microRNA expression atlas of grapevine based on small RNA sequencing
Source: BMC Genomics. 2015 May 16;16(1):393. doi: 10.1186/s12864-015-1610-5 (PMC4434875; doi:10.1186/s12864-015-1610-5)
Supplement: Additional file 3: — Stem-loop structure of two miRNA loci generating more than one duplex. Dot-bracket notation representing RNA stem-loop structure of some examples of miRNA loci, generating more than one distinct duplex from the same precursor. For each precursor MIR locus and mapping position (chromosome, strand, coordinates) are presented. Below the dot-bracket notation, distinct miRNA duplexes are aligned and the sum of the abundance (TP5M) of the reads is shown in parenthesis. The most abundant duplex is highlighted. [file 12864_2015_1610_MOESM3_ESM.pdf]

```
>vvi-miR159c    Vvi17-      2609190:2609409
TGGATGAAATTAGGGTTTTGGAGTGGAGCTCCTTGAAGTCCAATAGAGGGTCTTACTGGGTAGATTGAGCTGCTGACTTATGGATCCCACAGCCCTATCCCGTCAATGGGGGGCATTGGATAGGCTTGTGGCTTGCATATCTCAGGAGCTGCATTATCCAAGTTTAGATCCTTGTTTGGATTGAAGGGAGCTCTACACCTCTCTCTCTGTCCATCATCAT
..((((...((((....((.((((((((((((..((((((..((((((((.(((((((((..((((((.((((..((((.(..((((((.(((((((.(((.......)))...))))))).))))))..).))))..)))).)))).))))))))).)))..))))))))..)))))))..))))))))))))).)).....))))).....)))))..
vvi-miR159c.1            GAGCTCCTTGAAGTCCAATAG (5376)                                                                                                 TTTGGATTGAAGGGAGCTCTA (3260544)
vvi-miR159c.2                                                AGCTGCTGACTTATGGATCCC (60183)                                CTTGCATATCTCAGGAGCTGC (113250)
```

```
>vvi-miR169w    Vvi14+      29685578:29685790
CTTTTCCTTCATCTGAGGTCTTGCATGTGGGGATGAGGTTATGTGGTGCAGCCAAGGATGACTTGCCGGCAACTCCCTTTATTGTACTCATGCATGCTCATACTTATACTGTTGTGGGCATCATTTAAGTGGCAAAAATGGTGGTCGGCGAGTCATTCTTAGCTACATTTCTGCCTCATCTTCTCATGCTAGATTTATGATGATAATGCCTAA
........(((((..((((((.(((((.(((((((((((.....((((.(((.((((((((((((((((....((.(((.(((((((.(((.((((((((((.......).)))))))))))))...))).))))))).))..))))))))))))))))).))).))))...))))))))))).))))).))))))..))))).........

vvi-miR169w.1            TGTGGGGATGAGGTTATGTGGTG 208                                                                                                TTTCTGCCTCATCTTCTCATG 386
vvi-miR169w                                        CAGCCAAGGATGACTTGCCGG 77
```
