# Supplementary material for: miRVine: a microRNA expression atlas of grapevine based on small RNA sequencing
Source: BMC Genomics. 2015 May 16;16(1):393. doi: 10.1186/s12864-015-1610-5 (PMC4434875; doi:10.1186/s12864-015-1610-5)
Supplement: Additional file 15: — Primers used for Real Time PCR (RT-qPCR). [file 12864_2015_1610_MOESM15_ESM.pdf]

Oligo Name	Sequence 5' to 3'
Rev universal	GTGCAGGGTCCGAGGT
168-1Rev	GAGCTGGGTCCGAGGT
166-1Rev	GTGCAGGGAGGGAGGT
397-Rev	GTCGAGGGTCCGAGGT
vvi-miR172c-RT	GTCGTATCCAGTGCAGGGTCCGAGGTATTCGCACTGGATACGACCTGCAG
vvi-miR172c-For	TGGCGGGAATCTTGATGATG
vvi-miR319c-RT	GTCGTATCCAGAGCTGGGTCCGAGGTATTCGCTCTGGATACGACAGGGAG
vvi-miR319c-For	ACTGCGTTGGACTGAAGGGAG
vvi-miR396c-RT	GTCGTATCCAGTGCAGGGTCCGAGGTATTCGCACTGGATACGACCAGTTC
vvi-miR396c-For	GGCGGTTCCACAGCTTTCTT
vvi-miR166g-RT	GTCGTATCCAGTGCAGGGAGGGAGGTATTCGCACTGGATACGACGGGGAA
vvi-miR166g-for	TCACTTCGGACCAGGCTTCA
vvi-miR156f-RT	GTCGTATCCAGTGCAGGGAGGGAGGTATTCGCACTGGATACGACGTGCTC
vvi-miR156f-For	CGGCGGTTGACAGAAGATAGA
vvi-miR2950-RT	GTCGTATCCAGTCGAGGGTCCGAGGTATTCCGACTGGATACGACTCCAGT
vvi-miR2950-For	TCGCCTTCCATCTCTTGCAC
vvi-miR390-RT	GTCGTATCCAGTGCAGGGTCCGAGGTATTCGCACTGGATACGACGGCGCT
vvi-miR390-For	TTGCGAAGCTCAGGAGGGAT
vvi-U6for	CCGATAAAATTGGAACGATACAGAG
vvi-U6rev	TCGATTTGTGCGTGTCATCCT
vvi-UBI For	AATGGTCAGTTGGCCCTACCT
vvi-UBI Rev	TGGCTGAGACCCACAAAACC

Supplementary Figure 1. Dot-bracket notation representing RNA stem-loop structure of some examples of miRNA loci generating more than one distinct duplex from the same precursor. For each precursor MIR locus and mapping position (chromosome, strand, coordinates) are presented. Below the dot-bracket notation, distinct miRNA duplexes are aligned and the sum of the abundance (TP5M) of the reads are shown in parenthesis. The most abundant duplex is highlighted.

```
>vvi-miR159c    Vvi17-      2609190:2609409
TGGATGAAATTAGGGTTTTGGAGTGGAGCTCCTTGAAGTCCAATAGAGGGTCTTACTGGGTAGATTGAGCTGCTGACTTATGGATCCCACAGCCCTATCCCGTCAATGGGGGGCATTGGATAGGCTTGTGGCTTGCATATCTCAGGAGCTGCATTATCCAAGTTTAGATCCTTGTTTGGATTGAAGGGAGCTCTACACCTCTCTCTCTGTCCATCATCAT
..(((((...(((((....((.((((((((((((..(((((((..((((((((.(((((((((..((((((.((((..((((.(..((((((.(((((((.(((.......)))...))))))).))))))..).))))..)))).)))).)))))))).)))..))))))))..)))))))..))))))))))))).)).....)))))....)))))..
vvi-miR159c.1          GAGCTCCTTGAAGTCCAATAG (5376)                                                                                                   TTTGGATTGAAGGGAGCTCTA (3260544)
vvi-miR159c.2                                                  AGCTGCTGACTTATGGATCCC (60183)                                        CTTGCATATCTCAGGAGCTGC (113250)
```

```
>vvi-miR169w    Vvi14+      29685578:29685790
CTTTTCCTTCATCTGAGGTCTTGCATGTGGGGATGAGGTTATGTGGTGCAGCCAAGGATGACTTGCCGGCAACTCCCTTTATTGTACTCATGCATGCTCATACTTATACTGTTGTGGGCATCATTTAAGTGGCAAAAATGGTGGTCGGCGAGTCATTCTTAGCTACATTTCTGCCTCATCTTCTCATGCTAGATTTATGATGATAATGCCTAA
........(((((..((((((.(((((.(((((((((((.....((((.(((.((((((((((((((((((....((.(((.((((((.(((.((((((((((.......).)))))))))))...))).))))))).)).)))))))))))))))))).))).)))).)...)))))))))).))))).))))))..))))).........

vvi-miR169w.1          TGTGGGGATGAGGTTATGTGGTG 208                                                                                    TTTCTGCCTCATCTTCTCATG 386
vvi-miR169w                                   CAGCCAAGGATGACTTGCCGG 77
```

Supplementary Figure 2: Number of putative miRNA target within the (A) PN40024 and (B) Corvina data sets, identified using the TargetFinder (release 1.6). Novel and known refer to the differente classes of miRNAs.

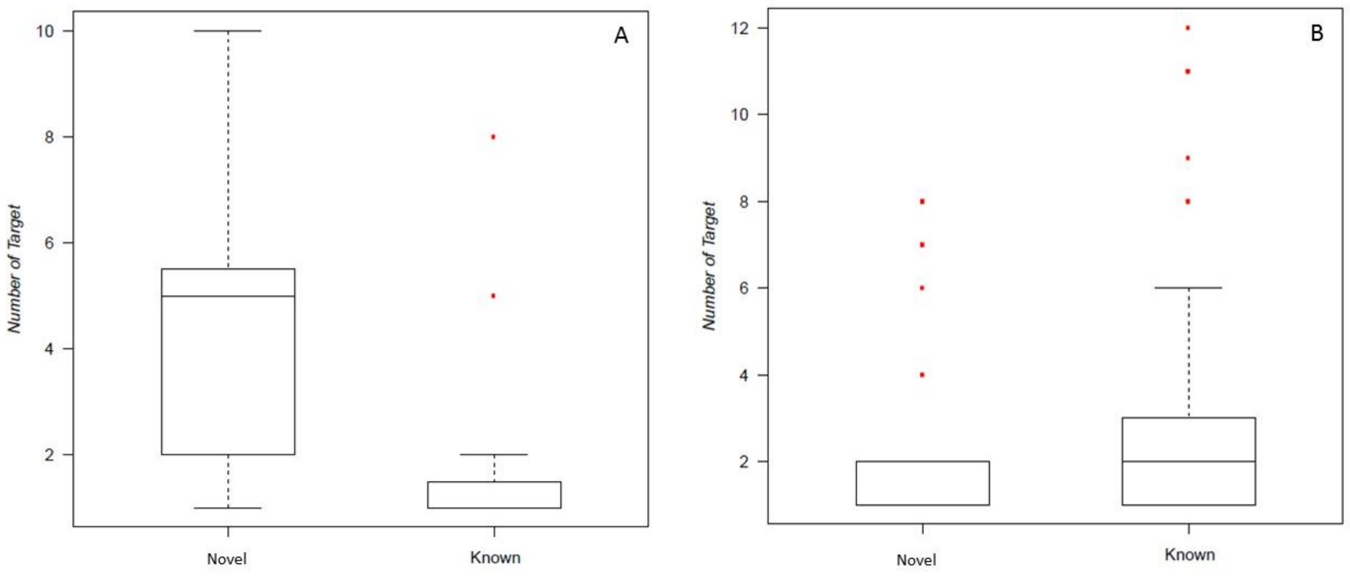

Supplementary Figure 3: Dendrogram of samples (columns) and miRNAs (rows) using log normalized data after replicates averaging.

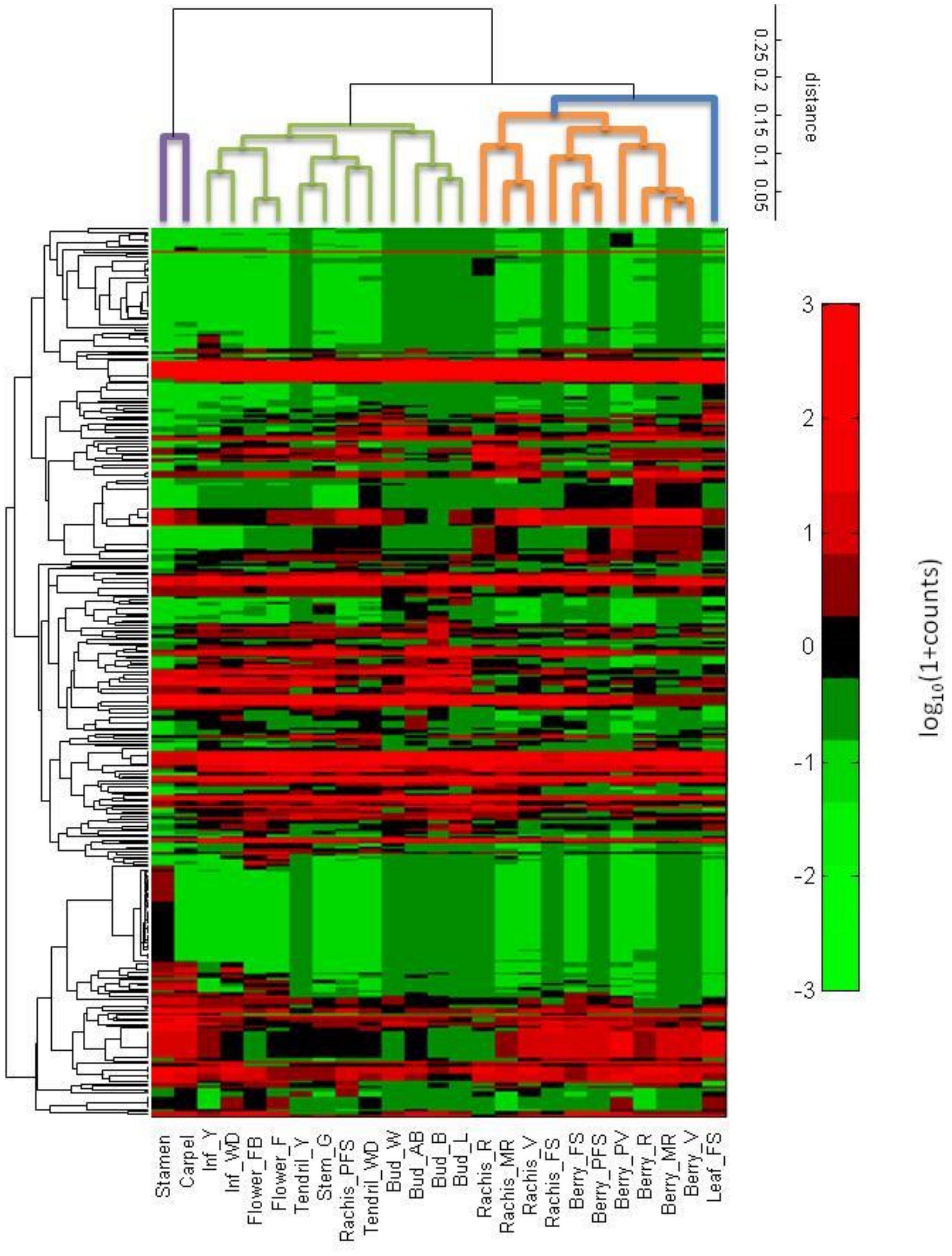

Methods S1: Supplementary Plant material

Buds were collected at five developmental stages: namely first season latent bud (E-L 23), winter dormant bud (E-L 1), bud, burst showing green tip (E-L 4) and final bud stage after burst, (E-L 5). Inflorescences were collected at two developmental stages: young inflorescence with single flower in compact groups (E-L 14), and well-developed inflorescence with single flower separated (E-L 17). Flowers were collected at the beginning of flowering (E-L 20) and flowering phase (E-L 23); flower organs were collected from undisclosed flowers. Pollen was collected from disclosed flowers at 80% caps off (E-L25).

Tendrils were collected from two developmental stages: the first when the shoot bears seven separate leaves (E-L 14) and the second corresponding to a pool of well-developed tendrils collected when the shoot bears 12 separate leaves (E-L 17). Leaves were collected at three developmental stages: young leaves when the shoot contains about five well-separated leaves (EL14), mature leaves when the berry size was about 4 mm in diameter (E-L 29), and leaves in senescence, collected before the beginning of leaf fall (E-L43).

Berries were collected at six developmental time points by freezing the whole berry and removing the seeds. The six stages are fruit set (FS), 15 days after flowering (DAF; E-L29), post fruit set (PFS), when berries reach >7 mm diam. (E-L 31), pre-veraison (E-L 34), veraison (V), when berries change color (10.4 °Brix,70 DAF; E-L35), mid-ripening (MR) stage (84 DAF; E-L 36,15.5 °Brix) and the last stage ripening (R) (115 DAF; E-L 38, 15.5 °Brix). For post-harvest samples, bunches were placed for three months in single layers in naturally ventilated rooms, without any artificial control of temperature and humidity. Samples were collected every month. Withering stage I was characterized by a weight reduction of 23.6% and 24.5 °Brix, the second stage by weight reduction of 30.3% and 25.9 °Brix and the last stage by a weight reduction of 32.7% and 26.7 °Brix.
